# Siderophore Activity of Partially Acetylated Fusarinines from the Sponge-Derived Fungus *Pseudogymnoascus verrucosus*

**DOI:** 10.1021/acs.jnatprod.5c00399

**Published:** 2025-07-10

**Authors:** Mariana Montanares, Carlos Jiménez, Jaime Rodríguez, Anaí Diaz, Lucía Ageitos, Renato Chávez, Inmaculada Vaca

**Affiliations:** † Departamento de Química, Facultad de Ciencias, 14655 Universidad de Chile, Las Palmeras 3425, Ñuñoa, 1025000 Santiago, Chile; ‡ CICA - Centro Interdisciplinar de Química e Bioloxía, Departamento de Química, Facultade de Ciencias, Universidade da Coruña, 15071 A Coruña, Spain; § Departamento de Biología, Facultad de Química y Biología, Universidad de Santiago de Chile (USACH), Alameda 3363, Estación Central, 9170022 Santiago, Chile

## Abstract

*Pseudogymnoascus* is a fungal genus prevalent in Antarctica whose metabolism, including the types of siderophores it synthesizes, remains largely unexplored. In this work, we investigated the siderophores produced by *Pseudogymnoascus verrucosus* FAE27, a strain isolated from the Antarctic marine sponge *Hymeniacidon* sp., grown under iron-limiting conditions. Two new hydroxamate-type siderophores, *N,N*′-diacetyl-fusarinine B (**7**) and *N*-acetyl-fusarinine A (**8**), are described here as the first naturally occurring partially acetylated fusarinines to date, along with the known fusarinine (**1**) and five of its analogues: *N*-acetyl-fusarinine (**2**), *N*-acetyl-fusarinine-methylester (**3**), *N,N′,N″-*triacetyl-fusarinine C (**4**), *N,N′*-diacetyl-fusarinine A (**5**), and *N,N′,N″*-triacetyl-fusarinine B (**6**). A comprehensive NMR spectroscopic characterization of their Ga^3+^ complexes was conducted. Surprisingly, the full spectroscopic characterization of most of these known siderophores was either incomplete or absent in the literature. The analysis of ^1^H, ^13^C, and 2D NMR data provided significant structural insights, including the impact of Ga^3+^ coordination on chemical shifts. Additionally, the absolute configuration of the amino acid units in compounds **1–8** was determined via acid hydrolysis and Marfey’s analysis. Finally, the siderophore activity of all purified compounds was confirmed by their ability to promote fungal growth under iron-limiting conditions.

## Graphical abstract

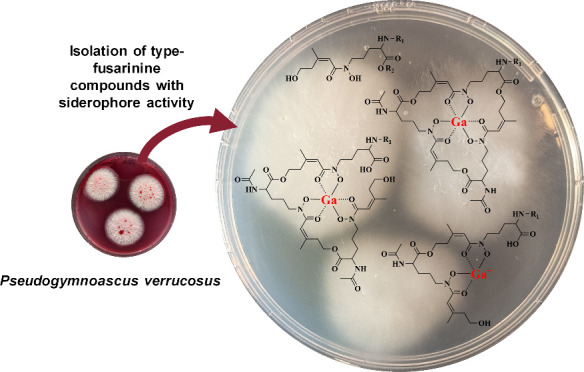

Siderophores are small metabolites (200–2000 Da) secreted mainly by microorganisms and plants, distinguished by their high-affinity for ferric iron.^1^ As in all siderophore-producing organisms, these compounds are essential for fungal development, as they facilitate the efficient iron acquisition in iron-deficient environments.^2^ Despite their functional importance, the siderophores produced by numerous fungal species, particularly those isolated from extreme environments, remain chemically uncharacterized.

The chemical diversity of siderophores synthesized by microorganisms from Antarctic environments remains largely unexplored. Previous studies on siderophore production by bacteria and yeasts collected in Antarctica have primarily focused on their biotechnological potential, as well as on the analysis of genes involved in siderophore biosynthesis or transport.^3–8^ However, the specific siderophores implicated in these studies have yet to be identified. To our knowledge, the only study addressing the isolation and structural characterization of siderophores from microorganisms from Antarctica was the identification of a novel pyoverdine from *Pseudomonas fluorescens* strain 51W.^9^ This knowledge gap is even more pronounced for siderophores produced by filamentous fungi of Antarctic origin.

As part of our ongoing investigations into the metabolism of *Pseudogymnoascus* strains isolated from marine sponges collected in the Antarctica,^10,11^ and the siderophores produced by marine microorganisms,^12–16^ we have focused on the siderophores biosynthesized by *P. verrucosus* FAE27, a strain isolated from a *Hymeniacidon* sp. sponge.^17^ *Pseudogymnoascus* is a prevalent fungal genus in Antarctic environments,^18^ suggesting that it plays a significant ecological role. Additionally, strains of this genus have been isolated from other cold environments worldwide, such as tundra, high mountains, and caves.^19–21^ Despite its broad distribution, knowledge of this genus remains limited, with only 24 species described to date.^22^ The chemical characterization of *Pseudogymnoascus*-derived metabolites has been scarcely explored, with most reported compounds originating from unclassified strains.^22–24^ To date, the only study on *Pseudogymnoascus verrucosus* metabolites was an analysis of volatile compounds published by Wilson and Forse in 2018.^24^ Therefore, a significant gap remains in the understanding of the specialized metabolites produced by *Pseudogymnoascus verrucosus.*

Among all known *Pseudogymnoascus* species, *P. destructans* is the only species in the genus for which siderophore production has been reported.^23^ Specifically, ferrichrome and *N,N′,N″*-triacetyl-fusarinine C (**4**, TAFC) have been detected in the wings of bats infected with *P. destructans* and in axenic cultures of the fungus.^23^

The present study aims to analyze the siderophores produced by the *P. verrucosus* FAE27, an isolate of Antarctic origin. Eight fusarinine-type hydroxamate siderophores, including three monomers, two dimers, and three trimers, in their *apo* (unchelated) or *holo* (Ga^3+^ chelated) forms, were isolated from the fermentation broth of this strain under iron-limiting conditions. Two of these, named *N,N′*-diacetyl-fusarinine B (**7**) and *N*-acetyl-fusarinine A (**8**), have not been reported previously and represent the first naturally occurring, partially acetylated fusarinines, described to date. The remaining six fusarinine derivatives were identified as known siderophores: fusarinine (**1**), *N*-acetyl-fusarinine (**2**), *N*-acetyl-fusarinine-methylester (**3**), *N,N′,N″*-triacetyl-fusarinine C (**4**), *N,N′,N″*-triacetyl-fusarinine B (**5**), and *N,N′*-diacetyl-fusarinine A (**6**).

Existing studies lack detailed nuclear magnetic resonance (NMR) data even for previously reported compounds, with spectral assignments being fragmentary or notably absent in the literature. This work addresses this gap by providing complete (1D/2D NMR, HRESIMS) data sets for all isolated metabolites, including known fusarinines, whose structural elucidation had relied on incomplete comparisons or indirect methods. By resolving these ambiguities, we establish a foundational reference for future studies while revealing acetylation-driven structural nuances that are critical for understanding siderophore function in microbial systems. Finally, the siderophore activity of the isolated compounds was evaluated.

## Results and Discussion

### Identification and Isolation of Fe- and Ga-Chelating Compounds from *P. verrucosus* Cultures

The fungal strain *P. verrucosus* FAE27, isolated from a *Hymeniacidon* sp. sponge collected in Antarctica,^17^ was cultured under iron-deficient conditions. The chrome azurol S (CAS) assay of the culture broth showed the characteristic color change from blue to orange indicating the presence of siderophores. The culture was then filtered to remove the mycelium, followed by centrifugation to obtain a clarified broth, which was divided into two portions. To stabilize the siderophores, each portion was incubated with either Fe^3+^ or Ga^3+^ salts and subsequently extracted with XAD-16 resins. The CAS positive containing fractions were eluted with methanol and the resulting residues, obtained after solvent evaporation, were analyzed by LC/HRMS. The unique isotopic cluster patterns of the Ga^3+^ and Fe^3+^ complexes observed in their HRESIMS spectra allowed both, the detection and identification of the Fe- and Ga-chelated compounds.

As shown in Table 1, all detected complexes were identified as members of the hydroxamate siderophore family, specifically fusarinines and ferrichrome. Among the fusarinines, a diacetylated dimer (**6**) and triacetylated trimers in both open-chain and cyclic forms (**4**-**5**) as Fe^3+^ and Ga^3+^ complexes were detected. In addition to the known siderophores, two compounds were detected with *m*/*z* values that did not match any compound reported in public databases or in the literature. These findings suggest that these compounds may represent potential new siderophores. To establish the structures of the putative new compounds, the culture was scaled up under iron-deficient conditions, incubated with Ga^3+^ salts to stabilize the siderophores as Ga^3+^ complexes, and to study them by nuclear magnetic resonance (NMR). To evaluate structural differences between chelated and nonchelated siderophores, another culture was prepared without metal supplementation. After incubation, the resulting mixtures were lyophilized to obtain the corresponding residues that were then extracted with MeOH. Once the solvent was removed, the methanolic extracts were submitted to HPLC separation using a DAD detector, yielding several fractions containing the purified hydroxamate compounds. Finally, their structure elucidation was carried out by (+)-HRESIMS and NMR data analysis (Tables S1–S31 and Figures S1–S143).

**Table 1:** Calculated and Observed *m*/*z* Values in the LC/(+)-HRESIMS Analysis of the Compounds Detected as Ga^3+^ and Fe^3+^ Complexes from Culture Broths of *P. verrucosus* FAE27 Grown in M9 Medium

	[M – 2H + Fe]^+^ ion^a^		[M – 2H + ^69/71^Ga]^+^ ions^b^	
Assignment	Observed *m*/*z*	Calculated *m/z*	Observed *m*/*z*	Calculated *m/z*
n.f.^c^	598.1935		611.1827/613.1816	
*N,N*′-diacetyl-fusarinine A	640.2046	640.2038	653.1936/655.1930	653.1950/655.1941
Ferrichrome	741.2383	741.2375	754.2275/756.2269	754.2282/756.2273
n.f.^c^	882.3314		895.3213/897.3209	
*N,N′,N*″*-*triacetyl-fusarinine B	924.3425	924.3410	937.3318/939.3316	937.3322/939.3313
*N,N′,N*″-triacetyl-fusarinine C	906.3320	906.3304	919.3210/921.3209	919.3217/921.3208

a In culture broths supplemented with Fe^3+^.

b In culture broths supplemented with Ga^3+^.

c Data corresponding to these were not found in the literature.

### Planar Structures for Compounds **1–8**

Although fusarinine-type siderophores have been known for over 50 years,^25^ the detailed spectroscopic data of their dimers and trimers, aside from partial data on TAFC, remain scarce in the literature. Traditionally, these compounds have been structurally characterized by hydrolyzing TAFC and fusarinine C,^26,27^ followed by spectroscopic analysis of the resulting monomers, along with crystallographic analysis of a TAFC-Fe^3+^ complex.^28^ The structure identification of dimers and trimers has primarily relied on mass spectrometry.^29,30^ This study aims to address this gap by providing a comprehensive spectroscopic characterization of both known and novel siderophores, including their gallium complexes. These not previously described gallium complexes may offer valuable insights into the three-dimensional structures they adopt when they are chelated with iron in their natural environment.

Comparison of the NMR and (+)-HRESIMS spectral data of **1** to those reported for the monomer *cis*-fusarinine^31,32^ allowed us to confirm the presence of this compound (Tables S2–S3 and Figures S1–S8).

The ^1^H NMR spectrum of compound **1** revealed three spin systems characteristic of the ornithine moiety. The first system consists of three methylene and a single α-methine group. This assignment was supported by a ^1^H–^1^H COSY experiment, which demonstrated connectivity from the α-CH signal (H-2), observed as a triplet at δ_H_ 3.63, to the β-CH_2_ group (H_2_-4) at δ_H_ 1.84/1.90, then to the γ-CH_2_ (H_2_-5) at δ_H_ 1.84/1.79, and finally to the δ-CH_2_ at δ_H_ 3.69 (H_2_-6). Two additional spin systems were identified: one comprising two methylene groups indicative of a side chain, and another corresponding to a *Z*-alkene moiety. Furthermore, two other spin systems were characterized: a homoallylic methylene group, represented by two protons at δ_H_ 3.72 (t, *J* = 6.4 Hz, H_2_-12) and δ_H_ 2.71 (bt, H_2_-11), and the trisubstituted *Z*-alkene with proton H-9 at δ_H_ 6.37 (broad singlet), along with a methyl group at δ_H_ 1.92 (broad singlet, H_3_–16). These groups were connected through HMBC correlations: from H_2_-12 to C-10 (δ_C_ 152.29), and from H-9 to C-16 (δ_C_ 25.16). These data collectively support the proposed structure of the anhydromevalonic moiety of fusarinine.

Compounds **2** and **3** were identified as *N*-acetyl-fusarinine,^33^ and *N*-acetylfusarinine-methylester,^34^ respectively, by the analysis of their (+)-HRESIMS along with their 1D and 2D NMR spectroscopic data (Tables S4–S5 and Figures S11–S18, and Tables S6–S7 and Figures S21–S28, respectively). As far as we know, the NMR data for **3** have not been reported yet.

Compound **4** was isolated as a Ga^3+^ complex (**4-Ga**), as indicated by the characteristic isotopic cluster of ^69^Ga/^71^Ga at *m*/*z* 941.3059/943.3063 [M-3H+^69/71^Ga+Na]^+^ in a 2:1 ratio, which matches with the molecular formula C_39_H_60_N_6_O_15_ (13 degrees of unsaturation) for *N,N′,N″*-triacetyl-fusarinine C (TAFC) coordinated with Ga^3+^ (Figure 1, Tables S8–S9 and Figures S31–S38). The ^1^H and ^13^C NMR data (Table 2, Table S9) of **4-Ga** were similar to those reported for *apo*-TAFC (**4**).^35–39^ This is the first time that spectroscopic data of the gallium complex TAFC (**4**-**Ga**) have been reported (Figure 2, Table S9 and Figures S31–S38).

**Table 2:** NMR Data for *N,N′,N″*-Triacetyl-fusarinine C (TAFC) Gallium Complex (**4-Ga**), *N,N′,N″*-Triacetyl-fusarinine B (TAFB) Gallium Complex (**5-Ga**), and *N,N′*-Diacetyl-fusarinine B (DAFB) Gallium Complex (**7-Ga**) in CD_3_OD

	**4-Ga**		**5-Ga**		**7-Ga**	
Position	δ_C_ mult	δ_H_ mult (*J* in Hz)	δ_C_ mult	δ_H_ mult (*J* in Hz)	δ_C_ mult	δ_H_ mult (*J* in Hz)
2	52.01, CH	4.34, dd (9.6, 3.0)	54.28, CH	4.33–4.27, m	55.41, CH	4.05, t (6.0)
2′					52.87, CH	4.39, t (5.9)
2″						
3	173.58, C		174.17, C		171.72, C	
3′			174.07, C			
3″			173.97, C			
4	28.12, CH_2_	1.53–1.48, m	29.67, CH_2_	1.79–1.87, m	28.54, CH_2_	1.85–1.74, m
4′		2.07–2.00, m			29.30, CH_2_	
4″					29.94, CH_2_	2.05–2.02 m
5	23.26, CH_2_	1.53–1.48, m	25.48, CH_2_	1.55–1.79, m	21.40, CH_2_	1.85–1.74, m
5′		2.07–2.00, m			22.31, CH_2_	
5″						2.05–2.02, m
6	49.80, CH_2_	3.54 tt (13.6, 3.7)	51.82, CH_2_	3.69–3.63, m	51.41, CH_2_	3.30–3.18, m
6′		4.09, tt (10.1, 3.5)			51.28, CH_2_	4.01–3.83, m
6″						
8	161.57, C		162.66, C		161.12, C	
8′					162.13, C	
8″						
9	116.03, CH	6.11, s	114.75, CH	6.08–6.11, m	116.53, CH	6.18, s
9′				6.16–6.19, m	113.73, CH	6.22, s
9″						
10	151.42, C		153.99, C		153.79, C	
10′						
10″						
11	34.23, CH_2_	2.20, m	34.08, CH_2_	2.06–2.13, m	32.65, CH_2_	3.43–3.32, m
11′		3.54, tt (13.6, 3.7)	38.17, CH_2_	2.84–2.57, m	34.12, CH_2_	3.30–3.18, m
11″					38.34, CH_2_	3.18–2.46, m
12	62.65, CH_2_	3.99, ddd (10.9, 6.9, 3.6)	63.71, CH_2_	4.11–4.07, m	65.78, CH_2_	
12′			62.12, CH_2_	4.33–4.27, m	67.63, CH_2_	4.29–4.10, m
12″		4.72, tt (8.3, 2.6)		3.79–3.74, m	61.59, CH_2_	3.83–3.43, m
14	172.55, C		173.82, C		172.96, C	
14′					173.66, C	
14″						
15	22.26, CH_3_	1.98, s	22.29, CH_3_	1.96, s	22.29, CH_3_	2.00, s
15′			22.68, CH_3_		22.35, CH_3_	2.01, s
15″			22.77, CH_3_			
16	23.55, CH_3_	1.97, d (1.4)	23.13, CH_3_	1.99, d (1.5)	24.07, CH_3_	1.97, d (1.4)
16′					24.39, CH_3_	
16″						

**Figure 1:**
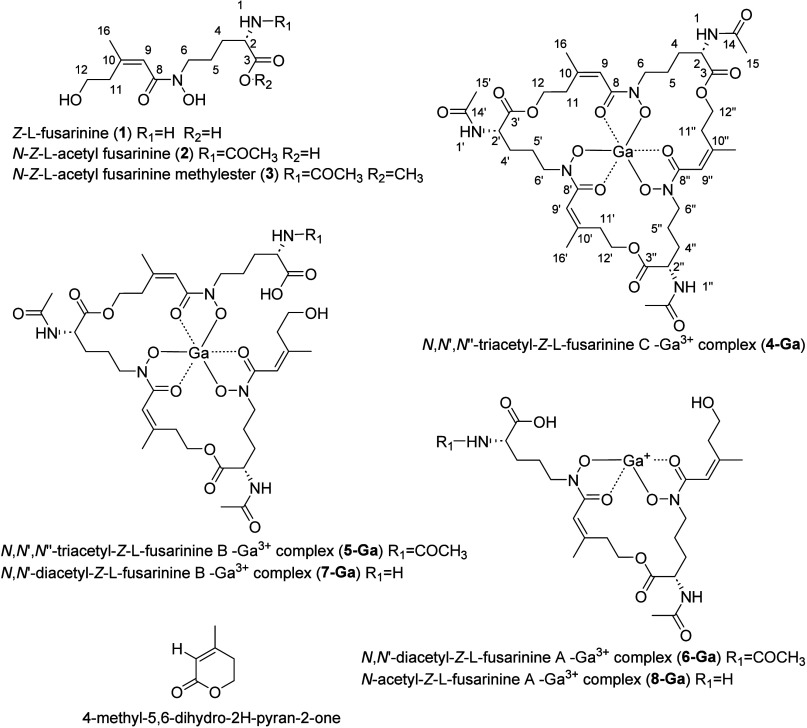
Structures of **1**–**8** isolated from *Pseudogymnoascus verrucosus* FAE27.

**Figure 2:**
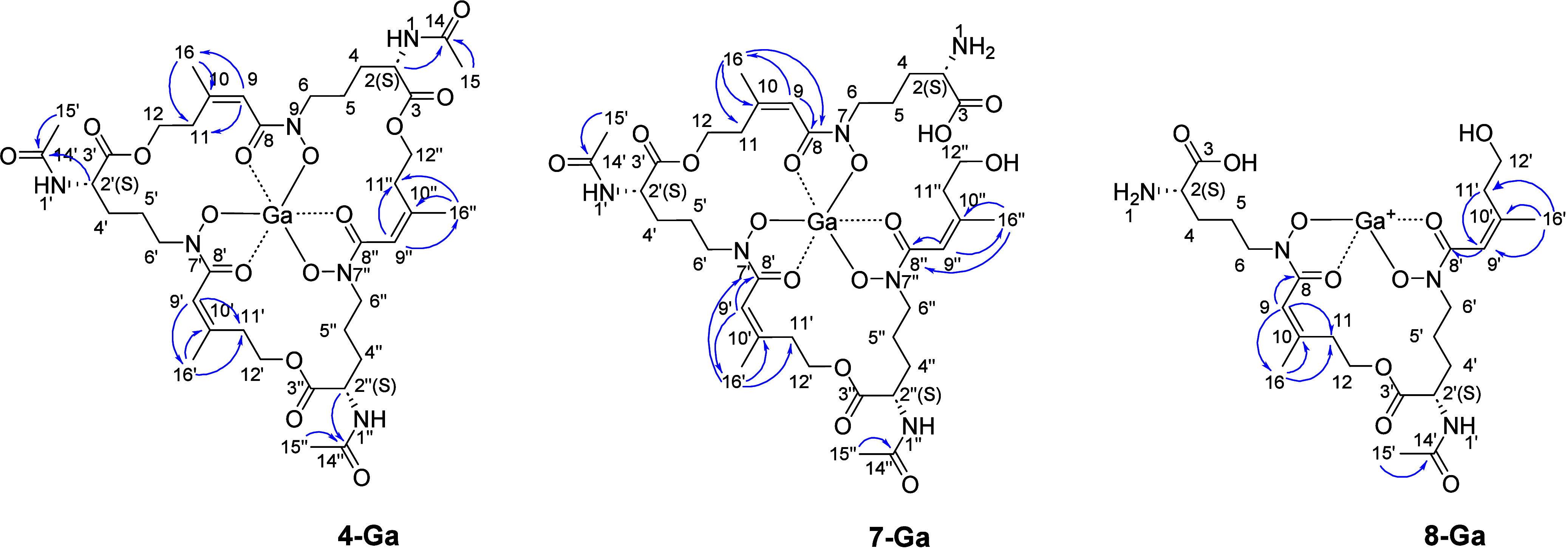
^1^H–^1^H COSY(−) and ^1^H–^13^C HMBC (−) correlations of *N,N′,N″*-triacetyl-fusarinine C gallium complex (**4-Ga**), *N,N′-*diacetyl-fusarinine B gallium complex (**7-Ga**), and *N*-acetyl-fusarinine A gallium complex (**8-Ga**).

The ^13^C NMR chemical shift of C-8 at δ_C_ 161.57 in the *holo*-TAFC form (**4-Ga**) at lower ppm relative to that of the *apo*-TAFC form (**4**) at δ_C_ 172, is consistent with coordination of the hydroxamic oxygen atoms to Ga^3+^ in the gallium complex. On the other hand, the ^1^H NMR spectrum of *apo-*TAFC (**4**) displayed a triplet at δ_H_ 2.64 which was assigned to the two methylene protons at position C-11.^38^ In contrast, the HMBC spectrum of the TAFC gallium complex **4-Ga** (Figure S38, Figure 2) showed two signals, a multiplet centered at δ_H_ 2.20 and a triplet of triplets at δ_H_ 3.54, corresponding to a pair of methylene protons that correlated with C-11 (δ_C_ 34.23).

A similar pattern was observed for the methylene protons at position C-12: a multiplet centered at δ_H_ 4.18 was assigned to C-12 in *apo-*TAFC (**4**) whereas the HMBC spectrum of the TAFC-gallium complex (**4-Ga**) displayed a doublet of doublet of doublets centered at δ_H_ 3.99 and a triplet of triplets at δ_H_ 4.72 that showed correlations with C-12 (δ_C_ 62.65). These spectroscopic data supported the planar structure of TAFC-gallium complex (**4-Ga**) which was similar to that proposed for TAFC-iron complex (**4-Fe**) by crystallographic analysis.^28^ The symmetric conformation of *apo*-TAFC (**4**) explains the multiplicity of the proton chemical shift signals of the methylene protons at C-12 in its ^1^H NMR spectrum.

The molecular formula of **5-Ga** was established as C_37_H_57_N_6_O_15_Ga (13 degrees of unsaturation) based on the molecular ion peaks observed at *m*/*z* 959.3139/961.3139 [M-3H+^69/71^Ga+Na]^+^ in its (+)-HRESIMS (Table S10 and Figures S39–S40) and supported by its NMR data (Table 2, Table S11 and Figures S41–S47). These data showed an increase of 18 units in the molecular weight of **5**-**Ga** compared to that of **4**-**Ga**. MS and NMR data analysis suggested that **5-Ga** is *seco*-**4-Ga**, a derivative in which one of the three ester bonds has been hydrolyzed. This compound was identified as the *N,N′,N″*-triacetyl-fusarinine B -Ga^3+^ complex.

The ^1^H, ^13^C, and 2DNMR spectroscopic data of **5-Ga** were quite similar to those of **4-Ga**, except for notable differences in specific chemical shifts. The methylene carbon chemical shifts at positions C-11/11′/11″ and C-12/12′/12″ in the ^13^C NMR spectrum of **4-Ga** exhibited a single signal at δ_C_ 34.23 and δ_C_ 62.65, respectively, indicating similar environments. In contrast, the ^13^C NMR of **5-Ga** displayed two distinct signals for C-11/11′/11″ (δ_C_ 34.08 and δ_C_ 38.17) and two additional signals for C-12/12′/12″ (δ_C_ 62.12 and δ_C_ 63.71). Proton integration analysis, combined with the HMBC spectrum of **5-Ga** (Figure S47), revealed that the signals at higher ppm (δ_C_ 38.17 for C-11 and δ_C_ 63.71 for C-12) corresponded to carbons bonded to two protons each, while the signals shifted at lower ppm (δ_C_ 34.08 for C-11′/C-11″ and δ_C_ 62.12 for C-12′/C-12″) corresponded to carbons bonded to four protons each. This pattern was consistent with the following structural arrangement: carbon positions 11, 11′, 12, and 12′ in **5-Ga** are adjacent to an ester bond, as in **4-Ga**, whereas positions 11″ and 12″ in **5-Ga** belong to the open-chain region containing the terminal hydroxyl group, as observed in the monomers **1–3**. On the other hand, carbon positions C-8, C-8′, and C-8″ (δ_C_ 162.66) in the TAFC gallium complex (**4-Ga**) resonated at lower ppm (δ_C_ 169) relative to the counterparts in *apo-*TAFB (**5**) (Table S13 and Figures S48–S56). A similar shift to lower ppm was observed for other carbons near the hydroxamic group: in *apo-*TAFB (**5**), carbon positions C-9, C-9′and C-9″ resonated at δ_C_ 118.94 whereas these signals shifted to lower ppm (δ_C_ 114.75) in the TAFB gallium complex (**5-Ga**). This shifted effect was consistent with the coordination of the oxygens of the hydroxamic groups to gallium. On the other hand, when the ^1^H NMR of the *apo-*TAFB (**5**) was recorded in (CD_3_)_2_SO (Table S14 and Figures S57–S63), different values of the chemical shift of NH-1 at δ_H_ 7.54–7.45 (1H) relative to those of NH-1′ and NH-1″ at δ_H_ 8.28 (d, *J* = 7.4 Hz, 2H), confirmed that the macrolactone ring of **4** is opened in **5**.

Finally, the strong diastereotopicity observed in the chemical shifts of H11/11′ and 12/12′ in the *holo* form in **5-Ga**, is most likely attributable to the asymmetry introduced by ring opening (relative to **4-Ga**), rather than to Δ/Λ isomerism.

The molecular formula of **6-Ga** was determined as C_26_H_39_N_4_O_11_Ga (10 degrees of unsaturation) based on the [M – 2H + ^69/71^Ga]^+^ ion peaks at *m*/*z* 653.1948/655.1942 in its (+)-HRESIMS (Table S15 and Figures S64–S65), further supported by its NMR data (Table 3, Table S16 and Figures S66–S72). These findings suggested the presence of two fusarinine units, indicating that **6-Ga** is a diacetylated dimer of **1**. Analysis of the COSY and HSQC spectra of **6-Ga** (Figures S70–S71) revealed two distinct −CH_2_–CH_2_– systems at positions C-11/C-12 and C-11′/C-12′. The first system exhibited δ_H_/δ_C_ signals for the methylene groups of 2.70–2.67 and 2.77–2.71/37.42 at position C-11 and δ_H_/δ_C_ 3.69–3.61/70.63 at position C-12. The second system displayed δ_H_/δ_C_ signals for the methylene groups of 2.65–2.56 and 2.77–2.71/38.03 at position C-11′ and of δ_H_/δ_C_ 3.71/61.37 at position C-12′. Based on these chemical shifts, the first −CH_2_–CH_2_– system was assigned to the unit C-11/C-12 adjacent to the ester bond, linking the two monomers, while the second corresponded to the -CH_2_–CH_2_– system C-11′/C-12′ linked to the terminal hydroxyl group. Further analysis of the *apo* form of **6** (Table S18 and Figures S75–S81) revealed modifications at positions C-6, C-8 and C-9 in relation to **6-Ga**, analogous to those observed in **5-Ga** when comparing its *apo* and *holo* (chelated with gallium) forms. In the *apo* form of **6**, the chemical shifts were δ_C_ 47.03 for C-6, δ_C_ 168.73 and δ_C_ 169.46 for C-8, and 118.96 for C-9. Upon gallium coordination distinct signals appeared, indicating a less symmetrical spatial distribution in **6-Ga** (δ_C_ 48.55 and 51.90 for C-6, δ_C_ 162.93 and 163.23 for C-8, and δ_C_ 119.12 and 114.44 for C-9). The ^1^H NMR spectrum of **6** in (CD_3_)_2_SO (Table S19 and Figures S82–S83) showed signals for the NH protons at δ_H_ 7.59–7.64 (1H) and δ_H_ 8.31 (1H). COSY correlations (Figure S86) assigned δ_H_ 3.96–4.02 to the proton at the α position (H-2) of the first monomer and δ_H_ 4.13–4.19 to that of the α position (H-2′) of the second monomer. Collectively, these data unambiguously identify compound **6-Ga** as *N,N*-diacetyl-fusarinine A (DAFA) gallium complex.

**Table 3:** NMR Data for *N,N′-*Diacetyl-fusarinine A (DAFA) Gallium Complex (**6-Ga**) and *N*-Acetyl-fusarinine A (AFA) Gallium Complex (**8-Ga**) in CD_3_OD

	**6-Ga**		**8-Ga**	
Position	δ_C_^a^ mult	δ_H_^b^ mult (*J* in Hz)	δ_C_^a^ mult	δ_H_^b^ mult (*J* in Hz)
2	55.45, CH	4.29–4.26, m	53.60, CH	3.89–3.86, m
2′	54.89, CH	4.31–4.29, m	52.67, CH	4.44–4.40, m
3	177.76, C		173.58, C	
3′	178.15, C		173.26, C	
4	29.94, CH_2_	1.64–1.74, m	28.88, CH_2_	1.77–1.60, m
4′	20.70, CH_2_	1.88–1.79, m	29.24, CH_2_	
5	24.42, CH_2_	1.64–1.74, m	24.38, CH_2_	1.77–1.60, m
5′		1.88–1.79, m		
6	48.55, CH_2_	3.69–3.61, m	50.59, CH_2_	3.64–3.61, m
				3.82–3.78, m
6′	51.90, CH_2_	2.85–2.74, m	50.73, CH_2_	3.56–3.54, m
				3.89–3.86, m
8	162.93, C		160.63, C	
8′	163.26, C		161.18, C	
9	119.12, CH	6.35, d (16.1)	116.78, CH	6.03, s
9′	114.44, CH	6.12, d (20.0)	113.01, CH	6.10, s
10	152.22, C		149.29, C	
10′	152.54, C		154.07, C	
11	37.42, CH_2_	2.70–2.67, m	34.46, CH_2_	2.43–2.37, m
		2.77–2.71, m		2.91–2.87, m
11′	38.03, CH_2_	2.65–2.56, m	39.24, CH_2_	2.62–2.59, m
		2.77–2.71, m		2.73–2.70, m
12	70.63, CH_2_	3.69–3.61, m	62.61, CH_2_	3.95–4.00, m
				4.61–4.55, m
12′	61.37, CH_2_	3.71, t (6.4)	61.06, CH_2_	3.48–3.47, m
				3.53–3.51, m
14	172.84, C		173.59, C	
14′				
15	22.77, CH_3_	1.987, s	22.44, CH_3_	2.00, s
15′	22.68, CH_3_	1.994, s		
16	25.55, CH_3_	1.93, d (1.5)	23.07, CH_3_	1.93, m
16′	25.25, CH_3_	1.97, d (1.4)	26.43, CH_3_	1.90, m

The structure of **7-Ga** was identified as the gallium complex of *N,N′*-diacetyl-fusarinine B, with the molecular formula of C_37_H_57_N_6_O_15_Ga (13 degrees of unsaturation). This assignment was based on the [M – 2H + ^69/71^Ga]^+^ ion peaks at *m*/*z* 895.3231/897.3234 observed in its (+)-HRESIMS (Table S20 and Figures S89–S90). A 42 Da difference in the molecular mass of **7**-**Ga** compared to that of **5**-**Ga** suggested the absence of an acetyl group in one of the three monomers present in **7**-**Ga**. Its HMBC data allowed the assignment of methylene groups at C-11/11′/11″ and C-12/12′/12″ positions in each of the three monomers (Table 2, Table S21, Figure 2). Additionally, the ^1^H NMR spectrum of **7-Ga** in (CD_3_)_2_SO (Table S22 and Figures S97–S103) revealed two distinct α-CH signals. The first monomer, bearing the terminal carboxylic acid, exhibited proton chemical shift signals for H-2 at δ_H_ 3.93–3.91 (δ_C_ 51.82/C-2) that correlated via COSY with NH_2_ protons at δ_H_ 8.26–8.22 (Figure S101). The second and third monomers displayed α-CH signals at δ_H_ 4.23 and 4.17 (δ_C_ 51.59/C-2′ and C-2″) that correlated with NH-1′ and NH-1″ protons at δ_H_ 8.26–8.22 and 8.37–8.34, respectively. These spectroscopic data confirmed that the macrolactone ring of **4**-**Ga** is open in **7**-**Ga** and the *N*-acetyl group next to the carboxylic acid-containing terminal monomer is missing.

The structure of compound **8-Ga** was determined to be the gallium complex of *N*-acetyl-fusarinine A, with a molecular formula of C_24_H_37_N_4_O_10_Ga (9 degrees of unsaturation). This assignment was based on the [M – 2H + ^69/71^Ga]^+^ ion peaks at *m*/*z* 611.1838/613.1830 observed in its (+)- HRESIMS (Table S24 and Figures S106–S107) and further supported by its NMR data (Table 3, Figure 2, Table S25). Again, a 42 Da difference in the molecular mass of **8**-**Ga** compared to that of **6**-**Ga** suggested the absence of an acetyl group in one of the two monomers present in **8**-**Ga**. HMBC data of **8-Ga** revealed two distinct pairs of methylene signals at positions C-11/11′ and C-12/12′ (Figure 2, Figure S113).

COSY correlations (Figure 2, Figure S111) allowed the assignment of the methylene signals (H_2_-11 and H_2_-12) adjacent to the ester group to those located near the hydroxyl group (H_2_-11′ and H_2_-12′). Additionally, the ^1^H NMR spectrum of **8-Ga** in (CD_3_)_2_SO (Table S26 and Figures S114–S120) displayed two distinct α-CH signals. The first α-CH signal, observed at δ_H_/δ_C_ 3.58–3.57/51.74, correlated via HMBC (Figure S120) with the carboxylic acid group (C-2) at δ_C_ 174.74. The second α-CH signal, at δ_H_/δ_C_ 4.27–4.22/51.19, exhibited COSY correlations (Figure S118) with the NH proton at δ_H_ 8.29–8.17.

Finally, **9** was identified as 4-methyl-5,6-dihydro-2H-pyran-2-one, a known degradation product of fusarinines (Tables S30–31 and Figures S136,-143).^25,27^

### Absolute Configuration of Compounds **1**–**8** by NMR and Marfey’s Analysis

The configuration of the Δ^9(19)^, Δ^9′(19′)^ and Δ^9″(19″)^ double bonds in the monomeric, dimeric, and trimeric forms was determined to be *Z*, based on NOESY and ROESY-NMR experiments conducted on the monomers **1**–**3** (Figures S9–S10, S19–S20 and S29–S30). This conclusion was further supported by characteristic carbon chemical shifts observed for C-11 and C-16 in all compounds. Specifically, C-11 resonated within the range of δ_C_ 32/38, while the δ_H_ and δ_C_ signals for C-16, were observed at δ_H_ 1.86/1.97 and δ_C_ 23/25, respectively, in agreement with a *Z* configuration.

The absolute configuration of the stereogenic centers of compounds **1**-**8** was established using Marfey′s analysis (Figure S144). The HPLC retention times of the Marfey derivatives of hydrolyzed samples of each compound were consistent with that of the l-ornithine derivative, establishing an l-configuration for all compounds. The identity of the main compound eluting at RT 16.3 min as the monosubstituted l-FDVA-l-ornithine derivative was further supported by LRESIMS analyses (*m*/*z* 413). These findings confirmed that compounds **1**-**8** produced by *P. verrucosus* FAE27, belong to the fusarinine family, characterized by a uniform *Z* double bond, and the *S* configuration on the α-protons derived from l-ornithine.

### Siderophore Activity Assays of **1–8**

Fusarinine monomers **1**–**3**, along with their corresponding macrolactone open dimers **6** and **8** and trimers **5** and **7** have been described as degradation products of **4**. To assess whether monomers could function as iron transport agents, Jalal et al. reported in the 1987 the synthesis of ^54^Fe^3+^ complexes using fusarinine monomer units and evaluated their ^54^Fe^3+^ uptake from the extracellular medium into the cell.^40^ However, it remains unclear whether fusarinine monomers present in culture media can naturally form iron-chelating complexes and thereby promote fungal growth. Furthermore, no experimental evidence has been reported on the siderophore activity of dimers and trimers with the open macrolactone. To address these questions, siderophore activity assays were performed. First, a growth-inhibitory condition for *P. verrucosus* FAE27 was established by supplementing the solid culture medium with 400 μM of bathophenanthroline disulfonic acid (BPS). Increasing concentrations of **4**-**8**, in both their *apo-* and *holo* (Ga^3+^ chelated) forms, were added to the medium resulting in the restoration of fungal growth, thereby confirming their siderophore activity (Figure 3). Regarding monomers **1**–**3**, only **3** exhibited siderophore activity at the highest initial concentration tested (50 μM). Consequently, an additional test was conducted using higher monomer concentrations, revealing that as the concentration increased, all three monomers (**1–3**) effectively promoted fungal growth.

**Figure 3:**
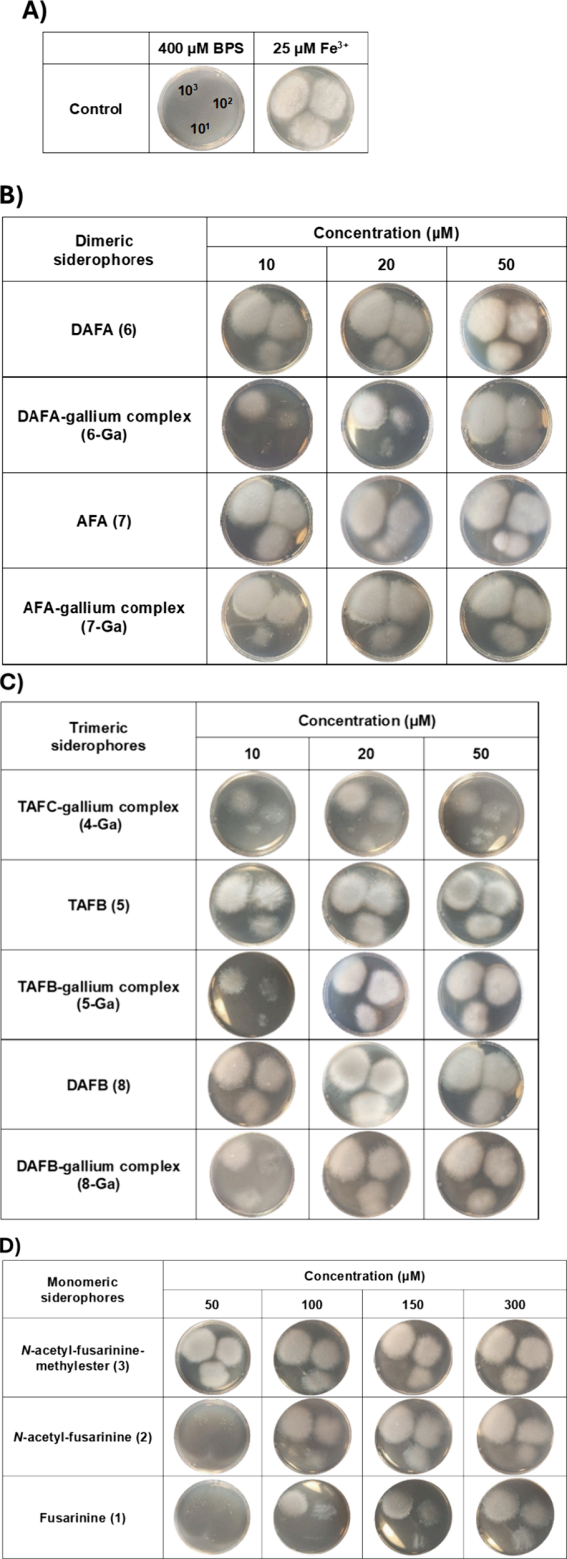
Bioassays for siderophore activity in *Pseudogymnoascus verrucosus* FAE27. All experiments were conducted on solid M9 medium supplemented with 400 μM BPS. *P. verrucosus* spores were inoculated in three quantities (10, 10^2^, 10^3^). Photographs were taken after 10 days of incubation at 15 °C. (**A**) Negative (M9 medium supplemented with BPS) and positive controls (M9 medium supplemented with Fe^3+^). (**B**) Plates were supplemented with 10, 20, and 50 μM dimers in their *apo*- and *holo*- (Ga^3+^ chelated) forms. (**C**) Plates were supplemented with 10, 20, and 50 μM trimers in their *apo*- (unchelated) and *holo*- (Ga^3+^ chelated) forms. (**D**) Plates were supplemented with 50, 100, 150, and 300 μM of monomers.

Notably, gallium complexes (**4-Ga** to **8-Ga**) also supported fungal growth. Under iron-limiting conditions, such as those used in this study, gallium­(III) salts have been shown to exert a fungistatic effect, likely due to their interference with iron homeostasis within the cell.^41^ Interestingly, the gallium complexes (**4-Ga** to **8-Ga**), at concentrations close to the minimum inhibitory concentration (MIC) reported for gallium salts, not only failed to exhibit antimicrobial activity but also promoted fungal growth. These results align with the established role of siderophores in protecting pathogens from metal ion toxicity.

### Biological Implications of Partially Deacetylated Compounds **7** and **8**

The mechanisms governing iron release from iron-siderophore complexes are highly specific. In *Aspergillus* spp., for example, three esterases have been identified that exhibit strict substrate specificity, hydrolyzing fusarinines intracellularly to generate monomers, dimers, and open macrolactone trimers.^42–45^ On the other hand, the possible presence of the deacetylated dimer **8** suggested the involvement of deacetylases in iron release process.^46^

The isolation and structural characterization of partially acetylated dimers and trimers in this study provide experimental support for a finely regulated hydrolysis mechanism involving both esterases and acetylases. Specifically, the detection of the diacetylated trimer DAFB (**8**), in which the amino group at position C-2 is deacetylated, suggests a stepwise process: first, deacetylation of the NH-1 takes placed and then, hydrolysis of the adjacent ester bond at C-3 occurs. Subsequent hydrolysis of a second ester bond in compound **8** would generate dimers **6** and **7**. The presence of the DAFB trimer (**8**) and the absence of trimers with a deacetylated amino group at positions C-2′ or C-2″ further support this hypothesis. These findings not only reinforce the proposed role of acetylases in siderophore metabolism^46^ but also support a dual mechanism: while acetylation stabilizes siderophores during biosynthesis,^26^ subsequent deacetylation primes them for esterase-mediated hydrolysis.

## Conclusions

This study successfully identified all trivalent metal-complexes (with Fe^3+^ or Ga^3+^) present in the culture broth of *P. verrucosus*. These compounds belong to the hydroxamate family, specifically fusarinines and ferrichromes. The isolation and characterization of fusarinines from the extracellular medium, in both their *apo-* and *holo-* gallium forms, led to the identification of six known compounds (**1**–**6**) and two novel, partially acetylated derivatives, AFA (**7**) and DAFB (**8**), which likely are originated from TAFC degradation (**4**). Notably, the comprehensive spectroscopic characterization performed in this study fills a critical gap in the structural analysis of fusarinine-type siderophores.

The analysis of acetylated and deacetylated sites in these compounds suggests a dual regulatory mechanism: acetylation stabilizes siderophores during biosynthesis, whereas deacetylation facilitates ester bond cleavage, promoting iron release.

Finally, *in vivo* uptake experiments demonstrated that all isolated compounds—monomers, dimers, and trimers, in both open and closed forms—effectively chelate Fe^3+^ and support fungal growth, underscoring their potential role in extracellular iron acquisition.

## Experimental Section

### General Experimental Procedures

Optical rotations were measured on a JASCO DIP-1000 polarimeter equipped with a Na (589 nm) lamp and filter. ^1^H, ^13^C, and 2D NMR spectra were recorded on a NEO Bruker Avance 500 spectrometer operating at 500.13 and 125.0 MHz, respectively, with CD_3_OD or (CD_3_)_2_SO as solvents. LRESIMS analyses were performed on a LTQ-Orbitrap Discovery system coupled to a U-HPLC Accela Thermo Fisher Scientific instrument. HRESIMS experiments were performed on a LC-MS Thermo Scientific - MSQ PLUS. HPLC separations were carried out on a Waters 1525 HPLC system equipped with a solvent degasser, a Waters 1525 Binary HPLC pump and a Waters 2996 Photodiode Array (PDA) detector.

### Fungal Strain and Growth Conditions

The fungal strain *Pseudogymnoascus verrucosus* FAE27, previously isolated from the Antarctic marine sponge *Hymeniacidon* sp. collected in King George Island, South Shetland Islands, Antarctica, in 2009,^17^ was used in this study. This strain was grown on potato dextrose agar (PDA, BD Difco) at 15 °C in darkness, for 15 days to promote conidia production. After this time, the conidia were harvested by scraping the surface of the plate with a cut tip in a defined volume of sterile water. The spore suspension was then filtered and the conidia counted in a Newbauer counting chamber.

To induce siderophore production, 5 × 10^6^ conidia were inoculated into 100 mL of M9 medium^47,48^ supplemented with 0.4% sucrose. Cultures were incubated at 15 °C for 10 days with constant shaking at 180 rpm.

To prevent iron contamination, all glassware was soaked in 1 M HCl overnight, rinsed three times with 6 M HCl, and thoroughly washed with ultrapure water prior to use.

### Analytical Detection of Fe^3+^ and Ga^3+^ Complexes

After 10 days, mycelia from 1 L of culture were removed by filtration using a Myracloth filter, followed by centrifugation at 9000 rpm for 10 min. The resulting culture broth was divided into two portions, and FeCl_3_·6H_2_O (0.1 mg/mL) or Ga­(acac)_3_ (0.07 mg/mL) was added to each, followed by gentle stirring. After incubation at 4 °C for 48 h, each solution containing Fe^3+^ or Ga^3+^ complexes was passed through an XAD-16 resin column. The resin was washed with water to remove the salt and then eluted with methanol. Methanol was evaporated, and 1 mg of each extract was reconstituted in 1 mL of (1:1) acetonitrile/water mixture. Samples were filtered through 0.45 μm polytetrafluoroethylene (PTFE) filters into 2 mL glass HPLC vials and submitted to HRESIMS analysis. This analysis was carried out using a C-18 reversed-phase column (Kinetex-C18, Phenomenex, 4.6 mm x 15 cm, 5 μm) with a mobile phase consisting of a 12 min gradient from 5 to 100% B (H_2_O with 0.1% formic acid as solvent A and acetonitrile with 0.1% formic acid as solvent B). The flow rate was 0.3 mL/min and the column temperature 40 °C. The injection volume was 3 μL. Analytes were ionized by electrospray ionization operating in the positive ionization mode with the following settings: nitrogen as desolvation gas with a flow of 600 L/h, capillary temperature 350 °C and capillary and cone voltages of 3.90 kV and 35 V, respectively.

### Isolation of *apo-* and *holo*- (Ga^3+^ Chelated) Forms

For gallium complexes isolation, 3 L of culture broth were treated with Ga­(acac)_3_ (0.07 mg/mL) and incubated at 4 °C for 48 h. The solution was frozen and lyophilized, yielding a dried residue which was extracted twice with methanol (HPLC grade). After evaporation of the methanol, 520 mg of residue was obtained. The methanol extract was separated by semipreparative HPLC using a C18 reversed-phase column (Sunfire C18, Waters, 10 × 25 cm, 5 μm) and a binary gradient of 5%–100% solvent B over 15 min (solvent A: H_2_O with 0.1% TFA; solvent B: acetonitrile with 0.02% TFA). The flow rate was 4.5 mL/min, and detection was carried out at 254 nm. Seven fractions were collected, neutralized to pH 7 with NaOH, concentrated, and lyophilized.

For the isolation of the *apo* forms, 1.5 L of culture broth was frozen and lyophilized. The dried broth was extracted with methanol, yielding 420 mg of a residue which was submitted to semipreparative HPLC as previously described for gallium complexes, to give fractions which were collected, concentrated and lyophilized. The purity and composition of 14 fractions were analyzed by analytical HPLC and direct injection into HRESIMS prior to spectroscopic characterization.

### NMR Analysis

The structure of the purified compounds was determined by ^1^H and ^13^C NMR spectroscopy in CD_3_OD or (CD_3_)_2_SO.

### Amino Acid Analysis Using Marfey’s Reagent Derivatization

Compounds **1**–**3** (1 mg each) were hydrolyzed in 1 mL of HI 57% at 110 °C for 18 h in a Schlenk tube. The hydrolysates were dried under a flow of N_2_ and resuspended in 100 μL of H_2_O. Then, 100 μL of 1% (w/v) *N*α-(2,4-dinitro-5-fluorophenyl)-l-valinamide (l-FDVA) in acetone and 50 μL of NaHCO_3_ (1M) were added. The mixtures were heated at 40 °C for 1 h and then, dried under a flow of N_2_. The derivatized amino acids were resuspended in a 1:1 H_2_O/CH_3_CN solution (500 μL), filtered and analyzed by HPLC (Sunfire C18, Waters, 4.6 × 25 cm, 5 μm). The separation was performed by using a linear gradient of 5%–55% B over 30 min (H_2_O with 0.1% formic acid as solvent A and acetonitrile with 0.1% formic acid as solvent B), a flow rate of 1 mL/min, an injection volume of 10 μL and a detection wavelength of 360 nm. In addition, the derivatives were analyzed by LC/LRESIMS using a C18 column (Discovery C18, Supelco, 4,6 × 15 cm, 5 μm) under the following conditions: solvent A (H_2_O in 0.1% formic acid) and solvent B (acetonitrile in 0.1% formic acid). Solvent B was increased from 5% to 55% in 30 min at a flow rate of 0.3 mL/min. Analytes were ionized by electrospray ionization operating in the positive mode with the following settings: N_2_ as desolvation gas with a flow of 600 L/h, capillary temperature 350 °C and capillary and cone voltages of 3.90 kV and 35 V, respectively.

The configuration of ornithine was determined by comparison with the retention times of Marfey′s derivatives prepared from authentic amino acid standards of d/l-ornithine (Sigma-Aldrich) and MS spectra. The retention times for l-FDVA derivatives were for d-ornithine 15.35 min and for l-ornithine 16.3 min.

### Siderophore Activity Assays

To evaluate the ability of *Pseudogymnoascus* to uptake and utilize siderophores as iron source, growth assays were performed on M9-agar plates (M9 broth iron-limited; 1.5 g/L agar) supplemented with 400 μM of the ferrous iron chelator bathophenanthroline disulfonic acid (BPS) and varying concentrations (10, 30, 50, 100, 150, or 300 μM) of the *apo* and *holo* (Ga^3+^ chelated) forms. Then, 10^1^, 10^2^ and 10^3^ conidia were point-inoculated at three equidistant points on each plate. Plates were incubated at 15 °C for 12 days. Controls included plates with 400 μM BPS (negative) and 25 μM FeCl_3_ 6H_2_O (positive).

#### Fusarinine (**1**)

RT 5.579 min; 35.3 mg/L in culture broths; yellow powder; [α]_D_^23^ −3 (*c* 0.1, MeOH); UV (MeOH) λ_max_ (log ε): 220.3 (1.35) nm; ^1^H, ^13^C, and HMBC NMR data in Table S3 (^13^C NMR was assigned with the aid of HMQC and HMBC); (+)-HRESIMS *m*/*z* 283.1265 [M + Na]^+^ (calcd for C_11_H_20_N_2_O_5_Na^+^ 283.1270, Δ 1.8); (+)-HRESIMS *m*/*z* 305.1084 [MCOONa + Na]^+^ (calcd for C_11_H_19_N_2_O_5_Na_2_^+^ 305.1090, Δ 2.0).

#### *N*-Acetyl-fusarinine (**2**)

RT 7.219 min; 12.8 mg/L in culture broths; yellow powder; [α]_D_^23^ +2 (*c* 0.1, MeOH); UV (MeOH) λ_max_ (log ε): 221.5 (0.9) nm; ^1^H, ^13^C, and HMBC NMR data in Table S5 (^13^C NMR was assigned with the aid of HMQC and HMBC); (+)-HRESIMS *m*/*z* 325.1371 [M + Na]^+^ (calcd for C_13_H_22_N_2_O_6_Na 325.1376, Δ 1.5); (+)-HRESIMS *m*/*z* 347.1190 [MCOONa + Na]^+^ (calcd for C_13_H_21_N_2_O_6_Na_2_^+^ 347.1196, Δ 1.7)

#### *N*-Acetyl-fusarinine-methylester (**3**)

RT 8.444 min; 13.3 mg/L in culture broths; yellow powder; [α]_D_^23^ −1 (*c* 0.1, MeOH); UV (MeOH) λ_max_ (log ε): 220.5 (1.35) nm; ^1^H, ^13^C, and HMBC NMR data in Table S7 (^13^C NMR was assigned with the aid of HMQC and HMBC); (+)-HRESIMS *m*/*z* 339.1524 [M + Na]^+^ (calcd for C_14_H_24_N_2_O_6_Na^+^ 339.1532, Δ 2.4)

#### *N*,*N*′,*N*″-Triacetyl-fusarinine C Ga^3+^ Complex (**4-Ga**)

RT 10.137 min; 1.5 mg/L in culture broths supplemented with Ga^3+^; white powder; [α]_D_^23^ −82 (*c* 0.1, MeOH); UV (MeOH) λ_max_ (log ε): 220.3 (0.205), 262.9 (0.140) nm; ^1^H, ^13^C NMR and HMBC data in Table 2 and Figure 2 (^13^C NMR was assigned with the aid of HMQC and HMBC); (+)-HRESIMS *m*/*z* 941.3059/943.3063 [M–3H+^69/71^Ga+Na]^+^ (calcd for C_39_H_57_GaN_6_O_15_Na^+^ 941.3037/943.3028, Δ 2.3/3.7); (+)-HRESIMS *m*/*z* 957.2797/959.2799 [M–3H+^69/71^Ga+K]^+^ (calcd for C_39_H_57_GaN_6_O_15_K^+^ 957.2776/959.2767, Δ 2.2/3.3)

#### *N*,*N*′,*N*″-Triacetyl-fusarinine B Ga^3+^ Complex (**5-Ga**)

RT 9.172 min; 1.6 mg/L in culture broths supplemented with Ga^3+^; white powder; [α]_D_^23^ −4 (*c* 0.1, MeOH); UV (MeOH) λ_max_ (log ε): 219.2 (0.370), 265.3 (0.290) nm; ^1^H, ^13^C NMR and HMBC data in Table S11 (^13^C NMR was assigned with the aid of HMQC and HMBC); (+)-HRESIMS *m*/*z* 959.3139/961.3139 [M-3H+^69/71^Ga+Na]^+^ (calcd for C_39_H_59_GaN_6_O_16_Na^+^ 959.3142/961.3133, Δ 0.3/0.6); (+)-HRESIMS *m*/*z* 981.2955/983.2953 [MCOONa + Ga + Na]^+^ (calcd for C_39_H_58_GaN_6_O_16_Na_2_^+^ 981.2962, Δ 0.7/1.8).

#### *N*,*N*′,*N*-Triacetyl-fusarinine B (**5**)

RT 10.270 min; 7.5 mg/L in culture broths; white powder; [α]_D_^23^ −3 (*c* 0.1, MeOH); UV (MeOH) λ_max_ (log ε): 223.9 (0.325) nm; ^1^H, ^13^C NMR and HMBC data in Tables S13–S14 (^13^C NMR was assigned with the aid of HMQC and HMBC); (+)-HRESIMS *m*/*z* 893.4132 [M + Na]^+^ (calcd for C_39_H_62_N_6_O_16_Na^+^ 893.4120, Δ 1.3); (+)-HRESIMS *m*/*z* 915.3950 [MCOONa + Na]^+^ (calcd for C_39_H_61_N_6_O_16_Na_2_^+^ 915.3940, Δ 1.1).

#### *N*,*N*′-Diacetyl-fusarinine A Ga^3+^ Complex (**6-Ga**)

RT 7.786 min; 1.9 mg/L in culture broths supplemented with Ga^3+^; white powder; [α]_D_^23^ +1 (*c* 0.1, MeOH); UV (MeOH) λ_max_ (log ε): 218.0 (0.330) nm; ^1^H, ^13^C NMR and HMBC data in Table S16 (^13^C NMR was assigned with the aid of HMQC and HMBC); (+)-HRESIMS *m*/*z* 653.1948/655.1942 [M – 2H + ^69/71^Ga]^+^ (calcd for C_26_H_40_GaN_4_O_11_^+^ 653.1950/655.1941, Δ 0.3/0.2); (+)-HRESIMS *m*/*z* 675.1767/677.1760 [MCOONa-2H+Ga]^+^ (calcd for C_26_H_39_GaN_4_O_11_Na^+^ 675.1770/677.1761, Δ 0.4/0.1)

#### *N*,*N*′-Diacetyl-fusarinine A (**6**)

RT 9.198 min; 9.3 mg/L in culture broths; white powder; [α]_D_^23^ +2 (*c* 0.1, MeOH); UV (MeOH) λ_max_ (log ε): 212.1 (1.27) nm; ^1^H, ^13^C NMR and HMBC data in Tables S18–S19 (^13^C NMR was assigned with the aid of HMQC and HMBC); (+)-HRESIMS *m*/*z* 609.2746 [M + Na]^+^ (calcd for C_26_H_42_N_4_O_11_Na^+^ 609.2748, Δ 0.3); (+)-HRESIMS *m*/*z* 625.2475 [M + K]^+^ (calcd for C_26_H_42_N_4_O_11_K^+^ 625.2487, Δ 1.9)

#### *N*,*N*′-Diacetyl-fusarinine B Ga^3+^ Complex (**7-Ga**)

RT 7.187 min; 1.3 mg/L in culture broths supplemented with Ga^3+^; white powder; [α]_D_^23^ −31 (*c* 0.1, MeOH); UV (MeOH) λ_max_ (log ε): 218.0 (0.800), 265.3 (0.600) nm; ^1^H, ^13^C NMR and HMBC data in Table 2 and Figure 2 (^13^C NMR was assigned with the aid of HMQC and HMBC); (+)-HRESIMS *m*/*z* 895.3231/897.3234 [M-3H+^69/71^Ga+H]^+^ (calcd for C_37_H_58_GaN_6_O_15_^+^ 895.3217/897.3208, Δ 1.6/2.3); (+)-HRESIMS *m*/*z* 917.3050/919.3054 [MCOONa + Ga + Na]^+^ (calcd for C_37_H_57_GaN_6_O_15_Na^+^ 917.3037/919.3028, Δ 1.4/2.8).

#### *N*,*N*′-Diacetyl-fusarinine B (**7**)

RT 8.035 min; 0.7 mg/L in culture broths; white powder; [α]_D_^23^ −13 (*c* 0.1, MeOH); UV (MeOH) λ_max_ (log ε): 219.2 (0.370), 265.3 (0.290) nm; (+)-HRESIMS *m*/*z* 829.4190 [M + H]^+^ (calcd for C_37_H_61_N_6_O_15_^+^ 829.4195, Δ 0.6); (+)-HRESIMS *m*/*z* 851.4011 [M + Na]^+^ (calcd for C_37_H_60_N_6_O_15_Na^+^ 851.4015, Δ 0.5).

#### *N*-Acetyl-fusarinine A Ga^3+^ Complex (**8-Ga**)

RT 8.868 min; 1.5 mg/L in culture broths supplemented with Ga^3+^; white powder; [α]_D_^23^ +4 (*c* 0.1, MeOH); UV (MeOH) λ_max_ (log ε): 210.0 (0.650), 260.5 (0.225) nm; ^1^H, ^13^C NMR and HMBC data in Table 3 and Figure 2 (^13^C NMR was assigned with the aid of HMQC and HMBC); (+)-HRESIMS *m*/*z* 611.1838/613.1830 [M – 2H + ^69/71^Ga]^+^ (calcd for C_24_H_38_GaN_4_O_10_^+^ 611.1844/613.1835, Δ 1.0/0.8); (+)-HRESIMS *m*/*z* 633.1657/635.1665 [MCOONa-2H+Ga]^+^ (calcd for C_24_H_37_GaN_4_O_10_Na^+^ 633.1664/635.1655, Δ 1.1/1.6)

#### *N*-Acetyl-fusarinine A (**8**)

RT 9.279 min; 13.3 mg/L in culture broths; white powder; [α]_D_^23^ −1 (*c* 0.1, MeOH); UV (MeOH) λ_max_ (log ε): 219.2 (1.15) nm; ^1^H, ^13^C NMR and HMBC data in Tables S28–S29 (^13^C NMR was assigned with the aid of HMQC and HMBC); (+)-HRESIMS *m*/*z* 545.2822 [M + H]^+^ (calcd for C_24_H_41_N_4_O_10_^+^ 545.2822, Δ 0); (+)-HRESIMS *m*/*z* 567.2641 [M + Na]^+^ (calcd for C_24_H_40_N_4_O_10_Na^+^ 567.2642, Δ 0.2)

## Supplementary Material



## Data Availability

The raw NMR data for compounds **1–9** have been deposited in the Natural Products Magnetic Resonance Database (NP-MRD; www.np-mrd.org) and can be found at NP0351070 (*Z*-l-fusarinine, **1**), NP0351071 (*N*-acetyl-*Z*-l-fusarinine, **2**), NP0351072 (*N*-acetyl-*Z*-l-fusarinine-methylester, **3**), NP0351084 (*N,N′,N″*-triacetyl-*Z*-l-fusarinine C Ga^3+^ complex, **4-Ga**), NP0351074 (*N,N′,N″*-triacetyl-*Z*-l-fusarinine B, **5**), NP0351075 (*N,N′,N″*-triacetyl-*Z*-l-fusarinine B Ga^3+^ complex, **5-Ga**), NP0351085 (*N,N′*-diacetyl-*Z*-l-fusarinine A, **6**), NP0351076 (*N,N′*-diacetyl-*Z*-l-fusarinine A Ga^3+^ complex, **6-Ga**), NP0351077 (*N,N′*-diacetyl-*Z*-l-fusarinine B Ga^3+^ complex, **7-Ga**), NP0351078 (*N*-acetyl-*Z*-l-fusarinine A, **8**), NP0351079 (*N*-acetyl-*Z*-l-fusarinine A Ga^3+^ complex, **8-Ga**) and NP0351073 (4-methyl-5,6-dihydro-2H-pyran-2-one, **9**).

## Notes

^1^H, ^13^C, DEPT-135, COSY, HSQC, HMBC, NOESY, and ROESY NMR spectra and (+) and (−)-HRESIMS spectra for fusarinines **1–8**. Analysis by advance Marfey’s method of fusarinines **1–8** (PDF)
