# Supplementary material for: Siderophore Activity of Partially Acetylated Fusarinines from the Sponge-Derived Fungus Pseudogymnoascus verrucosus
Source: J Nat Prod. 2025 Jul 10;88(7):1643–52. doi: 10.1021/acs.jnatprod.5c00399 (PMC12305646; doi:10.1021/acs.jnatprod.5c00399)
Supplement: Supplementary file 1 [file np5c00399_si_001.pdf]

Supporting information

Siderophore Activity of Partially Acetylated Fusarinines from the Sponge-Derived Fungus *Pseudogymnoascus verrucosus*

Mariana Montanares,† Carlos Jiménez,‡ Jaime Rodríguez,‡ * Anaí Diaz,† Lucía Ageitos,‡ Renato Chávez*,§ Inmaculada Vaca†**

† Departamento de Química, Facultad de Ciencias, Universidad de Chile, Las Palmeras 3425, Ñuñoa, Santiago, Chile

‡ CICA - Centro Interdisciplinar de Química e Bioloxía, Departamento de Química, Facultade de Ciencias, Universidade da Coruña, 15071 A Coruña, Spain

§ Departamento de Biología, Facultad de Química y Biología, Universidad de Santiago de Chile (USACH), Alameda 3363, Estación Central, Santiago, Chile

e-mail corresponding author: inmavaca@uchile.cl

Table of Contents

Table S1. LC/(+)-HRESIMS analysis of fractions from broth extracts of *P. verrucosus* FAE27 grown under iron-limiting conditions. 9

Z-L-fusarinine (1) 10

Table S2. Major *m/z* ions in the (+) HRMS-ESI of *Z*-L-fusarinine (**1**) 10

Figure S1. (+) HRMS-ESI of *Z*-L-fusarinine (**1**) 10

Table S3. NMR chemical shifts of *Z*-L-fusarinine (**1**) in CD_3OD (400 MHz) 11

Figure S2. ^1H-^1H COSY (▬) and ^1H-^{13}C HMBC correlations (⟶) of Z-L-fusarinine (**1**) in CD_3OD 11

Figure S3. ^1H-NMR spectrum of *Z*-L-fusarinine (**1**) in CD_3OD (400 MHz) 12

Figure S4. ^{13}C-NMR spectrum of *Z*-L-fusarinine (**1**) in CD_3OD (100 MHz) 13

Figure S5. DEPT135-NMR spectrum of *Z*-L-fusarinine (**1**) in CD_3OD (100 MHz) 13

Figure S6. 2D-COSY-NMR spectrum of *Z*-L-fusarinine (**1**) in CD_3OD 14

Figure S7. 2D-HSQC-NMR spectrum of *Z*-L-fusarinine (**1**) in CD_3OD 14

Figure S8. 2D-HMBC-NMR spectrum of *Z*-L-fusarinine (**1**) in CD_3OD 15

Figure S9. 1D-NOESY-NMR spectrum of *Z*-L-fusarinine (**1**) in CD_3OD (300 MHz), obtained band selective excitation of the signal at 6.37 ppm 15

Figure S10. 1D-ROESY-NMR spectrum of *Z*-L-fusarinine (**1**) in CD_3OD (300 MHz), obtained band selective excitation of the signal at 6.37 ppm 16

N-acetyl-*Z*-L-fusarinine (2) 17

Table S4. Major *m/z* ions in the (+) HRMS-ESI of *N*-acetyl-*Z*-L-fusarinine (**2**) 17

Figure S11. (+) HRMS-ESI of *N*-acetyl-*Z*-L-fusarinine (**2**) 17

Table S5. NMR chemical shifts of *N*-acetyl-*Z*-L-fusarinine (**2**) in CD_3OD (400 MHz) 18

Figure S12. ^1H-^1H COSY and ^1H-^{13}C HMBC correlations (⟶) of *N*-acetyl-*Z*-L-fusarinine (**2**) in CD_3OD 18

Figure S13. ^1H-NMR spectrum of *N*-acetyl-*Z*-L-fusarinine (**2**) in CD_3OD (400 MHz) 19

Figure S14. ^{13}C-NMR spectrum of *N*-acetyl-*Z*-L-fusarinine (**2**) in CD_3OD (100 MHz) 19

Figure S15. DEPT135-NMR spectrum of *N*-acetyl-*Z*-L-fusarinine (**2**) in CD_3OD (100 MHz) 20

Figure S16. 2D-COSY-NMR spectrum of *N*-acetyl-*Z*-L-fusarinine (**2**) in CD_3OD 20

Figure S17. 2D-HSQC-NMR spectrum of *N*-acetyl-*Z*-L-fusarinine (2) in CD_3OD 21

Figure S18. 2D-HMBC-NMR spectrum of *N*-acetyl-*Z*-L-fusarinine (**2**) in CD_3OD 21

Figure S19. 1D-NOESY-NMR spectrum of *N*-acetyl-*Z*-L-fusarinine (**2**) in CD_3OD (300 MHz), obtained band selective excitation of the signal at 6.36 ppm 22

Figure S20. 1D-ROESY-NMR spectrum of *N*-acetyl-*Z*-L-fusarinine (**2**) in CD_3OD (300 MHz), obtained band selective excitation of the signal at 6.36 ppm 22

N-acetyl-*Z*-L-fusarinine-methylester (3) 23

Table S6. Major *m/z* ions in the (+) HRMS-ESI of *N*-acetyl-*Z*-L-fusarinine-methylester (**3**) 23

Figure S21. (+) HRMS-ESI of *N*-acetyl-*Z*-L-fusarinine-methylester (**3**) 23

Table S7. NMR chemical shifts of *N*-acetyl-*Z*-L-fusarinine-methylester (**3**) in CD_3OD (500 MHz)....................24

Figure S22. ^{1}H-^{1}H COSY (-) and ^{1}H-^{13}C HMBC (⟶) correlations of *N*-acetyl-*Z*-L-fusarinine-methyl ester (**3**) in CD_3OD....................24

Figure S23. ^{1}H-NMR spectrum of *N*-acetyl-*Z*-L-fusarinine-methylester (**3**) in CD_3OD (500 MHz) 25

Figure S24. ^{13}C-NMR spectrum of *N*-acetyl-*Z*-L-fusarinine-methylester (**3**) in CD_3OD (125 MHz) 25

Figure S25. DEPT135-NMR spectrum of *N*-acetyl-*Z*-L-fusarinine-methylester (**3**) in CD_3OD (125 MHz) 26

Figure S26. 2D-COSY-NMR spectrum of *N*-acetyl-*Z*-L-fusarinine-methylester (**3**) in CD_3OD 26

Figure S27. 2D-HSQC-NMR spectrum of *N*-acetyl-*Z*-L-fusarinine-methylester (**3**) in CD_3OD 27

Figure S28. 2D-HMBC-NMR spectrum of *N*-acetyl-*Z*-L-fusarinine-methylester (**3**) in CD_3OD 27

Figure S29. 1D-NOESY-NMR spectrum of *N*-acetyl-*Z*-L-fusarinine-methylester (**3**) in CD_3OD (300 MHz), obtained band selective excitation of the signal at 6.37 ppm 28

Figure S30. 1D-ROESY-NMR spectrum of *N*-acetyl-*Z*-L-fusarinine-methylester (**3**) in CD_3OD (300 MHz), obtained band selective excitation of the signal at 6.37 ppm 28

N,N',N"-Z-L-triacetyl-fusarinine C Ga^{3+} complex (4-Ga) ________________ 29

Table S8. Major *m/z* ions in the spectra (+) and (-) HRMS-ESI of *N,N',N''*-triacetyl-*Z*-L-fusarinine C Ga^{3+} complex (**4-Ga**) 29

Figure S31. (+) HRMS-ESI of *N,N',N''*-triacetyl-*Z*-L-fusarinine C Ga^{3+} complex (**4-Ga**) 29

Figure S32. (-) HRMS-ESI of *N,N',N''*-triacetyl-*Z*-L-fusarinine C Ga^{3+} complex (**4-Ga**) 30

Table S9. NMR chemical shifts of *N,N',N''*-triacetyl-*Z*-L-fusarinine C Ga^{3+} complex (**4-Ga**) in CD_3OD (500 MHz) 31

Figure S33. ^{1}H-NMR spectrum of *N,N',N''*-triacetyl-*Z*-L-fusarinine C Ga^{3+} complex (**4-Ga**) in CD_3OD (500 MHz) 32

Figure S34. ^{13}C-NMR spectrum *N,N',N''*-triacetyl-*Z*-L-fusarinine C Ga^{3+} complex (**4-Ga**) in CD_3OD (125 MHz)32

Figure S35. DEPT135-NMR spectrum of *N,N',N''*-triacetyl-*Z*-L-fusarinine C Ga^{3+} complex (**4-Ga**) in CD_3OD (125 MHz) 33

Figure S36. 2D-COSY-NMR spectrum of *N,N',N''*-triacetyl-*Z*-L-fusarinine C Ga^{3+} complex (**4-Ga**) in CD_3OD ..33

Figure S37. 2D-HSQC-NMR spectrum of *N,N',N''*-triacetyl-*Z*-L-fusarinine C Ga^{3+} complex (**4-Ga**) in CD_3OD ..34

Figure S38. 2D-HMBC-NMR spectrum of *N,N',N''*-triacetyl-*Z*-L-fusarinine C Ga^{3+} complex (**4-Ga**) in CD_3OD .34

N,N',N''-triacetyl-*Z*-L-fusarinine B Ga^{3+} complex (5-Ga) ________________ 35

Table S10. Major *m/z* ions in the (+) and (-) HRMS-ESI of *N,N',N''*-triacetyl-*Z*-L-fusarinine B Ga^{3+} complex (**5-Ga**)....................35

Figure S39. (+) HRMS-ESI of *N,N',N''*-triacetyl-*Z*-L-fusarinine B Ga^{3+} complex (**5-Ga**) 35

Figure S40. (-) HRMS-ESI of *N,N',N''*-triacetyl-*Z*-L-fusarinine B Ga^{3+} complex (**5-Ga**) 36

Table S11. NMR chemical shifts of *N,N',N''*-triacetyl-*Z*-L-fusarinine B Ga^{3+} complex (**5-Ga**) in CD_3OD (500 MHz)....................37

Figure S41. ^{1}H-^{1}H COSY (▬) and ^{1}H-^{13}C HMBC(→) correlations of *N,N’,N’’*-triacetyl-*Z*-L-fusarinine B Ga^{3+} complex (**5-Ga**) in CD$_3$OD....38

Figure S42. ^{1}H-NMR spectrum of *N,N’,N”*-triacetyl-*Z*-L-fusarinine B Ga^{3+} complex (**5-Ga**) in CD$_3$OD (500 MHz) 39

Figure S43. ^{13}C-NMR spectrum of *N,N’,N’’*-triacetyl-*Z*-L-fusarinine B Ga^{3+} complex (**5-Ga**) in CD$_3$OD (125 MHz)....40

Figure S44. DEPT135-NMR spectrum of *N,N’,N’’*-triacetyl-*Z*-L-fusarinine B Ga^{3+} complex (**5-Ga**) in CD$_3$OD (125 MHz) 40

Figure S45. 2D-COSY-NMR spectrum of *N,N’,N’’*-triacetyl-*Z*-L-fusarinine B Ga^{3+} complex (**5-Ga**) in CD$_3$OD ..41

Figure S46. 2D-HSQC-NMR spectrum of *N,N’,N’’*-triacetyl-*Z*-L-fusarinine B Ga^{3+} complex (**5-Ga**) in CD$_3$OD ..41

Figure S47. 2D-HMBC-NMR spectrum of *N,N’,N’’*-triacetyl-*Z*-L-fusarinine B Ga^{3+} complex (**5-Ga**) in CD$_3$OD .42

N,N’,N’’-triacetyl-*Z*-L-fusarinine B (5) ____ 43

Table S12. Major *m/z* ions in (+) and (-) HRMS-ESI of *N,N’,N’’*-triacetyl-*Z*-L-fusarinine B (**5**) 43

Figure S48. (+) HRMS-ESI of *N,N’,N’’*-triacetyl-*Z*-L-fusarinine B (**5**)....43

Figure S49. (-) HRMS-ESI of *N,N’,N’’*-triacetyl-*Z*-L-fusarinine B (**5**)....44

Table S13. NMR chemical shifts of *N,N’,N’’*-triacetyl-*Z*-L-fusarinine B (**5**) in CD$_3$OD (400 MHz) 45

Figure S50. ^{1}H-^{1}H COSY (▬) and ^{1}H-^{13}C HMBC (→) correlations of *N,N’,N’’*-triacetyl-*Z*-L-fusarinine B (**5**) in CD$_3$OD....46

Figure S51. ^{1}H-NMR spectrum of *N,N’,N’’*-triacetyl-*Z*-L-fusarinine B (**5**) in CD$_3$OD (400 MHz) 47

Figure S52. ^{13}C-NMR spectrum of *N,N’,N’’*-triacetyl-*Z*-L-fusarinine B (**5**) in CD$_3$OD (100 MHz) 47

Figure S53. DEPT135-NMR spectrum of *N,N’,N’’*-triacetyl-*Z*-L-fusarinine B (**5**) in CD$_3$OD (100 MHz)....48

Figure S54. 2D-COSY-NMR spectrum of *N,N’,N’’*-triacetyl-*Z*-L-fusarinine B (**5**) in CD$_3$OD....48

Figure S55. 2D-HSQC-NMR spectrum of *N,N’,N’’*-triacetyl-*Z*-L-fusarinine B (**5**) in CD$_3$OD....49

Figure S56. 2D-HMBC-NMR spectrum of *N,N’,N’’*-triacetyl-*Z*-L-fusarinine B (**5**) in CD$_3$OD 49

Table S14. NMR chemical shifts of *N,N’,N’’*-triacetyl-*Z*-L-fusarinine B (**5**) in (CD$_3$)$_2$SO (400 MHz)....50

Figure S57. ^{1}H-^{1}H COSY (▬) and ^{1}H-^{13}C HMBC (→) correlations of *N,N’,N’’*-triacetyl-*Z*-L-fusarinine B (**5**) in (CD$_3$)$_2$SO 51

Figure S58. ^{1}H-NMR spectrum of *N,N’,N’’*-triacetyl-*Z*-L-fusarinine B (**5**) in (CD$_3$)$_2$SO (400 MHz) 52

Figure S59. ^{13}C-NMR spectrum of *N,N’,N’’*-triacetyl-*Z*-L-fusarinine B (**5**) in (CD$_3$)$_2$SO (100 MHz) 52

Figure S60. DEPT135-NMR spectrum of *N,N’,N’’*-triacetyl-*Z*-L-fusarinine B (**5**) in (CD$_3$)$_2$SO (100 MHz) 53

Figure S61. 2D-COSY-NMR spectrum of *N,N’,N’’*-triacetyl-*Z*-L-fusarinine B (**5**) in (CD$_3$)$_2$SO 53

Figure S62. 2D-HSQC-NMR spectrum of *N,N’,N’’*-triacetyl-*Z*-L-fusarinine B (**5**) in (CD$_3$)$_2$SO 54

Figure S63. 2D-HMBC-NMR spectrum of *N,N’,N’’*-triacetyl-*Z*-L-fusarinine B (**5**) in (CD$_3$)$_2$SO 54

N,N’-diacetyl-*Z*-L-fusarinine A Ga^{3+} complex (6-Ga) ____ 55

Table S15. Major *m/z* ions in (+) and (-) HRMS-ESI of *N,N’*-diacetyl-*Z*-L-fusarinine A Ga^{3+} complex (**6-Ga**) 55

Figure S64. (+) HRMS-ESI of *N,N’*-diacetyl-*Z*-L-fusarinine A Ga^{3+} complex (**6-Ga**)....55

Figure S65. (-) HRMS-ESI of *N,N'*-diacetyl-*Z*-L-fusarinine A Ga^{3+} complex (**6-Ga**)....56
Table S16. NMR chemical shifts of *N,N'*-diacetyl-*Z*-L-fusarinine A Ga^{3+} complex (**6-Ga**) in CD_3OD (500 MHz) 57
Figure S66. ^1H-^1H COSY (▬) and ^1H-^{13}C HMBC (⟶) correlations of *N,N'*-diacetyl-*Z*-L-fusarinine A Ga^{3+} complex (**6-Ga**) in CD_3OD....58
Figure S67. ^1H-NMR spectrum of *N,N'*-diacetyl-*Z*-L-fusarinine A Ga^{3+} complex (**6-Ga**) in CD_3OD (500 MHz)...59
Figure S68. ^{13}C-NMR spectrum of *N,N'*-diacetyl-*Z*-L-fusarinine A Ga^{3+} complex (**6-Ga**) in CD_3OD (125 MHz) .59
Figure S69. DEPT135-NMR spectrum of *N,N'*-diacetyl-*Z*-L-fusarinine A Ga^{3+} complex (**6-Ga**) in CD_3OD (125 MHz)....60
Figure S70. 2D-COSY-NMR spectrum of *N,N'*-diacetyl-*Z*-L-fusarinine A Ga^{3+} complex (**6-Ga**) in CD_3OD.........60
Figure S71. 2D-HSQC-NMR spectrum of *N,N'*-diacetyl-*Z*-L-fusarinine A Ga^{3+} complex (**6-Ga**) in CD_3OD.........61
Figure S72. 2D-HMBC-NMR spectrum of *N,N'*-diacetyl-*Z*-L-fusarinine A Ga^{3+} complex (**6-Ga**) in CD_3OD........61

N,N'-diacetyl-*Z*-L-fusarinine A (6) 62

Table S17. Major *m/z* ions in (+) and (-) HRMS-ESI of *N,N'*-diacetyl-*Z*-L-fusarinine A (**6**)....62
Figure S73. (+) HRMS-ESI of *N,N'*-diacetyl-*Z*-L-fusarinine A (**6**)....62
Figure S74. (-) HRMS-ESI of *N,N'*-diacetyl-*Z*-L-fusarinine A (**6**)....63
Table S18. NMR chemical shifts of *N,N'*-diacetyl-*Z*-L-fusarinine A (**6**) in CD_3OD (400 MHz)....64
Figure S75. ^1H-^1H COSY (▬) and ^1H-^{13}C HMBC (⟶) correlations of *N,N'*-diacetyl-*Z*-L-fusarinine A (**6**) in CD_3OD....65
Figure S76. ^1H-NMR spectrum of *N,N'*-diacetyl-*Z*-L-fusarinine A (**6**) in CD_3OD (400 MHz)....66
Figure S77. ^{13}C-NMR spectrum of *N,N'*-diacetyl-*Z*-L-fusarinine A(**6**) in CD_3OD (100 MHz)....66
Figure S78. DEPT135-NMR spectrum of *N,N'*-diacetyl-*Z*-L-fusarinine A (**6**) in CD_3OD (100 MHz)....67
Figure S79. 2D-COSY-NMR spectrum of *N,N'*-diacetyl-*Z*-L-fusarinine A (**6**) in CD_3OD....67
Figure S80. 2D-HSQC-NMR spectrum of *N,N'*-diacetyl-*Z*-L-fusarinine A (**6**) in CD_3OD....68
Figure S81. 2D-HMBC-NMR spectrum of *N,N'*-diacetyl-*Z*-L-fusarinine A (**6**) in CD_3OD....68
Table S19. NMR chemical shifts of *N,N'*-diacetyl-*Z*-L-fusarinine A (**6**) $(CD_3)_2SO$ (400 MHz)....69
Figure S82. ^1H-^1H COSY (▬) and ^1H-^{13}C HMBC (⟶) correlations of *N,N'*-diacetyl-*Z*-L-fusarinine A (**6**) in $(CD_3)_2SO$....70
Figure S83. ^1H-NMR spectrum of *N,N'*-diacetyl-*Z*-L-fusarinine A (**6**) in $(CD_3)_2SO$ (400 MHz)....71
Figure S84. ^{13}C-NMR spectrum of *N,N'*-diacetyl-*Z*-L-fusarinine A (**6**) in $(CD_3)_2SO$ (100 MHz)....71
Figure S85. DEPT135-NMR spectrum of *N,N'*-diacetyl-*Z*-L-fusarinine A (**6**) in $(CD_3)_2SO$ (100 MHz)....72
Figure S86. 2D-COSY-NMR spectrum of *N,N'*-diacetyl-*Z*-L-fusarinine A (**6**) in $(CD_3)_2SO$....72
Figure S87. 2D-HSQC-NMR spectrum of *N,N'*-diacetyl-*Z*-L-fusarinine A (**6**) in $(CD_3)_2SO$....73
Figure S88. 2D-HMBC-NMR spectrum of *N,N'*-diacetyl-*Z*-L-fusarinine A (**6**) in $(CD_3)_2SO$....73

N,N'-diacetyl-*Z*-L-fusarinine B Ga^{3+} complex (7-Ga) 74

Table S20. Major *m/z* ions in the (+) and (-) HRMS-ESI of *N,N'*-diacetyl-*Z*-L-fusarinine B Ga^{3+} complex (**7-Ga**) 74

Figure S89. (+) HRMS-ESI of *N,N'*-diacetyl-*Z*-L-fusarinine B Ga^{3+} complex (**7-Ga**)....74
Figure S90. (-) HRMS-ESI of *N,N'*-diacetyl-*Z*-L-fusarinine B Ga^{3+} complex (**7-Ga**)....75
Table S21. NMR chemical shifts of *N,N'*-diacetyl-*Z*-L-fusarinine B Ga^{3+} complex (**7-Ga**) in CD_3OD (500 MHz) 76
Figure S91. ^{1}H-NMR spectrum of *N,N'*-diacetyl-*Z*-L-fusarinine B Ga^{3+} complex (**7-Ga**) in CD_3OD (500 MHz)..77
Figure S92. ^{13}C-NMR spectrum of *N,N'*-diacetyl-*Z*-L-fusarinine B Ga^{3+} complex (**7-Ga**) in CD_3OD (125 MHz)..77
Figure S93. DEPT135-NMR spectrum of *N,N'*-diacetyl-*Z*-L-fusarinine B Ga^{3+} complex (**7-Ga**) in CD_3OD (125 MHz)....78
Figure S94. 2D-COSY-NMR spectrum of *N,N'*-diacetyl-*Z*-L-fusarinine B Ga^{3+} complex (**7-Ga**) in CD_3OD....78
Figure S95. 2D-HSQC-NMR spectrum of *N,N'*-diacetyl-*Z*-L-fusarinine B Ga^{3+} complex (**7-Ga**) in CD_3OD....79
Figure S96. 2D-HMBC-NMR spectrum of *N,N'*-diacetyl-*Z*-L-fusarinine B Ga^{3+} complex (**7-Ga)** in CD_3OD....79
Table S22. NMR chemical shifts of N,N'-diacetyl-Z-L-fusarinine B Ga^{3+} complex (**7-Ga**) in $(CD_3)_2SO$ (500 MHz) 80
Figure S97. ^{1}H-^{1}H COSY (▬) and ^{1}H-^{13}C HMBC (→) correlations of *N,N'*-diacetyl-*Z*-L-fusarinine B Ga^{3+} complex (**7-Ga**) in $(CD_3)_2SO$ 81
Figure S98. ^{1}H-NMR spectrum of *N,N'*-diacetyl-*Z*-L-fusarinine B Ga^{3+} complex (**7-Ga**) in $(CD_3)_2SO$ (500 MHz)82
Figure S99. ^{13}C-NMR spectrum of *N,N'*-diacetyl-*Z*-L-fusarinine B Ga^{3+} complex (**7-Ga**) in $(CD_3)_2SO$ (125 MHz) 82
Figure S100. DEPT-NMR spectrum of *N,N'*-diacetyl-*Z*-L-fusarinine B Ga^{3+} complex (**7-Ga**) in $(CD_3)_2SO$ (125 MHz)....83
Figure S101. 2D-COSY-NMR spectrum of *N,N'*-diacetyl-*Z*-L-fusarinine B Ga^{3+} complex (**7-Ga**) in $(CD_3)_2SO$...83
Figure S102. 2D-HSQC-NMR spectrum of *N,N'*-diacetyl-*Z*-L-fusarinine B Ga^{3+} complex (**7-Ga**) in $(CD_3)_2SO$...84
Figure S103. 2D-HMBC-NMR spectrum of *N,N'*-diacetyl-*Z*-L-fusarinine B Ga^{3+} complex (**7-Ga**) in $(CD_3)_2SO$...84

***N,N'*-diacetyl-*Z*-L-fusarinine B (7)** 85

Table S23. Major *m/z* ions in the (+) and (-) HRMS-ESI of *N,N'*-diacetyl-*Z*-L-fusarinine B (**7**) 85
Figure S104. (+) HRMS-ESI of *N,N'*-diacetyl-*Z*-L-fusarinine B (**7**) 85
Figure S105. (-) HRMS-ESI of *N,N'*-diacetyl-*Z*-L-fusarinine B (**7**) 86

***N*-acetyl- *Z*-L-fusarinine A Ga^{3+} complex (8-Ga)** 87

Table S24. Major *m/z* ions in the (+) and (-) HRMS-ESI of *N*-acetyl-*Z*-L-fusarinine A Ga^{3+} complex (**8-Ga**)....87
Figure S106. (+) HRMS-ESI of *N*-acetyl-*Z*-L-fusarinine A Ga^{3+} complex (**8-Ga**)....87
Figure S107. (-) HRMS-ESI of *N*-acetyl-*Z*-L-fusarinine A Ga^{3+} complex (**8-Ga**)....88
Table S25. NMR chemical shifts of *N*-acetyl-*Z*-L-fusarinine A Ga^{3+} complex (**8-Ga**) in CD_3OD (500 MHz) 89
Figure S108. ^{1}H-NMR spectrum of *N*-acetyl-*Z*-L-fusarinine A Ga^{3+} complex (**8-Ga**) in CD_3OD (500 MHz)....90
Figure S109. ^{13}C-NMR spectrum of *N*-acetyl-*Z*-L-fusarinine A Ga^{3+} complex (**8-Ga**) in CD_3OD (125 MHz)....90
Figure S110. DEPT135-NMR spectrum of *N*-acetyl-*Z*-L-fusarinine A Ga^{3+} complex (**8-Ga**) in CD_3OD (125 MHz) 91
Figure S111. 2D-COSY-NMR spectrum of *N*-acetyl-*Z*-L-fusarinine A Ga^{3+} complex (**8-Ga**) in CD_3OD....91

Figure S112. 2D-HSQC-NMR spectrum of *N*-acetyl-*Z*-L-fusarinine A Ga^{3+} complex (**8-Ga**) in CD_3OD.............92
Figure S113. 2D-HMBC-NMR spectrum of *N*-acetyl-*Z*-L-fusarinine A Ga^{3+} complex (**8-Ga**) in CD_3OD.............92
Table S26. NMR chemical shifts of *N*-acetyl-*Z*-L-fusarinine A Ga^{3+} complex (**8-Ga**) in $(CD_3)_2SO$ (500 MHz).....93
Figure S114. ^1H-^1H COSY (▬) and ^1H-^{13}C HMBC (⟶) correlations of *N*-acetyl-*Z*-L-fusarinine A Ga^{3+} complex (**8-Ga**) in $(CD_3)_2SO$ 94
Figure S115. ^1H-NMR spectrum of *N*-acetyl-*Z*-L-fusarinine A Ga^{3+} complex (**8-Ga**) in $(CD_3)_2SO$ (500 MHz)......95
Figure S116. ^{13}C-NMR spectrum of *N*-acetyl-*Z*-L-fusarinine A Ga^{3+} complex (8-Ga) in $(CD_3)_2SO$ (125 MHz).....95
Figure S117. DEPT135-NMR spectrum of *N*-acetyl-*Z*-L-fusarinine A Ga^{3+} complex (**8-Ga**) in $(CD_3)_2SO$ (125 MHz)....................96
Figure S118. 2D-COSY-NMR spectrum of *N*-acetyl-*Z*-L-fusarinine A Ga^{3+} complex (**8-Ga**) in $(CD_3)_2SO$ 96
Figure S119. 2D-HSQC-NMR spectrum of *N*-acetyl-*Z*-L-fusarinine A Ga^{3+} complex (**8-Ga**) in $(CD_3)_2SO$ 97
Figure S120. 2D-HMBC-NMR spectrum of *N*-acetyl-*Z*-L-fusarinine A Ga^{3+} complex (**8-Ga**) in $(CD_3)_2SO$ 97

N-acetyl- *Z*-L-fusarinine A (8) 98

Table S27. Major *m/z* ions in the (+) HRMS-ESI of *N*-acetyl-*Z*-L-fusarinine A (**8**)....................98
Figure S121. (+) HRMS-ESI of *N*-acetyl-*Z*-L-fusarinine A (**8**) 98
Table S28. NMR chemical shifts of *N*-acetyl-*Z*-L-fusarinine A (**8**) in CD_3OD (400 MHz)....................99
Figure S122. ^1H-^1H COSY (▬) and ^1H-^{13}C HMBC (⟶) correlations of *N*-acetyl-*Z*-L-fusarinine A (**8**) in CD_3OD 100
Figure S123. ^1H-NMR spectrum of *N*-acetyl-*Z*-L-fusarinine A (**8**) in CD_3OD (400 MHz)....................101
Figure S124. ^{13}C-NMR spectrum of *N*-acetyl-*Z*-L-fusarinine A (**8**) in CD_3OD (100 MHz)....................101
Figure S125. DEPT135-NMR spectrum of *N*-acetyl-*Z*-L-fusarinine A (**8**) in CD_3OD (100 MHz) 102
Figure S126. 2D-COSY-NMR spectrum of *N*-acetyl-*Z*-L-fusarinine A (**8**) in CD_3OD 102
Figure S127. 2D-HSQC-NMR spectrum of *N*-acetyl-*Z*-L-fusarinine A (**8**) in CD_3OD 103
Figure S128. 2D-HMBC-NMR spectrum of *N*-acetyl-*Z*-L-fusarinine A (**8**) in CD_3OD....................103
Table S29. NMR chemical shifts of N-acetyl-Z-L-fusarinine A (**8**) in $(CD_3)_2SO$ (400 MHz)....................104
Figure S129. ^1H-^1H COSY (▬) and ^1H-^{13}C HMBC (⟶) correlations of *N*-acetyl-*Z*-L-fusarinine A (**8**) in $(CD_3)_2SO$ 105
Figure S130. ^1H-NMR spectrum of *N*-acetyl-*Z*-L-fusarinine A (**8**) in $(CD_3)_2SO$ (400 MHz)....................106
Figure S131. ^{13}C-NMR spectrum of *N*-acetyl-*Z*-L-fusarinine A (**8**) in $(CD_3)_2SO$ (100 MHz)....................106
Figure S132. DEPT135-NMR spectrum of *N*-acetyl-*Z*-L-fusarinine A (**8**) in $(CD_3)_2SO$ (100 MHz)....................107
Figure S133. 2D-COSY-NMR spectrum of *N*-acetyl-*Z*-L-fusarinine A (**8**) in $(CD_3)_2SO$....................107
Figure S134. 2D-HSQC-NMR spectrum of *N*-acetyl-*Z*-L-fusarinine A (**8**) in $(CD_3)_2SO$....................108
Figure S135. 2D-HMBC-NMR spectrum of *N*-acetyl-*Z*-L-fusarinine A (**8**) in $(CD_3)_2SO$....................108

4-methyl-5,6-dihydro-2H-pyran-2-one 109

Table S30. Major *m/z* ions in the (+) HRMS-ESI of 4-methyl-5,6-dihydro-2H-pyran-2-one 109

Figure S136. (+) HRMS-ESI of 4-methyl-5,6-dihydro-2H-pyran-2-one 109

Table S31. NMR chemical shifts of 4-methyl-5,6-dihydro-2H-pyran-2-one in CD_3OD (400 MHz).....................110

Figure S137. ^{1}H-^{1}H COSY (▬) and ^{1}H-^{13}C HMBC (→) correlations of 4-methyl-5,6-dihydro-2H-pyran-2-one in CD_3OD...110

Figure S138. ^{1}H-NMR spectrum of 4-methyl-5,6-dihydro-2H-pyran-2-one in CD_3OD (400 MHz) 111

Figure S139. ^{13}C-NMR spectrum of 4-methyl-5,6-dihydro-2H-pyran-2-one in CD_3OD (100 MHz) 111

Figure S140. DEPT135-NMR spectrum of 4-methyl-5,6-dihydro-2H-pyran-2-one in CD_3OD (100 MHz) 112

Figure S141. 2D-COSY-NMR spectrum of 4-methyl-5,6-dihydro-2H-pyran-2-one in CD_3OD 112

Figure S142. 2D-HSQC-NMR spectrum of 4-methyl-5,6-dihydro-2H-pyran-2-one in CD_3OD 113

Figure S143. 2D-HMBC-NMR spectrum of 4-methyl-5,6-dihydro-2H-pyran-2-one in CD_3OD 113

Marfey's reaction ______________________________________ 114

Figure S144. Analysis by advance Marfey´s method of 1-8: HPLC chromatograms with UV detector at 360 nm showing the L-FDAA derivatives of D- and L-ornithine standards and the hydrolyzed products isolated from the fungus P. verrucosus FAE27. Diastereoisomers with a retention time of 16.3 min, corresponding to those formed with L-ornithine, are highlighted with a red box. 115

Table S1. LC/(+)-HRESIMS analysis of fractions from broth extracts of *P. verrucosus* FAE27 grown under iron-limiting conditions.

Compound	Identified siderophore	Form	RT (min)	Amount (mg)	*m/z* observed
**1**	fusarinine	fusarinine	5.579	52.9	[M+Na]$^+$ 283.1265<br>[MCOONa+Na]$^+$ 305.1084
**2**	acetylfusarinine	acetylfusarinine	7.219	19.2	[M+Na]$^+$ 325.1371<br>[MCOONa+Na]$^+$ 347.1190
**3**	acetylfusarinine-methylester	acetylfusarinine-methylester	8.444	21.5	[M+Na]$^+$ 339.1524
**4**	TAFC	*holo*-TAFC (Ga^{3+} chelated)	10.137	4.6	[M-3H+$^{69/71}$Ga+Na]$^+$ 941.3059/943.3063<br>[M-3H+$^{69/71}$Ga+K]$^+$ 957.2797/959.2799
**5**	TAFB	*holo*-TAFB (Ga^{3+} chelated)	9.172	4.7	[M-3H+$^{69/71}$Ga+Na]$^+$ 959.3139/961.3139<br>[MCOONa+$^{69/71}$Ga+Na]$^+$ 981.2955/983.2955
		apo-TAFB	10.270	11.2	[M+Na]$^+$ 893.4132<br>[MCOONa+Na]$^+$ 915.3950
**6**	DAFA	*holo*-DAFA (Ga^{3+} chelated)	7.786	5.6	[M-2H+$^{69/71}$Ga]$^+$ 653.1948/655.1942<br>[MCOONa-2H+$^{69/71}$Ga]$^+$ 675.1767/677.1760
		apo-DAFA	9.198	13.9	[M+Na]$^+$ 609.2746<br>[M+K]$^+$ 625.2475
7	**n.d.	*holo*-siderophore 1 (Ga^{3+} chelated)	7.187	4.6	[M-2H+$^{69/71}$Ga]$^+$ 611.1838/613.1830<br>[MCOONa-2H+$^{69/71}$Ga]$^+$ 633.1657/635.1665
		apo-siderophore 1	8.035	19.9	[M+H]$^+$ 545.2822<br>[M+Na]$^+$ 567.2641
**8**	**n.d.	*holo*-siderophore 2 (Ga^{3+} chelated)	8.868	4.0	[M-3H+$^{69/71}$Ga+H]$^+$ 895.3231/897.3234<br>[MCOONa-3H+$^{69/71}$Ga]$^+$ 917.3050/919.3054
		apo-siderophore 2	9.279	1.1	[M+H]$^+$ 829.4190<br>[M+Na]$^+$ 851.4011

**n. d. No corresponding data found in the literature

Z-L-fusarinine (1)

Table S2. Major *m/z* ions in the (+) HRMS-ESI of *Z*-L-fusarinine (**1**)

Adduct	Observed *m/z*	Theoretical *m/z*	Error [ppm]
$[M+Na]^+$	283.1265	283.1270	1.8
$[MCOONa+Na]^+$	305.1084	305.1090	2.0

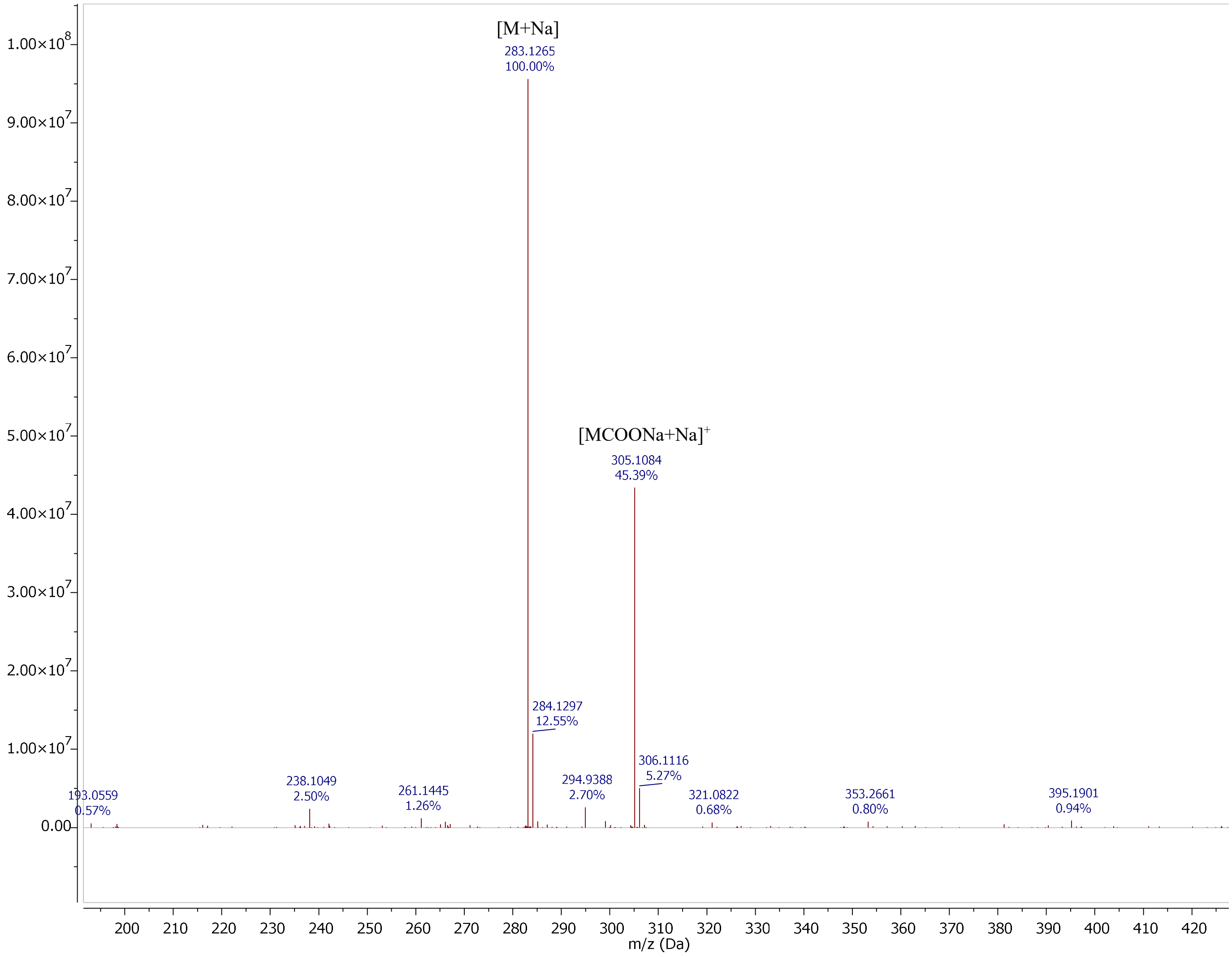

Figure S1. (+) HRMS-ESI of *Z*-L-fusarinine (**1**)

Table S3. NMR chemical shifts of *Z*-L-fusarinine (**1**) in CD_3OD (400 MHz)

Position	δ_C [a]mult	δ_H[b] mult (*J* in Hz)	∫	HMBC (H→C#)	COSY (H→H#)
**2**	55.65, CH	3.63, t (5.8)	1	3, 4, 5	4, 5
**3**	174.71, C				
**4**	29.34, CH_2	1.84 - 1.74, m<br>1.90 - 1.84, m	1<br>1	2, 3, 5, 6	2, 5
**5**	23.70, CH_2	1.84 - 1.74, m	2	2, 4, 6	2, 4
**6**	48.15, CH_2	3.69, dt (8.7, 2.6)	2	4, 5	4, 5
**8**	169.52, C				
**9**	118.95, CH	6.37, s	1	11, 16	16
**10**	152.29, C				
**11**	37.34, CH_2	2.71, t (6.4)	2	9, 10, 12, 16	12
**12**	61.22, CH_2	3.72, d (6.4)	2	10, 11	11
**16**	25.16, CH_2	1.92, d (1.4)	3	9, 10, 11	9

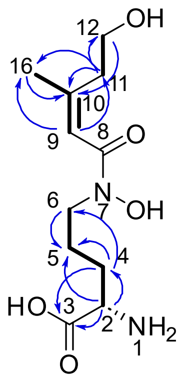

Figure S2. ^{1}H-^{1}H COSY (▬) and ^{1}H-^{13}C HMBC correlations (→) of Z-L-fusarinine (**1**) in CD_3OD

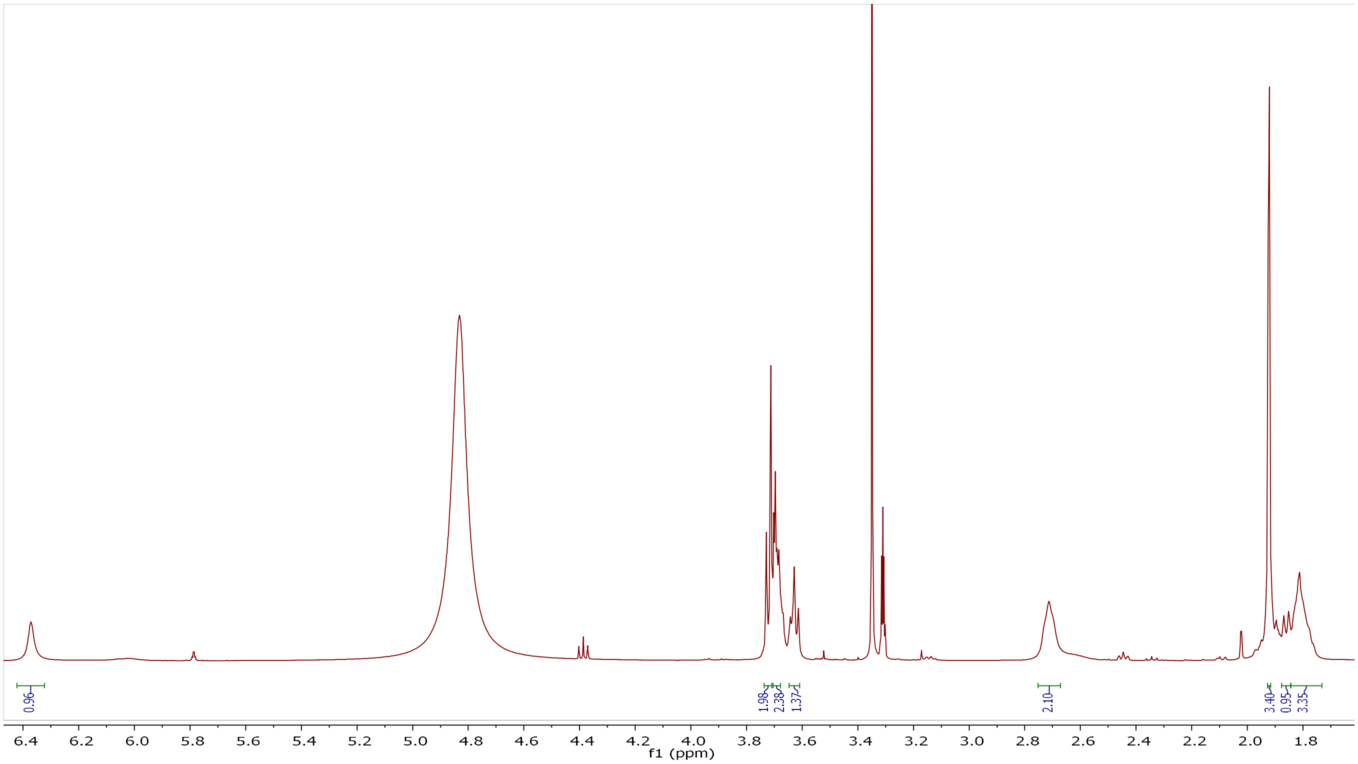

Figure S3. ^{1}H-NMR spectrum of *Z*-L-fusarinine (**1**) in CD_3OD (400 MHz)

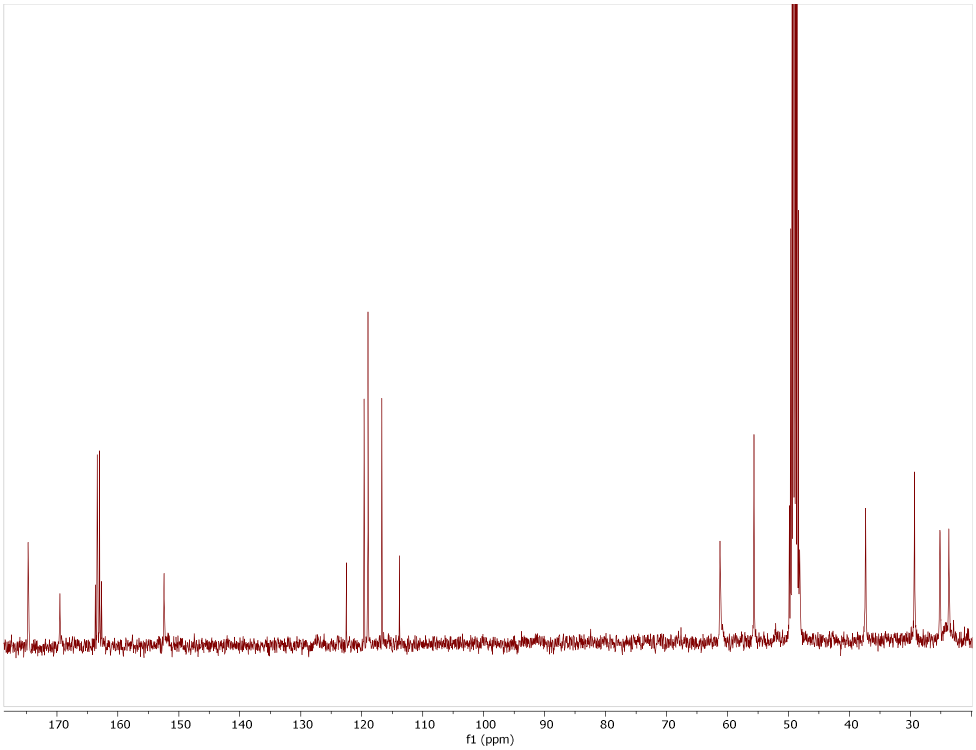

Figure S4. ^{13}C-NMR spectrum of *Z*-L-fusarinine (**1**) in CD_3OD (100 MHz)

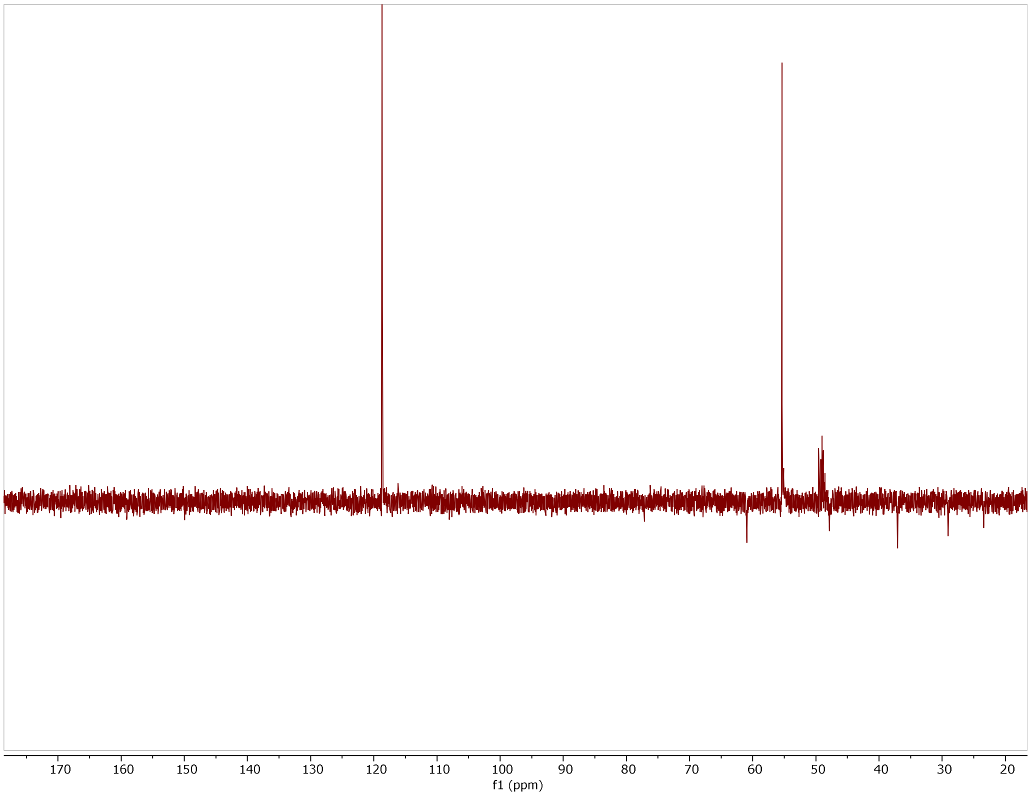

Figure S5. DEPT135-NMR spectrum of *Z*-L-fusarinine (**1**) in CD_3OD (100 MHz)

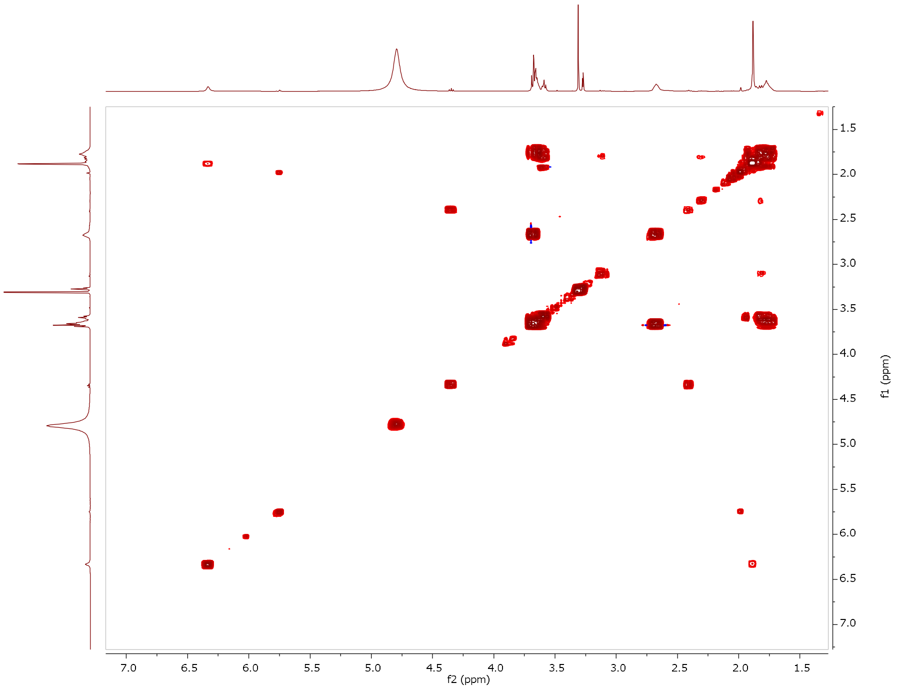

Figure S6. 2D-COSY-NMR spectrum of *Z*-L-fusarinine (**1**) in CD_3OD

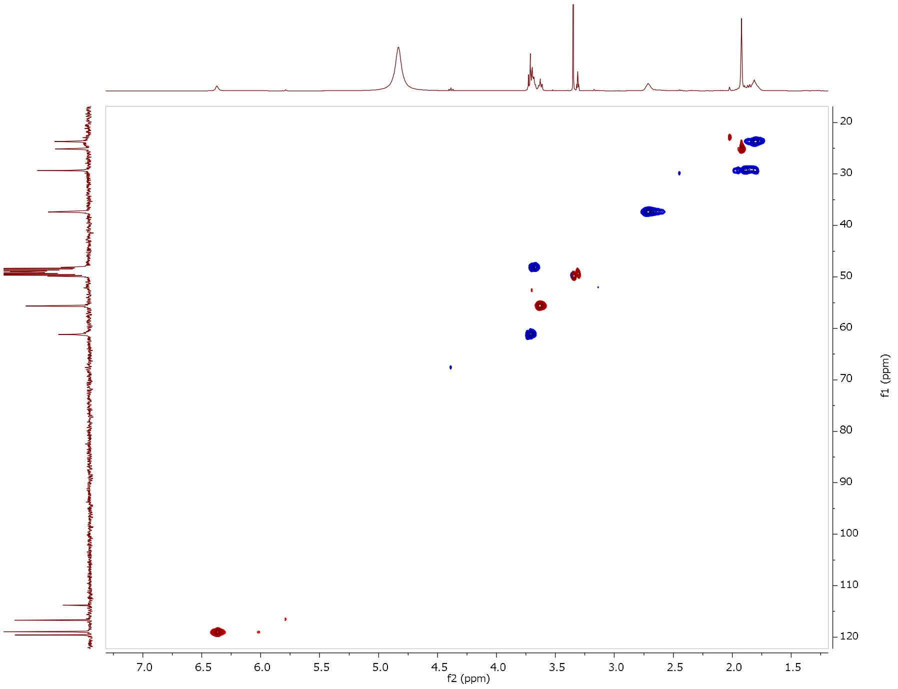

Figure S7. 2D-HSQC-NMR spectrum of *Z*-L-fusarinine (**1**) in CD_3OD

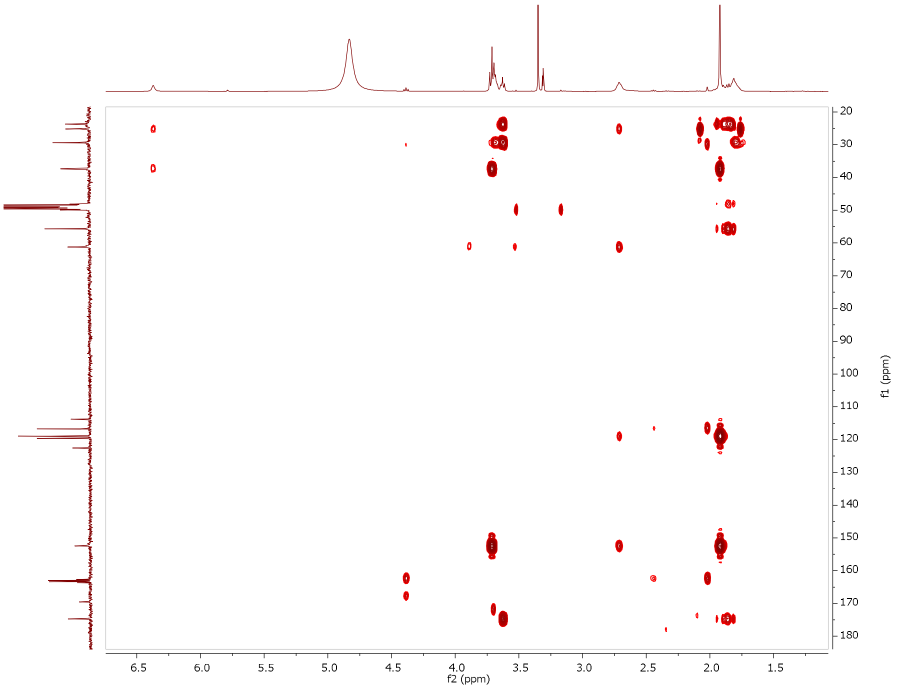

Figure S8. 2D-HMBC-NMR spectrum of *Z*-L-fusarinine (**1**) in CD_3OD

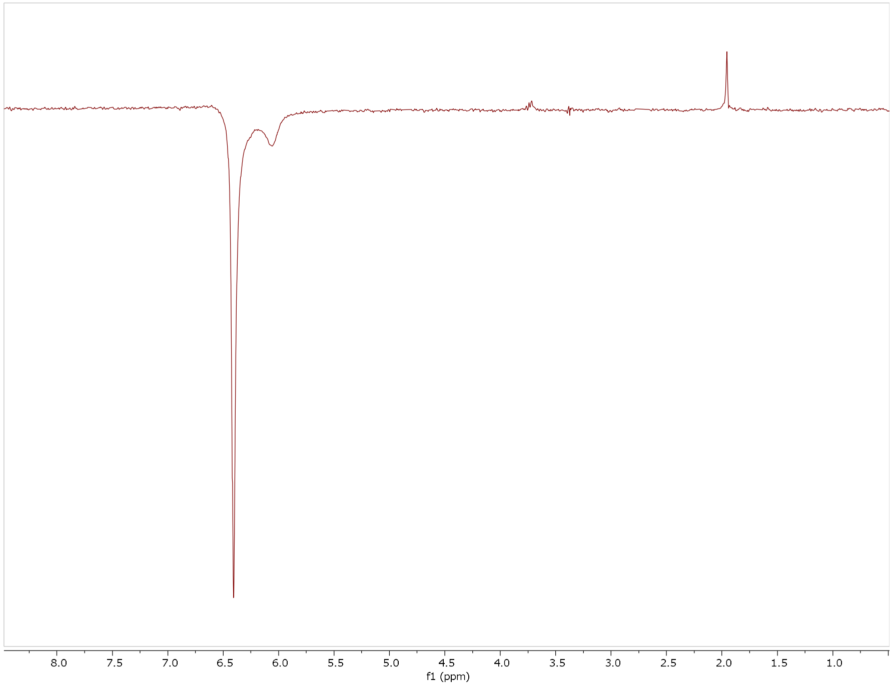

Figure S9. 1D-NOESY-NMR spectrum of *Z*-L-fusarinine (**1**) in CD_3OD (300 MHz), obtained band selective excitation of the signal at 6.37 ppm

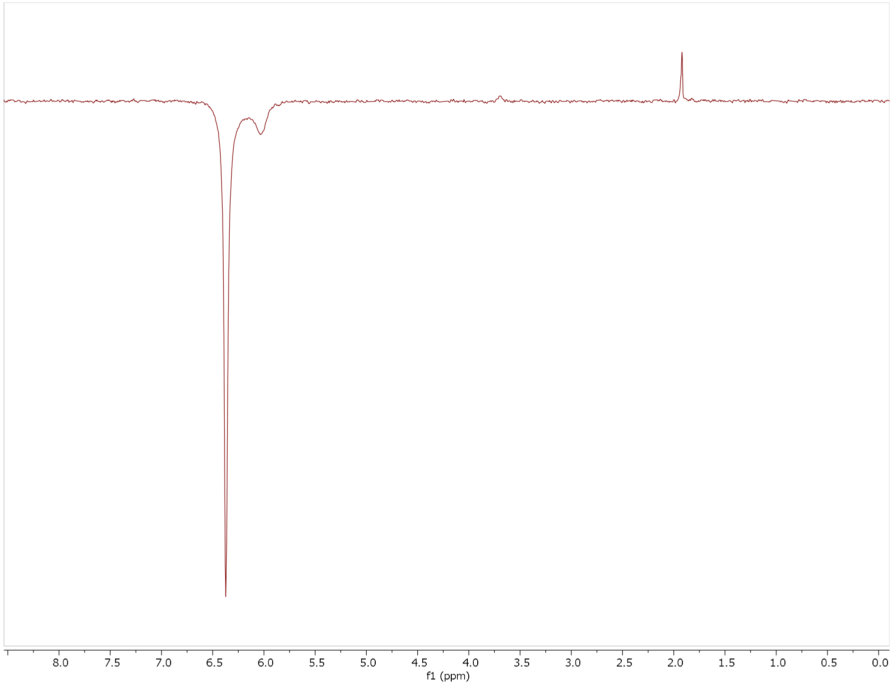

Figure S10. 1D-ROESY-NMR spectrum of *Z*-L-fusarinine (**1**) in CD_3OD (300 MHz), obtained band selective excitation of the signal at 6.37 ppm

N-acetyl-*Z*-L-fusarinine (2)

Table S4. Major *m/z* ions in the (+) HRMS-ESI of *N*-acetyl-*Z*-L-fusarinine (**2**)

Adduct	Observed *m/z*	Theoretical *m/z*	Error [ppm]
$[M+Na]^+$	325.1371	325.1376	1.5
$[MCOONa+Na]^+$	347.1190	347.1196	1.7

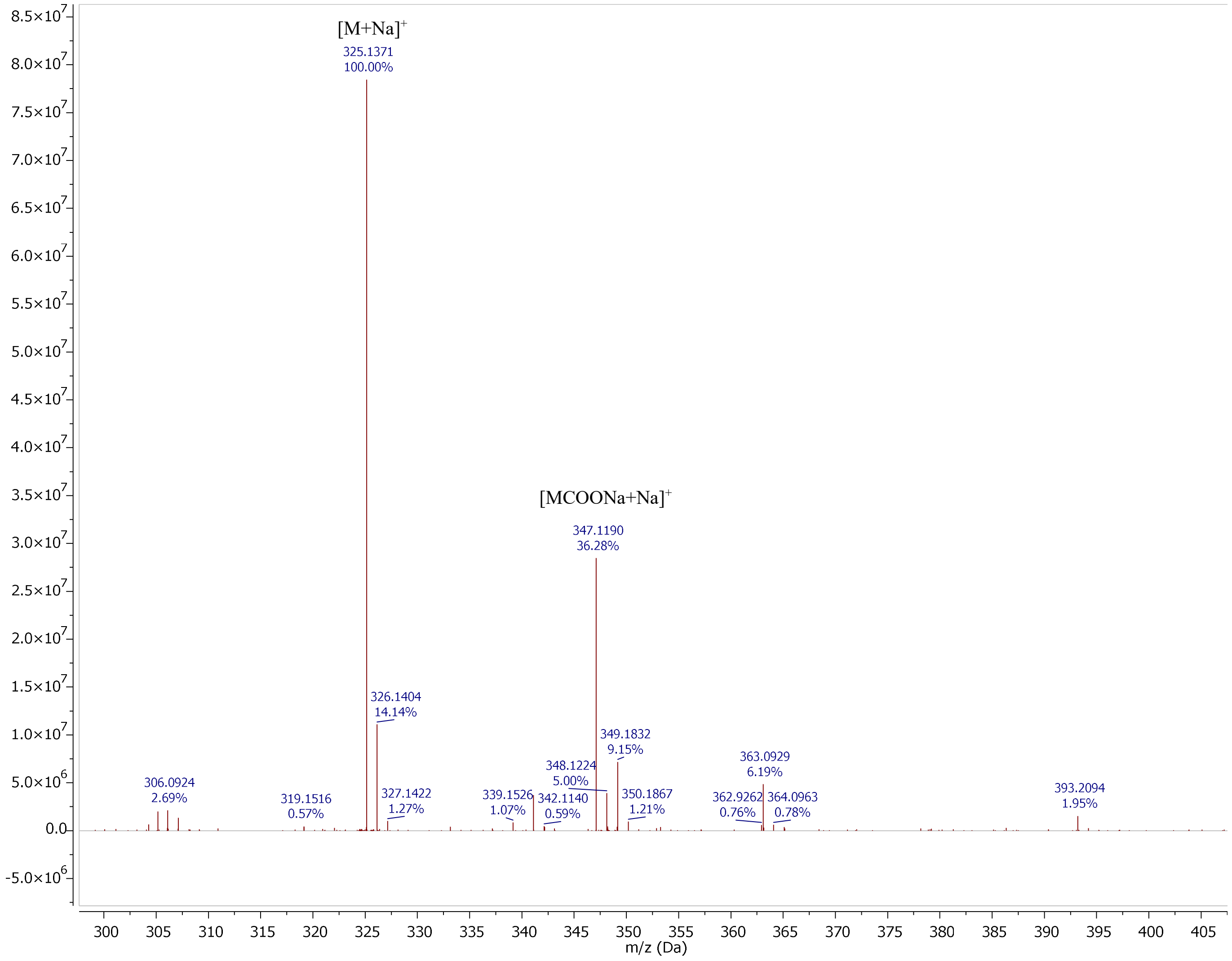

Figure S11. (+) HRMS-ESI of *N*-acetyl-*Z*-L-fusarinine (**2**)

Table S5. NMR chemical shifts of *N*-acetyl-*Z*-L-fusarinine (**2**) in CD_3OD (400 MHz)

Position	δ_C [a]mult	δ_H[b] mult (*J* in Hz)	∫	HMBC (H→C#)	COSY (H→H#)
**2**	55.47, CH	4.27, t	1	3, 4, 5	4, 5
**3**	178.24, C				
**4**	30.63, CH_2<br>30.63, CH_2	1.76 - 1.59, m<br>1.88 - 1.76, m	1<br>1	5	2, 4, 5, 6
**5**	24.43, CH_2	1.76 - 1.59, m	2	4	2, 4, 6
**6**	48.46, CH_2	3.65, td (6.7, 2.2)	2	4, 5	4, 5
**8**	169.43, C				
**9**	119.07, CH	6.36, s	1	11, 16	16
**10**	152.10, C				
**11**	37.40, CH_2	2.64 - 2.77, m	2	9, 10, 12, 16	12
**12**	61.21, CH_2	3.71, t (6.5)	2	10, 11	11
**14**	172.84, C				
**15**	22.65, CH_3	1.99, s	3	14	
**16**	25.16, CH_3	1.93, d (1.4)	3	9, 10, 11	9

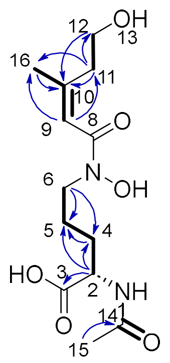

Figure S12. ^1H-^1H COSY and ^1H-^{13}C HMBC correlations (→) of *N*-acetyl-*Z*-L-fusarinine (**2**) in CD_3OD

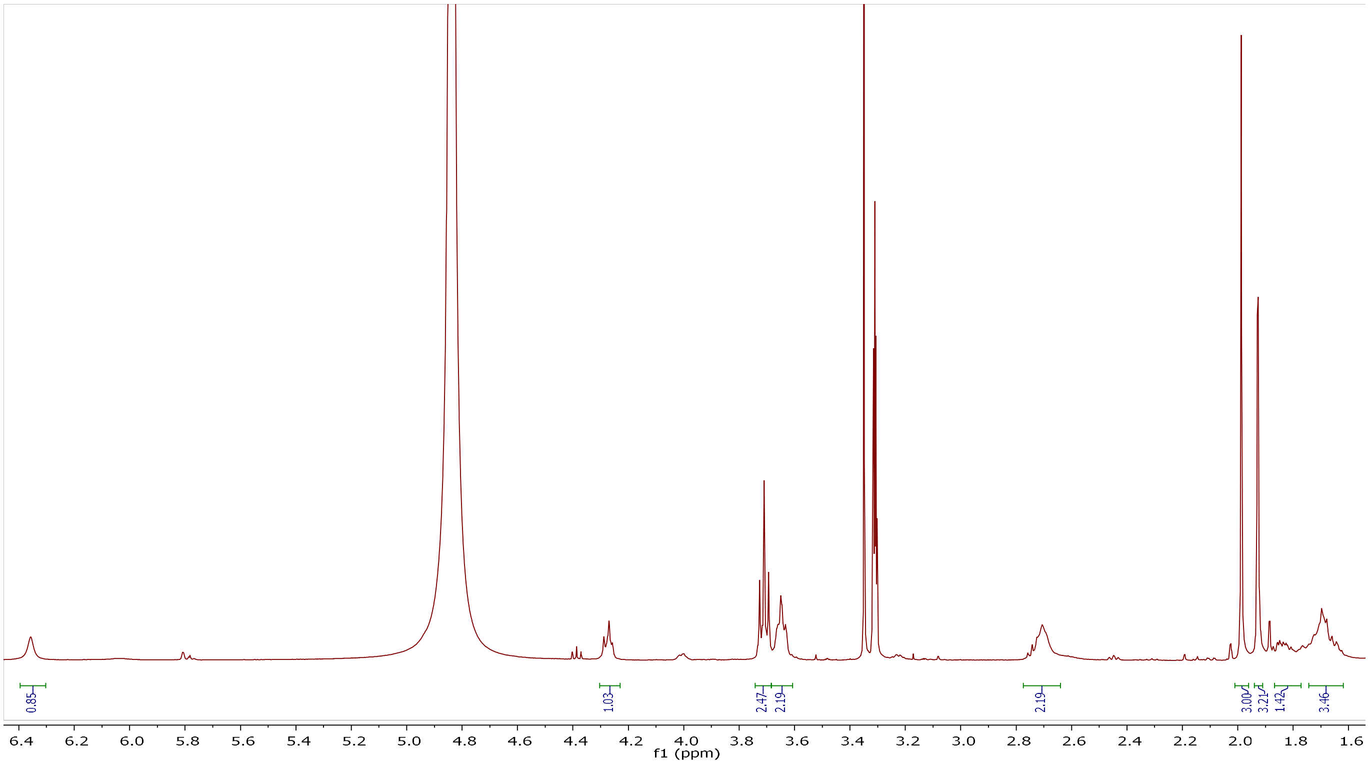

Figure S13. ^{1}H-NMR spectrum of *N*-acetyl-*Z*-L-fusarinine (**2**) in CD_3OD (400 MHz)

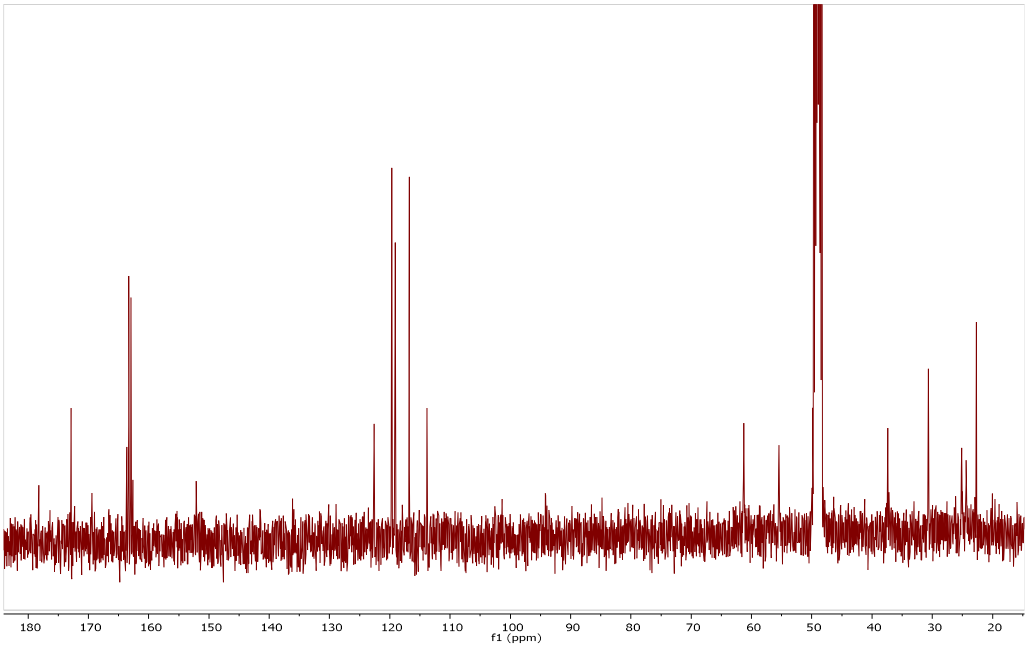

Figure S14. ^{13}C-NMR spectrum of *N*-acetyl-*Z*-L-fusarinine (**2**) in CD_3OD (100 MHz)

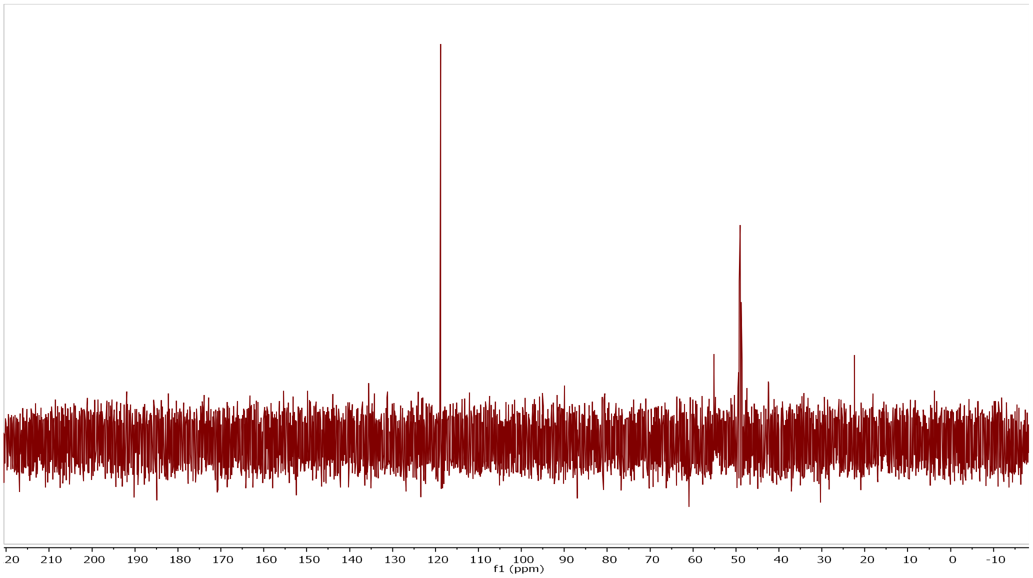

Figure S15. DEPT135-NMR spectrum of *N*-acetyl-*Z*-L-fusarinine (**2**) in CD_3OD (100 MHz)

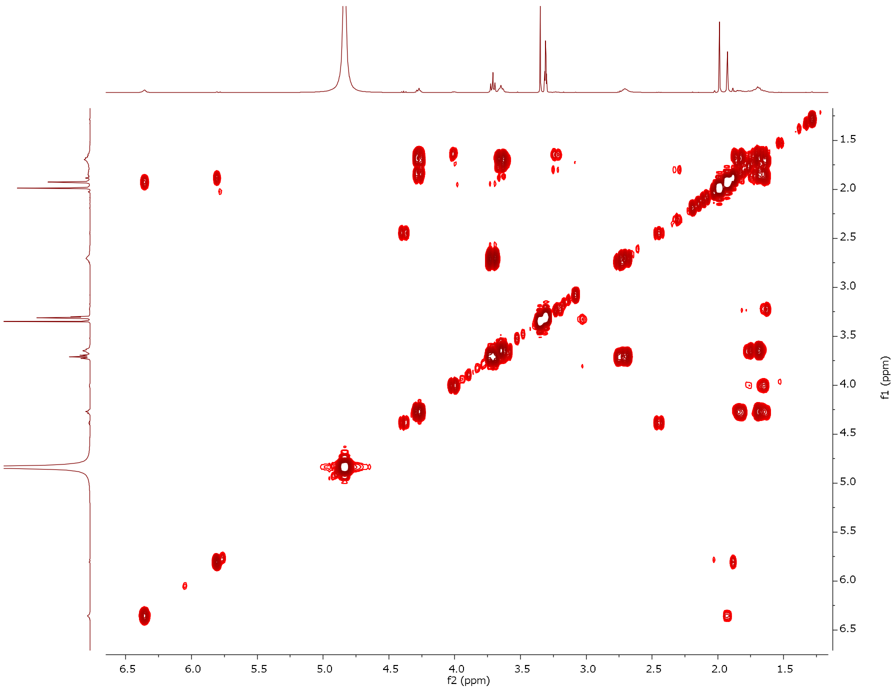

Figure S16. 2D-COSY-NMR spectrum of *N*-acetyl-*Z*-L-fusarinine (**2**) in CD_3OD

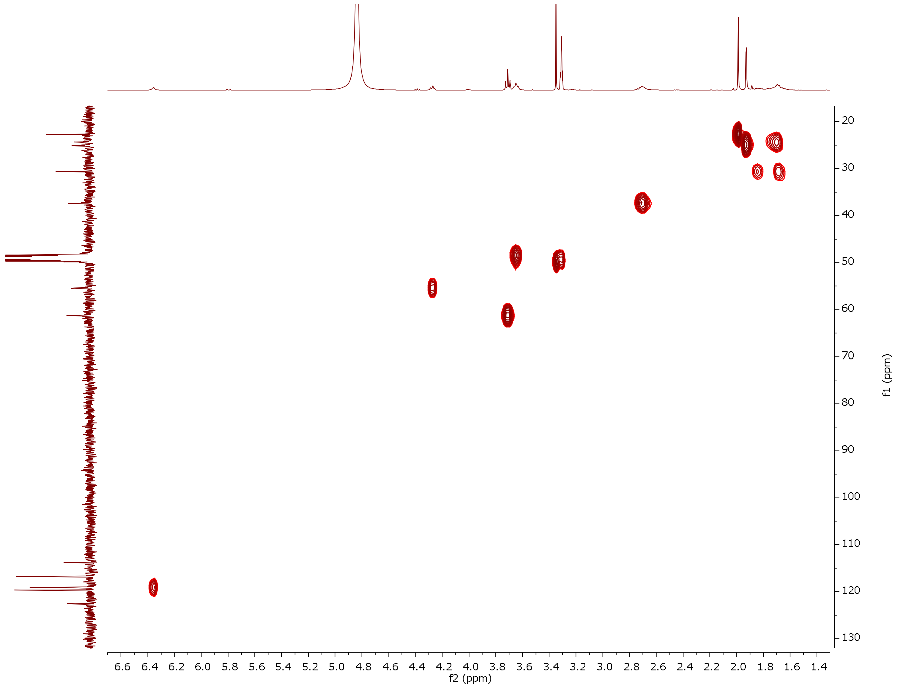

Figure S17. 2D-HSQC-NMR spectrum of *N*-acetyl-*Z*-L-fusarinine (2) in CD_3OD

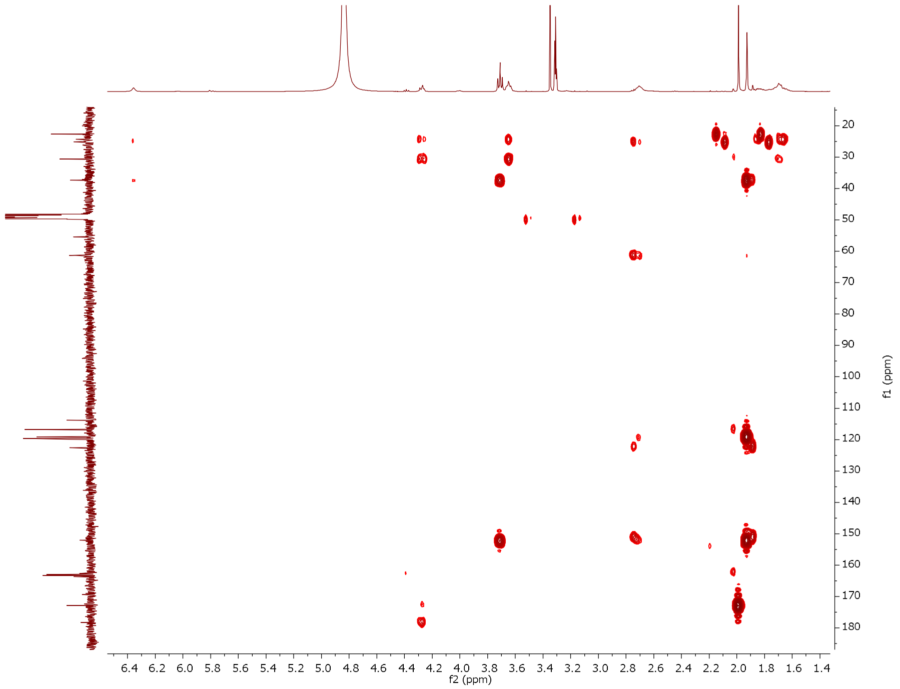

Figure S18. 2D-HMBC-NMR spectrum of *N*-acetyl-*Z*-L-fusarinine (**2**) in CD_3OD

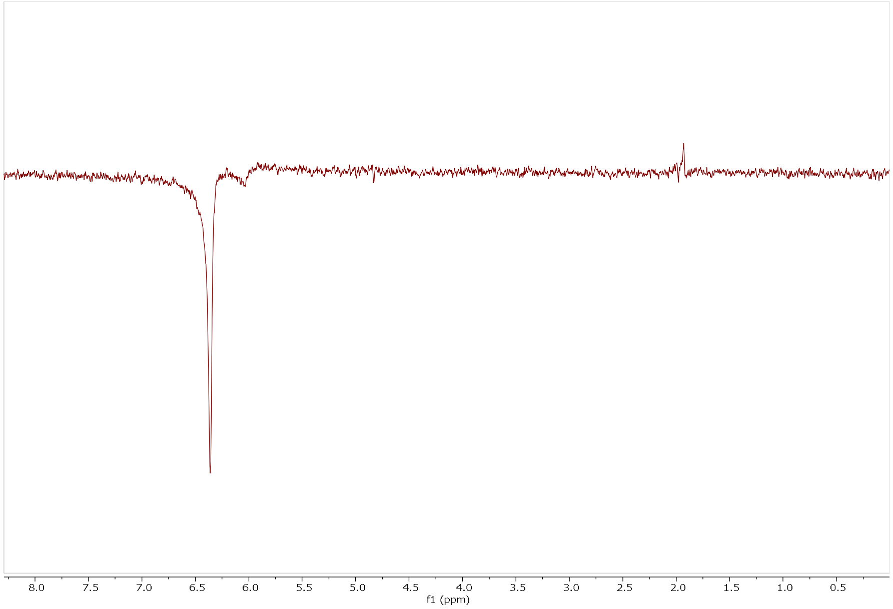

Figure S19. 1D-NOESY-NMR spectrum of *N*-acetyl-*Z*-L-fusarinine (**2**) in CD_3OD (300 MHz), obtained band selective excitation of the signal at 6.36 ppm

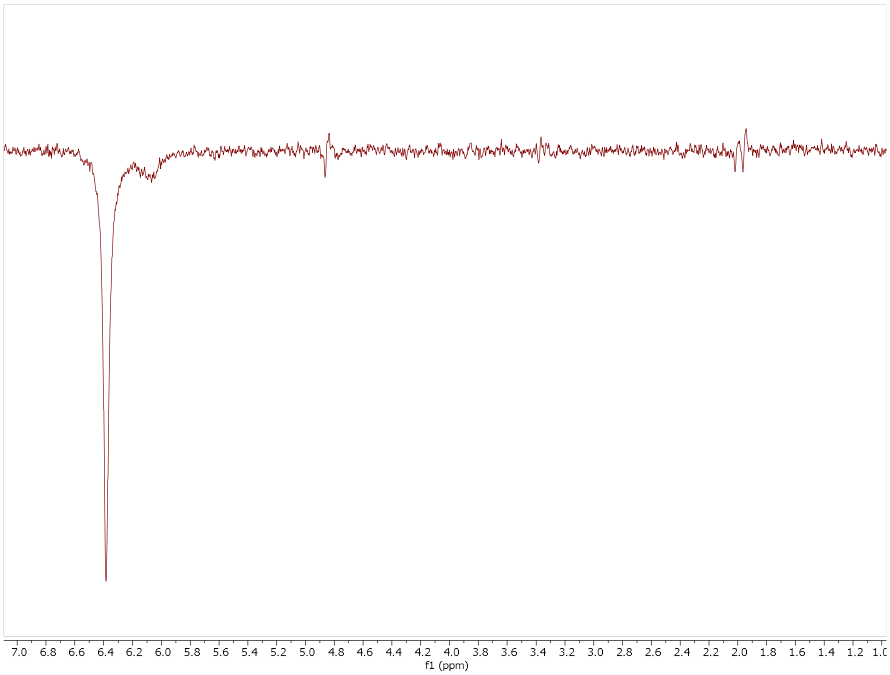

Figure S20. 1D-ROESY-NMR spectrum of *N*-acetyl-*Z*-L-fusarinine (**2**) in CD_3OD (300 MHz), obtained band selective excitation of the signal at 6.36 ppm

N-acetyl-*Z*-L-fusarinine-methylester (3)

Table S6. Major *m/z* ions in the (+) HRMS-ESI of *N*-acetyl-*Z*-L-fusarinine-methylester (**3**)

Adduct	Observed *m/z*	Theoretical *m/z*	Error [ppm]
$[M+Na]^+$	339.1524	339.1532	2.4

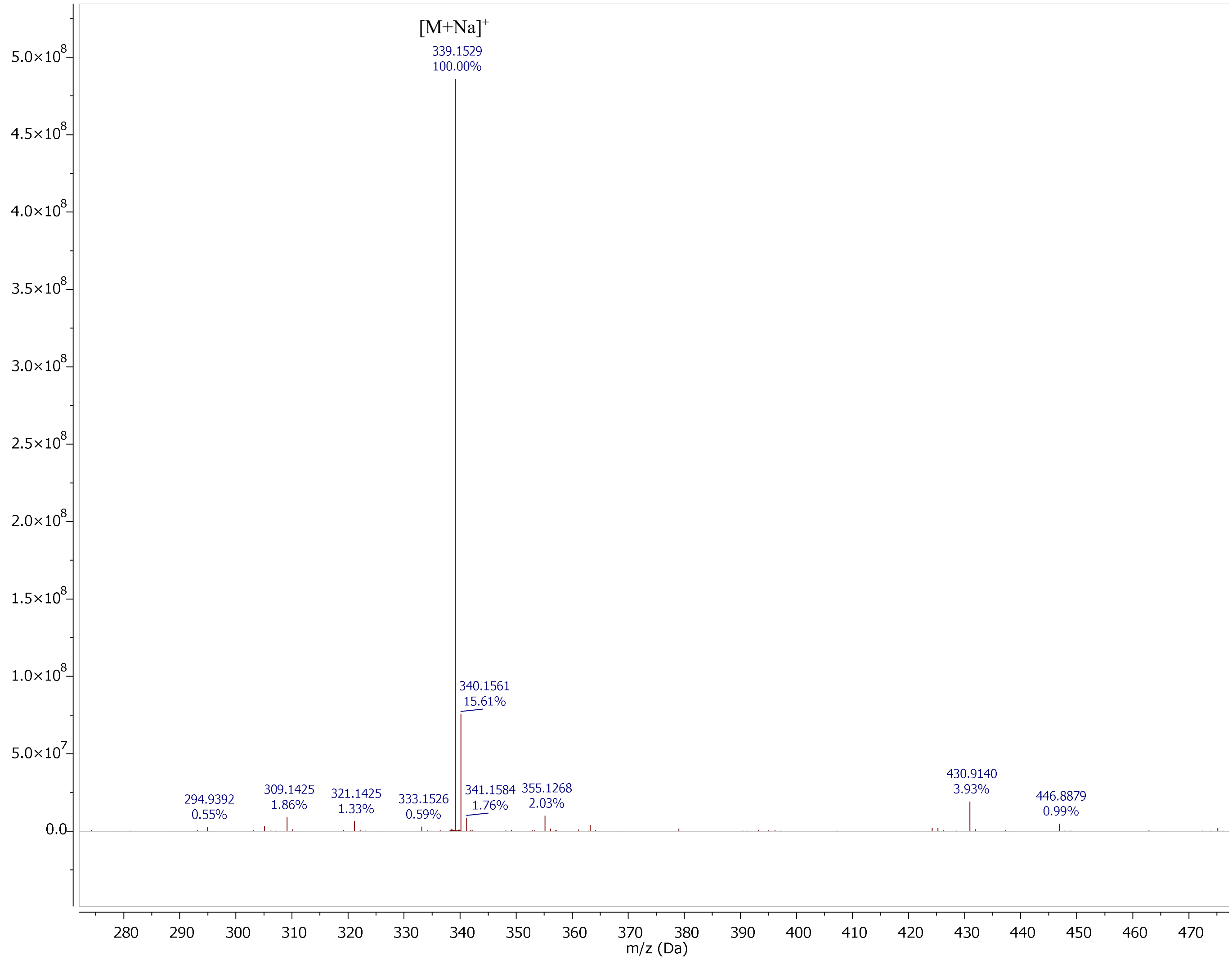

Figure S21. (+) HRMS-ESI of *N*-acetyl-*Z*-L-fusarinine-methylester (**3**)

Table S7. NMR chemical shifts of *N*-acetyl-*Z*-L-fusarinine-methylester (**3**) in CD_3OD (500 MHz)

Position	δ_C[a] mult	δ_H[b] mult (*J* in Hz)	∫	HMBC (H→C#)	COSY (H→H#)
**2**	53.60, CH	4.45 - 4.39, m	1	3, 4	4, 5
**3**	174.24, C				
**4**	29.49, CH_2	1.75 - 1.64, m<br>1.88 - 1.79, m	1<br>1	2, 5	2, 4, 5, 6
**5**	24.27, CH_2	1.75 - 1.64, m	2	6	2, 4, 6
**6**	48.03, CH_2	3.69 - 3.63, m	2		4, 5
**8**	169.45, C				
**9**	118.91, CH	6.37, s	1	11, 16	16
**10**	152.56, C				
**11**	37.34, CH_2	2.78 – 2.75, m	2	9, 10, 12, 16	12
**12**	61.33, CH_2	3.73 - 3.70, m	2	10, 11	11
**14**	173.45, C				
**15**	22.27, CH_3	1.99, brs	3	14	
**16**	25.27, CH_2	1.94, d (1.4)	3	9, 10, 11	9
**17**	52.75, CH_2	3.71, s	3	3	

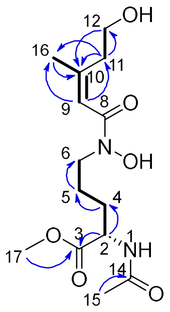

Figure S22. ^{1}H-^{1}H COSY (-) and ^{1}H-^{13}C HMBC (⟶) correlations of *N*-acetyl-*Z*-L-fusarinine-methyl ester (**3**) in CD_3OD

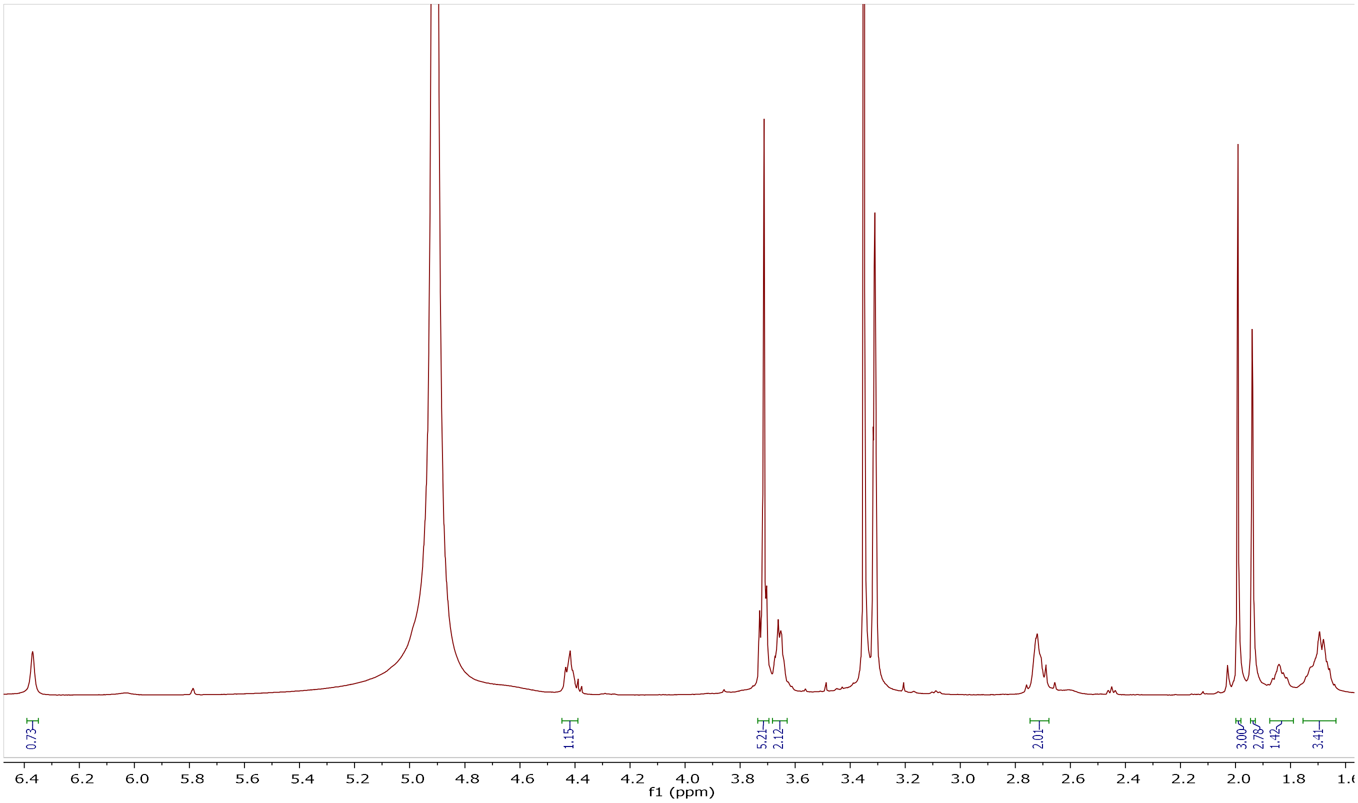

Figure S23. ^{1}H-NMR spectrum of *N*-acetyl-*Z*-L-fusarinine-methylester (**3**) in CD_3OD (500 MHz)

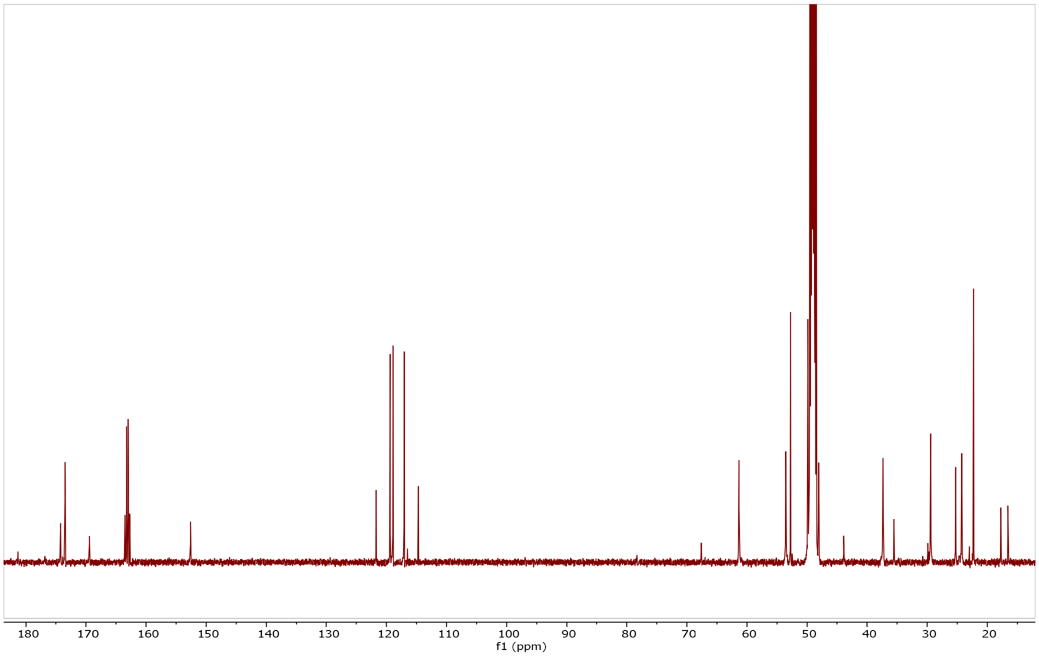

Figure S24. ^{13}C-NMR spectrum of *N*-acetyl-*Z*-L-fusarinine-methylester (**3**) in CD_3OD (125 MHz)

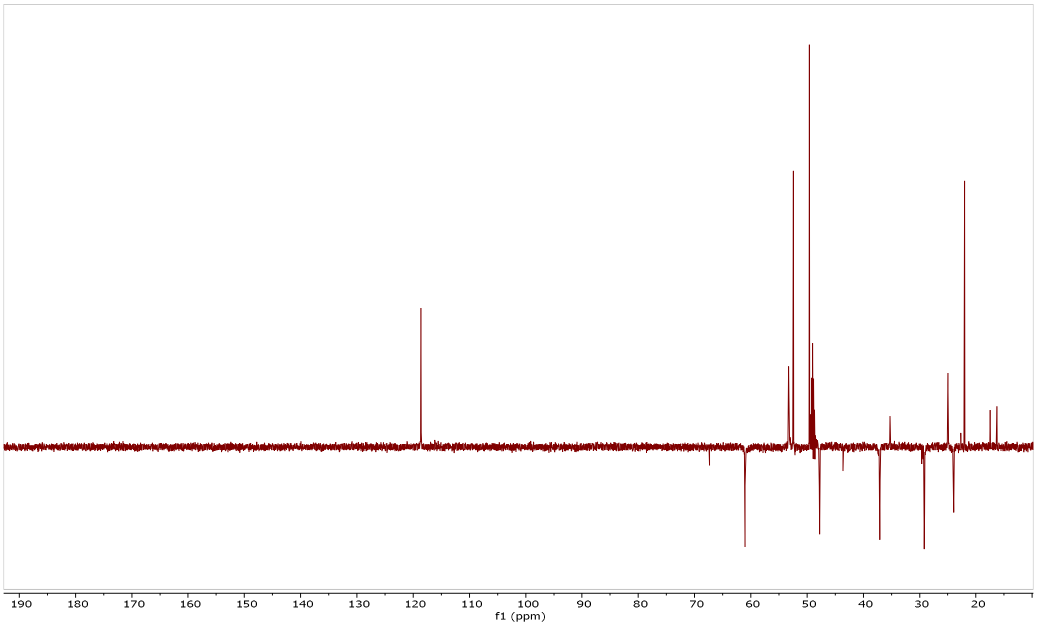

Figure S25. DEPT135-NMR spectrum of *N*-acetyl-*Z*-L-fusarinine-methylester (**3**) in CD_3OD (125 MHz)

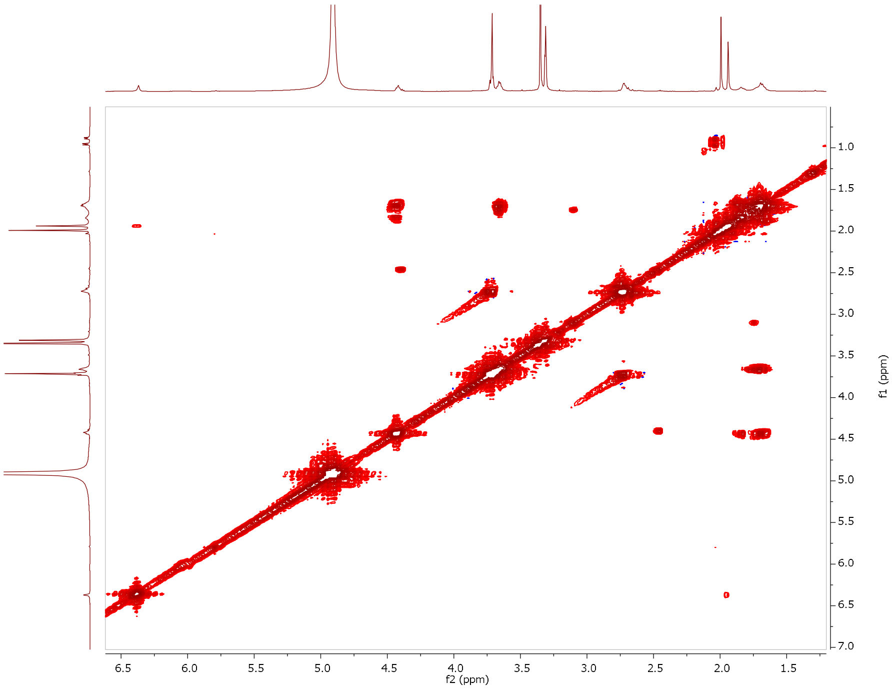

Figure S26. 2D-COSY-NMR spectrum of *N*-acetyl-*Z*-L-fusarinine-methylester (**3**) in CD_3OD

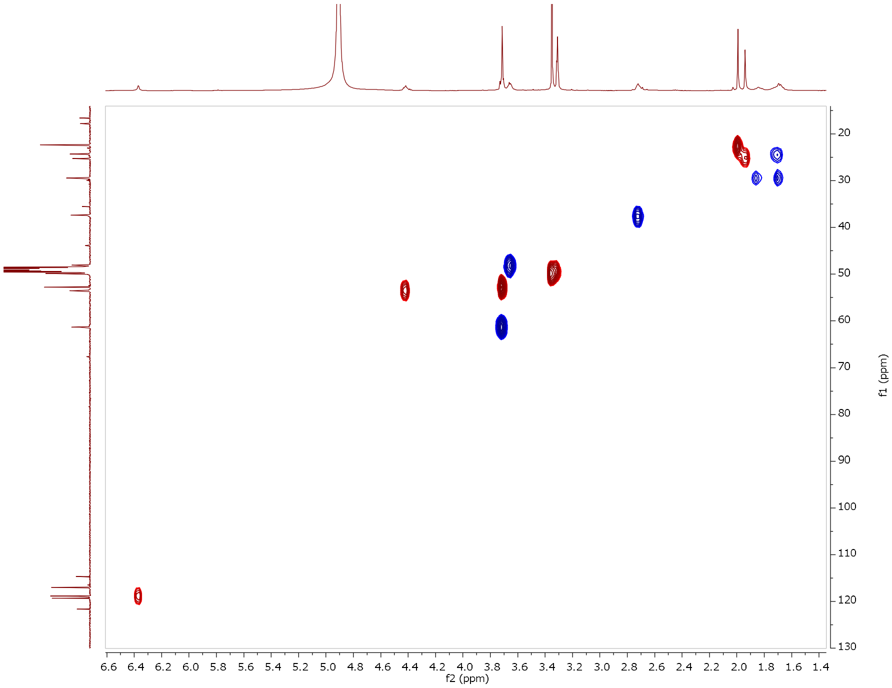

Figure S27. 2D-HSQC-NMR spectrum of *N*-acetyl-*Z*-L-fusarinine-methylester (**3**) in CD_3OD

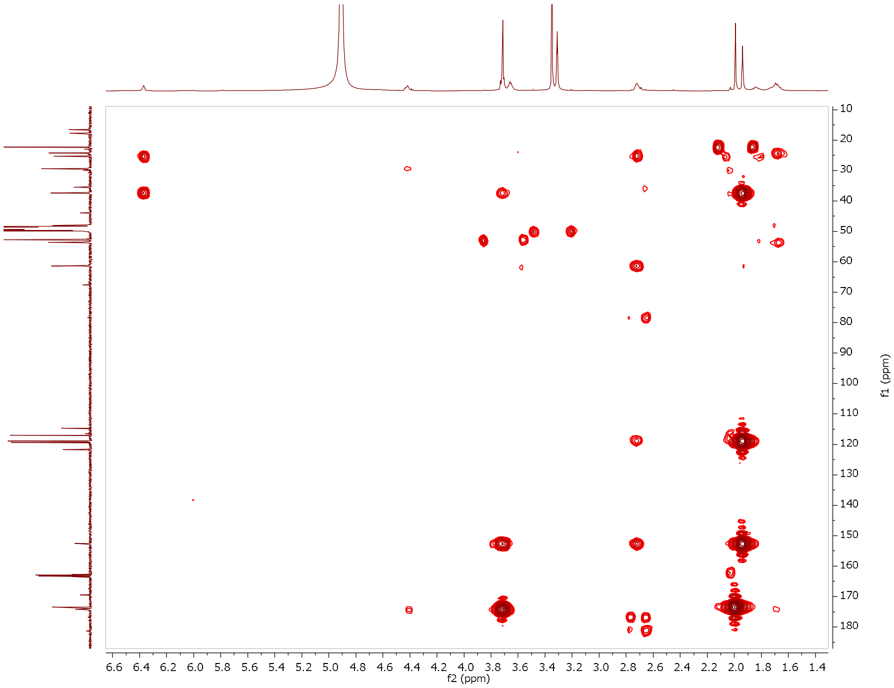

Figure S28. 2D-HMBC-NMR spectrum of *N*-acetyl-*Z*-L-fusarinine-methylester (**3**) in CD_3OD

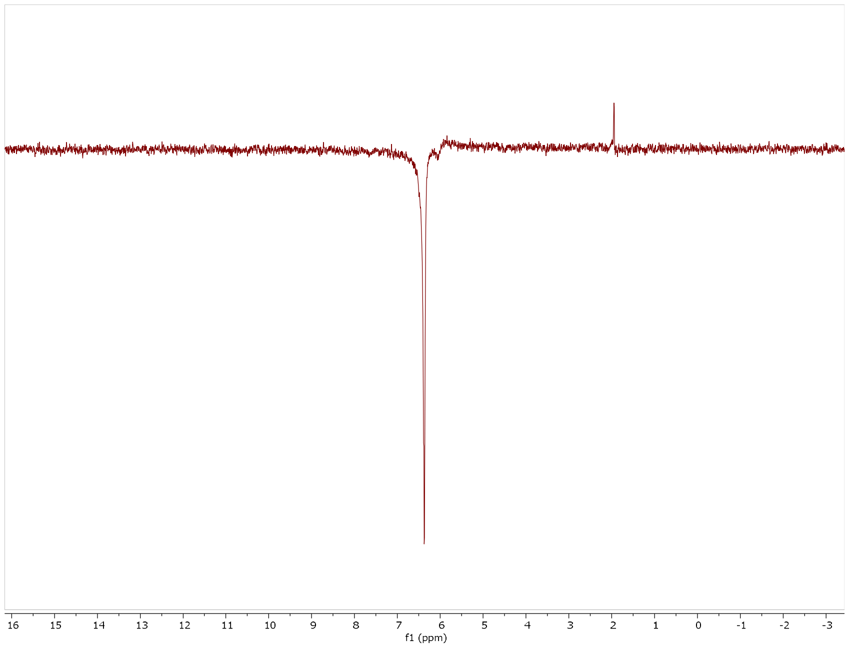

Figure S29. 1D-NOESY-NMR spectrum of *N*-acetyl-*Z*-L-fusarinine-methylester (**3**) in CD_3OD (300 MHz), obtained band selective excitation of the signal at 6.37 ppm

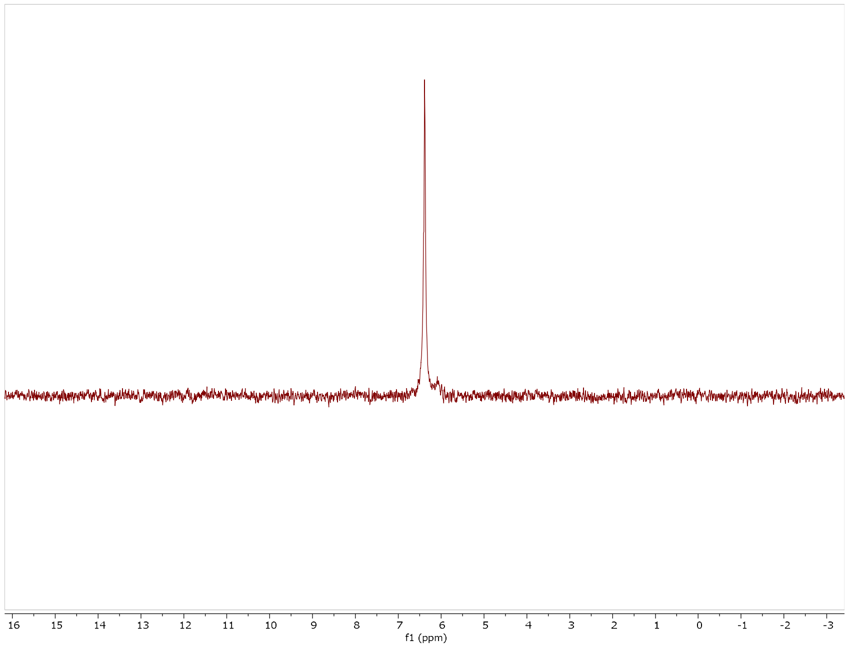

Figure S30. 1D-ROESY-NMR spectrum of *N*-acetyl-*Z*-L-fusarinine-methylester (**3**) in CD_3OD (300 MHz), obtained band selective excitation of the signal at 6.37 ppm

N,N',N''-Z-L-triacetyl-fusarinine C Ga^{3+} complex (4-Ga)

Table S8. Major *m/z* ions in the spectra (+) and (-) HRMS-ESI of *N,N',N''*-triacetyl-*Z*-L-fusarinine C Ga^{3+} complex (**4-Ga**)

Adduct	Observed *m/z*	Theoretical *m/z*	Error [ppm]
$[M\text{-}3H+^{69/71}Ga+Na]^+$	941.3059/943.3063	941.3037/943.3028	2.3/3.7
$[M\text{-}3H+^{69/71}Ga+K]^+$	957.2797/959.2799	957.2776/959.2767	2.2/3.3
$[M\text{-}3H+^{69/71}Ga\text{-}H]^-$	917.3063/919.3059	917.3065/919.3056	0.2/0.3
$[M\text{-}3H+^{69/71}Ga+Cl]^-$	953.2838/955.2817	953.2826/955.2817	1.3/0
$[M\text{-}3H+^{69/71}Ga+FA\text{-}H]^-$	963.3118/965.3115	963.3120/965.3111	0.2/0.4

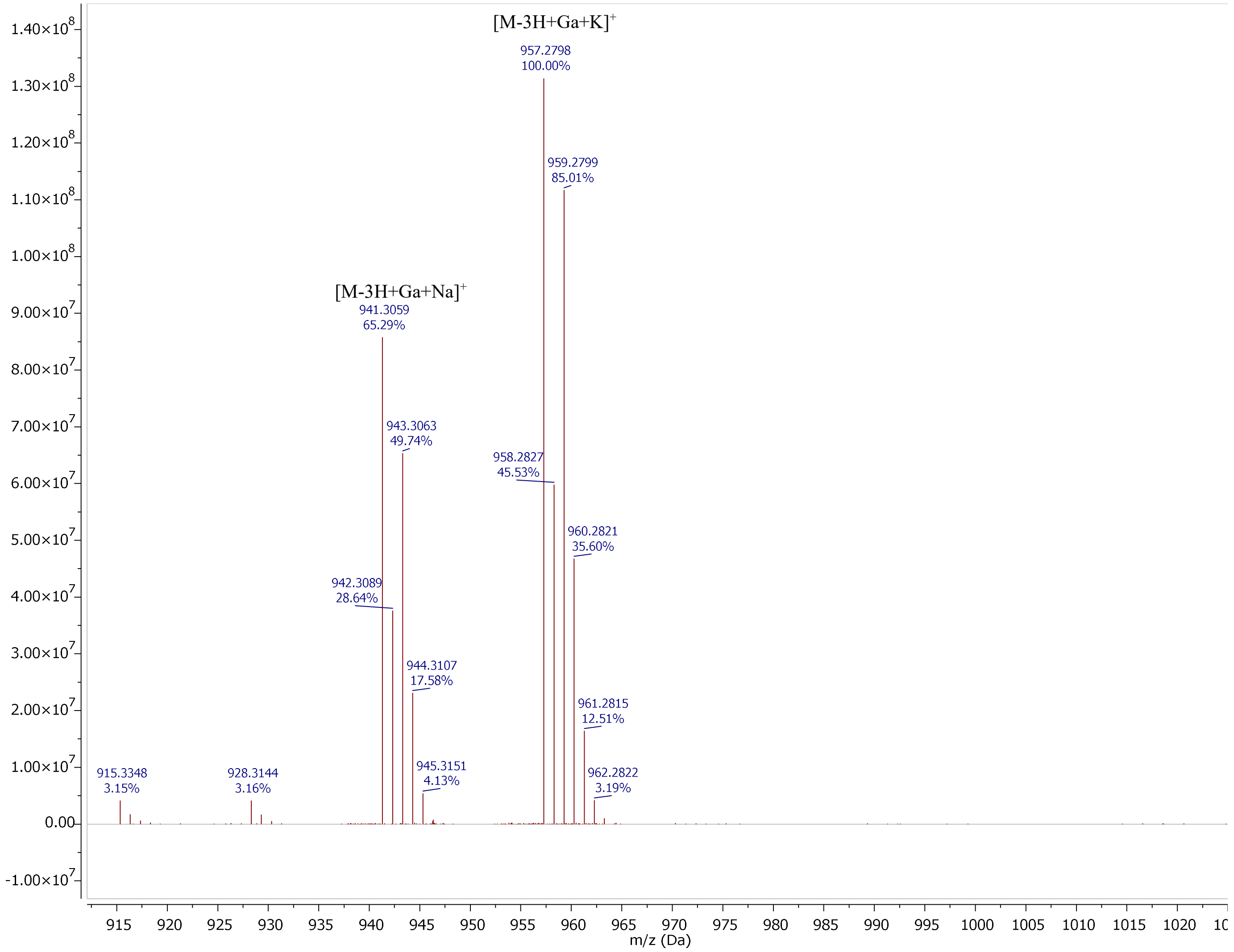

Figure S31. (+) HRMS-ESI of *N,N',N''*-triacetyl-*Z*-L-fusarinine C Ga^{3+} complex (**4-Ga**)

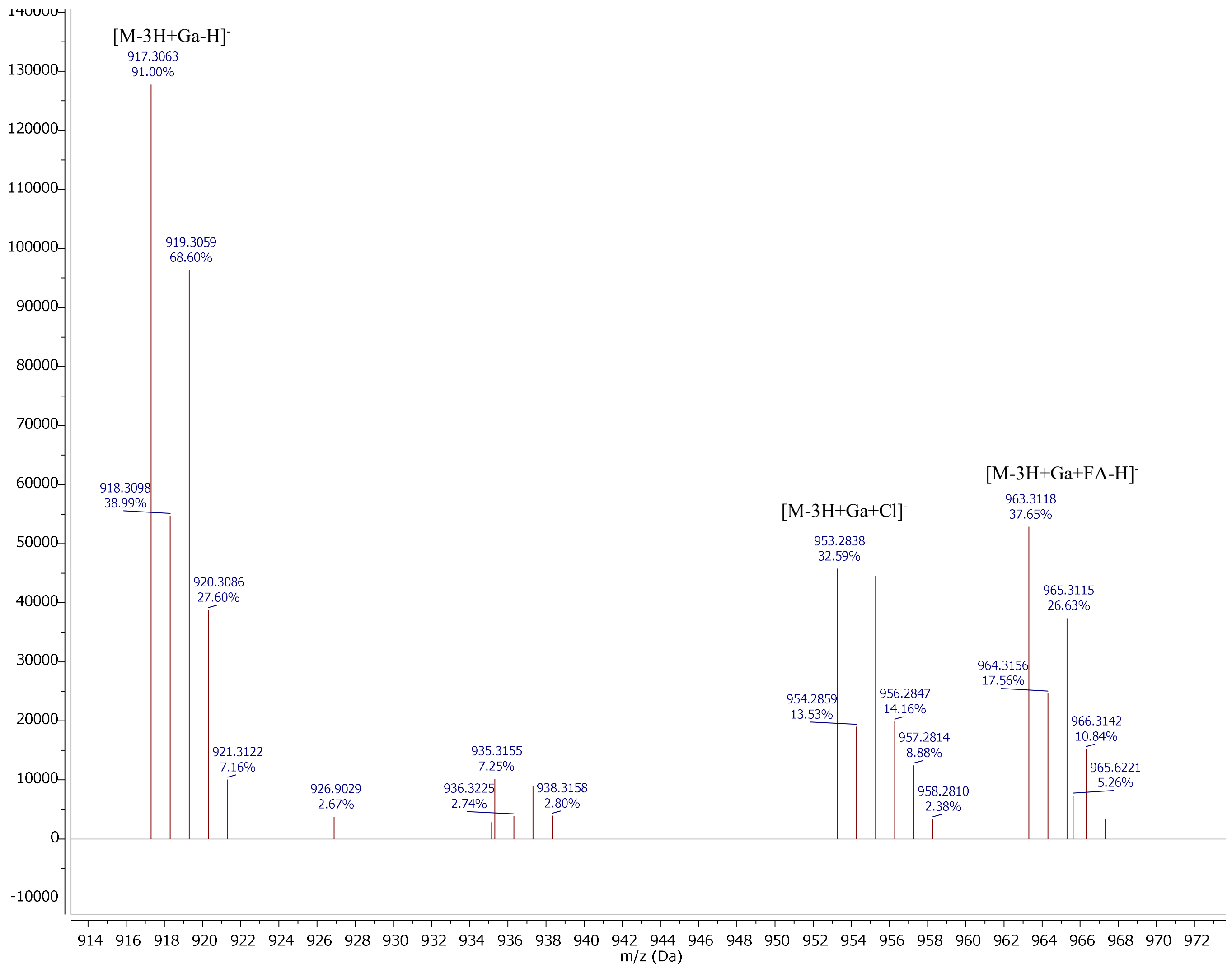

Figure S32. (-) HRMS-ESI of *N,N',N''*-triacetyl-*Z*-L-fusarinine C Ga^{3+} complex (**4-Ga**)

Table S9. NMR chemical shifts of *N,N',N''*-triacetyl-*Z*-L-fusarinine C Ga^{3+} complex (**4-Ga**) in CD_3OD (500 MHz)

Position	δ_C ᵃ mult	δ_H ᵇ mult (*J* in Hz)	∫	HMBC (H→C#)	COSY (H→H#)
**2 2' 2''**	52.01, CH	4.34, dd (9.6, 3.0)	3	14 14' 14''	4 4' 4'', 5 5' 5''
**3 3' 3''**	173.58, C				
**4 4' 4''**	28.12, CH_2	1.53 - 1.48, m<br>2.07 - 2.00, m	3<br>3		2 2' 2'', 4 4' 4'',<br>5 5' 5''
**5 5' 5''**	23.26, CH_2	1.53 - 1.48, m<br>2.07 - 2.00, m	3<br>3		2 2' 2'', 4 4' 4''<br>5 5' 5'', 6 6' 6''
**6 6' 6''**	49.80, CH_2	3.54 tt (13.6, 3.7)<br>4.09, tt (10.1, 3.5)	3<br>3		5 5' 5'', 6 6' 6''
**8 8' 8''**	161.57, C				
**9 9' 9''**	116.03, CH	6.11, s	3	11 11' 11'', 16 16' 16''	16 16' 16''
**10 10' 10''**	151.42, C				
**11 11' 11''**	34.23, CH_2	2.20, m<br>3.54, tt (13.6, 3.7)	3<br>3		11 11' 11''<br>12 12' 12''
**12 12' 12''**	62.65, CH_2	3.99, ddd (10.9, 6.9, 3.6)<br>4.72, tt (8.3, 2.6)	3<br>3		11 11' 11''<br>12 12' 12''
**14 14' 14''**	172.55, C				
**15 15' 15''**	22.26, CH_3	1.98, s	9	14 14' 14''	
**16 16' 16''**	23.55, CH_3	1.97, d (1.4)	9	9 9' 9'', 10 10' 10'',<br>11 11' 11''	9 9' 9''

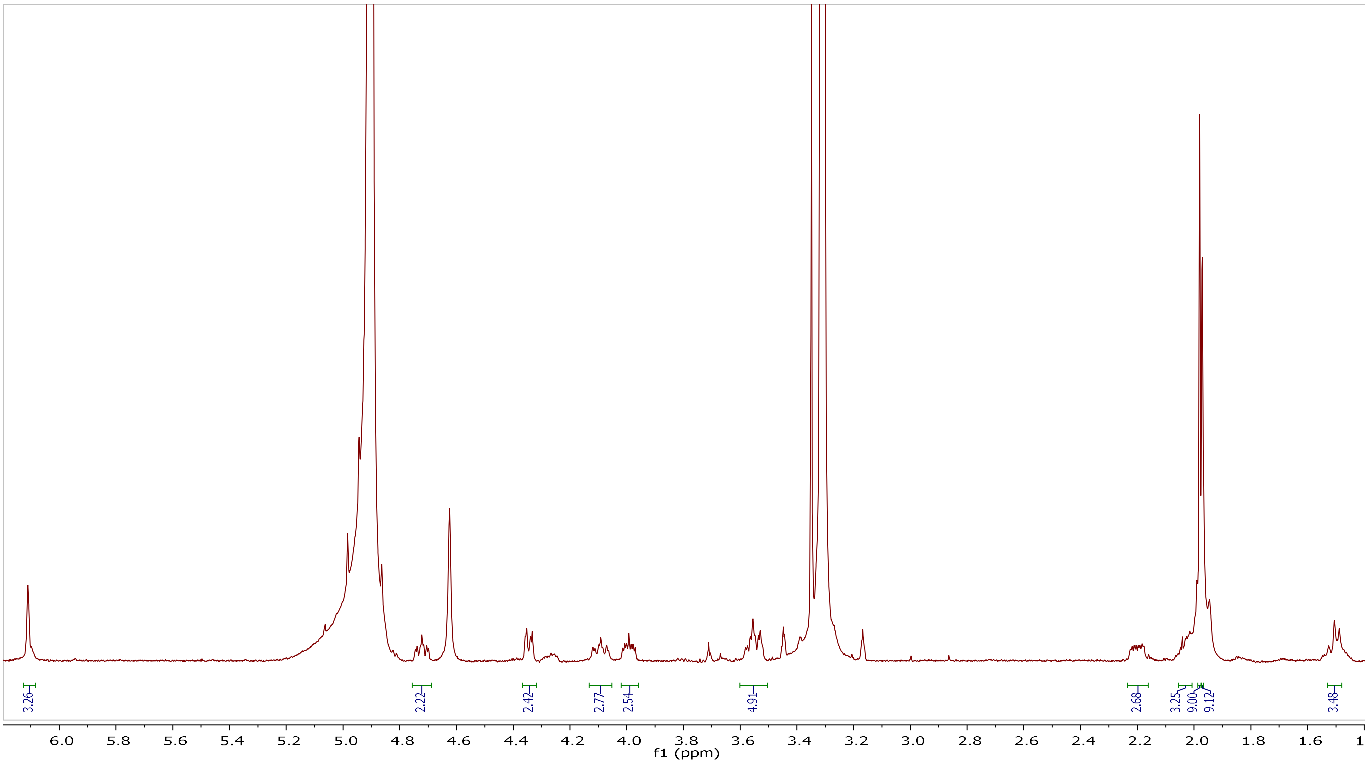

Figure S33. ^{1}H-NMR spectrum of *N,N',N''*-triacetyl-*Z*-L-fusarinine C Ga^{3+} complex (**4-Ga**) in CD_3OD (500 MHz)

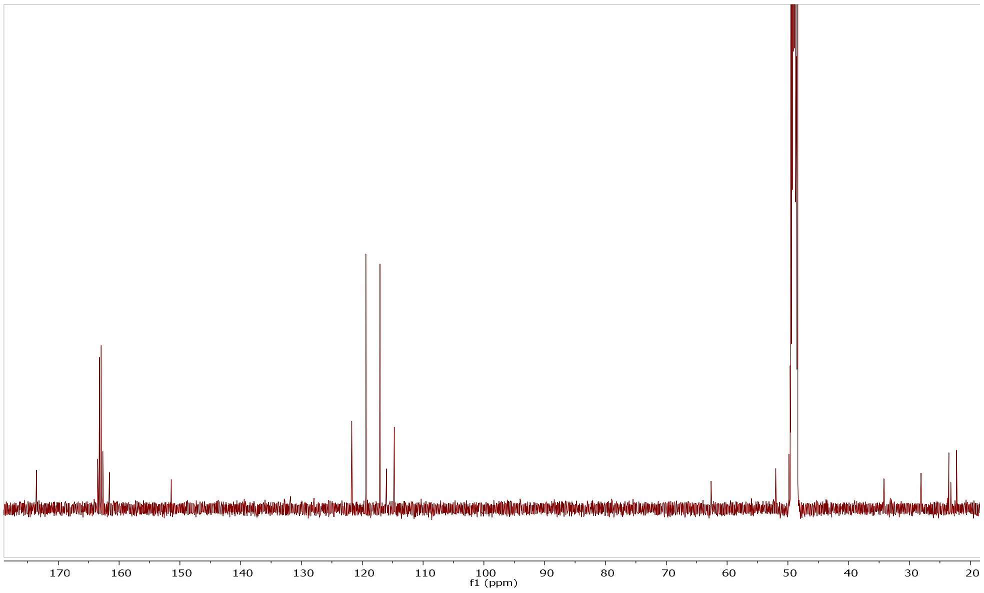

Figure S34. ^{13}C-NMR spectrum *N,N',N''*-triacetyl-*Z*-L-fusarinine C Ga^{3+} complex (**4-Ga**) in CD_3OD (125 MHz)

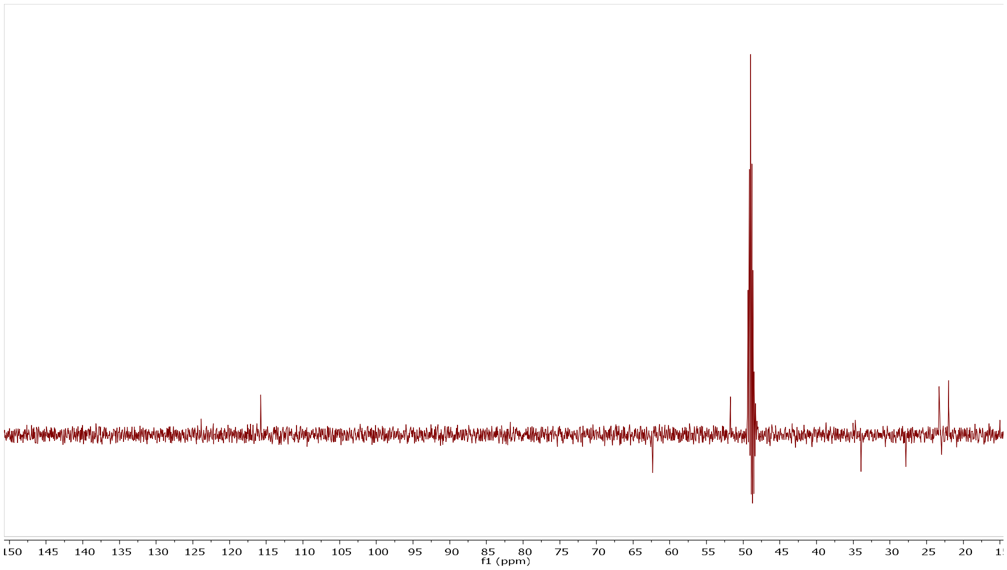

Figure S35. DEPT135-NMR spectrum of *N,N',N''*-triacetyl-*Z*-L-fusarinine C Ga^{3+} complex (**4-Ga**) in CD_3OD (125 MHz)

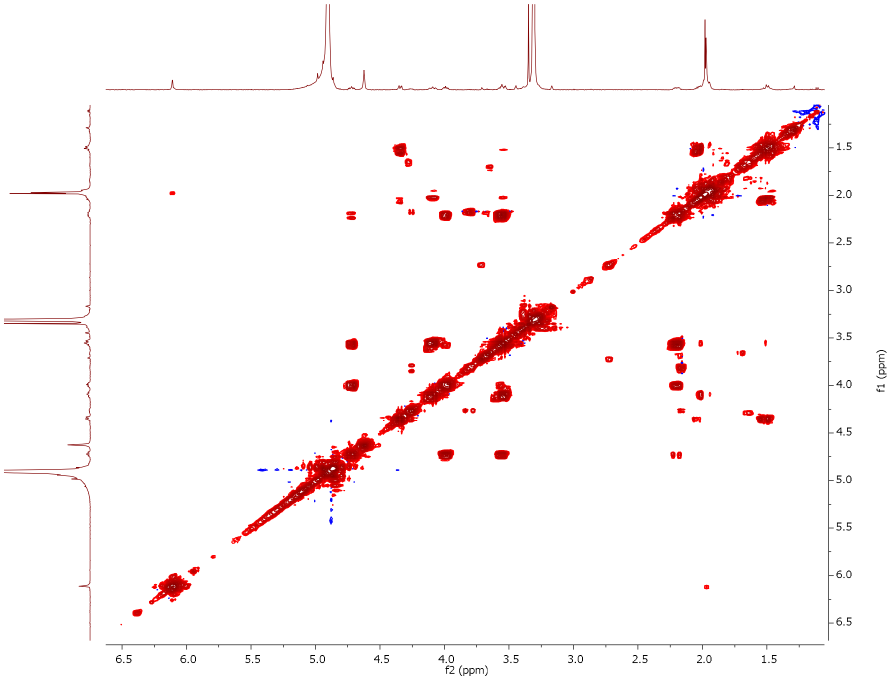

Figure S36. 2D-COSY-NMR spectrum of *N,N',N''*-triacetyl-*Z*-L-fusarinine C Ga^{3+} complex (**4-Ga**) in CD_3OD

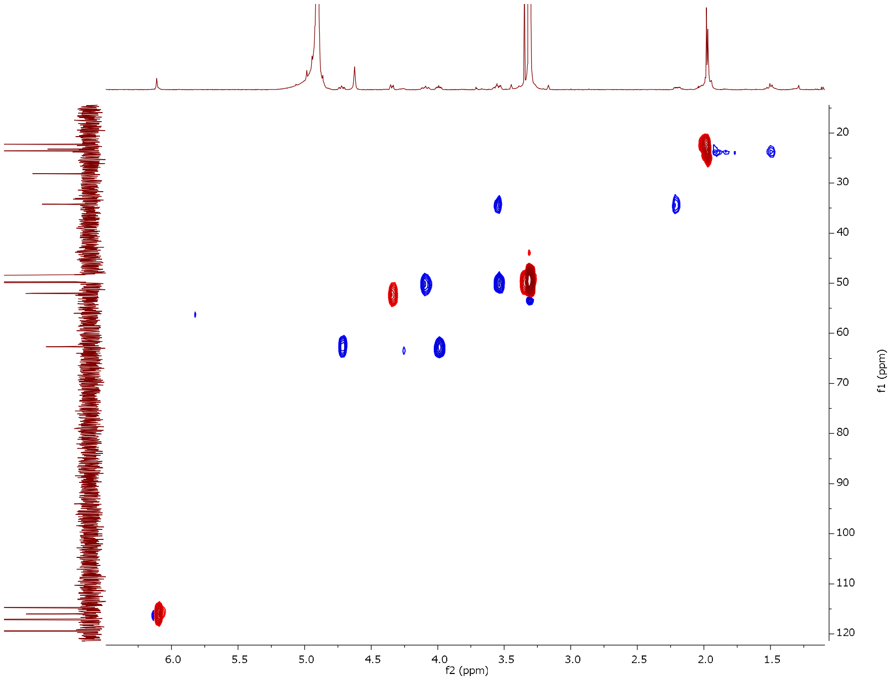

Figure S37. 2D-HSQC-NMR spectrum of *N,N',N''*-triacetyl-*Z*-L-fusarinine C Ga^{3+} complex (**4-Ga**) in CD_3OD

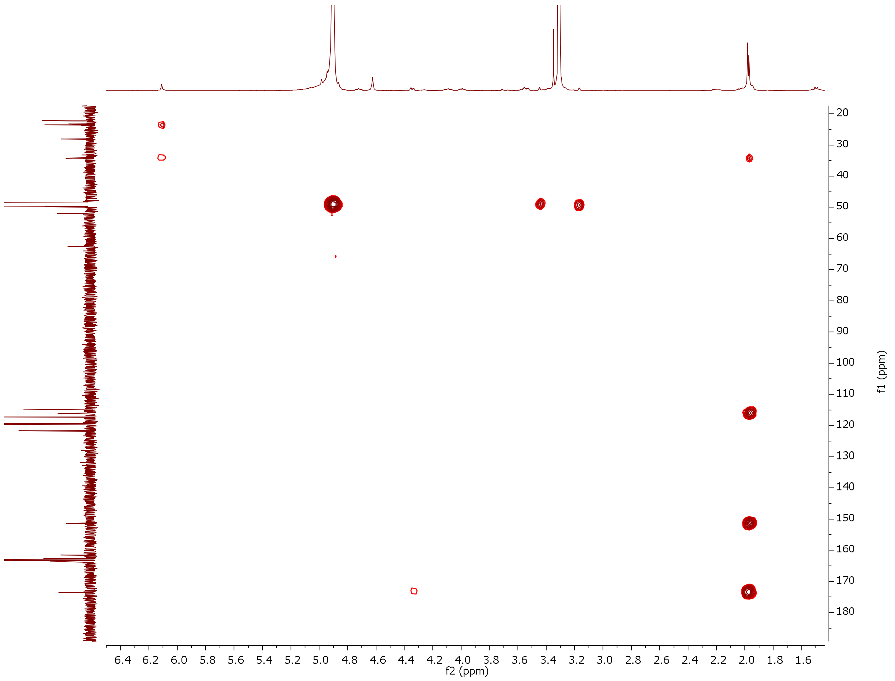

Figure S38. 2D-HMBC-NMR spectrum of *N,N',N''*-triacetyl-*Z*-L-fusarinine C Ga^{3+} complex (**4-Ga**) in CD_3OD

N,N',N''-triacetyl-*Z*-L-fusarinine B Ga^{3+} complex (5-Ga)

Table S10. Major *m/z* ions in the (+) and (-) HRMS-ESI of *N,N',N''*-triacetyl-*Z*-L-fusarinine B Ga^{3+} complex (**5-Ga**)

Adduct	Observed *m/z*	Theoretical *m/z*	Error [ppm]
$[M\text{-}3H+^{69/71}Ga+Na]^+$	959.3139/961.3139	959.3142/961.3133	0.3/0.6
$[MCOONa+^{69/71}Ga+Na]^+$	981.2955/983.2955	981.2962/983.2953	0.7/0.2
$[M\text{-}3H+^{69/71}Ga\text{-}H]^-$	935.3178/937.3179	935.3171/937.3162	0.7/1.8

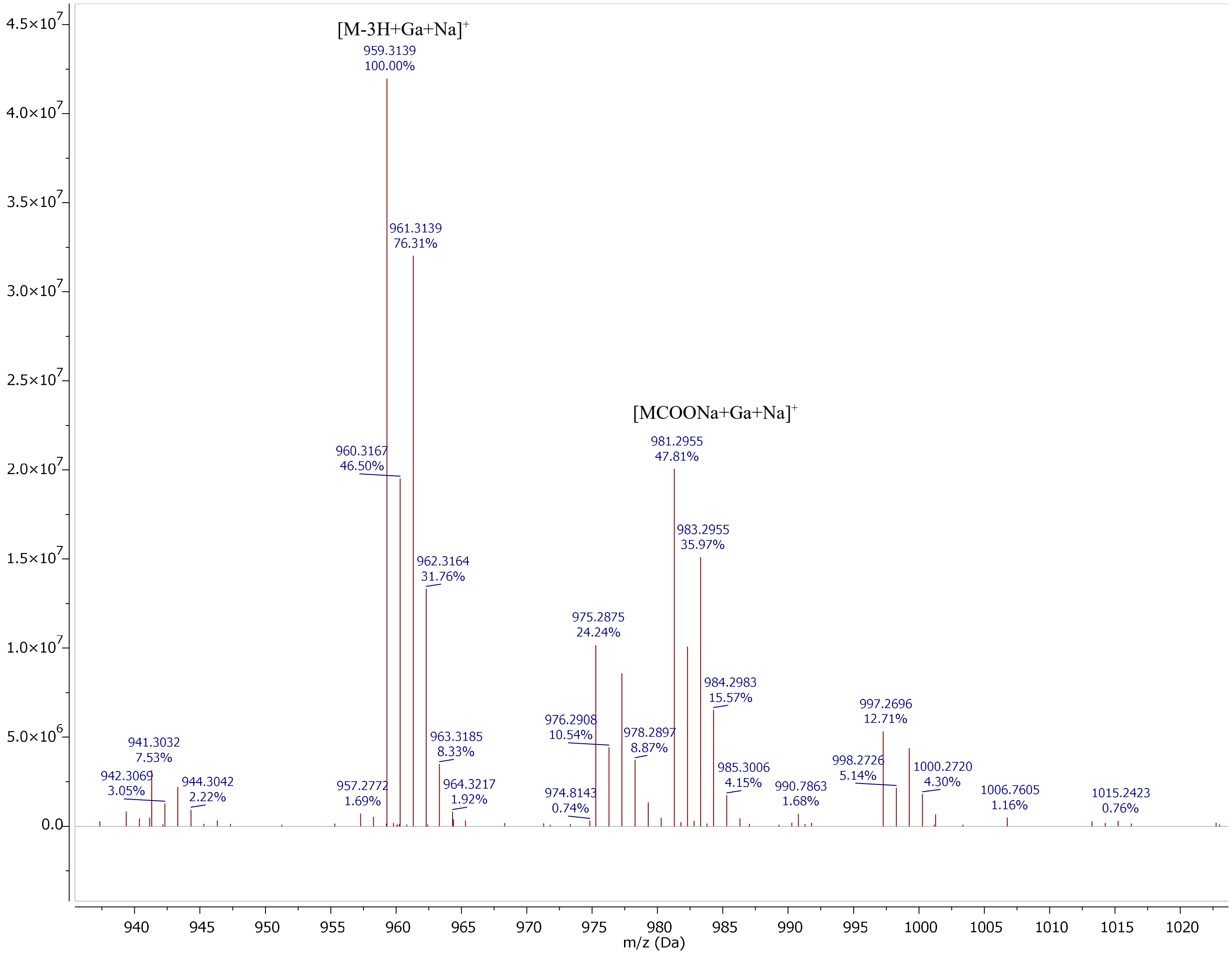

Figure S39. (+) HRMS-ESI of *N,N',N''*-triacetyl-*Z*-L-fusarinine B Ga^{3+} complex (**5-Ga**)

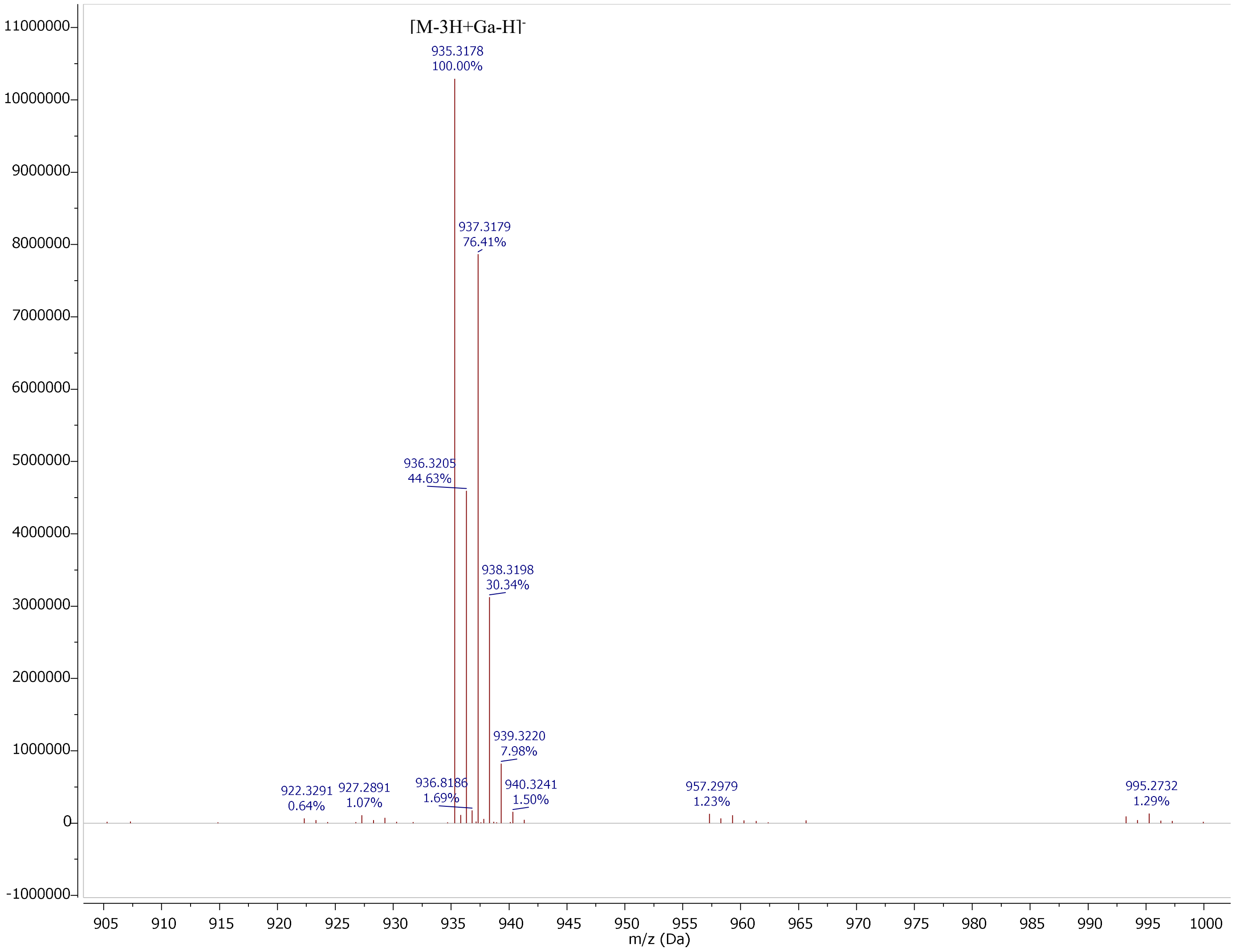

Figure S40. (-) HRMS-ESI of *N,N',N''*-triacetyl-*Z*-L-fusarinine B Ga^{3+} complex (**5-Ga**)

Table S11. NMR chemical shifts of *N,N',N''*-triacetyl-*Z*-L-fusarinine B Ga^{3+} complex (**5-Ga**) in CD_3OD (500 MHz)

Position	δ_C [a] mult	δ_H [b] mult (*J* in Hz)	∫	HMBC (H→C#)	COSY (H→H#)
**2 2' 2''**	54.28, CH	4.33 - 4.27, m	3		4 4' 4'', 5 5' 5''
**3**	174.17, C				
**3' 3''**	174.07, C<br>173.97, C				
**4 4' 4''**	29.67, CH_2	1.79 - 1.87, m	6		2 2' 2'', 5 5' 5''
**5 5' 5''**	25.48, CH_2	1.55 - 1.79, m	6		2 2' 2'', 4 4' 4'', 6 6' 6''
**6 6' 6''**	51.82, CH_2	3.69 - 3.63, m	6		5 5' 5''
**8 8' 8''**	162.66, C				
**9 9' 9''**	114.75, CH	6.08 - 6.11, m<br>6.16 - 6.19, m	2<br>1	11 11' 11'', 16 16' 16''	16 16' 16''
**10 10' 10''**	153.99, C				
**11 11'**	34.08, CH_2	2.06 - 2.13, m<br>2.84 - 2.57, m	3<br>1		11 11', 12 12'
**11''**	38.17, CH_2	2.84 - 2.57, m	2		12''
**12 12'**	63.71, CH_2	4.11 - 4.07, m<br>4.33 - 4.27, m	2<br>2		11 11'
**12''**	62.12, CH_2	3.79 - 3.74, m	2		11''
**14 14' 14''**	173.82, C				
**15 15' 15''**	22.29, CH_3<br>22.68, CH_3<br>22.77, CH_3	1.96, s	9	14 14' 14''	
**16 16' 16''**	23.13, CH_3	1.99, d (1.5)	9	9 9' 9'', 10 10'10'', 11 11' 11''	9 9' 9''

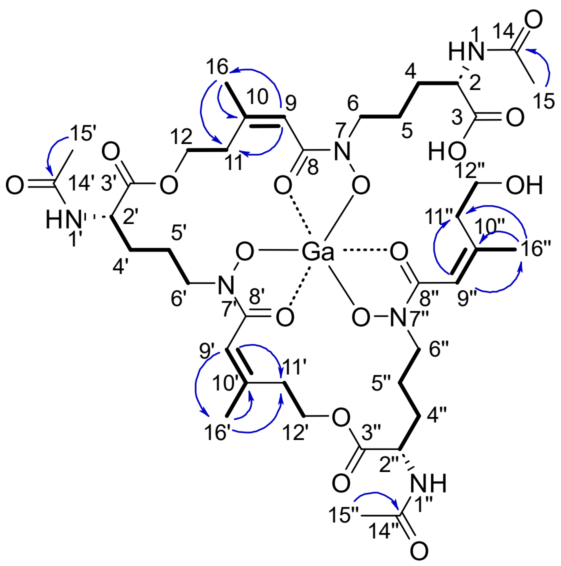

Figure S41. ^{1}H-^{1}H COSY (▬) and ^{1}H-^{13}C HMBC(→) correlations of *N,N',N''*-triacetyl-*Z*-L-fusarinine B Ga^{3+} complex (**5-Ga**) in CD_3OD

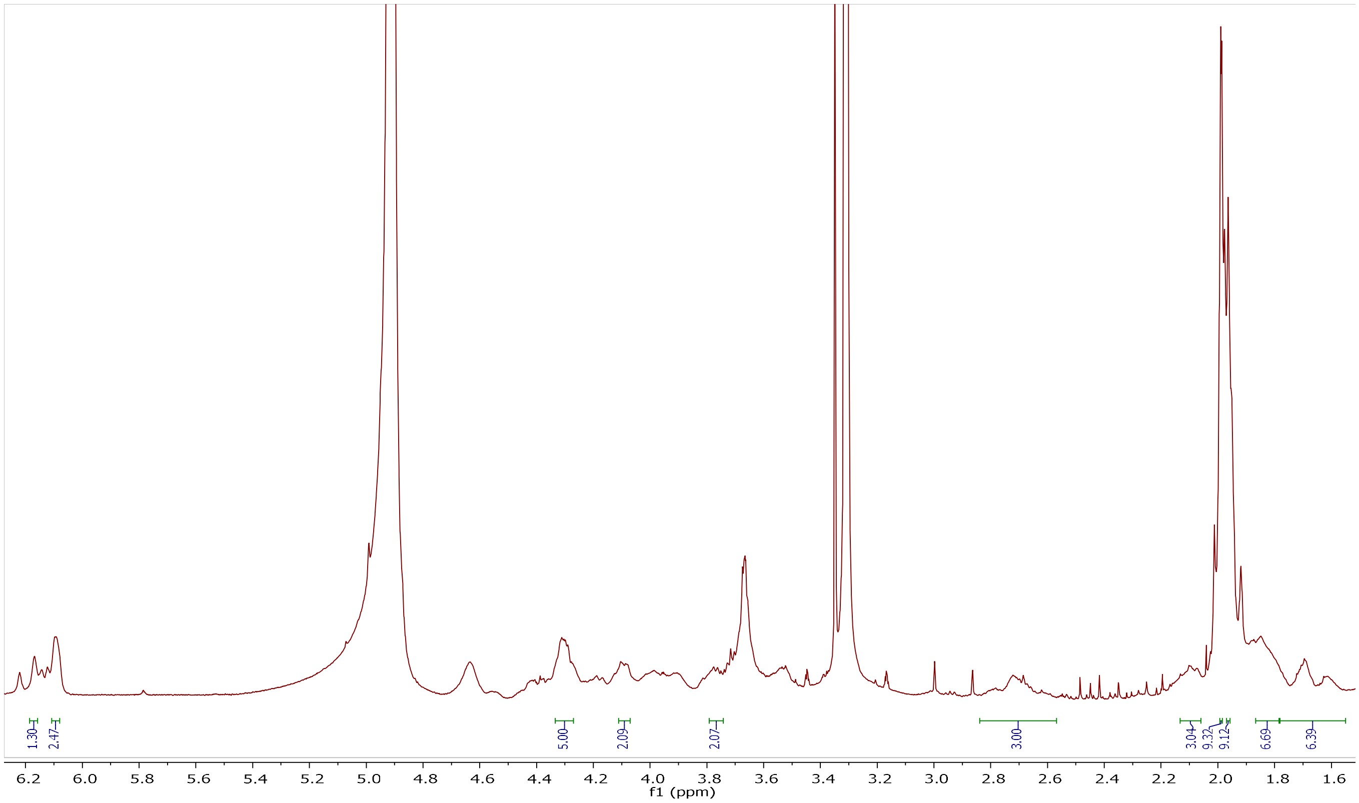

Figure S42. ^{1}H-NMR spectrum of *N,N',N''*-triacetyl-*Z*-L-fusarinine B Ga^{3+} complex (**5-Ga**) in CD_3OD (500 MHz)

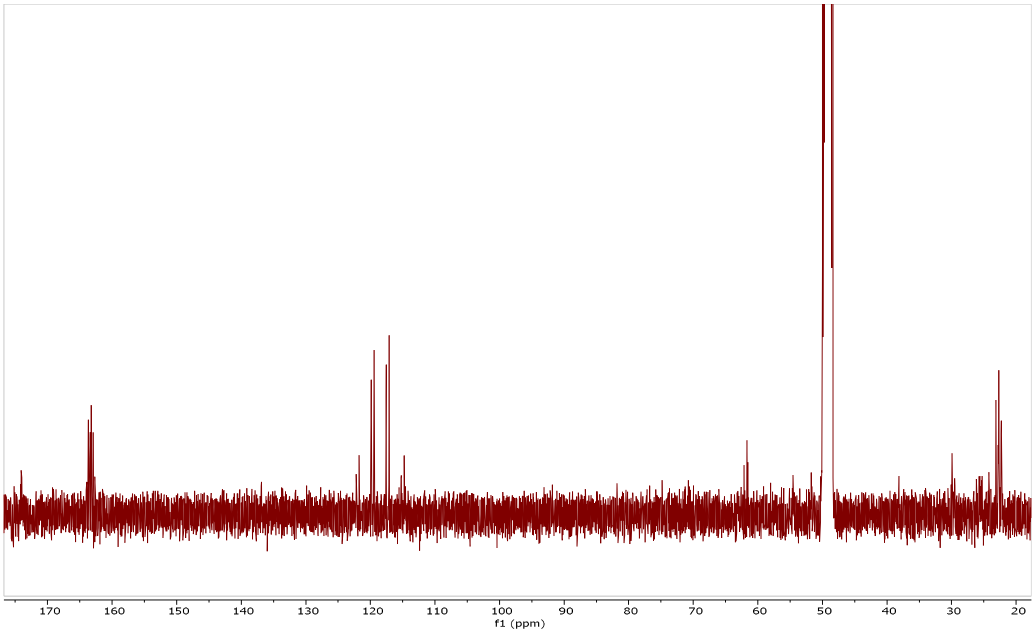

Figure S43. ^{13}C-NMR spectrum of *N,N',N''*-triacetyl-*Z*-L-fusarinine B Ga^{3+} complex (**5-Ga**) in CD_3OD (125 MHz)

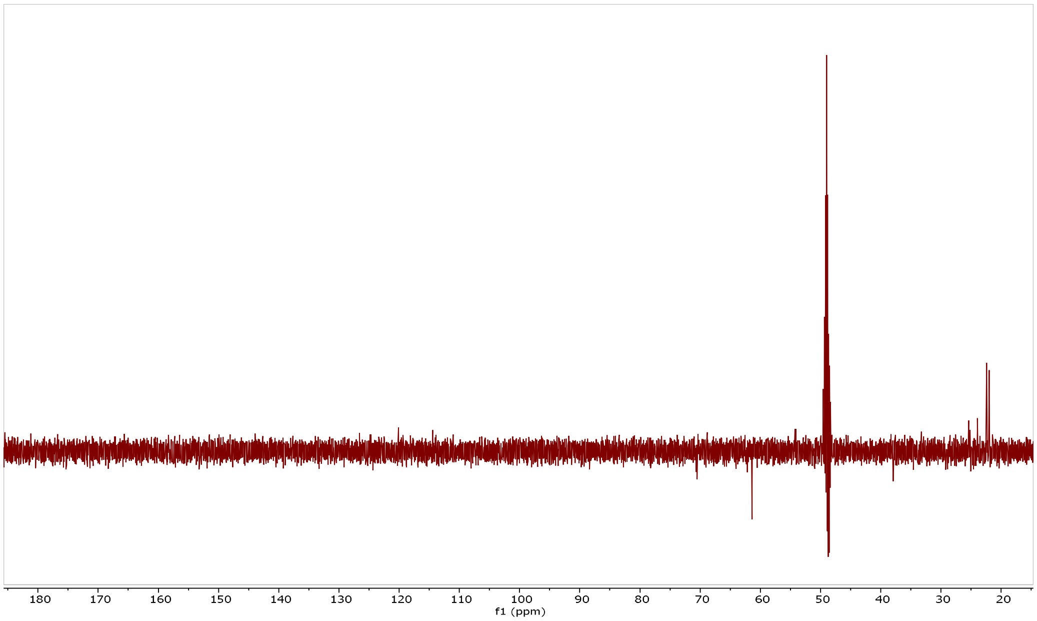

Figure S44. DEPT135-NMR spectrum of *N,N',N''*-triacetyl-*Z*-L-fusarinine B Ga^{3+} complex (**5-Ga**) in CD_3OD (125 MHz)

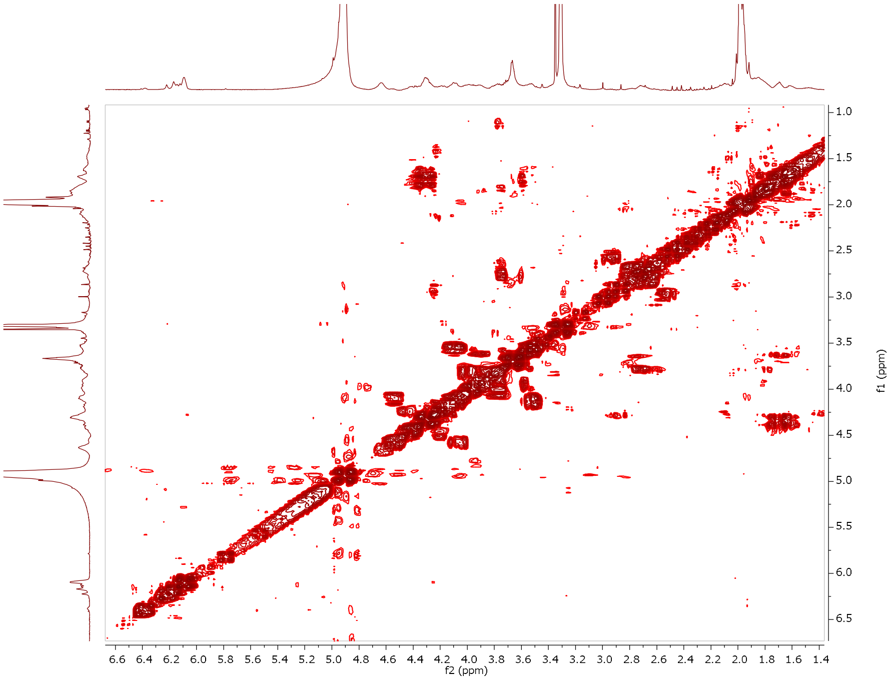

Figure S45. 2D-COSY-NMR spectrum of *N,N',N''*-triacetyl-*Z*-L-fusarinine B Ga^{3+} complex (**5-Ga**) in CD_3OD

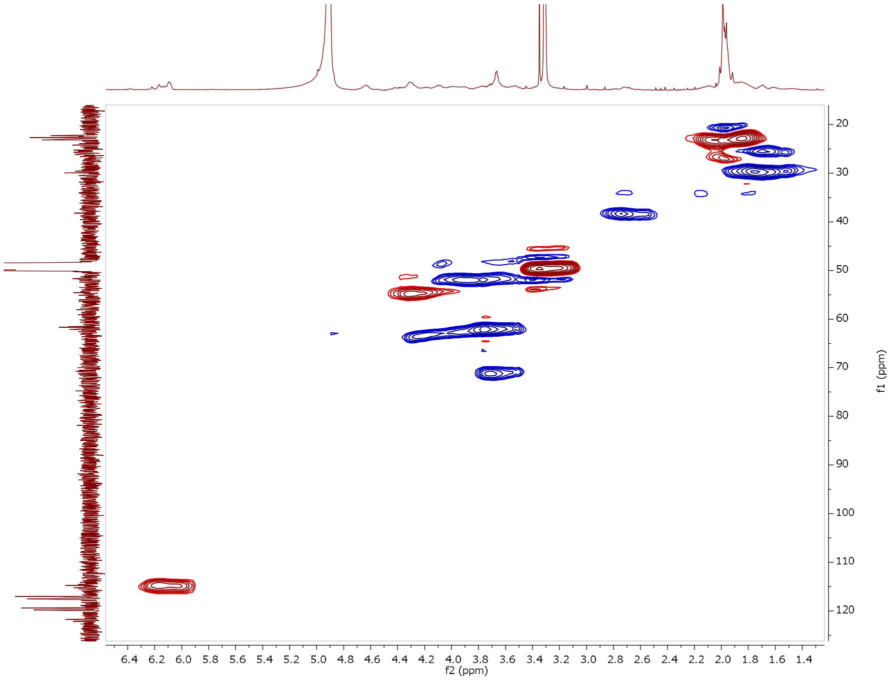

Figure S46. 2D-HSQC-NMR spectrum of *N,N',N''*-triacetyl-*Z*-L-fusarinine B Ga^{3+} complex (**5-Ga**) in CD_3OD

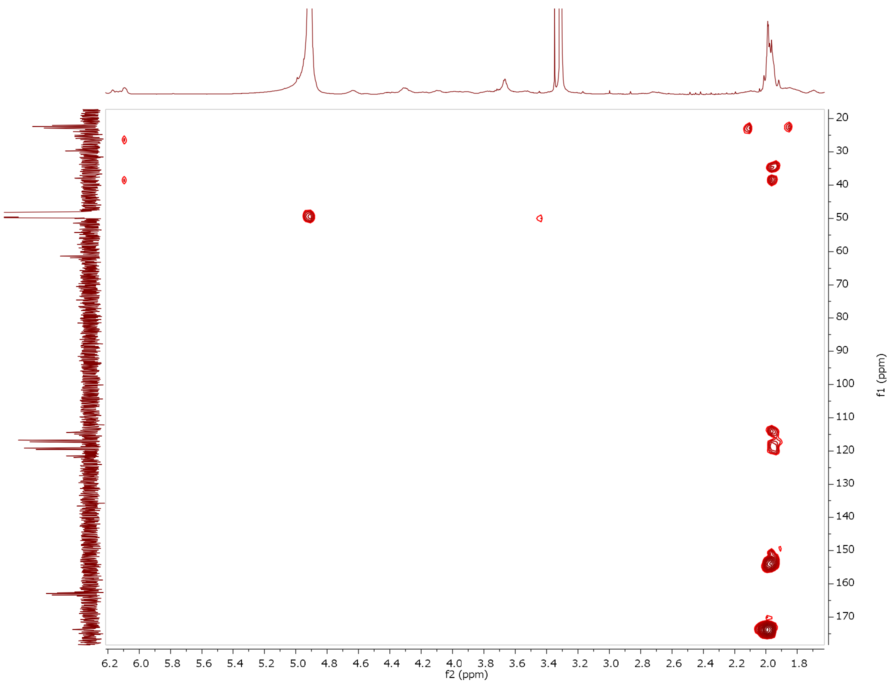

Figure S47. 2D-HMBC-NMR spectrum of *N,N',N''*-triacetyl-*Z*-L-fusarinine B Ga^{3+} complex (**5-Ga**) in CD_3OD

N,N’,N’’-triacetyl-*Z*-L-fusarinine B (5)

Table S12. Major *m/z* ions in (+) and (-) HRMS-ESI of *N,N’,N’’*-triacetyl-*Z*-L-fusarinine B (**5**)

Adduct	Observed *m/z*	Theoretical *m/z*	Error [ppm]
$[M+Na]^+$	893.4132	893.4120	1.3
$[MCOONa+Na]^+$	915.3950	915.3940	1.1
$[M-H]^-$	869.4156	869.4144	1.4

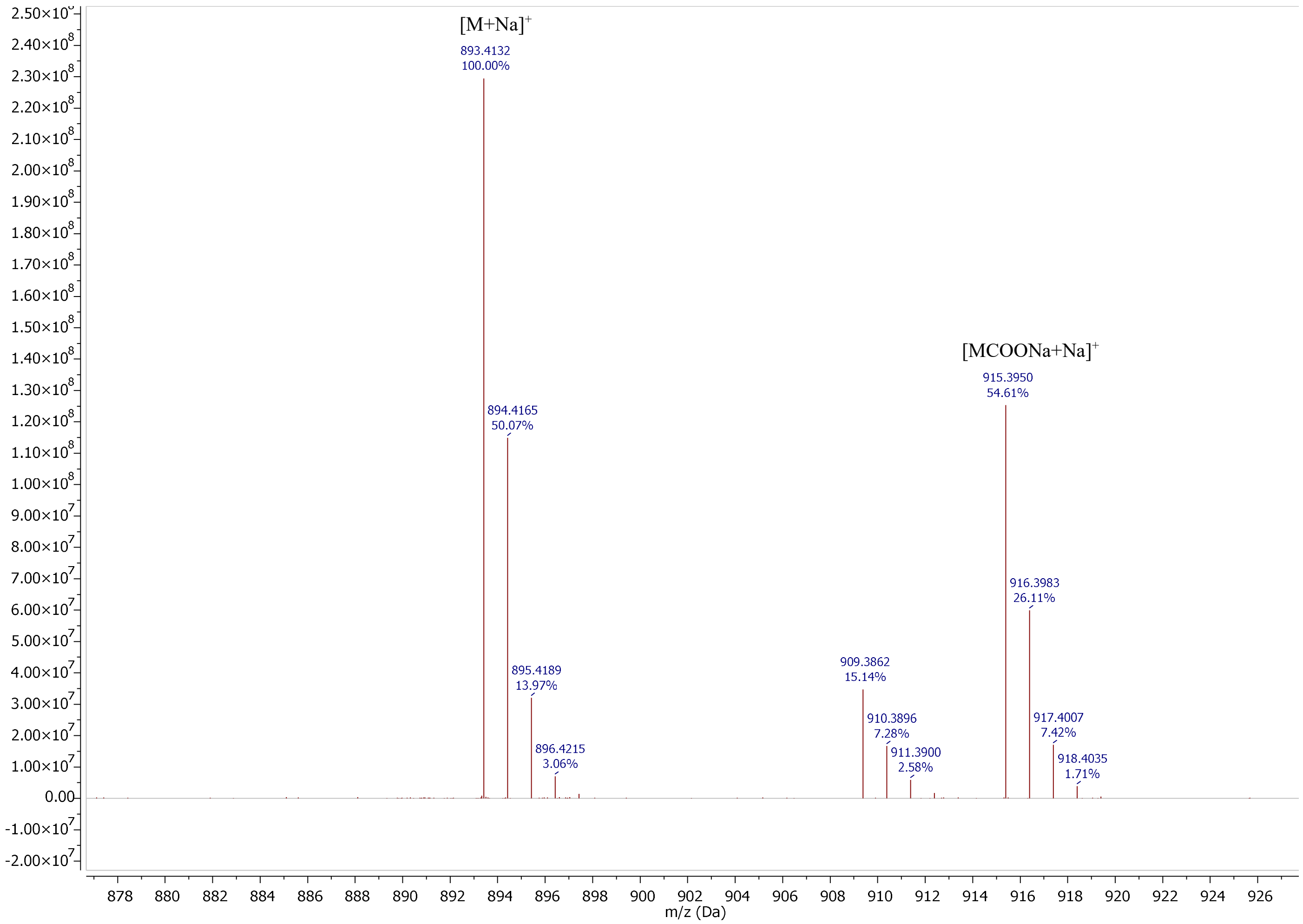

Figure S48. (+) HRMS-ESI of *N,N’,N’’*-triacetyl-*Z*-L-fusarinine B (**5**)

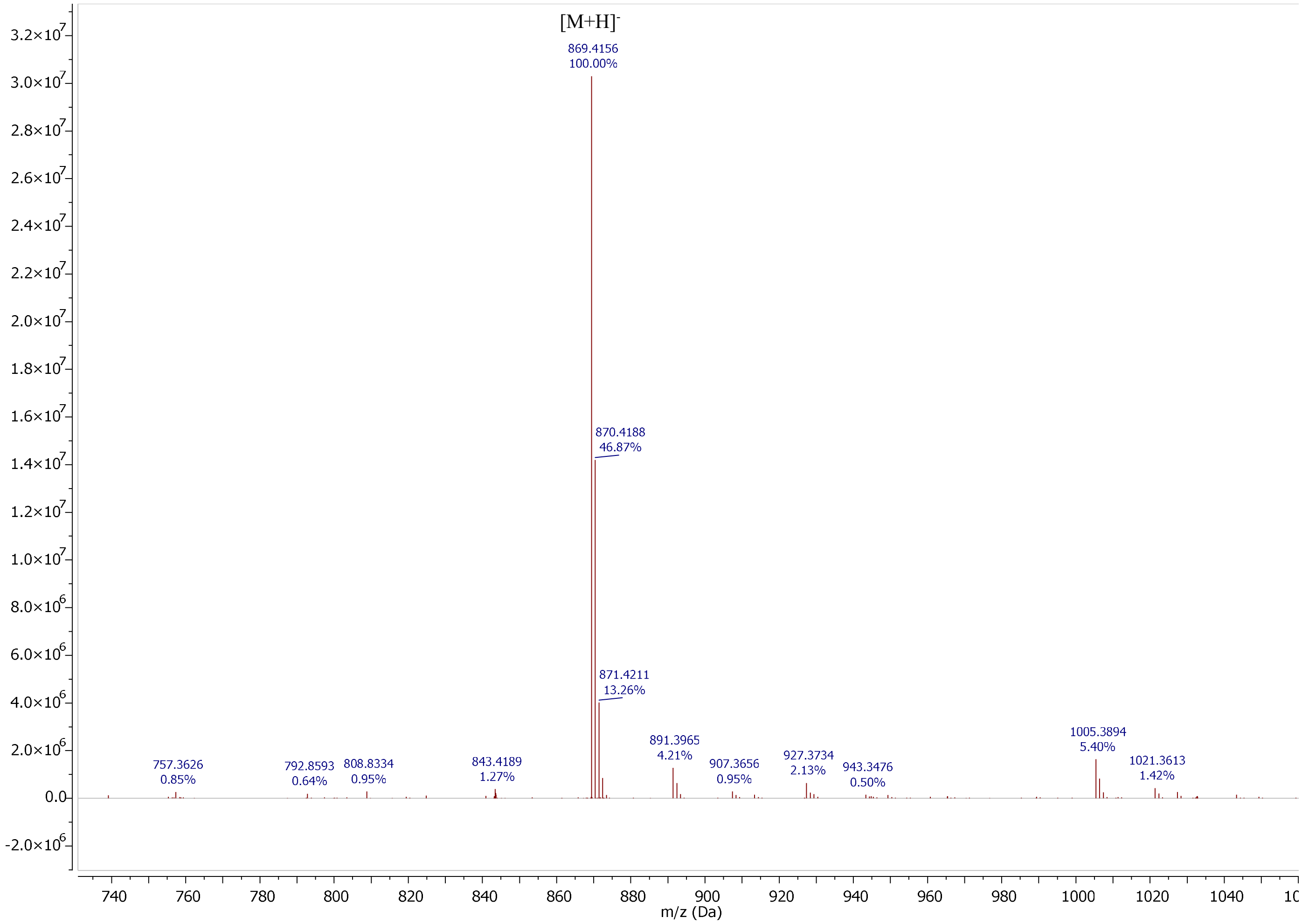

Figure S49. (-) HRMS-ESI of *N,N',N''*-triacetyl-*Z*-L-fusarinine B (**5**)

Table S13. NMR chemical shifts of *N,N',N''*-triacetyl-*Z*-L-fusarinine B (**5**) in CD_3OD (400 MHz)

Position	δ_C [a] mult	δ_H [b] mult (*J* in Hz)	∫	HMBC (H→C#)	COSY (H→H#)
**2**	55.48, CH	4.28 - 4.22, m	1	3, 4, 5	4, 5
**2' 2''**	53.78, CH	4.38 - 4.33, m	2	4' 4'', 5' 5''	4' 4'', 5' 5''
**3**	178.16, C				
**3' 3''**	173.57, C				
**4**	30.77, CH_2	1.71 - 1.58, m	2		2, 5
**4' 4''**	29.55, CH_2	1.71 - 1.58, m<br>1.85 - 1.75, m	1<br>3		2 2', 5' 5''
**5 5' 5''**	24.43, CH_2	1.71 - 1.58, m	6		6 6' 6''
**6 6' 6''**	48.42, CH_2	3.64 - 3.57, m	6	4 4' 4'', 5 5'5'',<br>8 8'8''	5 5' 5''
**8 8' 8''**	168.72, C<br>169.43, C				
**9 9' 9''**	118.94	6.34, s	3		16 16' 16''
**10 10'**	151.49, C				
**10''**	152.58, C				
**11 11'**	33.48, CH_2	2.95 - 2.76, m	4		12 12'
**11''**	37.49, CH_2	2.68, t (6.3)	2		12''
**12 12'**	64.91, CH_2	4.28 - 4.22, m	4	10 10', 11 11'	11 11'
**12''**	61.38, CH_2	3.68, t (6.5)	2	10'', 11''	11''
**14 14' 14''**	173.43, C				
**15**	22.36, CH_3	1.947, s	3	14	
**15' 15''**	22.75, CH_3	1.951, s	6	14' 14''	
**16 16'**	25.71, CH_3	1.91, m	6	9 9', 10 10',<br>11 11'	9 9'
**16''**	25.28, CH_3	1.90, m	3	9'', 10'', 11''	9''

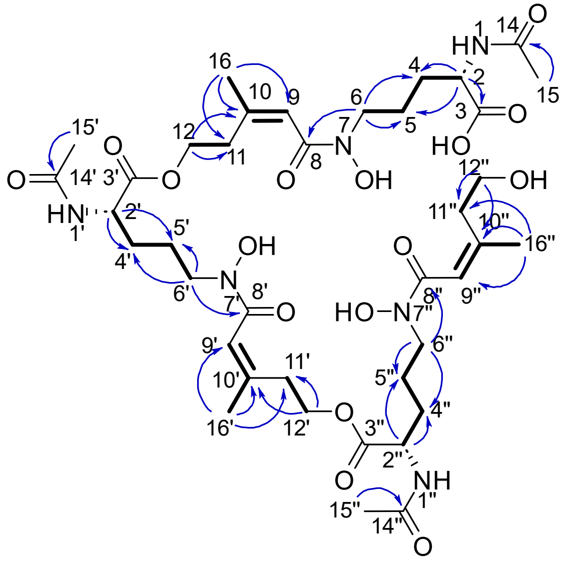

Figure S50. ^{1}H-^{1}H COSY (▬) and ^{1}H-^{13}C HMBC (→) correlations of *N,N',N''*-triacetyl-*Z*-L-fusarinine B (**5**) in CD_3OD

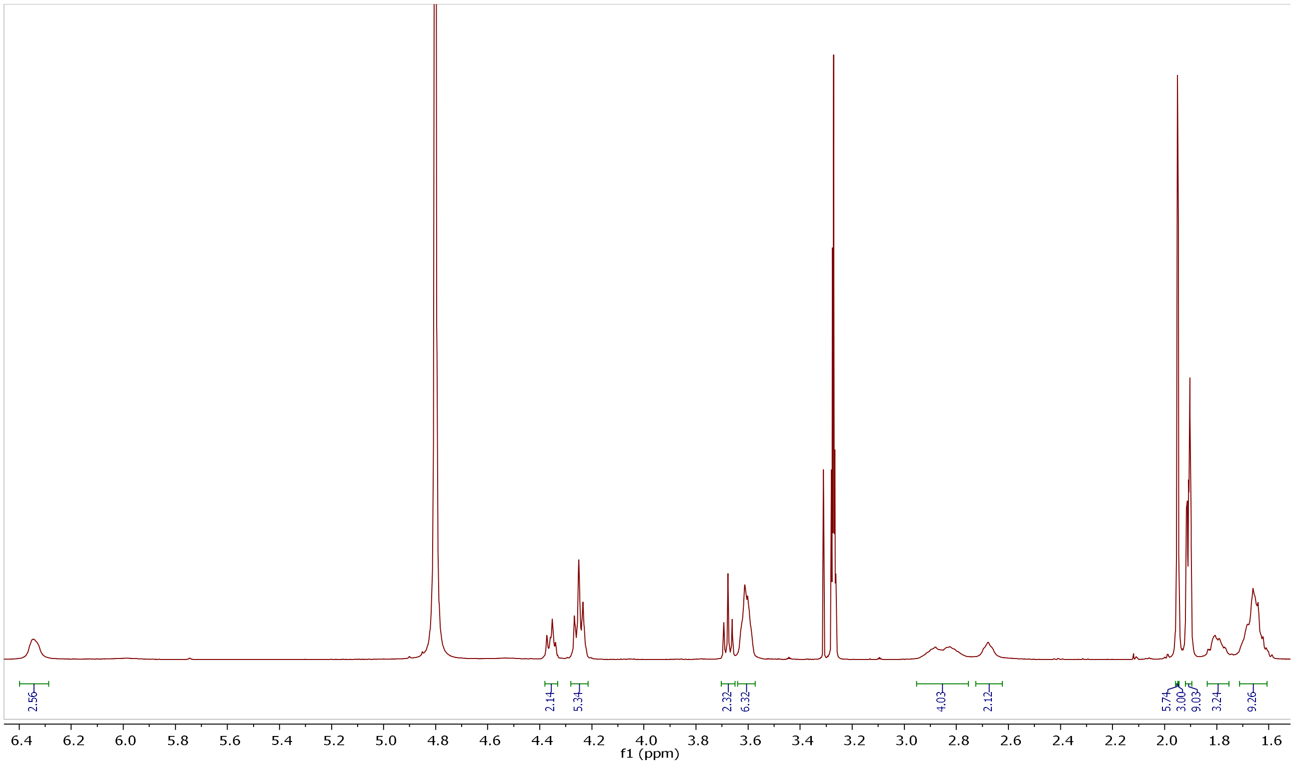

Figure S51. ^{1}H-NMR spectrum of *N,N',N''*-triacetyl-*Z*-L-fusarinine B (**5**) in CD_3OD (400 MHz)

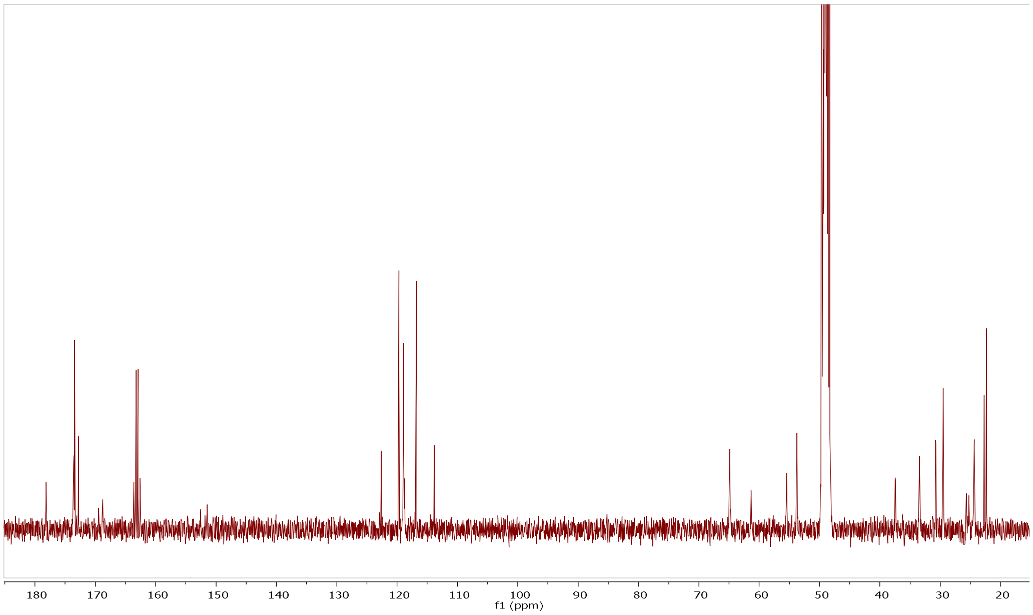

Figure S52. ^{13}C-NMR spectrum of *N,N',N''*-triacetyl-*Z*-L-fusarinine B (**5**) in CD_3OD (100 MHz)

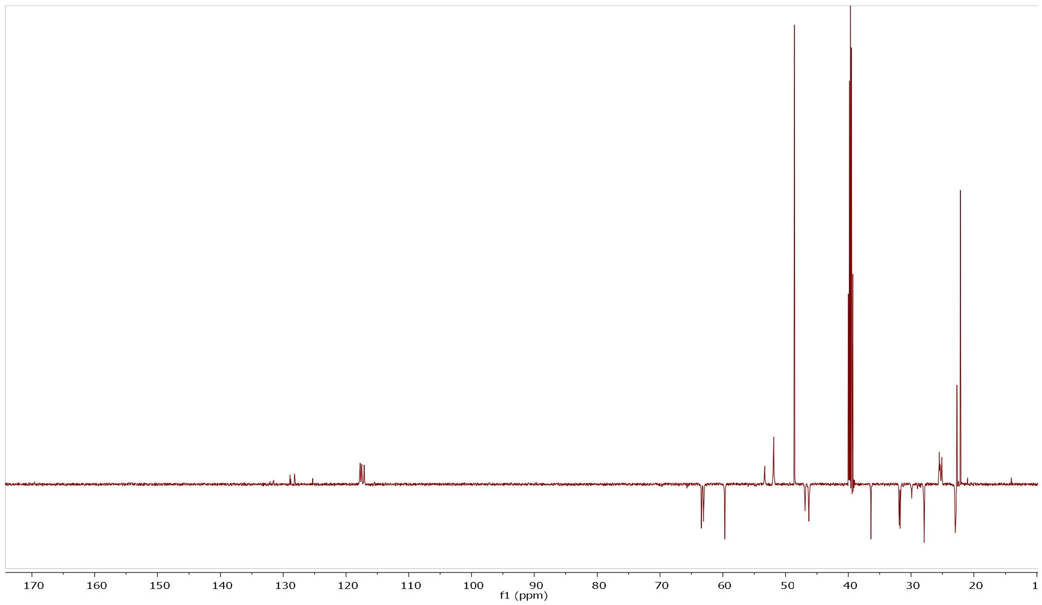

Figure S53. DEPT135-NMR spectrum of *N,N',N''*-triacetyl-*Z*-L-fusarinine B (**5**) in CD_3OD (100 MHz)

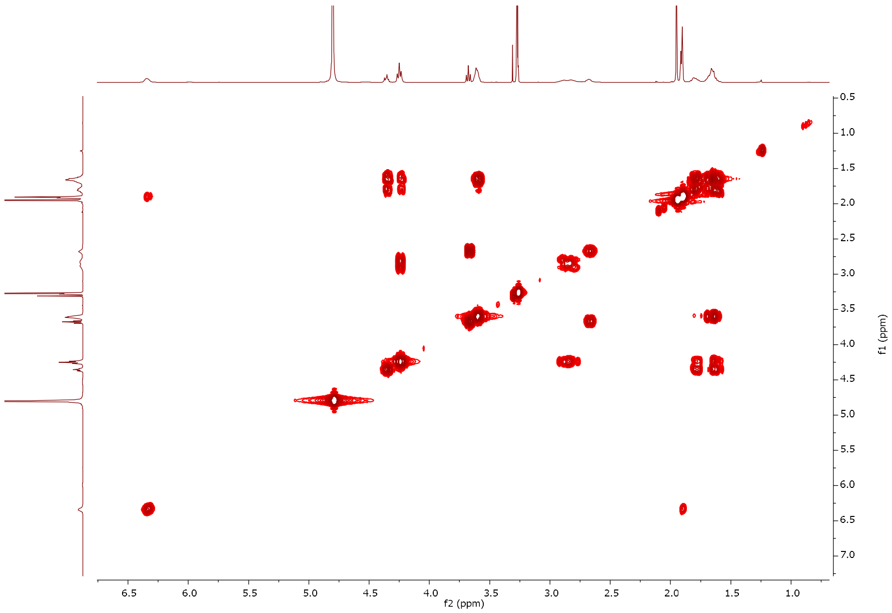

Figure S54. 2D-COSY-NMR spectrum of *N,N',N''*-triacetyl-*Z*-L-fusarinine B (**5**) in CD_3OD

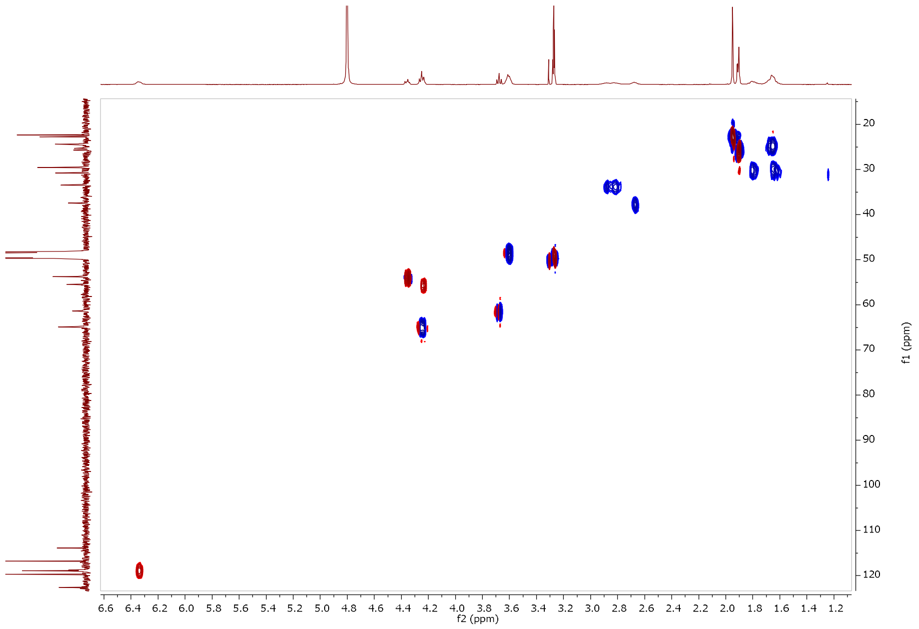

Figure S55. 2D-HSQC-NMR spectrum of *N,N',N''*-triacetyl-*Z*-L-fusarinine B (**5**) in CD_3OD

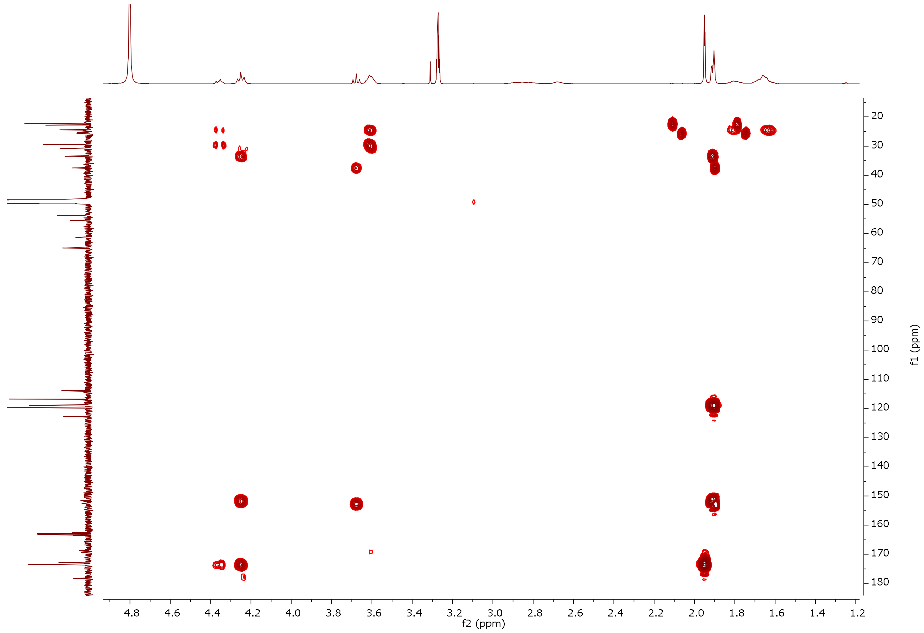

Figure S56. 2D-HMBC-NMR spectrum of *N,N',N''*-triacetyl-*Z*-L-fusarinine B (**5**) in CD_3OD

Table S14. NMR chemical shifts of *N,N',N''*-triacetyl-*Z*-L-fusarinine B (**5**) in $(CD_3)_2SO$ (400 MHz)

**Position**	**δ_C [a] mult**	**δ_H [b] mult (*J* in Hz)**	**∫**	**HMBC (H→C#)**	**COSY (H→H#)**
**1**		7.54 - 7.45, m	1		2
**1''**		8.28, d (7.4)	2	2' 2'', 14' 14''	2' 2''
**2**	53.43, CH	4.00 - 3.94, m	1		4
**2' 2''**	51.97, CH	4.20 - 4.17, m	2	3' 3'', 4' 4''	4' 4''
**3 3' 3''**	172.13, C				
**4 4' 4''**	28.04, CH_2	1.56 - 1.47, m	3		5 5' 5''
		1.70 - 1.56, m	3		
	22,81, CH_2	1.56 - 1.47, m	1		
		1.70 - 1.56, m	1		
**5 5' 5''**	22,94, CH_2	1.56 - 1.47, m	1		4 4' 4'', 6 6' 6''
		1.70 - 1.56, m	1		
	23,11, CH_2	1.56 - 1.47, m	1		
		1.70 - 1.56, m	1		
**6 6' 6''**	46.47, CH_2<br>47.06, CH_2	3.54 - 3.47, m	6	4 4' 4''	4 4' 4''
**8 8' 8''**	157.91, C<br>158.23, C				
**9 9' 9''**	117.22, CH	6.25, s	1		16 16' 16''
	117.89, CH	6.30, s	1		
		6.33, s	1		
**10 10'**	150.12, C				
**10''**	151.52, C				
**11 11'**	32.02, CH_2	2.89 - 2.68, m	4		12 12'
**11''**	36.47, CH_2	2.66 - 2.56, m	2		12''
**12 12'**	63.46, CH_2	4.15 - 4.13, m	4	10 10'	11 11'
**12''**	59.73, CH_2	3.54 - 3.47, m	2	10'', 11''	11''
**14**	168.45, C				
**14' 14''**	169.63, C				
**15**	22.82, CH_3	1.81, s	3	14	
**15' 15''**	22.23, CH_3	1.84, s	6	14' 14''	
**16**	25.10, CH_3	1.87, d (1.5)	3	11	9
**16' 16''**	25.40, CH_3	1.85, d (1.4)	6	11' 11''	9' 9''

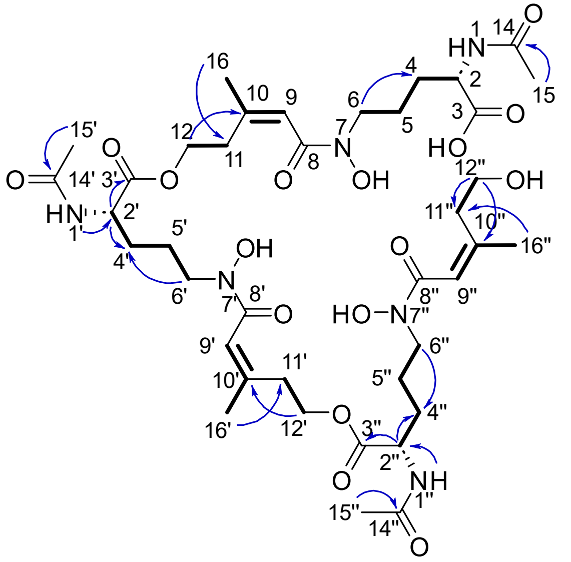

Figure S57. ^{1}H-^{1}H COSY (▬) and ^{1}H-^{13}C HMBC (→) correlations of *N,N',N''*-triacetyl-*Z*-L-fusarinine B (**5**) in $(CD_3)_2SO$

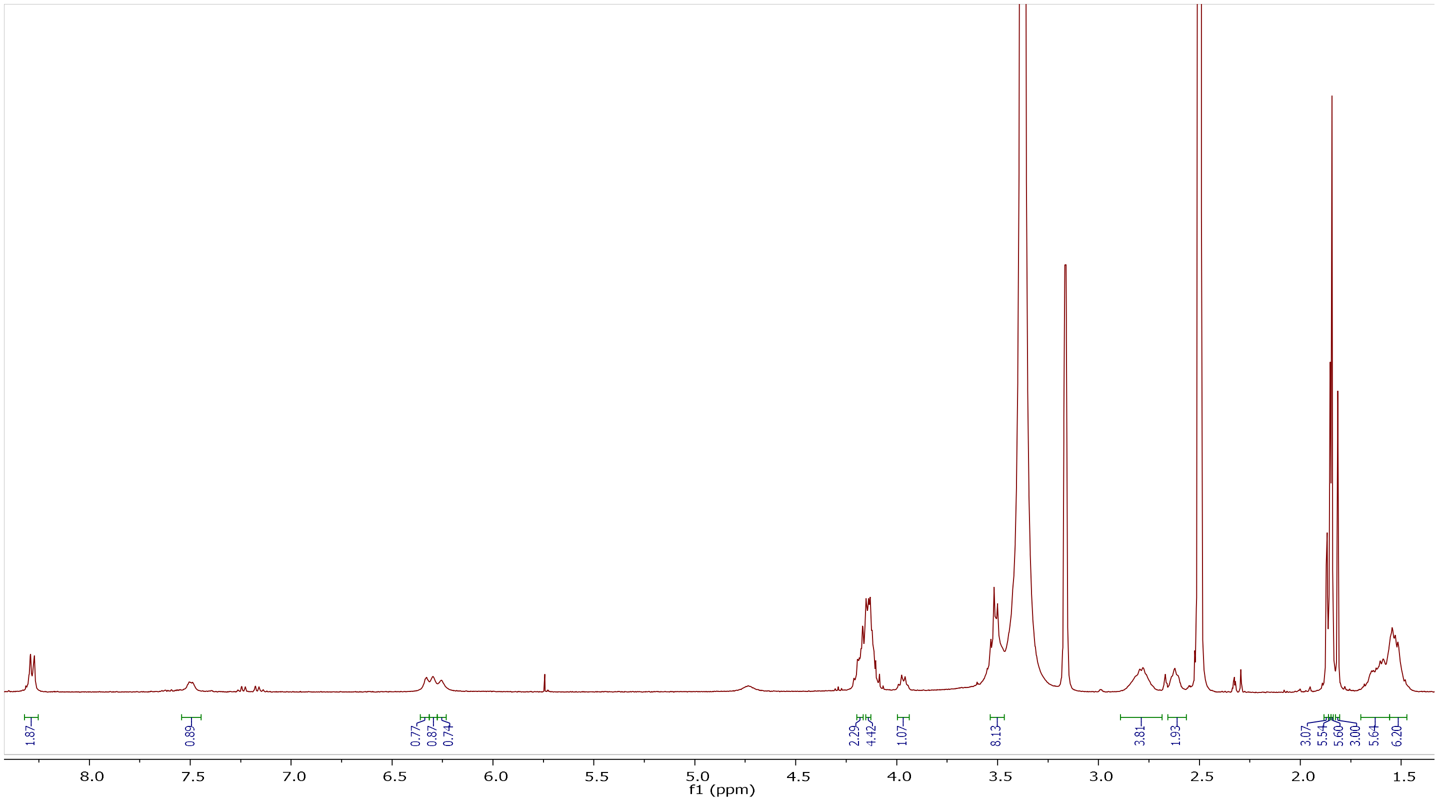

Figure S58. ^{1}H-NMR spectrum of *N,N',N''*-triacetyl-*Z*-L-fusarinine B (**5**) in $(CD_3)_2SO$ (400 MHz)

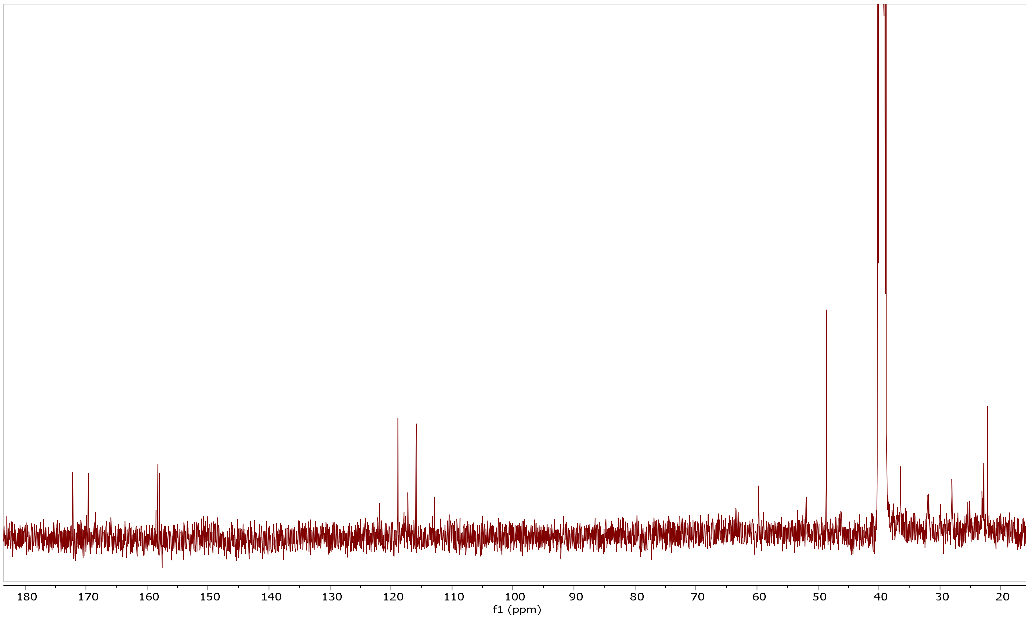

Figure S59. ^{13}C-NMR spectrum of *N,N',N''*-triacetyl-*Z*-L-fusarinine B (**5**) in $(CD_3)_2SO$ (100 MHz)

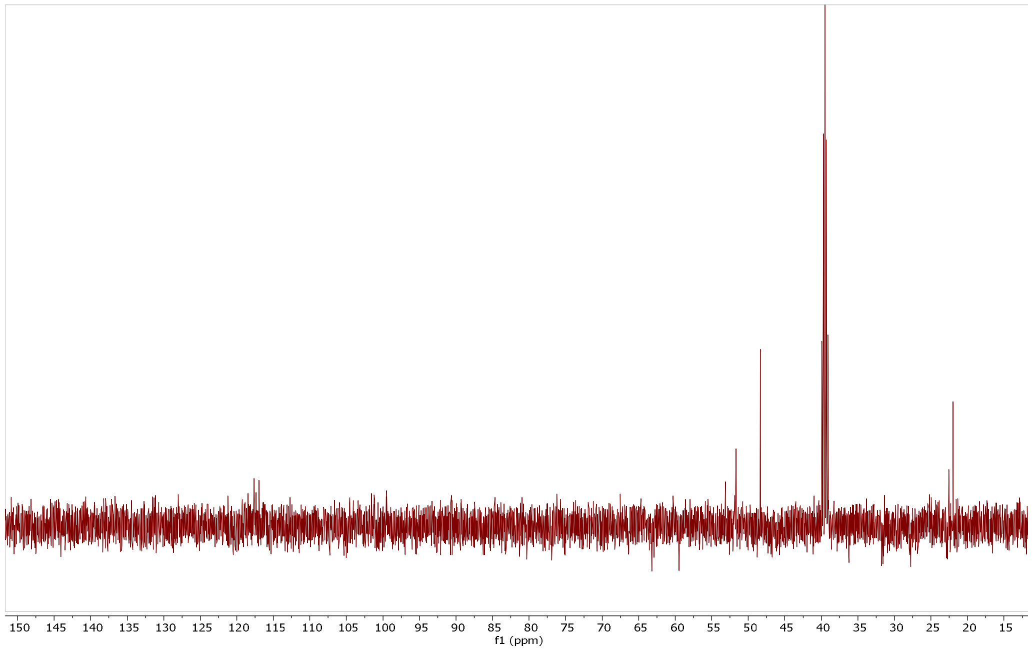

Figure S60. DEPT135-NMR spectrum of *N,N',N''*-triacetyl-*Z*-L-fusarinine B (**5**) in $(CD_3)_2SO$ (100 MHz)

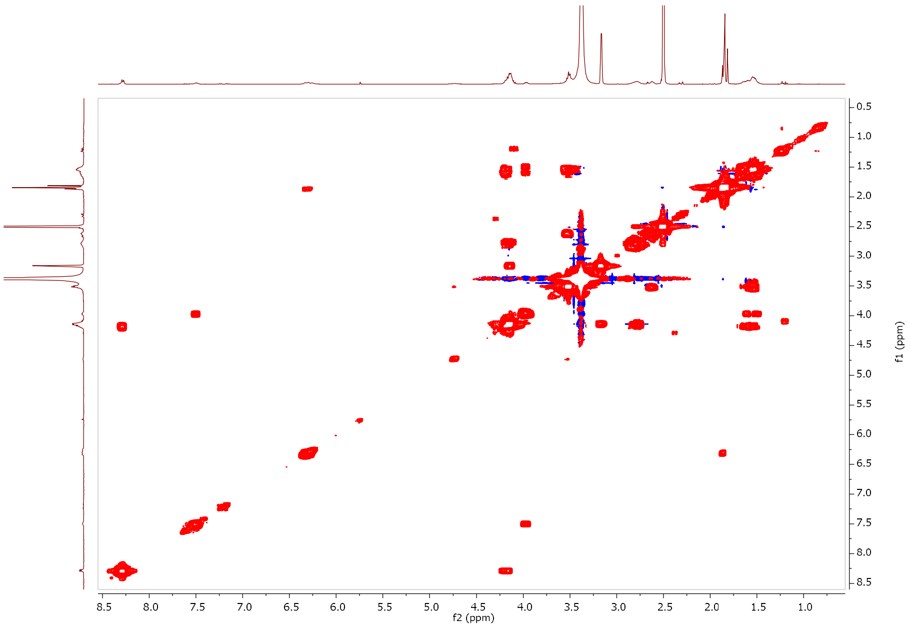

Figure S61. 2D-COSY-NMR spectrum of *N,N',N''*-triacetyl-*Z*-L-fusarinine B (**5**) in $(CD_3)_2SO$

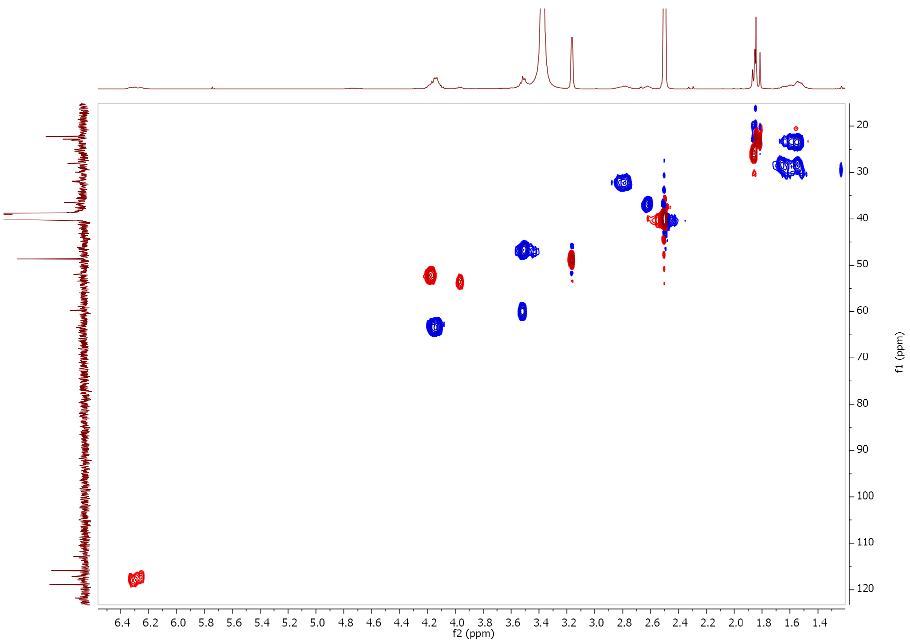

Figure S62. 2D-HSQC-NMR spectrum of *N,N',N''*-triacetyl-*Z*-L-fusarinine B (**5**) in $(CD_3)_2SO$

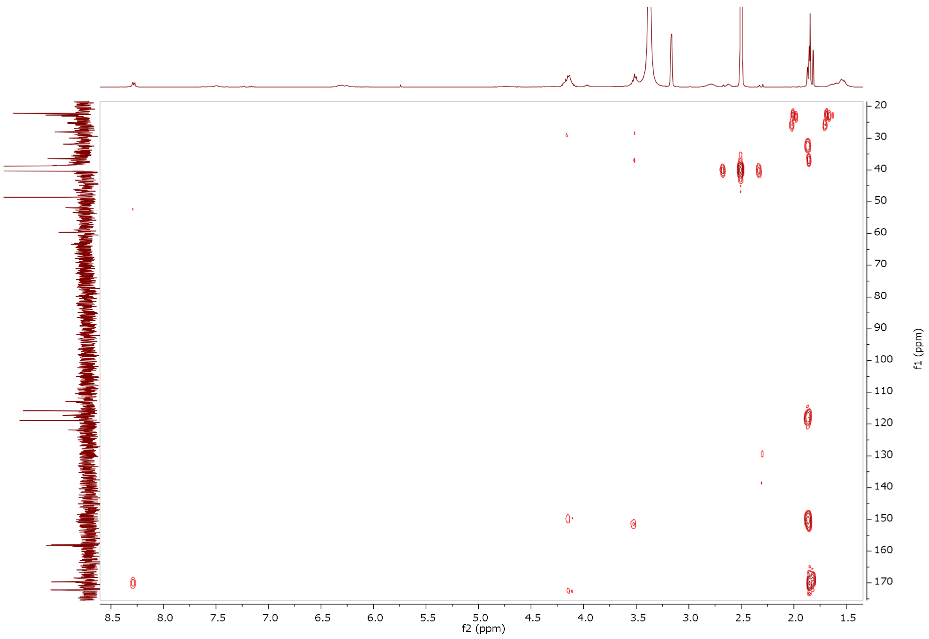

Figure S63. 2D-HMBC-NMR spectrum of *N,N',N''*-triacetyl-*Z*-L-fusarinine B (**5**) in $(CD_3)_2SO$

N,N'-diacetyl-*Z*-L-fusarinine A Ga^{3+} complex (6-Ga)

Table S15. Major *m/z* ions in (+) and (-) HRMS-ESI of *N,N'*-diacetyl-*Z*-L-fusarinine A Ga^{3+} complex (**6-Ga**)

Adduct	Observed *m/z*	Theoretical *m/z*	Error [ppm]
[M-2H+$^{69/71}$Ga]$^+$	653.1948/655.1942	653.1950/655.1941	0.3/0.2
[MCOONa-2H+$^{69/71}$Ga]$^+$	675.1767/677.1760	675.1770/677.1761	0.4/0.1
[M-3H+$^{69/71}$Ga-H]$^-$	651.1791/653.1783	651.1794/653.1785	0.5/0.3

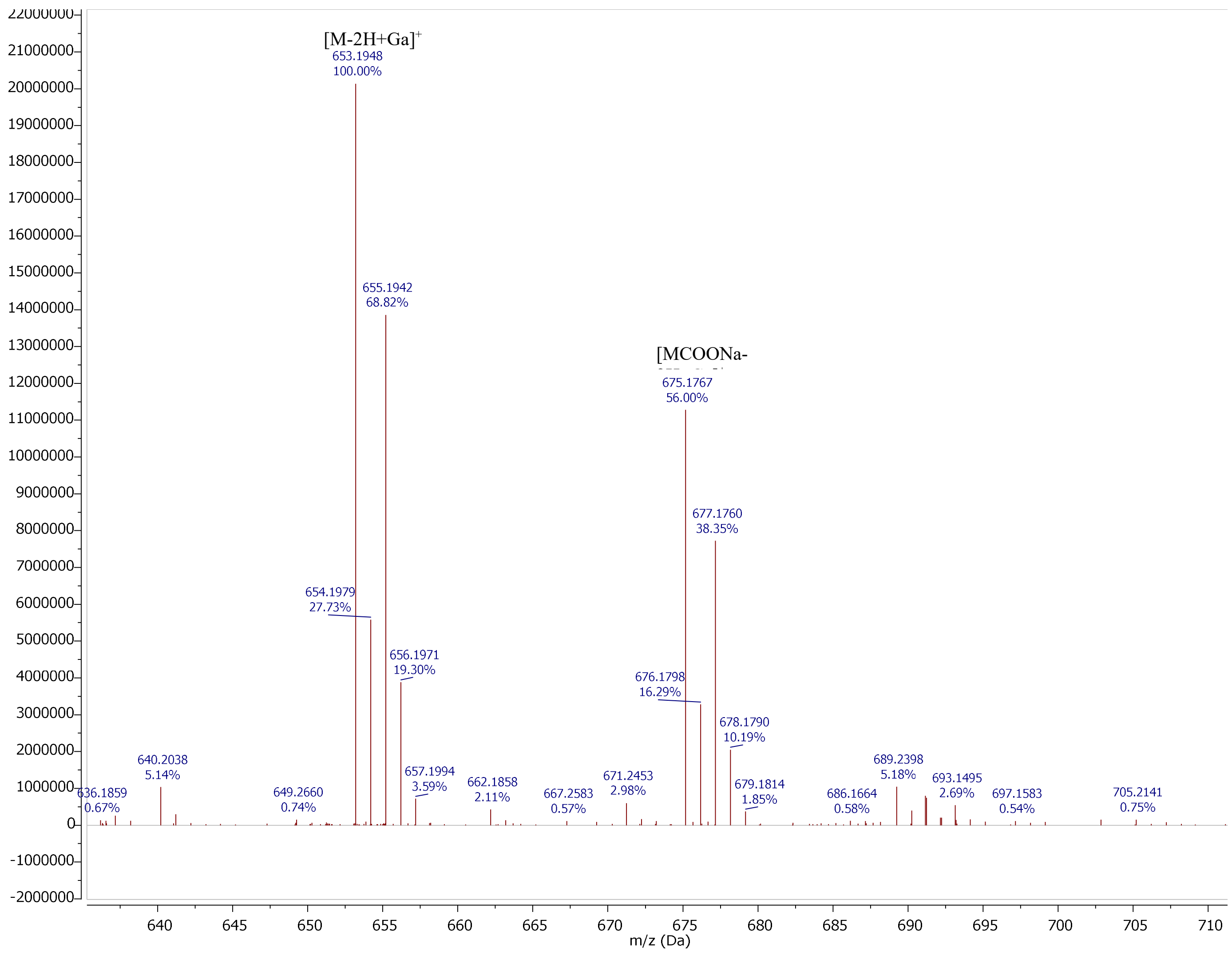

Figure S64. (+) HRMS-ESI of *N,N'*-diacetyl-*Z*-L-fusarinine A Ga^{3+} complex (**6-Ga**)

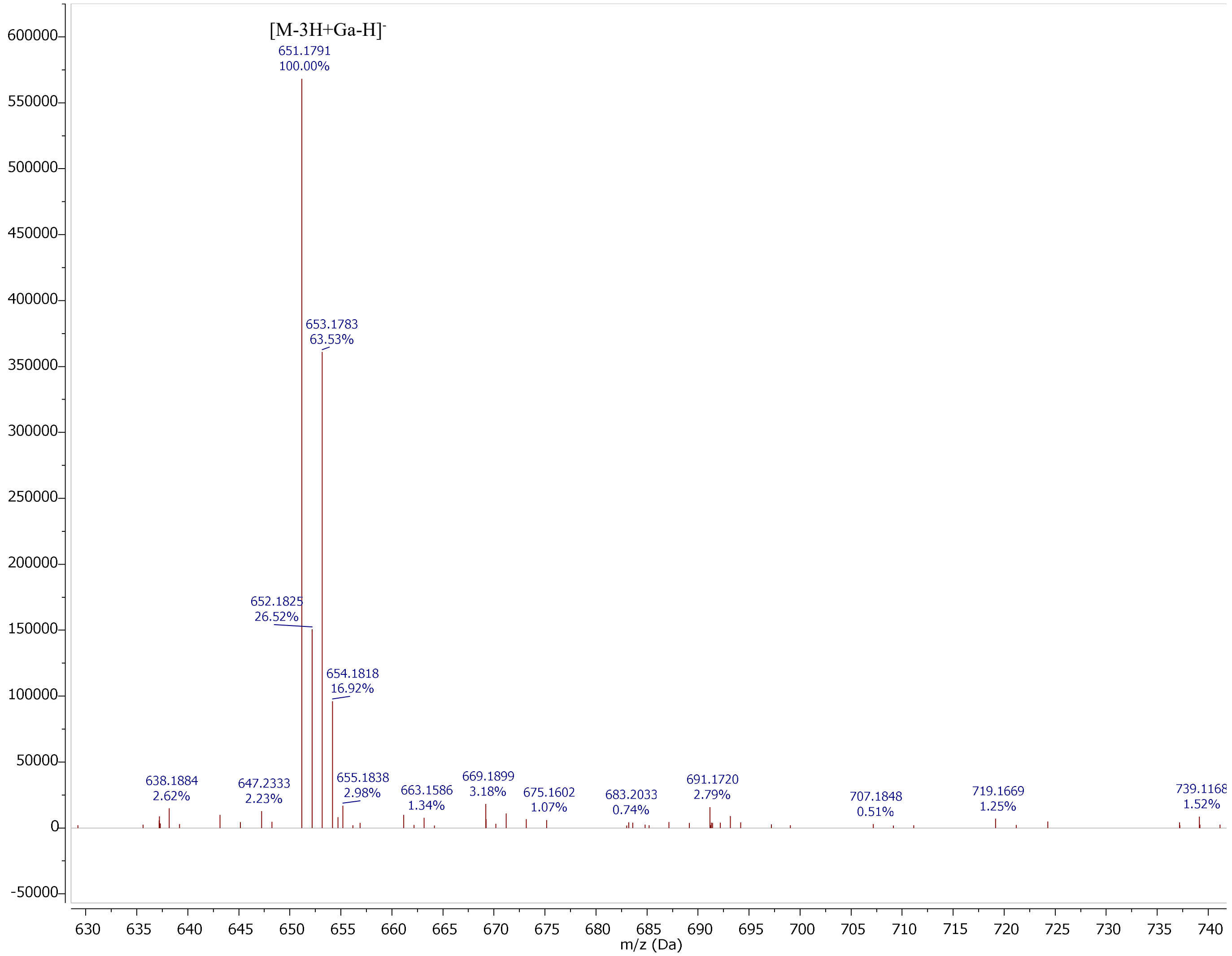

Figure S65. (-) HRMS-ESI of *N,N'*-diacetyl-*Z*-L-fusarinine A Ga^{3+} complex (**6-Ga**)

Table S16. NMR chemical shifts of *N,N'*-diacetyl-*Z*-L-fusarinine A Ga^{3+} complex (**6-Ga**) in CD_3OD (500 MHz)

Position	δ_C [a] mult	δ_H [b] mult (*J* in Hz)	∫	HMBC (H→C#)	COSY (H→H#)
**2 2'**	55.45, CH<br>54.89, CH	4.29 - 4.26, m<br>4.31 - 4.29, m	1<br>1		4 4'
**3 3'**	177.76, C<br>178.15, C				
**4 4'**	29.94, CH_2<br>20.70, CH_2	1.64 - 1.74, m<br>1.88 - 1.79, m	2<br>2		5 5'
**5 5'**	24.42, CH_2	1.64 - 1.74, m<br>1.88 - 1.79, m	2<br>2		2 2', 4 4'
**6 6'**	48.55, CH_2<br>51.90, CH_2	3.69 - 3.61, m<br>2.85 - 2.74, m	2<br>2		5 5'
**8 8'**	162.93, C<br>163.26, C				
**9**	119.12, CH	6.35, d (16.1)	1	11, 16	16
**9'**	114.44, CH	6.12, d (20.0)	1	11', 16'	16'
**10**	152.22, C				
**10'**	152.54, C				
**11**	37.42, CH_2	2.70 - 2.67, m<br>2.77 - 2.71, m	1<br>1		12
**11'**	38.03, CH_2	2.65 - 2.56, m<br>2.77 - 2.71, m	1<br>1		12'
**12**	70.63, CH_2	3.69 - 3.61, m	2		11
**12'**	61.37, CH_2	3.71, t (6.4)	2		11'
**14 14'**	172.84, C				
**15 15'**	22.77, CH_3<br>22.68, CH_3	1.987, s<br>1.994, s	3<br>3	14 14'	
**16**	25.55, CH_3	1.93, d (1.5)	3	9, 10, 11	9
**16'**	25.25, CH_3	1.97, d (1.4)	3	9', 10', 11'	9'

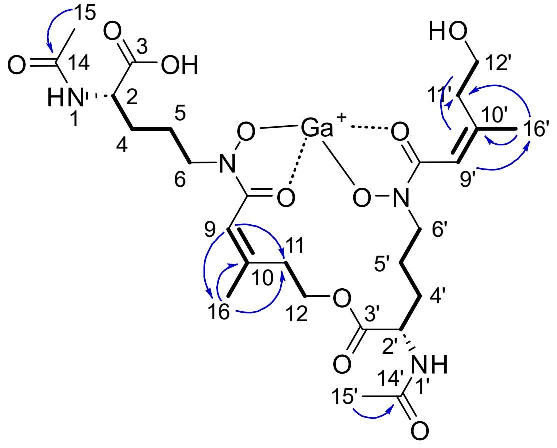

Figure S66. ^{1}H-^{1}H COSY (▬) and ^{1}H-^{13}C HMBC (⟶) correlations of *N,N'*-diacetyl-*Z*-L-fusarinine A Ga^{3+} complex (**6-Ga**) in CD_3OD

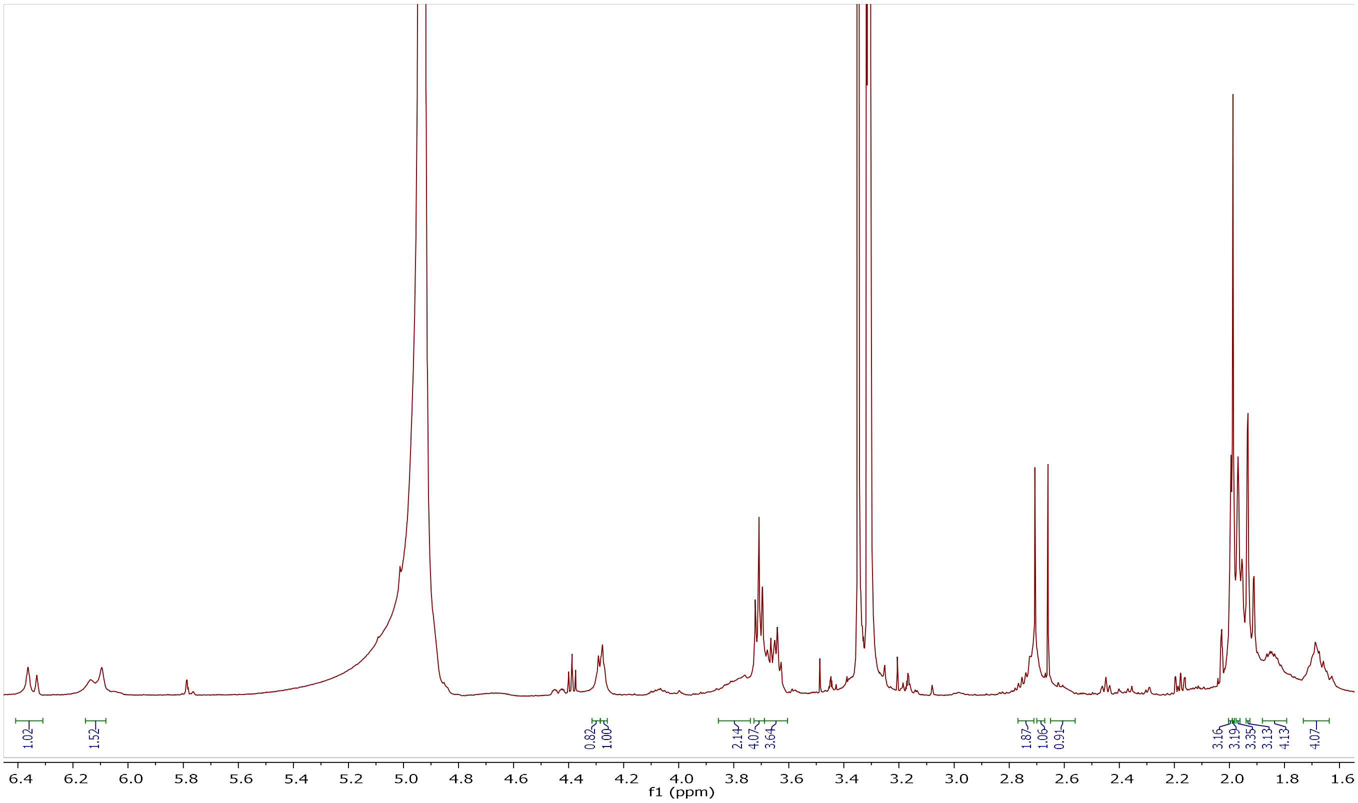

Figure S67. ^{1}H-NMR spectrum of *N,N'*-diacetyl-*Z*-L-fusarinine A Ga^{3+} complex (**6-Ga**) in CD_3OD (500 MHz)

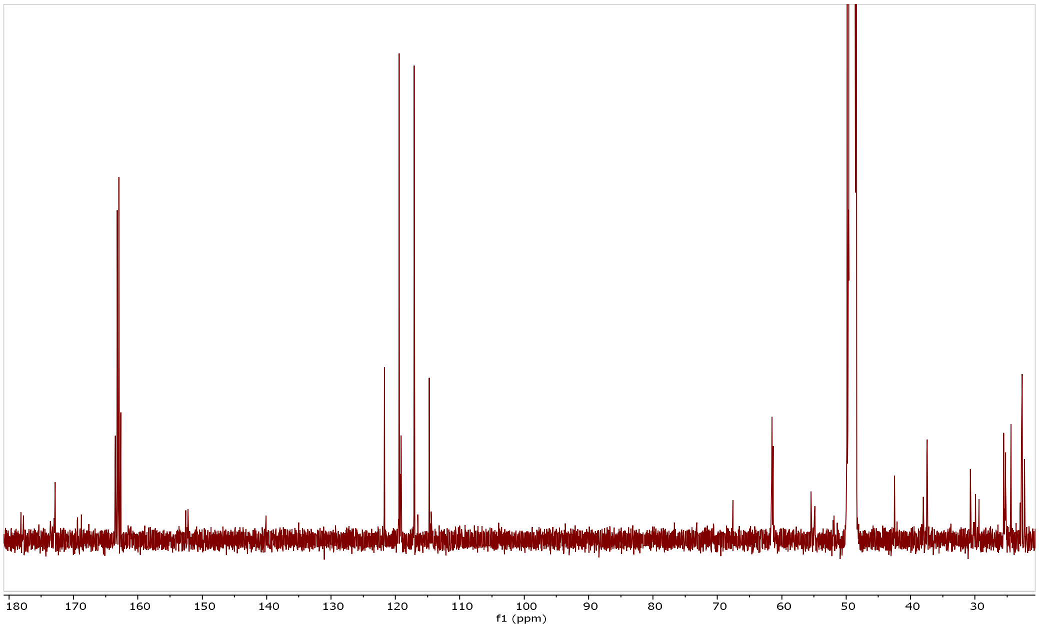

Figure S68. ^{13}C-NMR spectrum of *N,N'*-diacetyl-*Z*-L-fusarinine A Ga^{3+} complex (**6-Ga**) in CD_3OD (125 MHz)

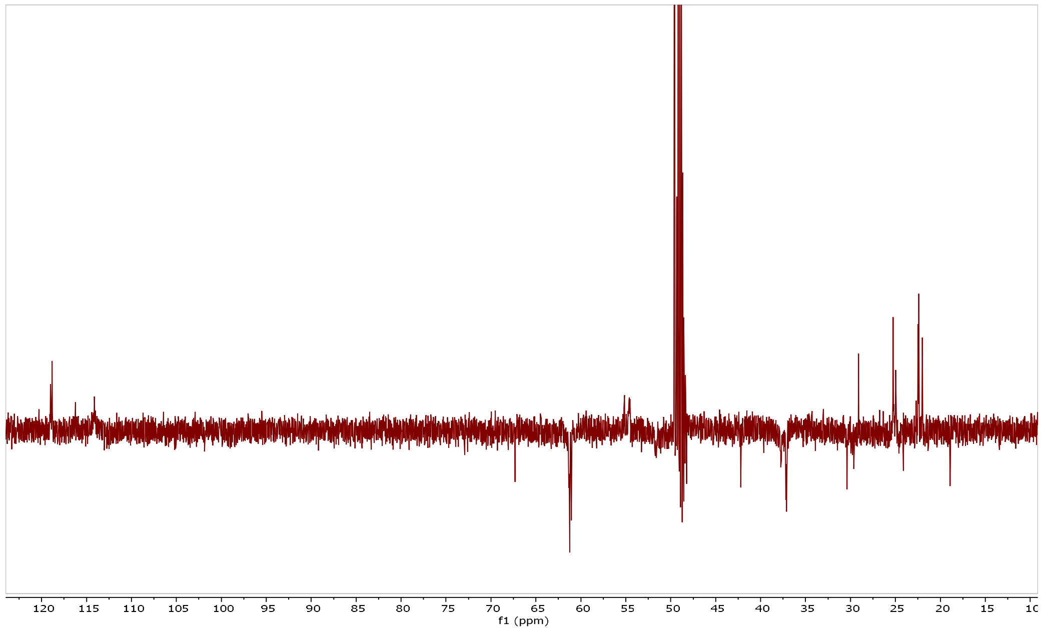

Figure S69. DEPT135-NMR spectrum of *N,N'*-diacetyl-*Z*-L-fusarinine A Ga^{3+} complex (**6-Ga**) in CD_3OD (125 MHz)

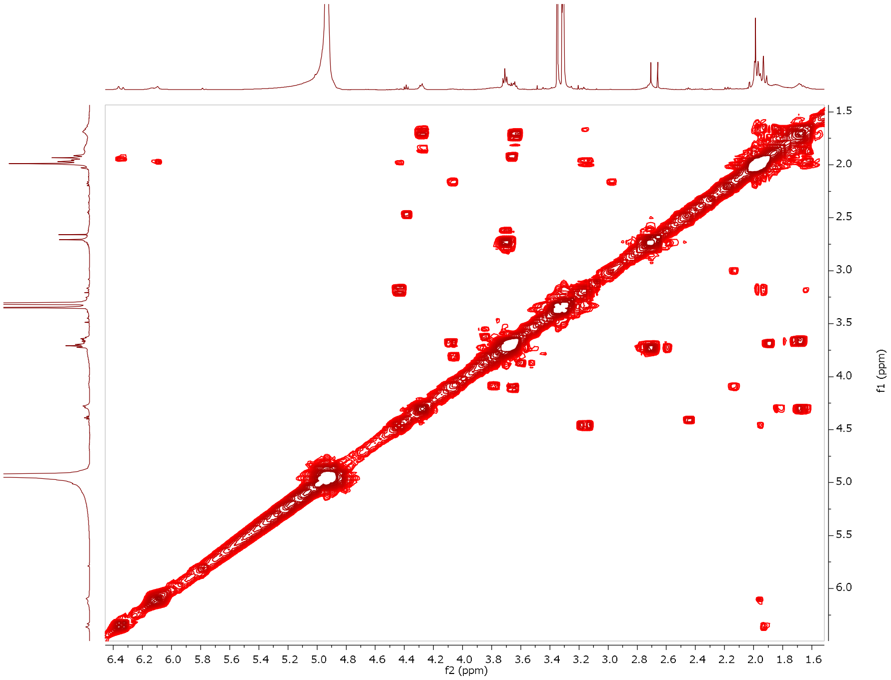

Figure S70. 2D-COSY-NMR spectrum of *N,N'*-diacetyl-*Z*-L-fusarinine A Ga^{3+} complex (**6-Ga**) in CD_3OD

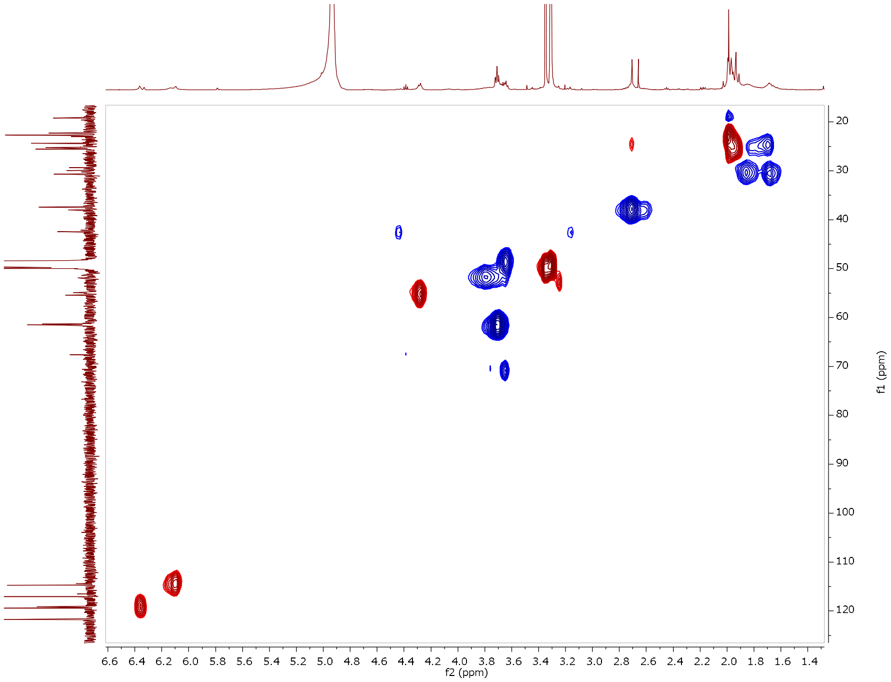

Figure S71. 2D-HSQC-NMR spectrum of *N,N'*-diacetyl-*Z*-L-fusarinine A Ga^{3+} complex (**6-Ga**) in CD_3OD

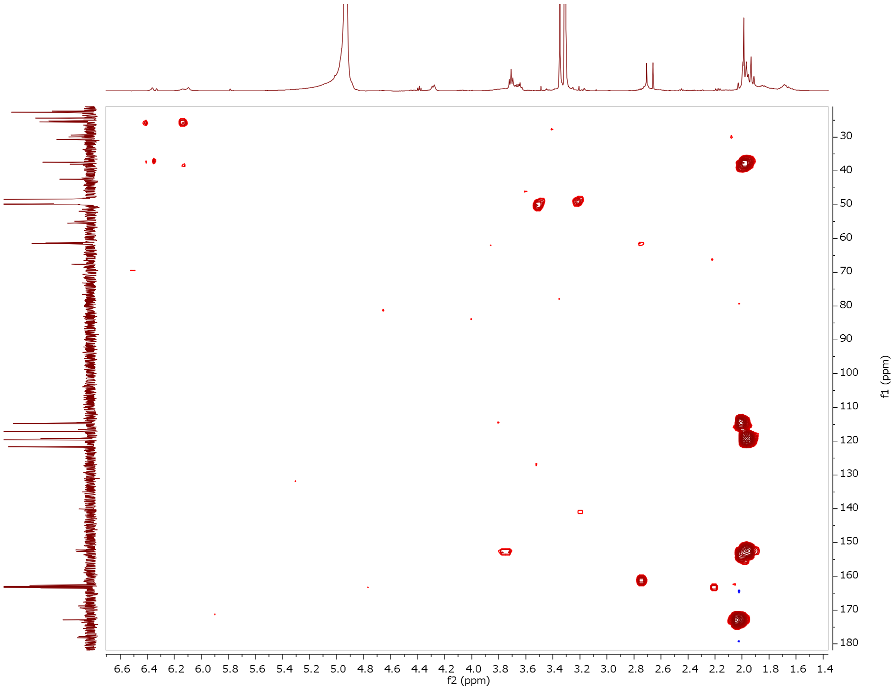

Figure S72. 2D-HMBC-NMR spectrum of *N,N'*-diacetyl-*Z*-L-fusarinine A Ga^{3+} complex (**6-Ga**) in CD_3OD

N,N'-diacetyl-*Z*-L-fusarinine A (6)

Table S17. Major *m/z* ions in (+) and (-) HRMS-ESI of *N,N'*-diacetyl-*Z*-L-fusarinine A (**6**)

Adduct	Observed *m/z*	Theoretical *m/z*	Error [ppm]
$[M+Na]^+$	609.2746	609.2748	0.3
$[M+K]^+$	625.2475	625.2487	1.9
$[MCOONa+Na]^+$	631.2564	631.2568	0.6
$[MCOONa+K]^+$	647.2305	647.2307	0.3
$[M-H]^-$	585.2777	585.2772	0.8

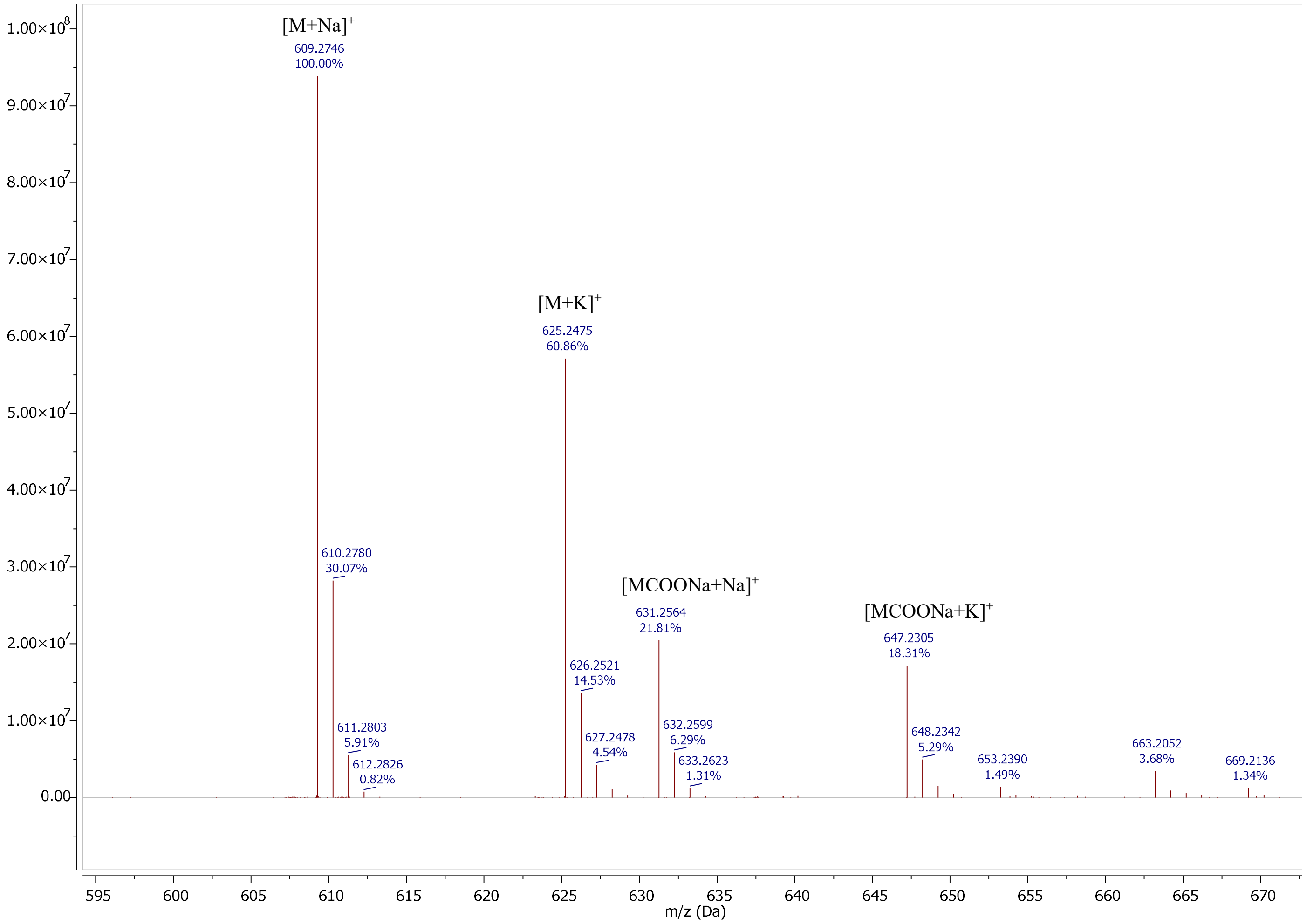

Figure S73. (+) HRMS-ESI of *N,N'*-diacetyl-*Z*-L-fusarinine A (**6**)

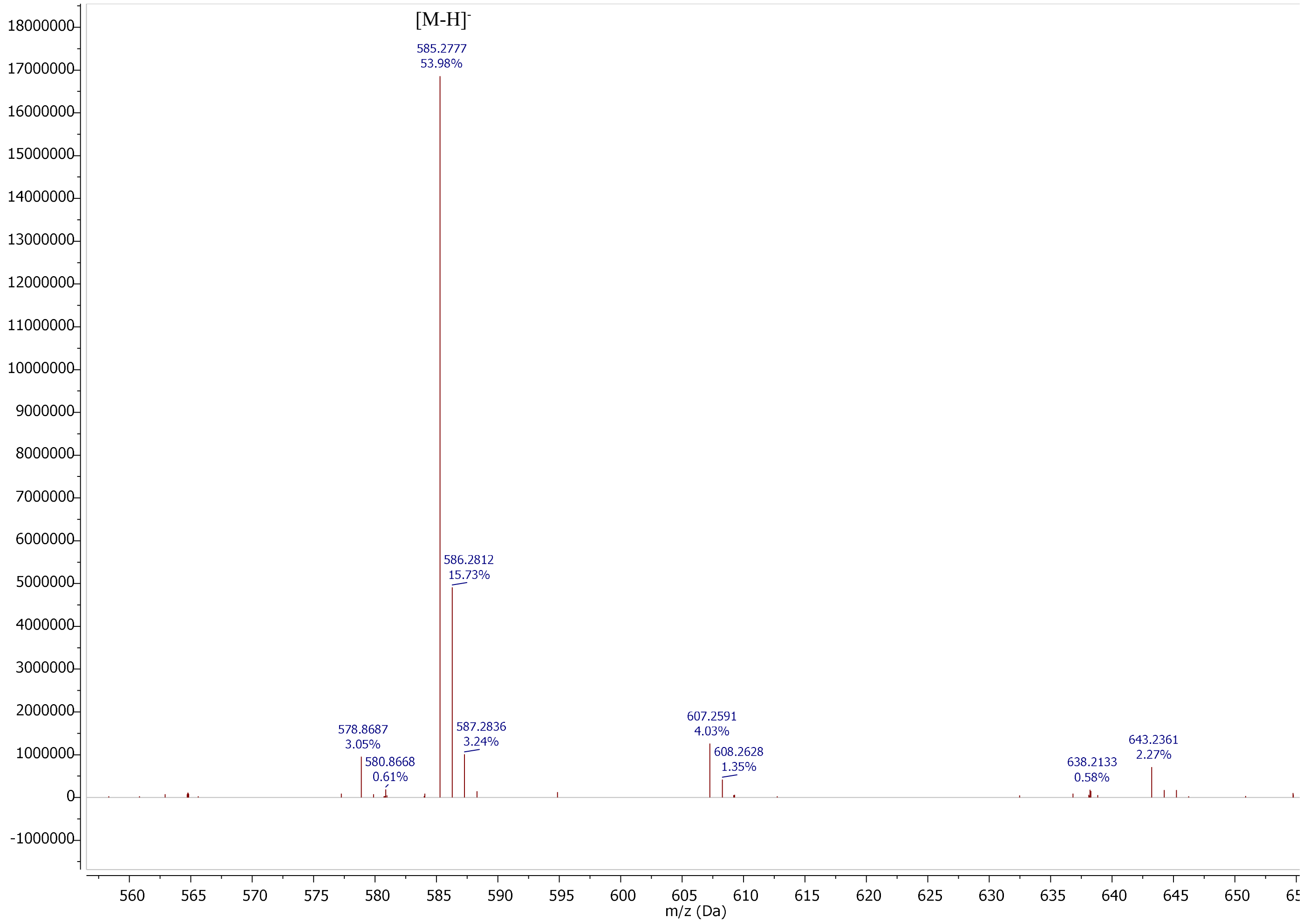

Figure S74. (-) HRMS-ESI of *N,N'*-diacetyl-*Z*-L-fusarinine A (**6**)

Table S18. NMR chemical shifts of *N,N'*-diacetyl-*Z*-L-fusarinine A (**6**) in CD_3OD (400 MHz)

Position	δ_C [a] mult	δ_H [b] mult (*J* in Hz)	∫	HMBC (H→C#)	COSY (H→H#)
**2**	55.10, CH	4.29 - 4.21, m	1	3	4, 5
**2'**	53.70, CH	4.39 - 4.33, m	1	3', 4', 5'	4', 5'
**3**	177.79, C				
**3'**	173.64, C				
**4**	29.45, CH_2	1.72 - 1.59, m	2	2, 5	2, 5
**4'**	30.57, CH_2	1.84 - 1.75, m	2	5'	2', 5'
**5 5'**	24.34, CH_2	1.72 - 1.59, m	4		6 6'
**6 6'**	47.03, CH_2	3.61, td (6.4, 2.7)	4	4 4', 5 5'	5 5'
**8 8'**	168.73, C<br>169.46, C				
**9 9'**	118.96, CH	6.34, brs	2		16 16'
**10**	151.44, C				
**10'**	152.51, C				
**11**	33.40, CH_2	2.96 - 2.76, m	2		12
**11'**	37.43, CH_2	2.68, t (6.6)	2	10'	12'
**12**	64.87, CH_2	4.29 - 4.21, m	2	10, 11	11
**12'**	61.32, CH_2	3.68, t (6.4)	2	10', 11'	11'
**14**	173.45, C				
**14'**	172.92, C				
**15**	22.68, CH_3	1.953, s	3	14	
**15'**	22.34, CH_3	1.95, s	3	14'	
**16**	25.58, CH_3	1.91, d (1.4)	3	9, 10, 11	9
**16'**	25.25, CH_3	1.90, d(1.4)	3	9, 10', 11'	9'

Figure S75. ^{1}H-^{1}H COSY (▬) and ^{1}H-^{13}C HMBC (→) correlations of *N,N'*-diacetyl-*Z*-L-fusarinine A (**6**) in CD_3OD

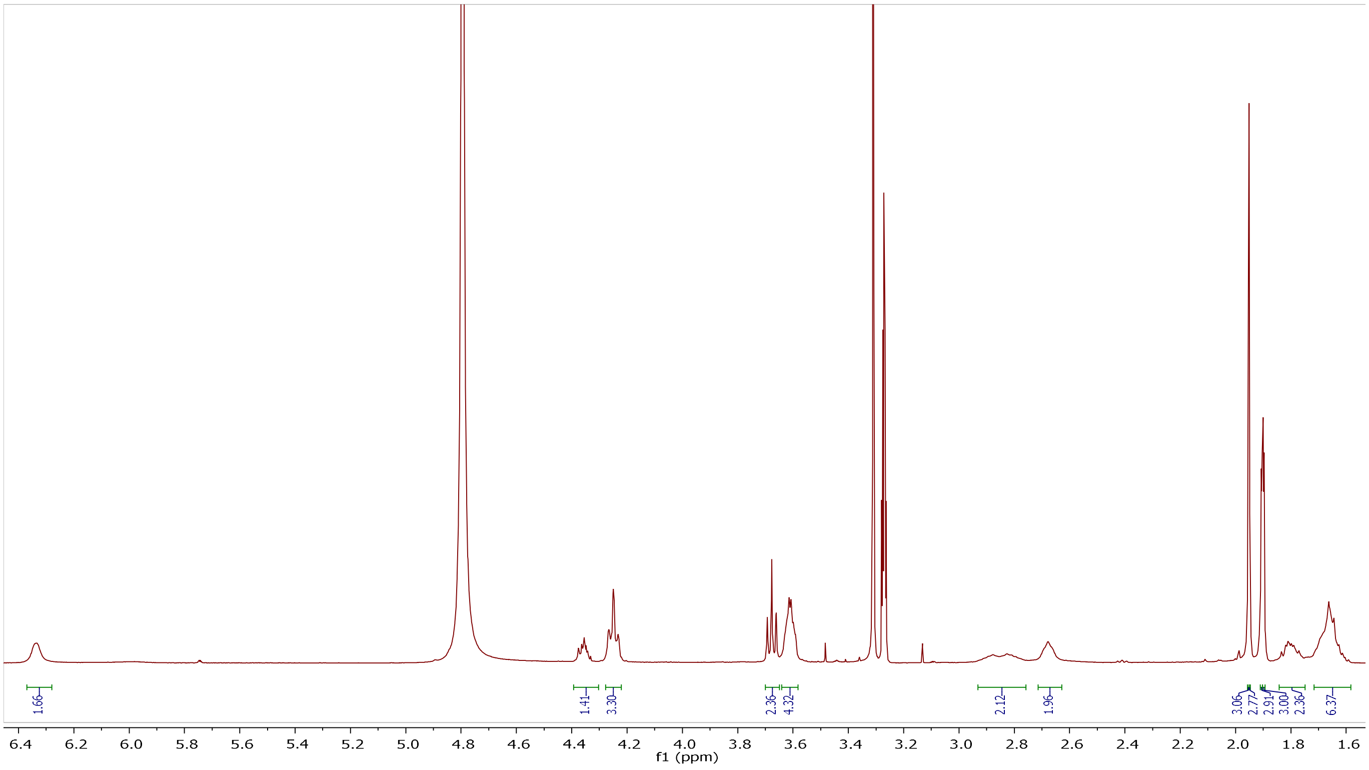

Figure S76. ^{1}H-NMR spectrum of *N,N'*-diacetyl-*Z*-L-fusarinine A (**6**) in CD_3OD (400 MHz)

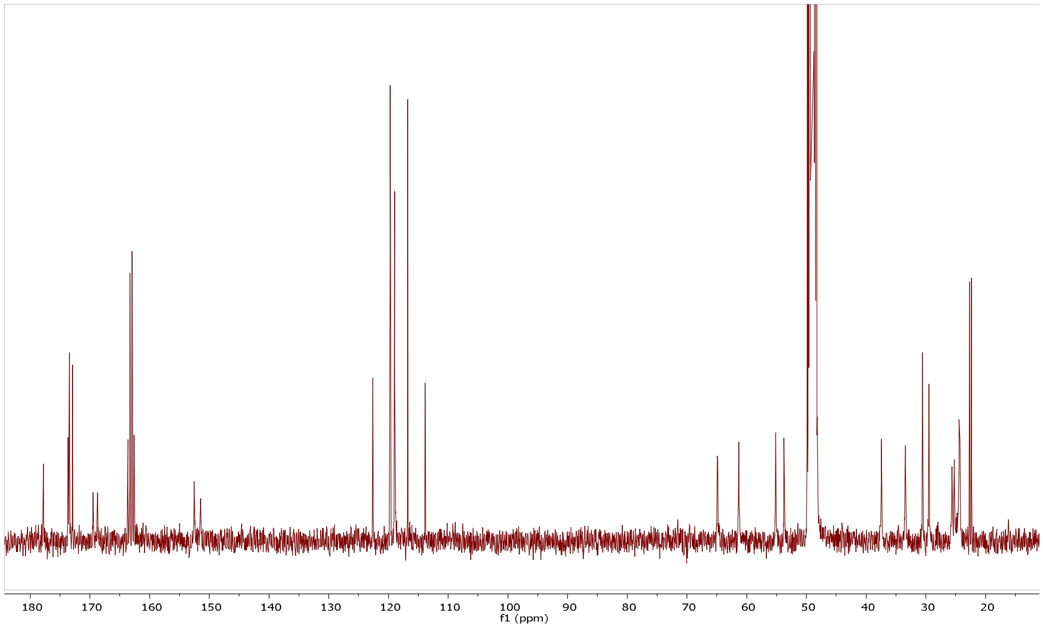

Figure S77. ^{13}C-NMR spectrum of *N,N'*-diacetyl-*Z*-L-fusarinine A(**6**) in CD_3OD (100 MHz)

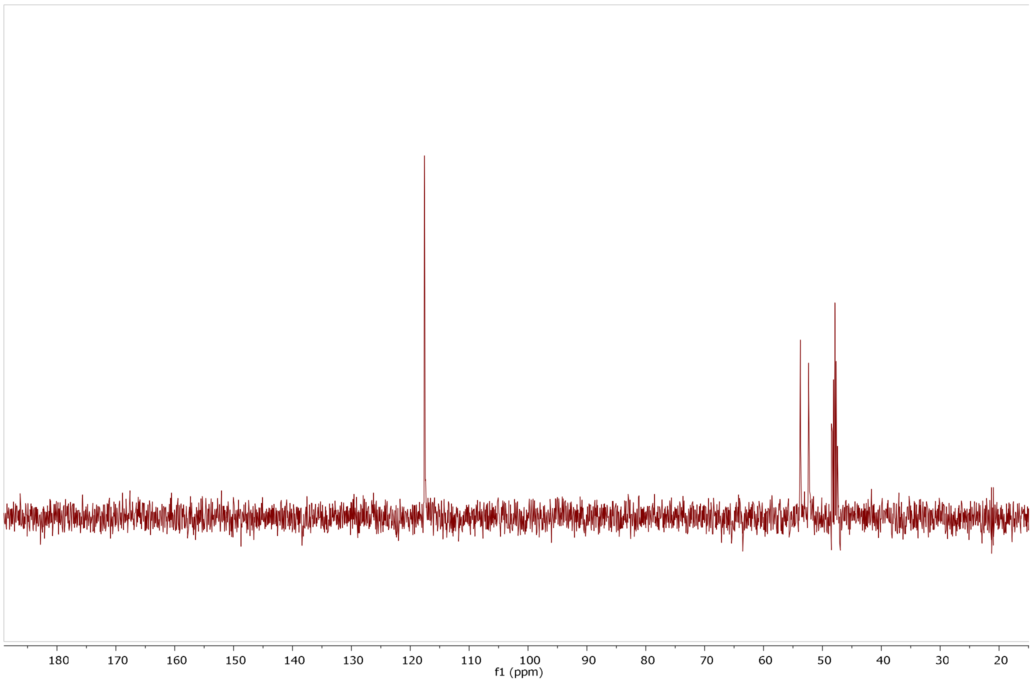

Figure S78. DEPT135-NMR spectrum of *N,N'*-diacetyl-*Z*-L-fusarinine A (**6**) in CD_3OD (100 MHz)

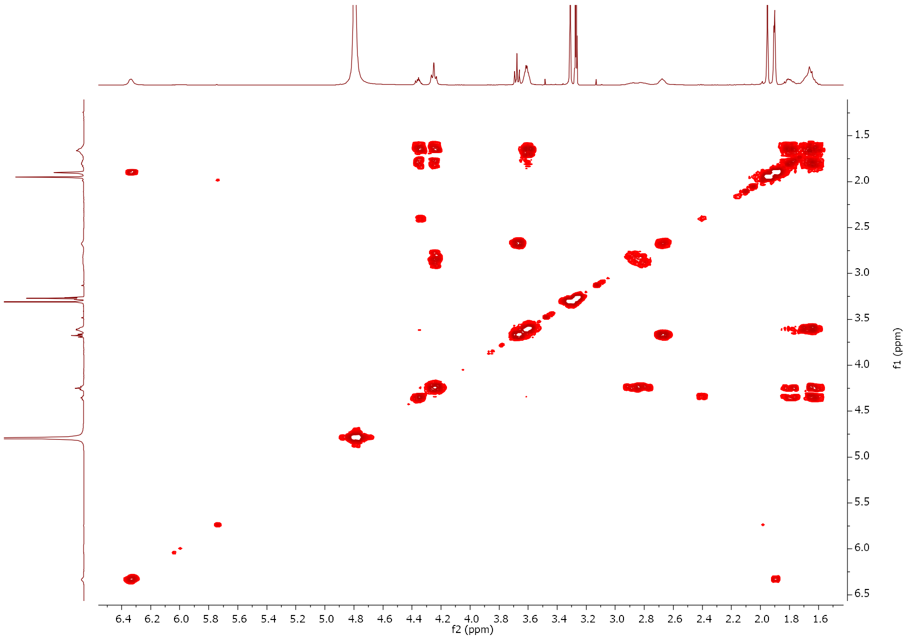

Figure S79. 2D-COSY-NMR spectrum of *N,N'*-diacetyl-*Z*-L-fusarinine A (**6**) in CD_3OD

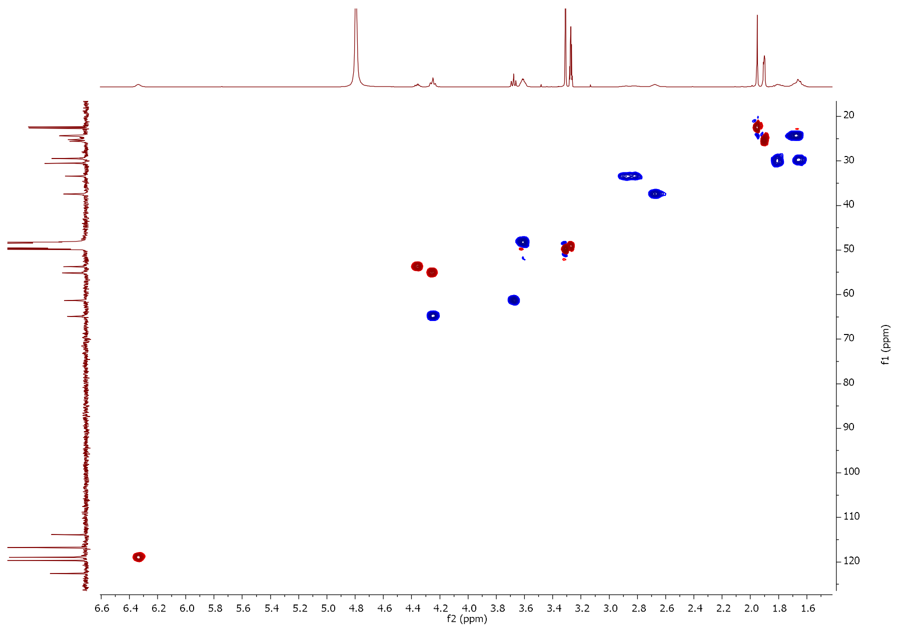

Figure S80. 2D-HSQC-NMR spectrum of *N,N'*-diacetyl-*Z*-L-fusarinine A (**6**) in CD_3OD

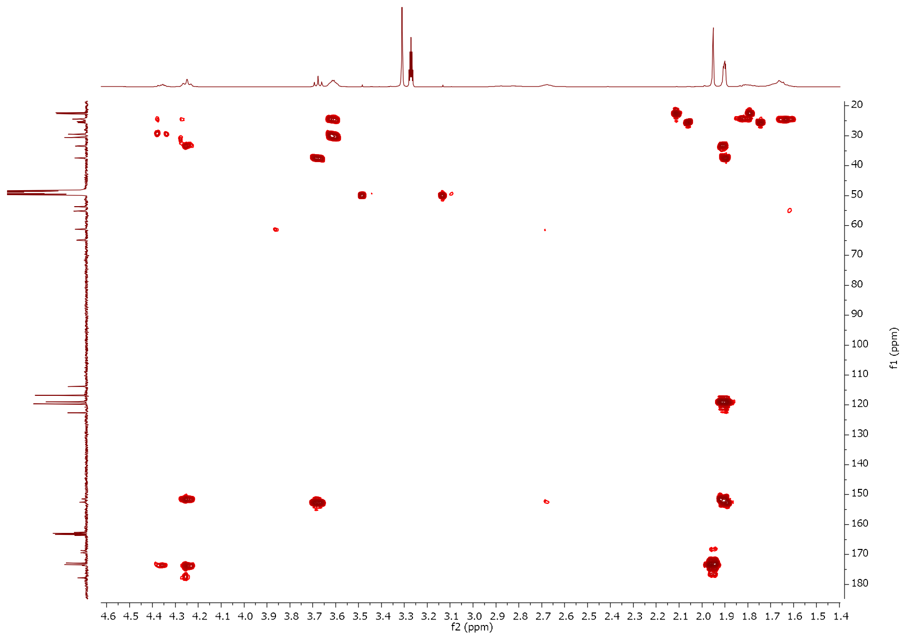

Figure S81. 2D-HMBC-NMR spectrum of *N,N'*-diacetyl-*Z*-L-fusarinine A (**6**) in CD_3OD

Table S19. NMR chemical shifts of *N,N'*-diacetyl-*Z*-L-fusarinine A (**6**) $(CD_3)_2SO$ (400 MHz)

Position	δ_C [a] mult	δ_H [b] mult (*J* in Hz)	∫	HMBC (H→C#)	COSY (H→H#)
**1**		7.64 - 7.59, m	1		2
**1'**		8.31, d (7.5)	1	14'	2'
**2**	51.99, CH	3.96 - 4.02, m	1		1, 4
**2'**	53.21, CH	4.19 - 4.13, m	1		1', 4'
**3 3'**	172.14, C				
**4 4'**	28.03, CH_2	1.57 - 1.51, m	1		5 5'
		1.67 - 1.57, m	1		
	29.74, CH_2	1.57 - 1.51, m	1		
		1.67 - 1.57, m	1		
**5 5'**	23.11, CH_2	1.57 - 1.51, m	1		4 4'
		1.67 - 1.57, m	1		
**6 6'**	46.90, CH_2	3.54 - 3.49, m	4		5 5'
**8 8'**	164.36, C				
**9**	117.81, CH	6.30, s	1		16
**9**	117.31, CH	6.26, s	1		16'
**10'**	149.10, C				
**10**	150.88, C				
**11**	31.93, CH_2	2.86 – 2.72			12
**11'**	36.52, CH_2	2.65 – 2.58			12'
**12**	63.26, CH_2	4.19 – 4.13			11
**12'**	59.74, CH_2	3.54 – 3.49		11	11'
**14**	168.65, C				
**14'**	169.63, C				
**15**	22.25, CH_3	1.82, brs		14	
**15'**	22.77, CH_3	1.85, s		14'	
**16**	25.46, CH_3	1.86, d (1.7)		9', 10', 11'	9'
**16'**	25.30, CH_3	1.85, d (1.4)		9, 10, 11	9

Figure S82. ^{1}H-^{1}H COSY (▬) and ^{1}H-^{13}C HMBC (→) correlations of *N,N'*-diacetyl-*Z*-L-fusarinine A (**6**) in $(CD_3)_2SO$

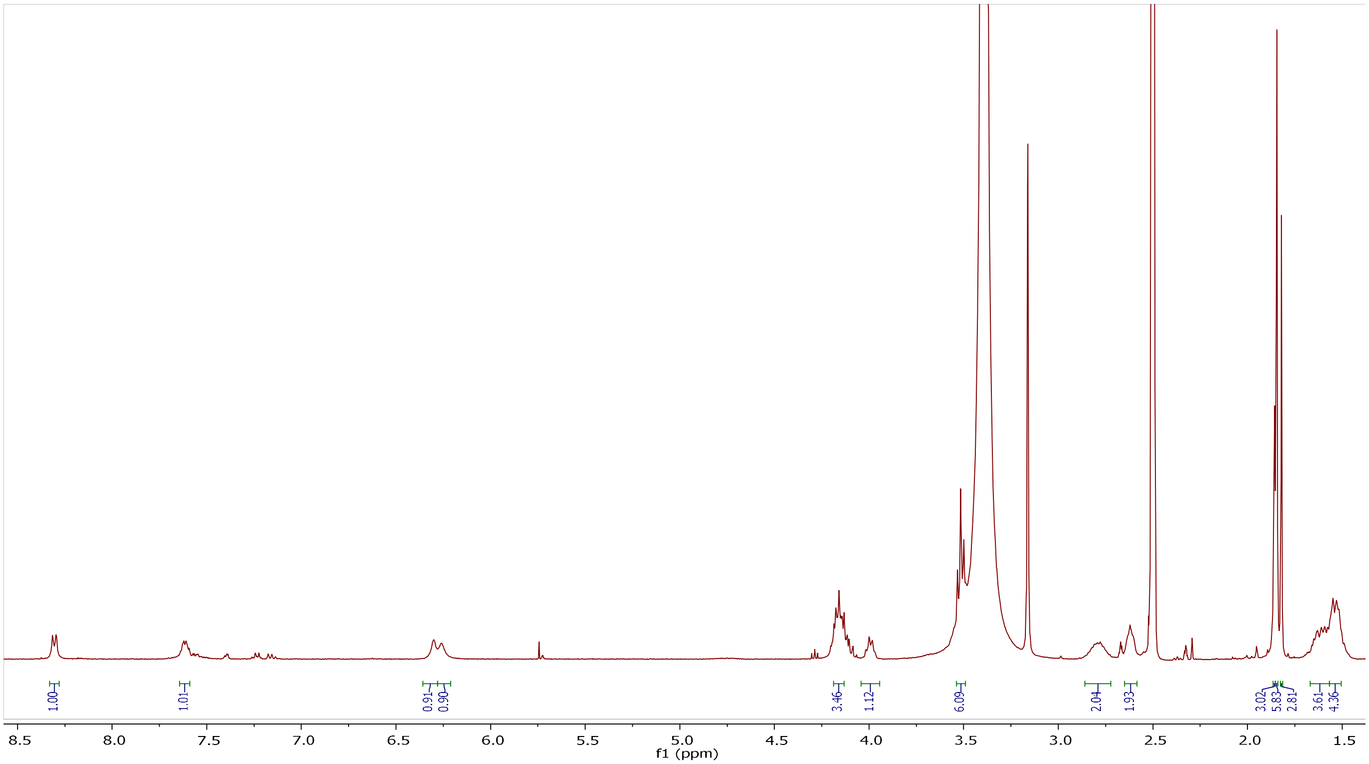

Figure S83. ^{1}H-NMR spectrum of *N,N'*-diacetyl-*Z*-L-fusarinine A (**6**) in $(CD_3)_2SO$ (400 MHz)

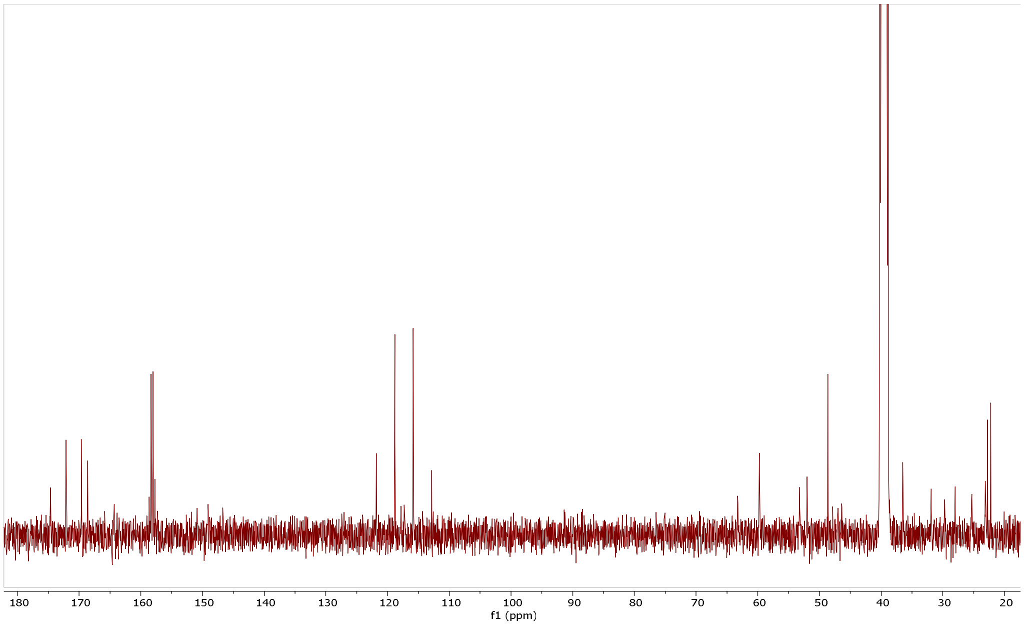

Figure S84. ^{13}C-NMR spectrum of *N,N'*-diacetyl-*Z*-L-fusarinine A (**6**) in $(CD_3)_2SO$ (100 MHz)

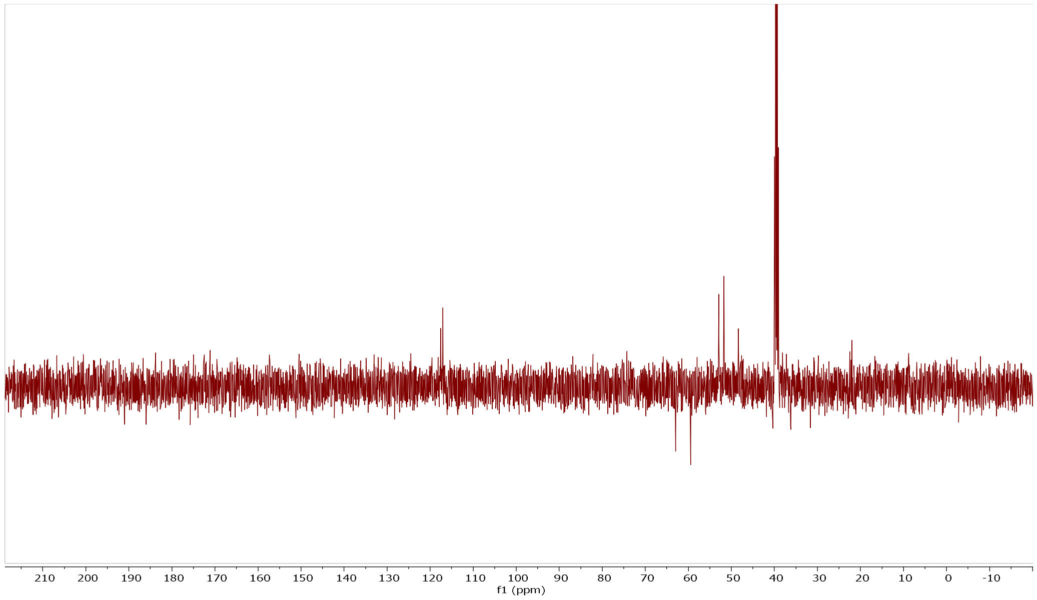

Figure S85. DEPT135-NMR spectrum of *N,N'*-diacetyl-*Z*-L-fusarinine A (**6**) in $(CD_3)_2SO$ (100 MHz)

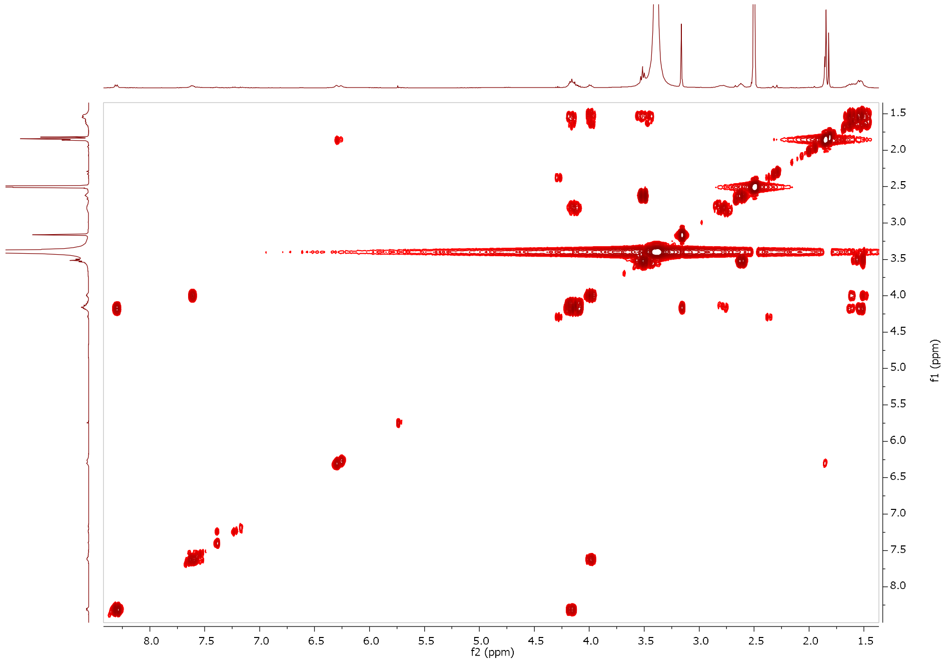

Figure S86. 2D-COSY-NMR spectrum of *N,N'*-diacetyl-*Z*-L-fusarinine A (**6**) in $(CD_3)_2SO$

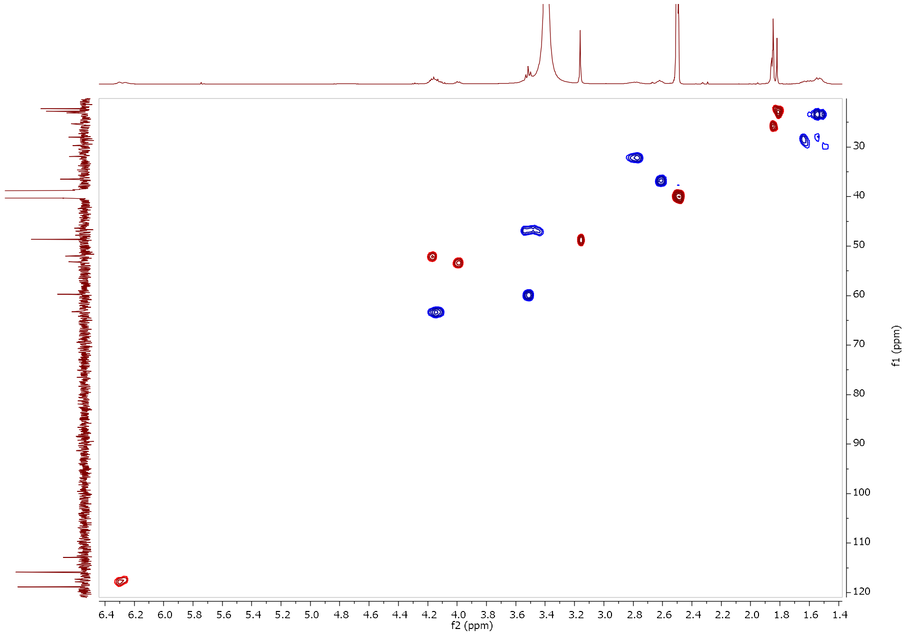

Figure S87. 2D-HSQC-NMR spectrum of *N,N'*-diacetyl-*Z*-L-fusarinine A (**6**) in $(CD_3)_2SO$

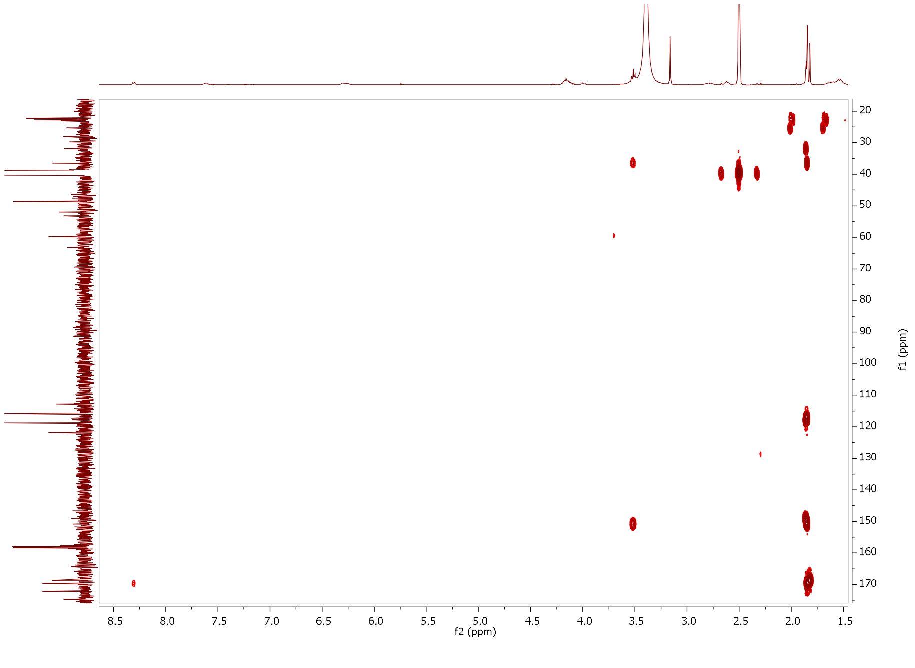

Figure S88. 2D-HMBC-NMR spectrum of *N,N'*-diacetyl-*Z*-L-fusarinine A (**6**) in $(CD_3)_2SO$

N,N'-diacetyl-Z-L-fusarinine B Ga^{3+} complex (7-Ga)

Table S20. Major *m/z* ions in the (+) and (-) HRMS-ESI of *N,N'*-diacetyl-*Z*-L-fusarinine B Ga^{3+} complex (**7-Ga**)

Adduct	Observed *m/z*	Theoretical *m/z*	Error [ppm]
$[M\text{-}3H+^{69/71}Ga+H]^+$	895.3231/897.3234	895.3217/897.3208	1.6/2.3
$[MCOONa\text{-}3H+^{69/71}Ga]^+$	917.3050/919.3054	917.3037/919.3028	1.4/2.8
$[MCOOK\text{-}3H+^{69/71}Ga]^+$	933.2775/935.2772	933.2776/935.2767	0.1/0.5
$[M\text{-}3H+^{69/71}Ga\text{-}H]^-$	893.3057/895.3054	893.3061/895.3052	0.4/0.2

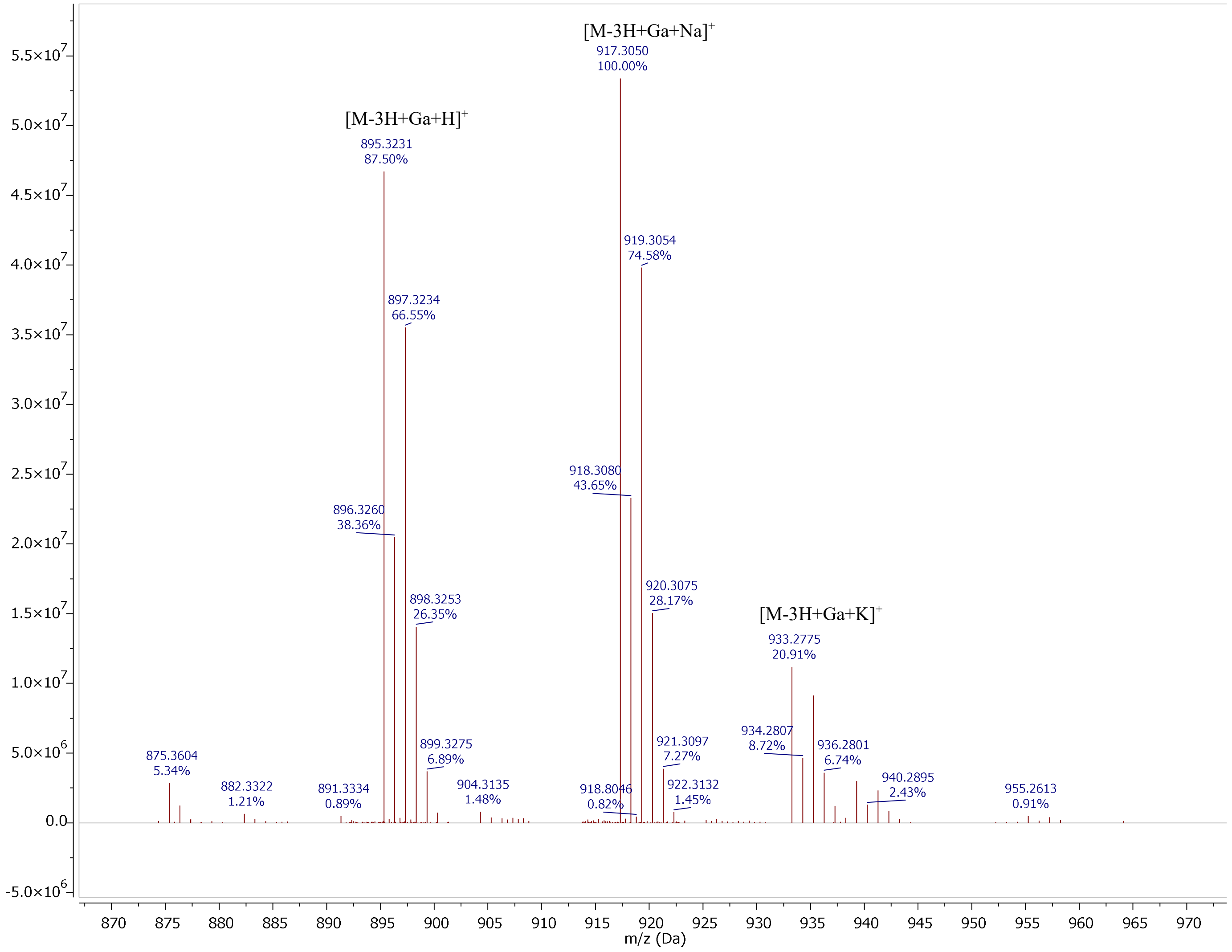

Figure S89. (+) HRMS-ESI of *N,N'*-diacetyl-*Z*-L-fusarinine B Ga^{3+} complex (**7-Ga**)

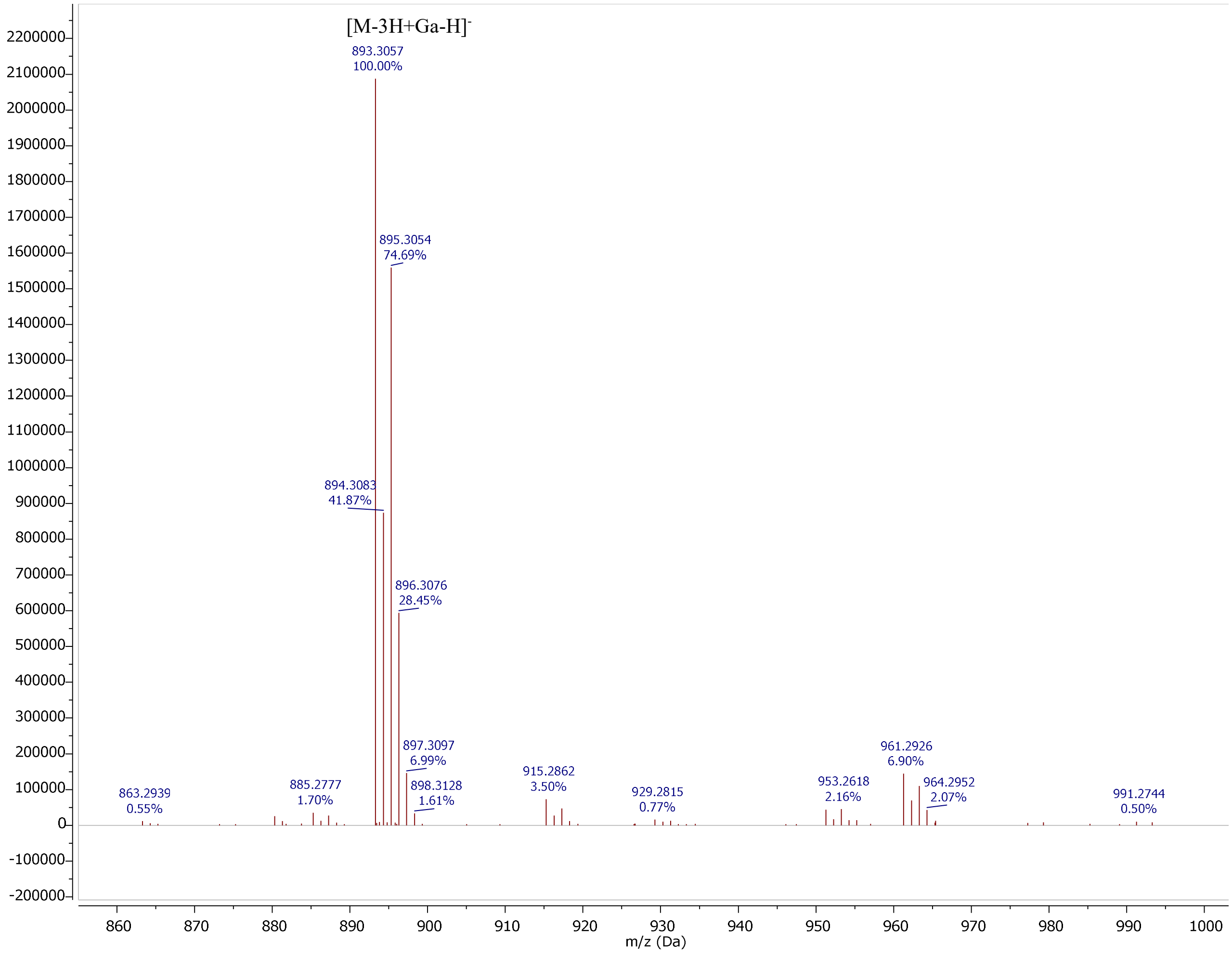

Figure S90. (-) HRMS-ESI of *N,N'*-diacetyl-*Z*-L-fusarinine B Ga^{3+} complex (**7-Ga**)

Table S21. NMR chemical shifts of *N,N'*-diacetyl-*Z*-L-fusarinine B Ga^{3+} complex (**7-Ga**) in CD_3OD (500 MHz)

**Position**	**δ_C [a] mult**	**δ_H [b] mult (*J* in Hz)**	**∫**	**HMBC (H→C#)**	**COSY (H→H#)**
**2**	55.41, CH	4.05, t (6.0)	1		4
**2' 2''**	52.87, CH	4.39, t (5.9)	2		4' 4''
**3 3' 3''**	171.72, C				
**4 4' 4''**	28.54, CH_2	1.85 - 1.74, m<br>2.05 - 2.02, m	3<br>3		5 5' 5''
	29.30, CH_2<br>29.94, CH_2	1.85 - 1.74, m<br>2.05 - 2.02, m			
**5 5' 5''**	21.40, CH_2	1.85 - 1.74, m<br>2.05 - 2.02, m	3<br>3		4 4' 4'', 6 6' 6''
	22.31, CH_2	1.85 - 1.74, m<br>2.05 - 2.02, m			
**6 6' 6''**	51.41, CH_2<br>51.28, CH_2	3.30 - 3.18, m<br>4.01 - 3.83, m	4<br>2		5 5' 5''
**8 8' 8''**	161.12, C<br>162.13, C				
**9 9' 9''**	116.53, CH<br>113.73, CH	6.18, s<br>6.22, s	1<br>2	8 8' 8'', 16 16' 16''	16 16' 16''
**10 10'10''**	153.79, C				
**11 11'**	32.65, CH_2	3.43 - 3.32, m	2		12 12'
	34.12, CH_2	3.43 - 3.32, m<br>3.30 - 3.18, m	1<br>1		
**11''**	38.34, CH_2	3.30 - 3.18, m<br>3.18 - 2.46, m	1<br>1		12''
**12 12'**	65.78, CH_2<br>67.63, CH_2	4.29 - 4.10, m	4		11 11'
**12''**	61.59, CH_2	3.83 - 3.43, m	2		11''
**14' 14''**	172.96, C<br>173.66, C				
**15' 15''**	22.29, CH_3<br>22.35, CH_3	2.00, s<br>2.01, s		14' 14''	
**16 16' 16''**	24.07, CH_3<br>24.39, CH_3	1.97, d (1.4)	9	8 8' 8'', 9 9' 9''<br>10 10'10'', 11 11'	9 9' 9''

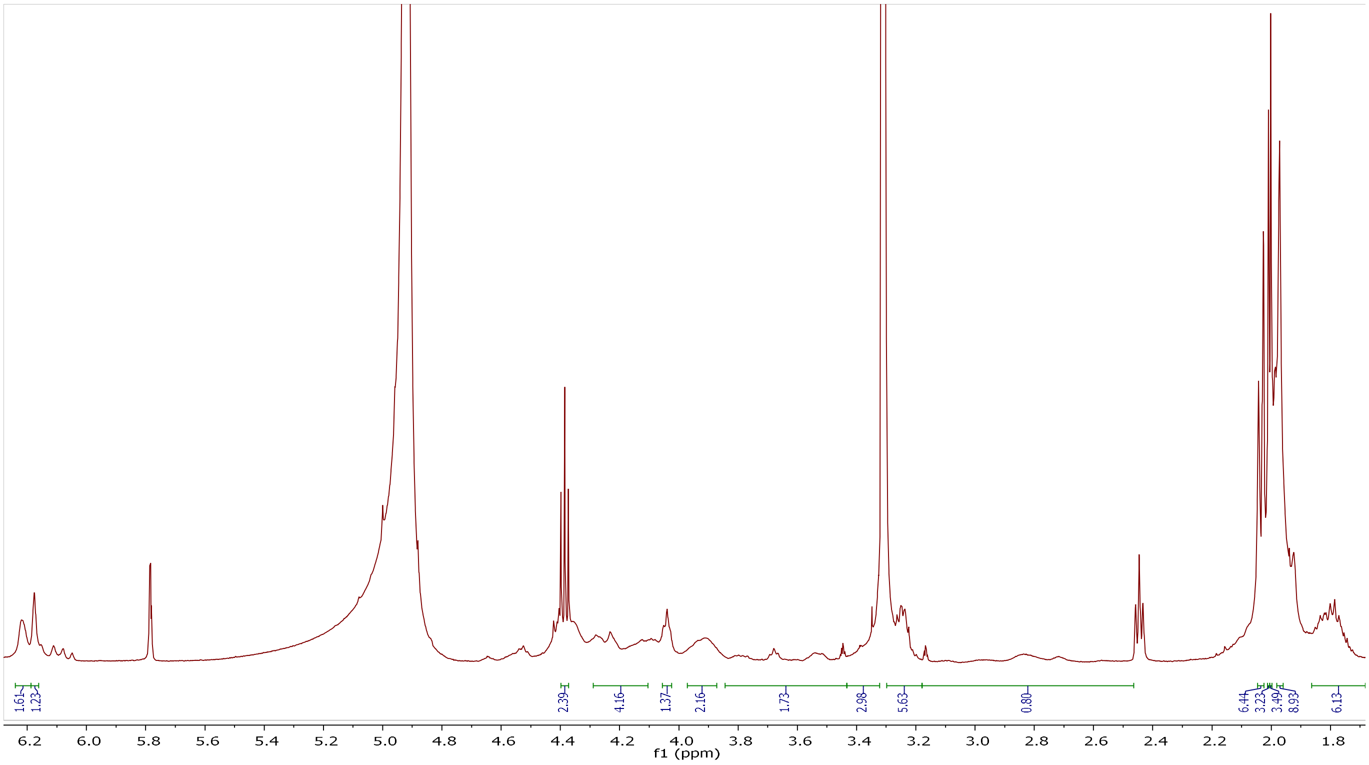

Figure S91. ^{1}H-NMR spectrum of *N,N'*-diacetyl-*Z*-L-fusarinine B Ga^{3+} complex (**7-Ga**) in CD_3OD (500 MHz)

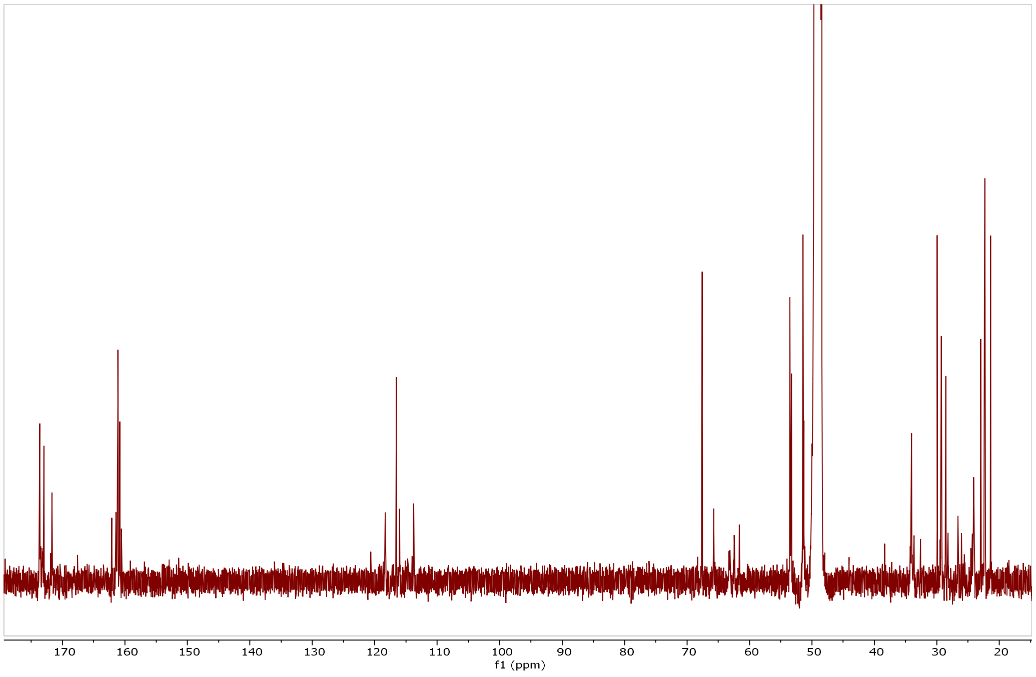

Figure S92. ^{13}C-NMR spectrum of *N,N'*-diacetyl-*Z*-L-fusarinine B Ga^{3+} complex (**7-Ga**) in CD_3OD (125 MHz)

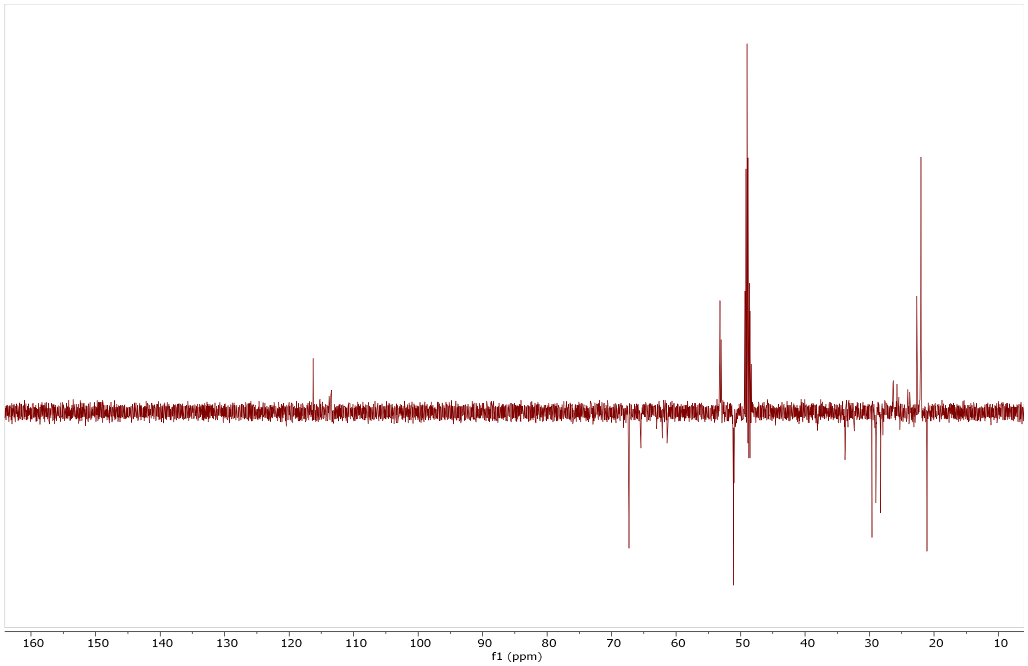

Figure S93. DEPT135-NMR spectrum of *N,N'*-diacetyl-*Z*-L-fusarinine B Ga^{3+} complex (**7-Ga**) in CD_3OD (125 MHz)

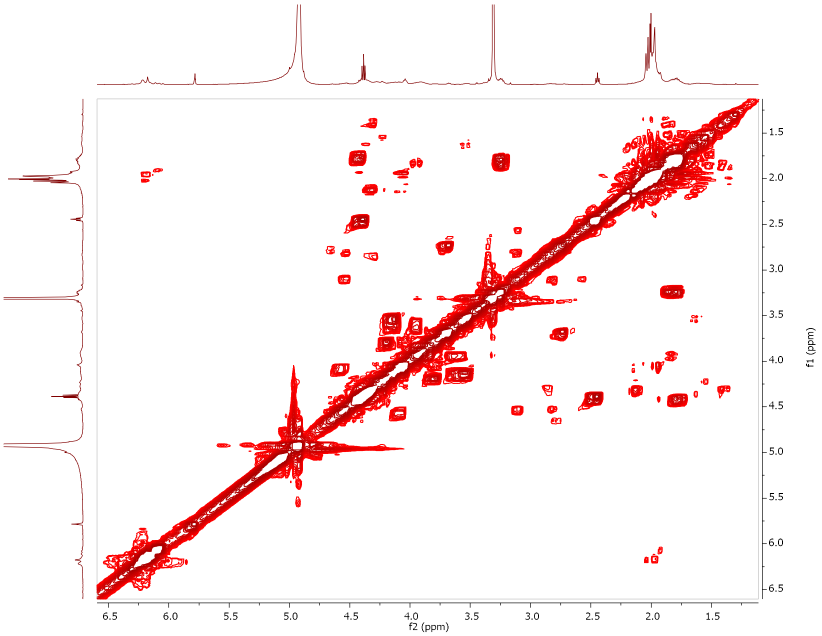

Figure S94. 2D-COSY-NMR spectrum of *N,N'*-diacetyl-*Z*-L-fusarinine B Ga^{3+} complex (**7-Ga**) in CD_3OD

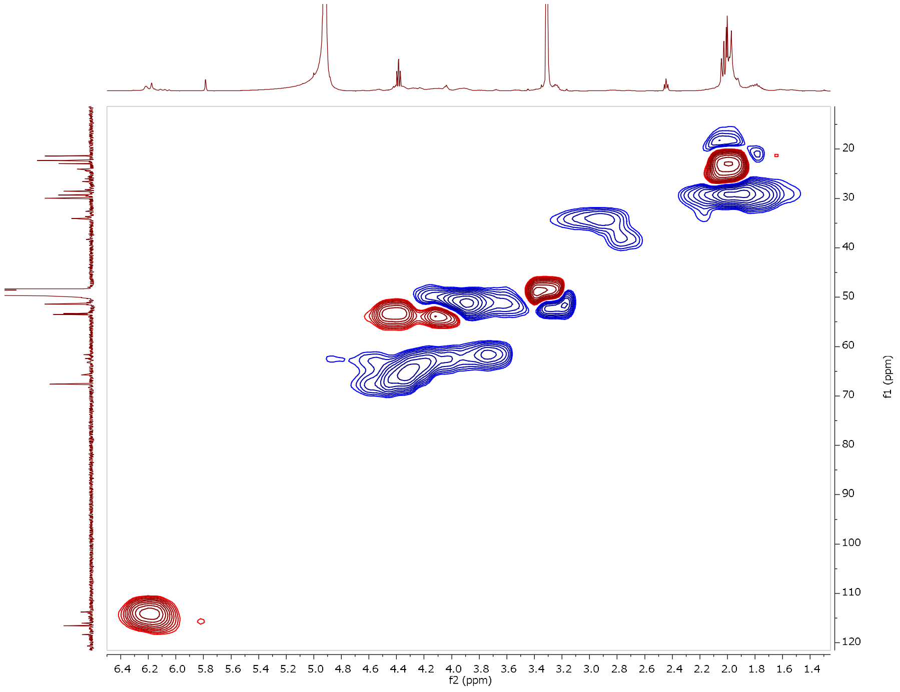

Figure S95. 2D-HSQC-NMR spectrum of *N,N'*-diacetyl-*Z*-L-fusarinine B Ga^{3+} complex (**7-Ga**) in CD_3OD

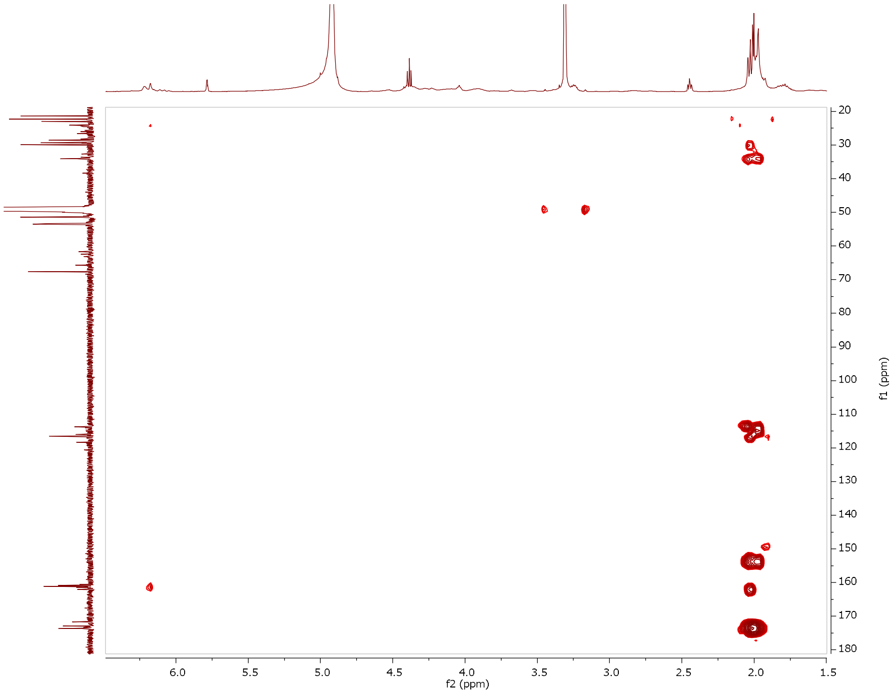

Figure S96. 2D-HMBC-NMR spectrum of *N,N'*-diacetyl-*Z*-L-fusarinine B Ga^{3+} complex **(7-Ga)** in CD_3OD

Table S22. NMR chemical shifts of N,N'-diacetyl-Z-L-fusarinine B Ga^{3+} complex (**7-Ga**) in $(CD_3)_2SO$ (500 MHz)

Position	δ_C [a] mult	δ_H [b] mult (*J* in Hz)	∫	HMBC (H→C#)	COSY (H→H#)
**1**		8.26 - 8.22, m	2		2
**1' 1''**		8.26 - 8.22, m<br>8.37 - 8.34, m	1<br>1		2' 2''
**2**	51.82, CH	3.93 - 3.91, m	1		
**2' 2''**	51.59, CH	4.23 - 4.17, m	2		
**3 3' 3''**	171.14, C<br>171.97, C				
**4 4' 4''**	27.75, CH_2	1.71 - 1.43, m<br>1.82 - 1.71, m	3<br>3		2 2' 2'', 5 5' 5''
	27.07, CH_2	1.71 - 1.43, m<br>1.82 - 1.71, m			
**5 5' 5''**	20.10, CH_2	1.67 - 1.58, m<br>1.83 - 1.67, m	3<br>3		4 4' 4'', 6 6' 6''
	23.14, CH_2	1.67 - 1.58, m<br>1.83 - 1.67, m			
**6 6' 6''**	49.76, CH_2<br>50.01, CH_2	3.11 - 3.06, m<br>3.78 - 3.76, m	1<br>2		5 5' 5''
**8 8' 8''**	159.90, C				
**9 9' 9''**	115.54, CH	6.30 - 6.03, m			16 16' 16''
**10 10' 10''**	150.86, CH				
**11 11'**	32.46, CH_2	2.55 - 2.53, m<br>2.83 - 2.73, m	1<br>1		12 12'
	32.56, CH_2	2.55 - 2.53, m<br>2.83 - 2.73, m	1<br>1		
**11''**	36.86, CH_2	2.55 - 2.53, m	2		12''
**12 12'**	63.48, CH_2<br>65.82, CH_2	4.09, q (7.1)<br>4.29, t (6.3)	2<br>2		11 11'
**12''**	59.89, CH_2	3.48 - 3.47, m			11''
**14' 14''**	169.83, C				
**15' 15''**	22.33, CH_3<br>22.63, CH_3	1.84, brs<br>1.86, brs	3<br>3	14' 14''	
**16 16' 16''**	25.64, CH_3<br>25.69, CH_3	1.86, d (1.5)<br>1.89 - 1.87, m<br>1.96 - 1.93, m	3<br>3<br>3	8 8' 8'', 9 9' 9''<br>10 10'10''	9 9' 9''

Figure S97. ^{1}H-^{1}H COSY (▬) and ^{1}H-^{13}C HMBC (→) correlations of *N,N'*-diacetyl-*Z*-L-fusarinine B Ga^{3+} complex (**7-Ga**) in $(CD_3)_2SO$

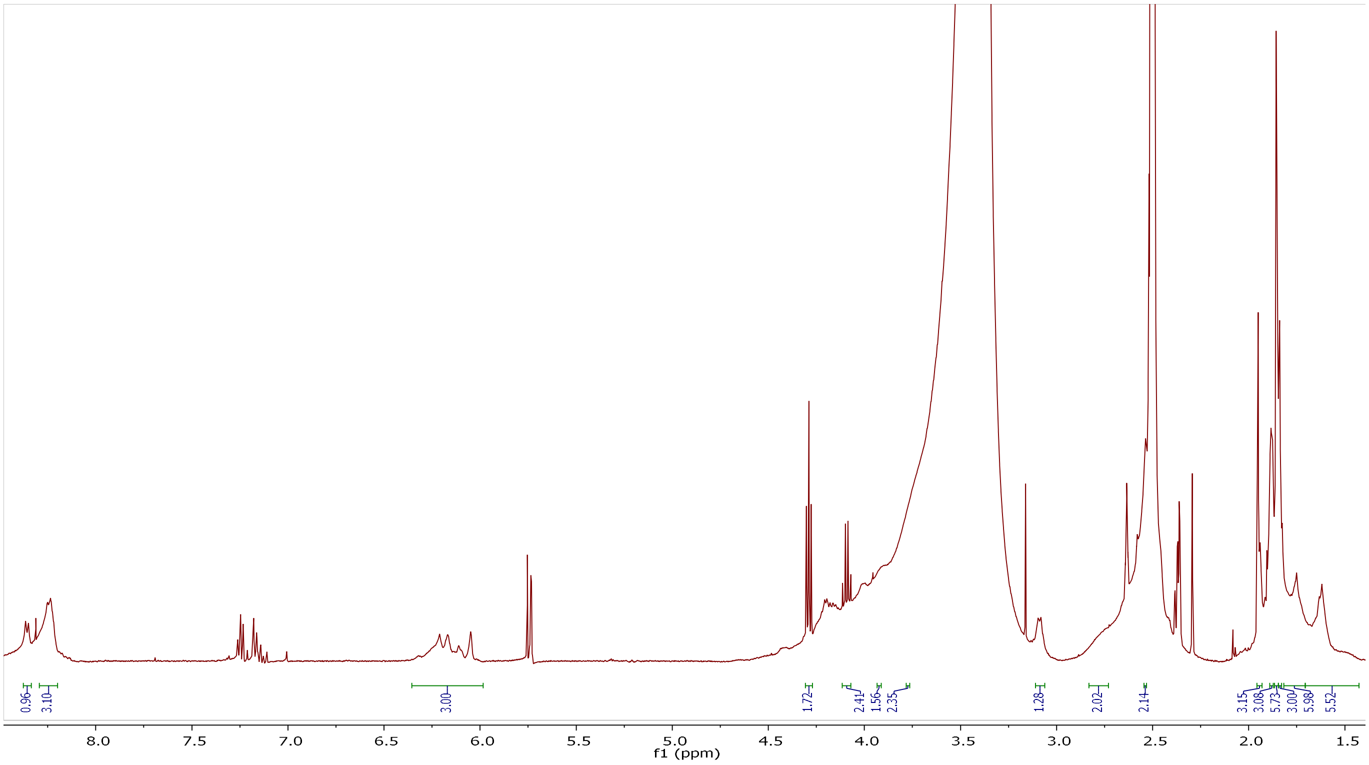

Figure S98. ^{1}H-NMR spectrum of *N,N'*-diacetyl-*Z*-L-fusarinine B Ga^{3+} complex (**7-Ga**) in $(CD_3)_2SO$ (500 MHz)

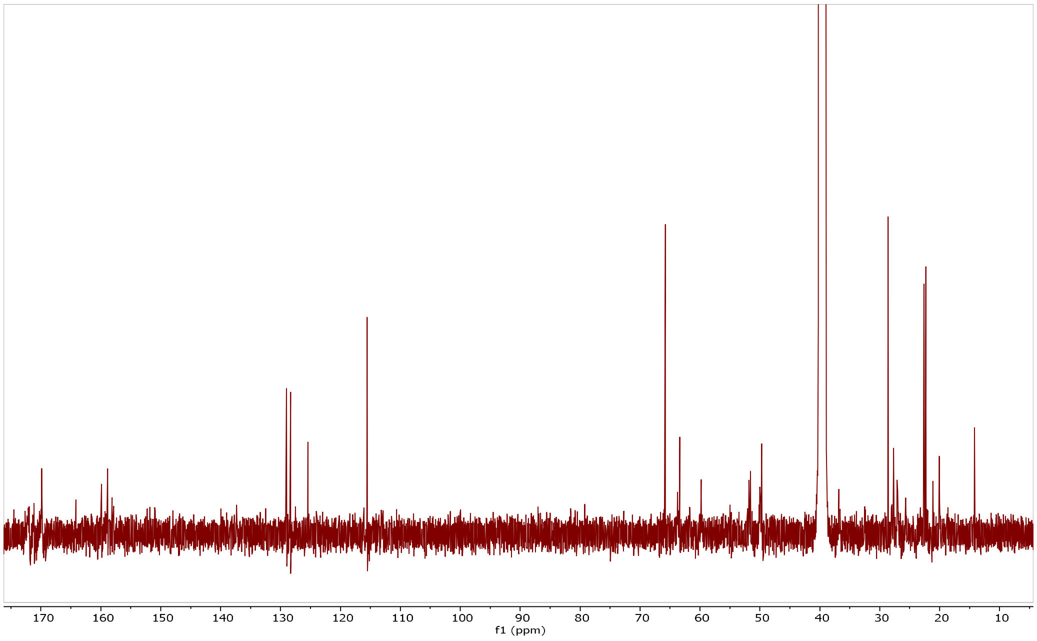

Figure S99. ^{13}C-NMR spectrum of *N,N'*-diacetyl-*Z*-L-fusarinine B Ga^{3+} complex (**7-Ga**) in $(CD_3)_2SO$ (125 MHz)

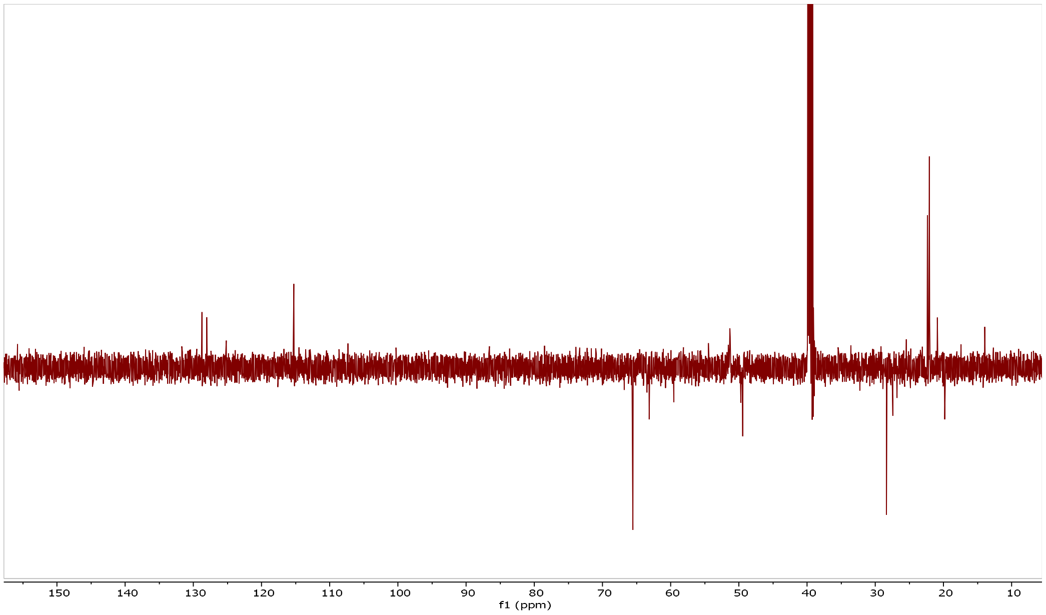

Figure S100. DEPT-NMR spectrum of *N,N'*-diacetyl-*Z*-L-fusarinine B Ga^{3+} complex (**7-Ga**) in $(CD_3)_2SO$ (125 MHz)

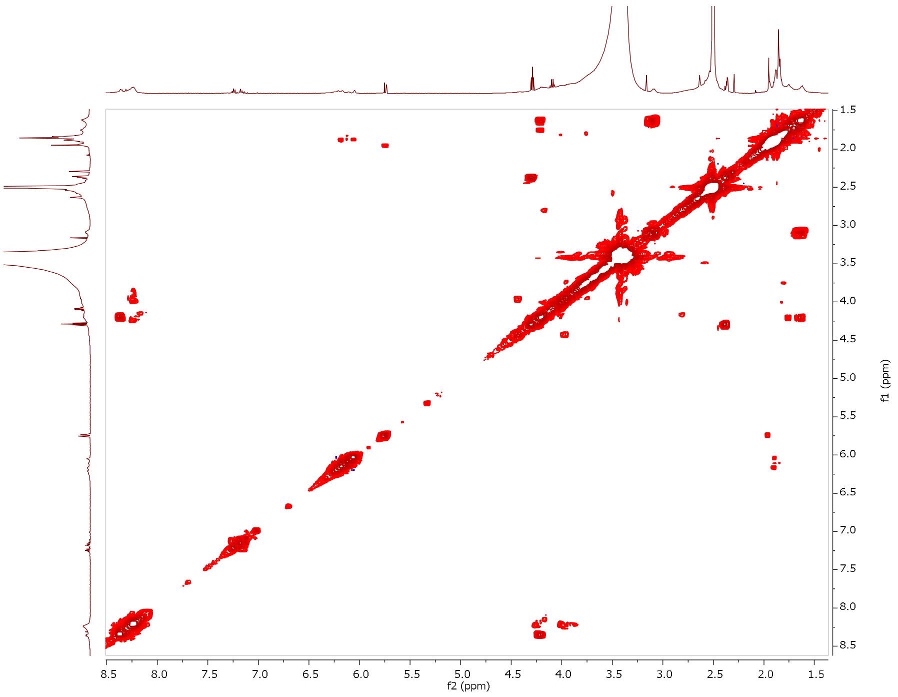

Figure S101. 2D-COSY-NMR spectrum of *N,N'*-diacetyl-*Z*-L-fusarinine B Ga^{3+} complex (**7-Ga**) in $(CD_3)_2SO$

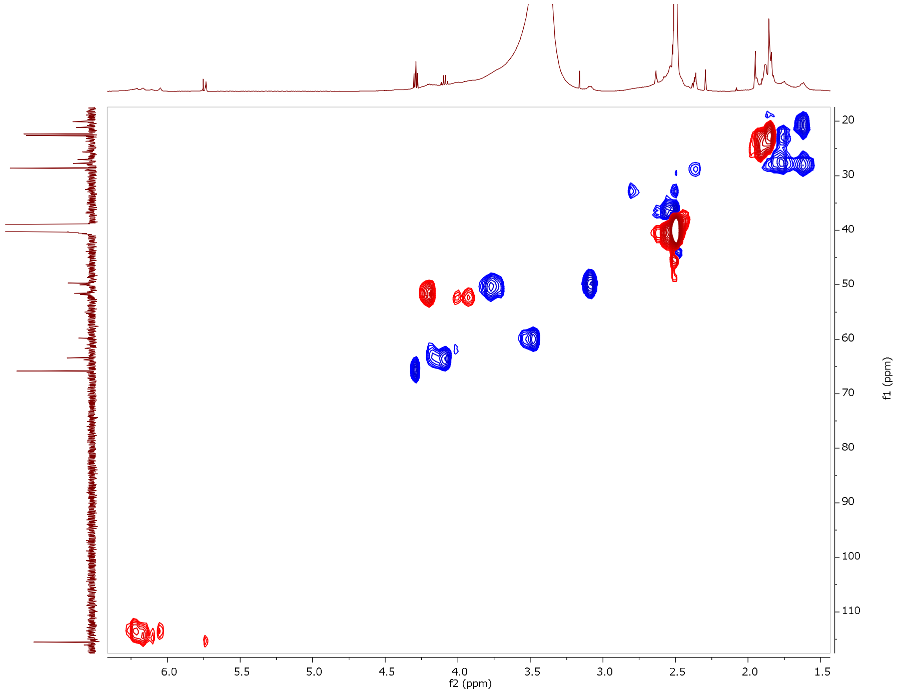

Figure S102. 2D-HSQC-NMR spectrum of *N,N'*-diacetyl-*Z*-L-fusarinine B Ga^{3+} complex (**7-Ga**) in $(CD_3)_2SO$

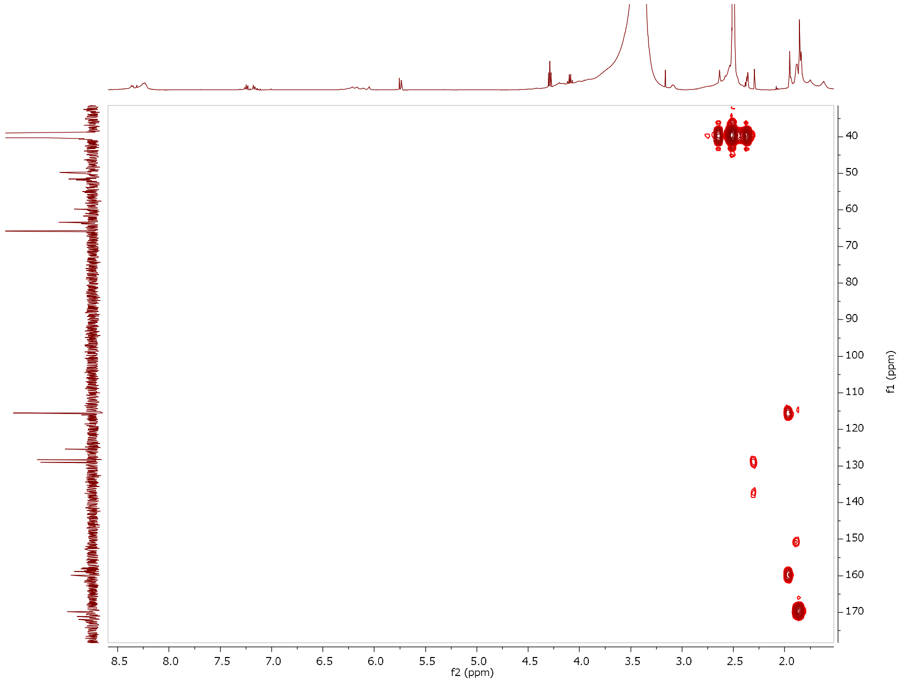

Figure S103. 2D-HMBC-NMR spectrum of *N,N'*-diacetyl-*Z*-L-fusarinine B Ga^{3+} complex (**7-Ga**) in $(CD_3)_2SO$

N,N'-diacetyl-Z-L-fusarinine B (7)

Table S23. Major m/z ions in the (+) and (-) HRMS-ESI of N,N'-diacetyl-Z-L-fusarinine B (**7**)

Adduct	Observed m/z	Theoretical m/z	Error [ppm]
$[M+H]^+$	829.4190	829.4195	0.6
$[M+Na]^+$	851.4011	851.4015	0.5
$[MCOONa+Na]^+$	873.3830	873.3835	0.6
$[M-H]^-$	827.4034	827.4044	1.2

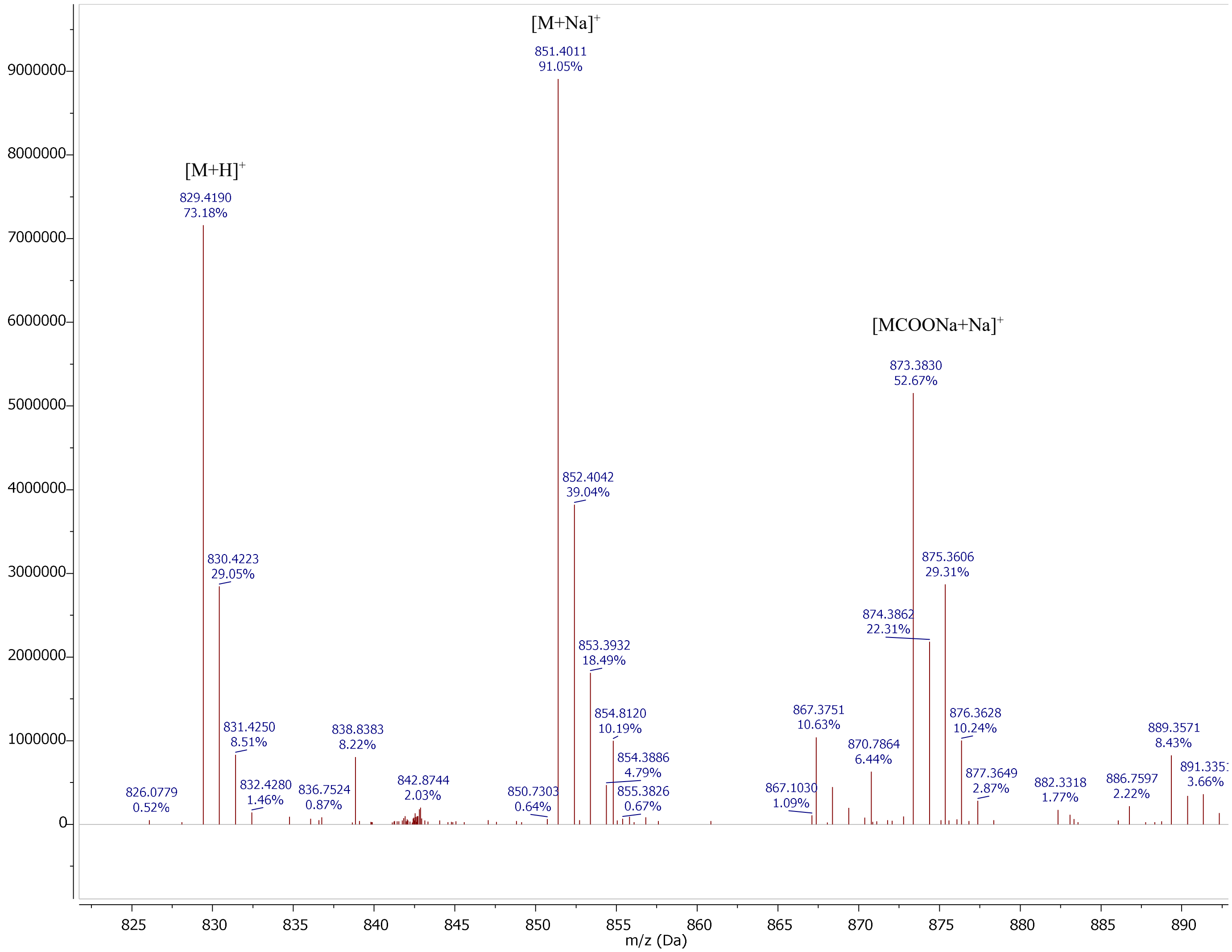

Figure S104. (+) HRMS-ESI of N,N'-diacetyl-Z-L-fusarinine B (**7**)

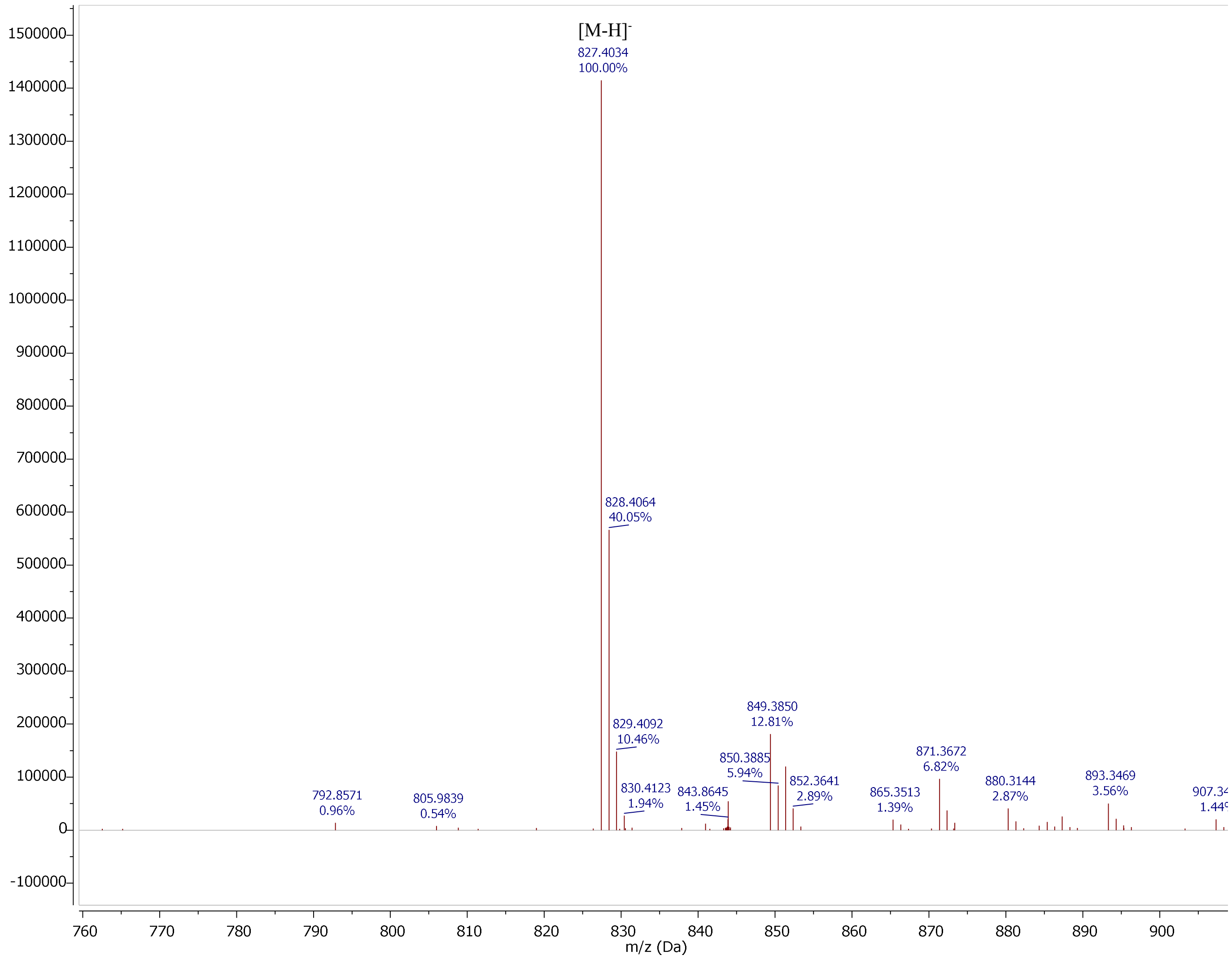

Figure S105. (-) HRMS-ESI of *N,N'*-diacetyl-*Z*-L-fusarinine B (**7**)

N-acetyl- *Z*-L-fusarinine A Ga^{3+} complex (8-Ga)

Table S24. Major *m/z* ions in the (+) and (-) HRMS-ESI of *N*-acetyl-*Z*-L-fusarinine A Ga^{3+} complex (**8-Ga**)

Adduct	Observed *m/z*	Theoretical *m/z*	Error [ppm]
$[M\text{-}2H\text{+}^{69/71}Ga]^+$	611.1838/613.1830	611.1844/613.1835	1.0/0.8
$[MCOONa\text{-}2H\text{+}^{69/71}Ga]^+$	633.1657/635.1665	633.1664/635.1655	1.1/1.6
$[M\text{-}3H\text{+}^{69/71}Ga\text{-}H]^-$	609.1692/611.1684	609.1688/611.1679	0.7/0.8

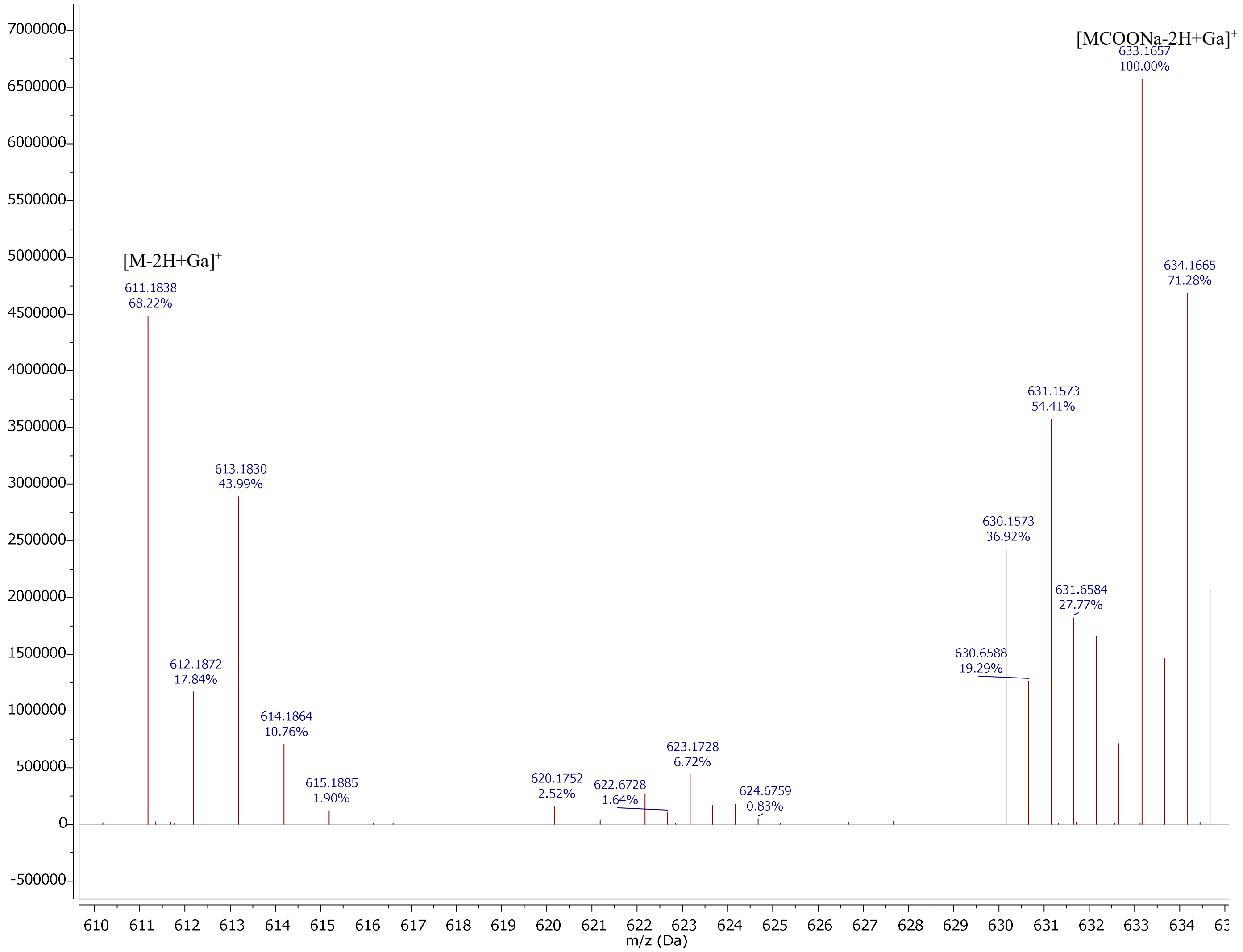

Figure S106. (+) HRMS-ESI of *N*-acetyl-*Z*-L-fusarinine A Ga^{3+} complex (**8-Ga**)

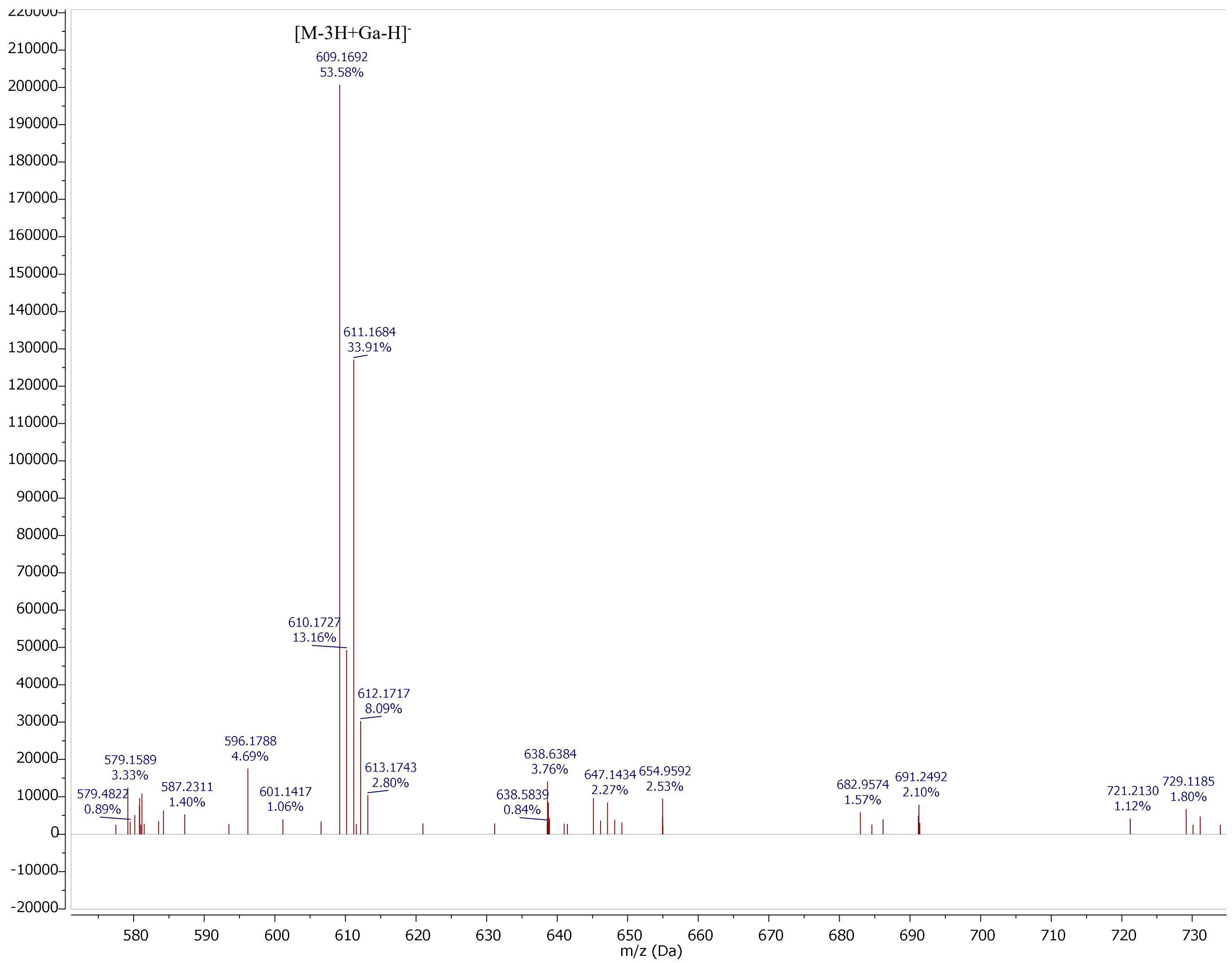

Figure S107. (-) HRMS-ESI of *N*-acetyl-*Z*-L-fusarinine A Ga^{3+} complex (**8-Ga**)

Table S25. NMR chemical shifts of *N*-acetyl-*Z*-L-fusarinine A Ga^{3+} complex (**8-Ga**) in CD_3OD (500 MHz)

Position	δ_C [a] mult	δ_H [b] mult (*J* in Hz)	∫	HMBC (H→C#)	COSY (H→H#)
**2**	53.60,CH	3.89 - 3.86, m	1		4
**2'**	52.67, CH	4.44 - 4.40, m	1		4'
**3**	173.58, C				
**3'**	173.26, C				
**4 4'**	28.88, CH_2<br>29.24, CH_2	1.77 - 1.60, m	4		2 2', 5 5'
**5 5'**	24.38, CH_2	1.77 - 1.60, m	4		4 4', 6 6'
**6**	50.59, CH_2	3.64 - 3.61, m<br>3.82 - 3.78, m	1<br>1		5
**6'**	50.73, CH_2	3.56 - 3.54, m<br>3.89 - 3.86, m	1<br>1		5'
**8**	160.63, C				
**8'**	161.18, C				
**9**	116.78, CH	6.03, s	1	8, 11, 16	16
**9'**	113.01, CH	6.10, s	1	8', 11', 16'	16'
**10**	149.29, C				
**10'**	154.07, C				
**11**	34.46, CH_2	2.43 - 2.37, m<br>2.91 - 2.87, m	1<br>1		12
**11'**	39.24, CH_2	2.62 - 2.59, m<br>2.73 - 2.70, m	1<br>1		12'
**12**	62.61, CH_2	3.95 - 4.00, m<br>4.61 - 4.55, m	1<br>1		11
**12'**	61.06, CH_2	3.48 - 3.47, m<br>3.53 - 3.51, m	1<br>1		11'
**14'**	173.59, C				
**15'**	22.44, CH_3	2.00, s	3	14'	
**16**	23.07, CH_3	1.93, m	3	9, 10, 11	9
**16'**	26.43, CH_3	1.90, m	3	9', 10', 11'	9'

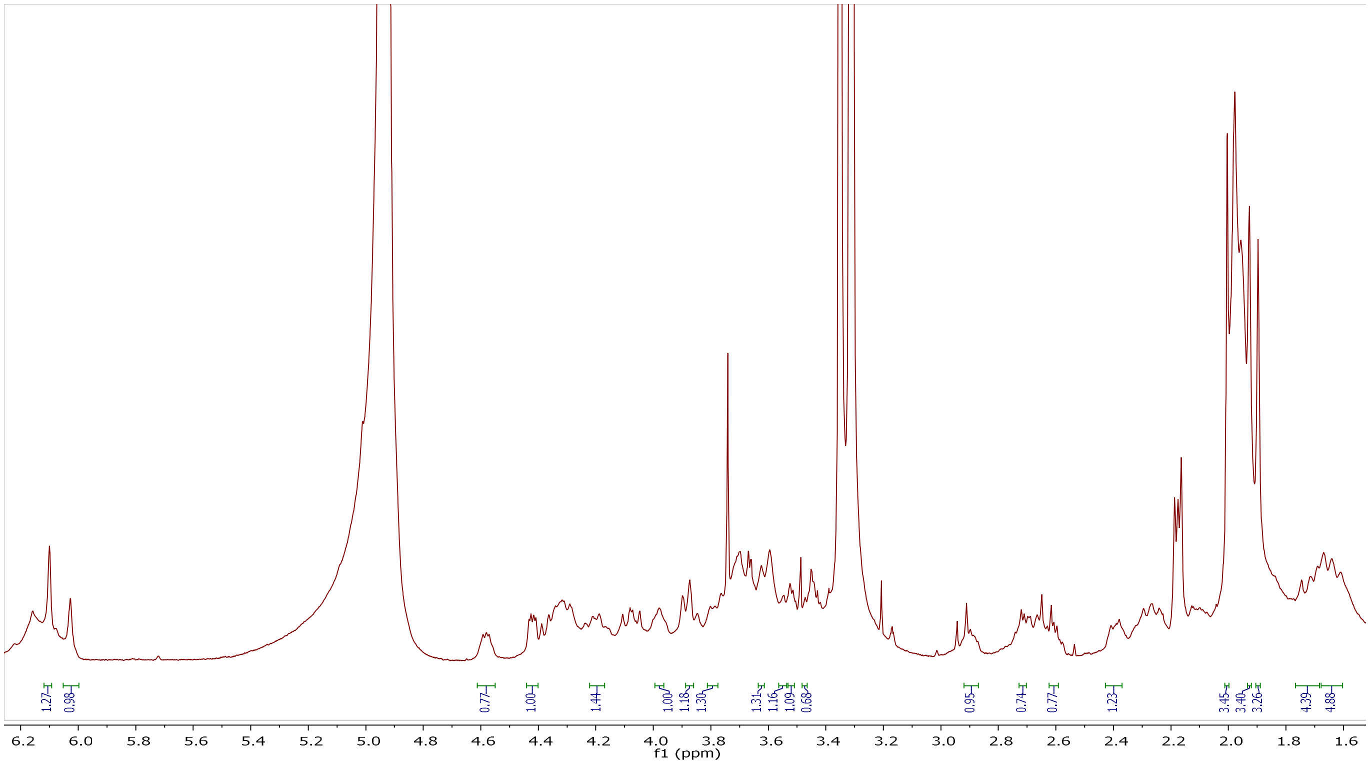

Figure S108. ^{1}H-NMR spectrum of *N*-acetyl-*Z*-L-fusarinine A Ga^{3+} complex (**8-Ga**) in CD_3OD (500 MHz)

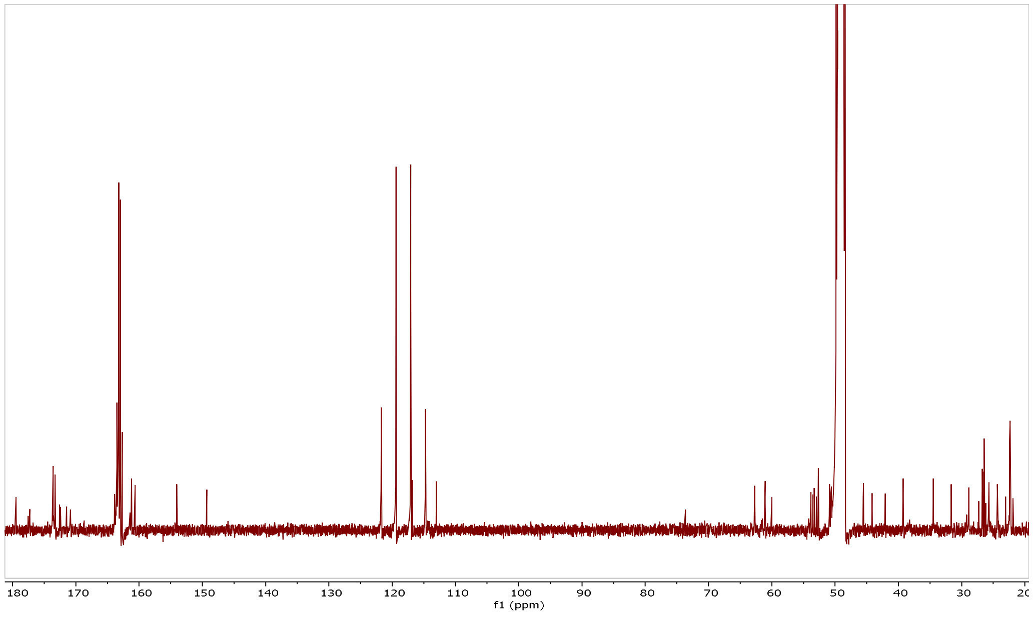

Figure S109. ^{13}C-NMR spectrum of *N*-acetyl-*Z*-L-fusarinine A Ga^{3+} complex (**8-Ga**) in CD_3OD (125 MHz)

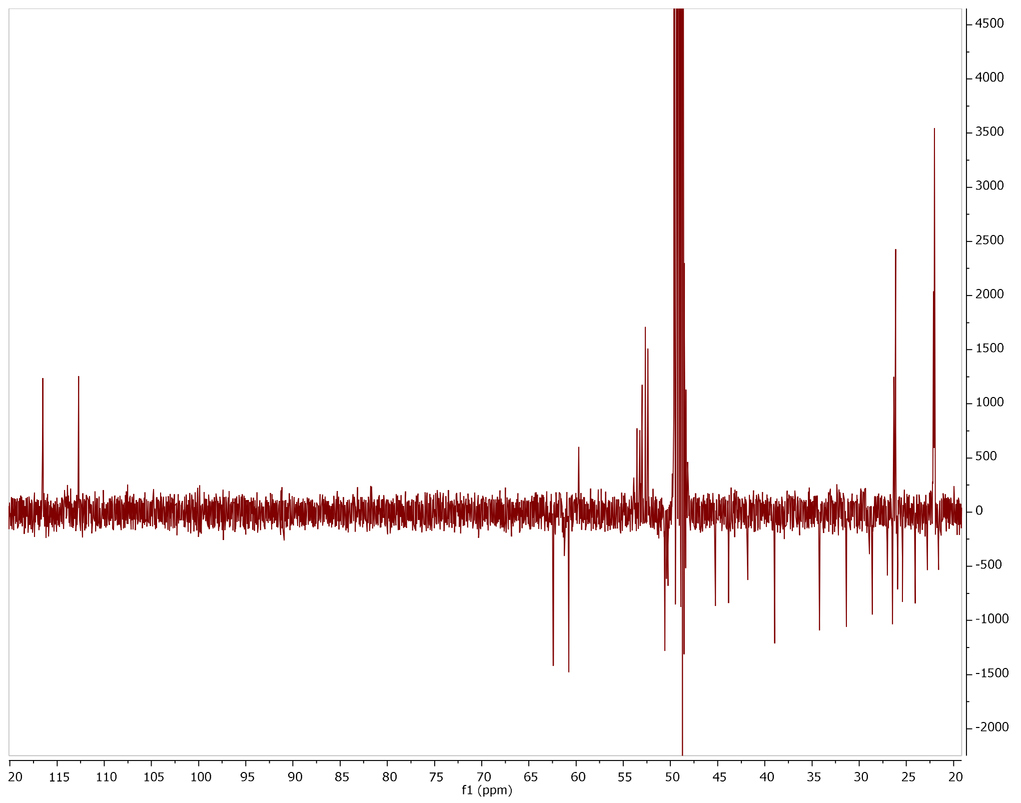

Figure S110. DEPT135-NMR spectrum of *N*-acetyl-*Z*-L-fusarinine A Ga^{3+} complex (**8-Ga**) in CD_3OD (125 MHz)

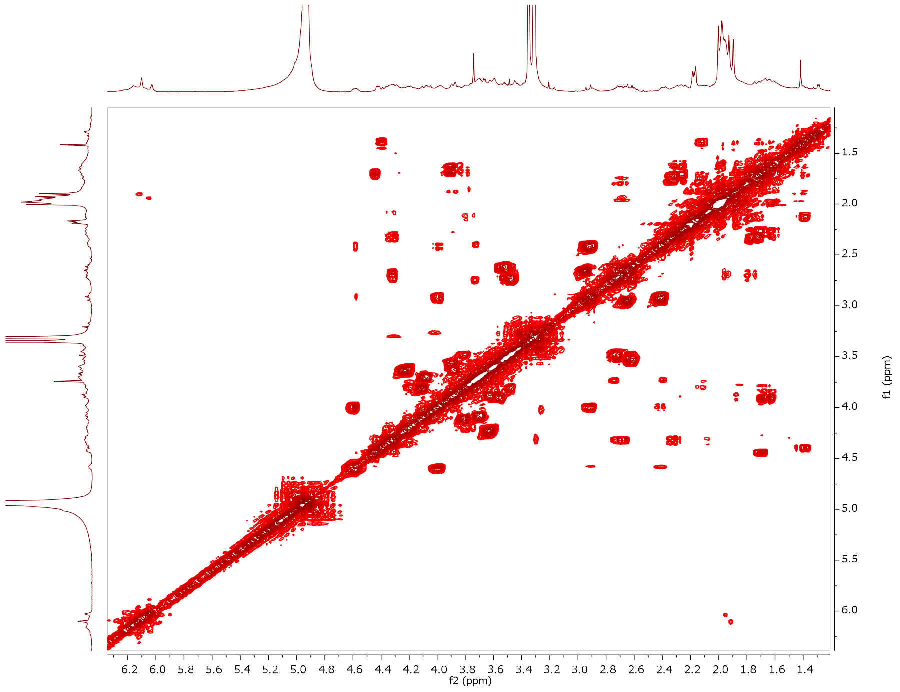

Figure S111. 2D-COSY-NMR spectrum of *N*-acetyl-*Z*-L-fusarinine A Ga^{3+} complex (**8-Ga**) in CD_3OD

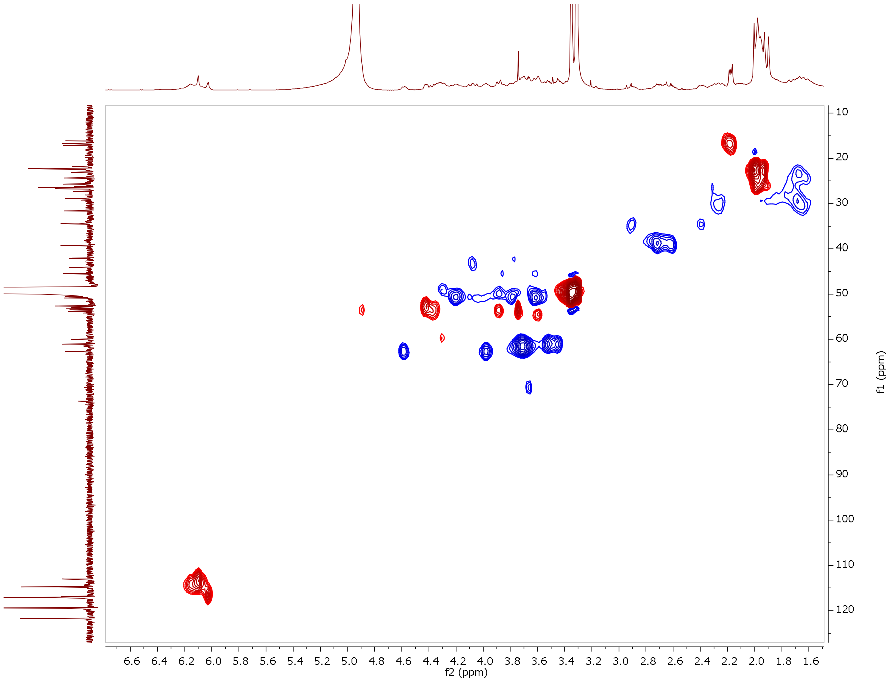

Figure S112. 2D-HSQC-NMR spectrum of *N*-acetyl-*Z*-L-fusarinine A Ga^{3+} complex (**8-Ga**) in CD_3OD

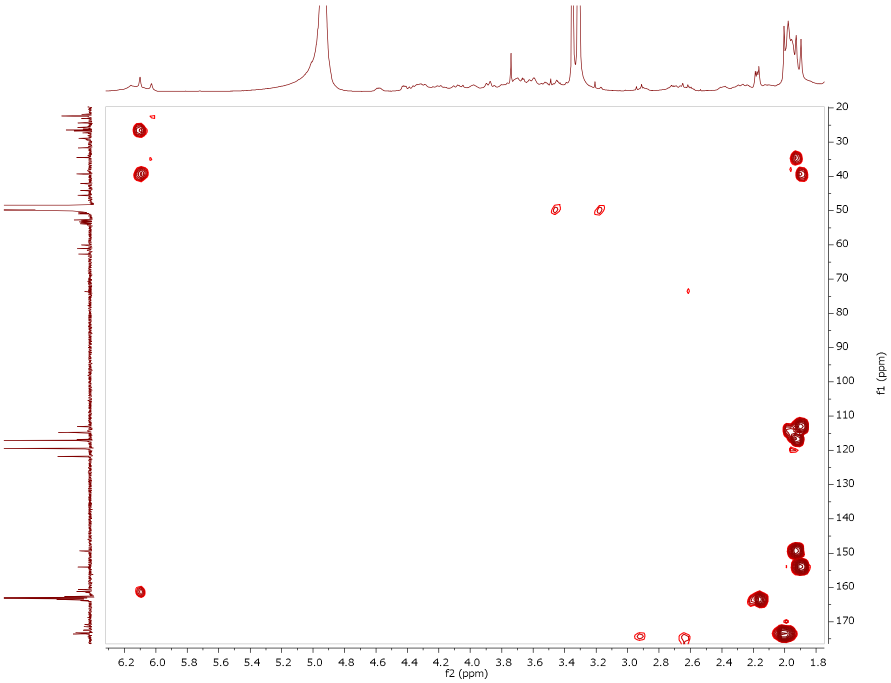

Figure S113. 2D-HMBC-NMR spectrum of *N*-acetyl-*Z*-L-fusarinine A Ga^{3+} complex (**8-Ga**) in CD_3OD

Table S26. NMR chemical shifts of *N*-acetyl-*Z*-L-fusarinine A Ga^{3+} complex (**8-Ga**) in $(CD_3)_2SO$ (500 MHz)

Position	δ_C [a] mult	δ_H [b] mult (*J* in Hz)	∫	HMBC (H→C#)	COSY (H→H#)
**1'**		8.29 - 8.17, m	1		2'
**2**	51.74, CH	3.58 - 3.57, m	1	3	4
**2'**	51.19, CH	4.27 - 4.22, m	1		1', 4'
**3**	174.74, C				
**3'**	171.96, C				
**4 4'**	26.65, CH_2<br>27.97, CH_2	1.66 - 1.48, m	4		5 5'
**5 5'**	23.52, CH_2<br>23.55, CH_2	1.77 - 1.66, m	4		4 4', 6 6'
**6 6'**	48.48, CH_2<br>49.21, CH_2	3.77 - 3.74, m<br>4.12 - 4.06, m	2<br>2		5 5'
**8 8'**	158.61, C				
**9**	115.57, CH	6.12, d (12.3)	1	11	16
**9'**	111.63, CH	6.02, d (14.0)	1	11', 16'	16'
**10**	147.96, C				
**10'**	150.64, C				
**11**	32.87, CH_2	2.33 - 2.22<br>2.90 - 2.80	1<br>1		12
**11'**	37.43, CH_2	2.65 - 2.63, m	2		12'
**12**	63.42, CH_2	4.60 - 4.38, m	2		11
**12'**	59.83, CH_2	3.50 - 3.49	2		11'
**14'**	169.71, C				
**15'**	22.37, CH_3	1.88, s	3	14'	
**16**	24.66, CH_3	1.90 – 1.88, m	3	9, 10, 11	9
**16'**	25.24, CH_3	1.84 - 1.78, m	3	9', 10', 11'	9'

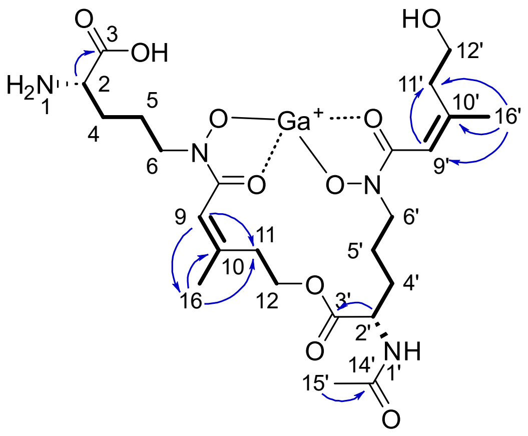

Figure S114. ^{1}H-^{1}H COSY (▬) and ^{1}H-^{13}C HMBC (→) correlations of *N*-acetyl-*Z*-L-fusarinine A Ga^{3+} complex (**8-Ga**) in $(CD_3)_2SO$

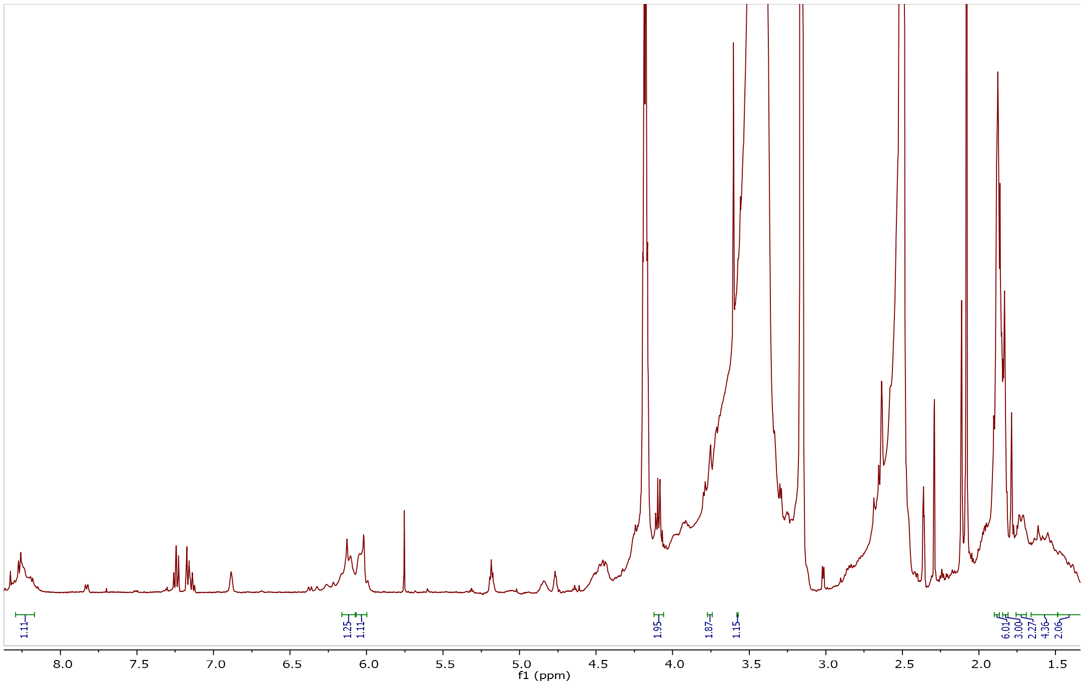

Figure S115. ^{1}H-NMR spectrum of *N*-acetyl-*Z*-L-fusarinine A Ga^{3+} complex (**8-Ga**) in $(CD_3)_2SO$ (500 MHz)

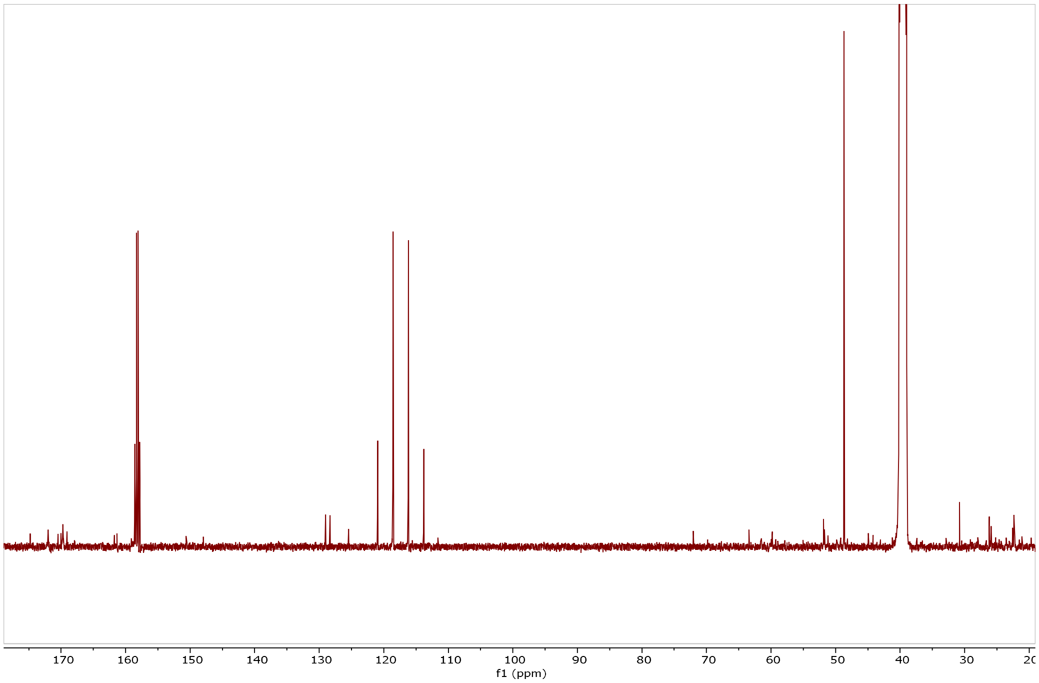

Figure S116. ^{13}C-NMR spectrum of *N*-acetyl-*Z*-L-fusarinine A Ga^{3+} complex (8-Ga) in $(CD_3)_2SO$ (125 MHz)

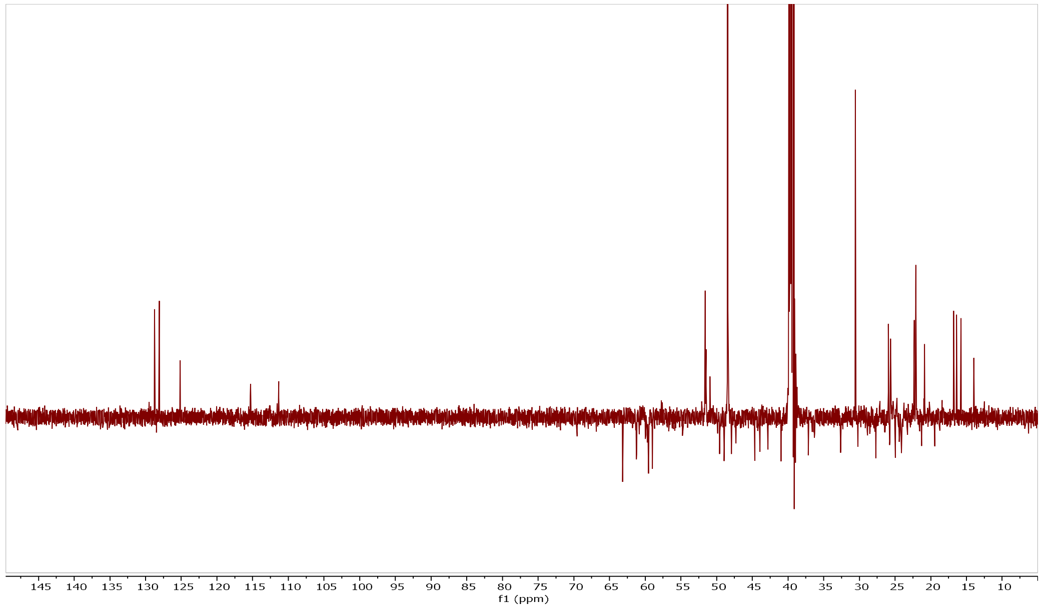

Figure S117. DEPT135-NMR spectrum of *N*-acetyl-*Z*-L-fusarinine A Ga^{3+} complex (**8-Ga**) in $(CD_3)_2SO$ (125 MHz)

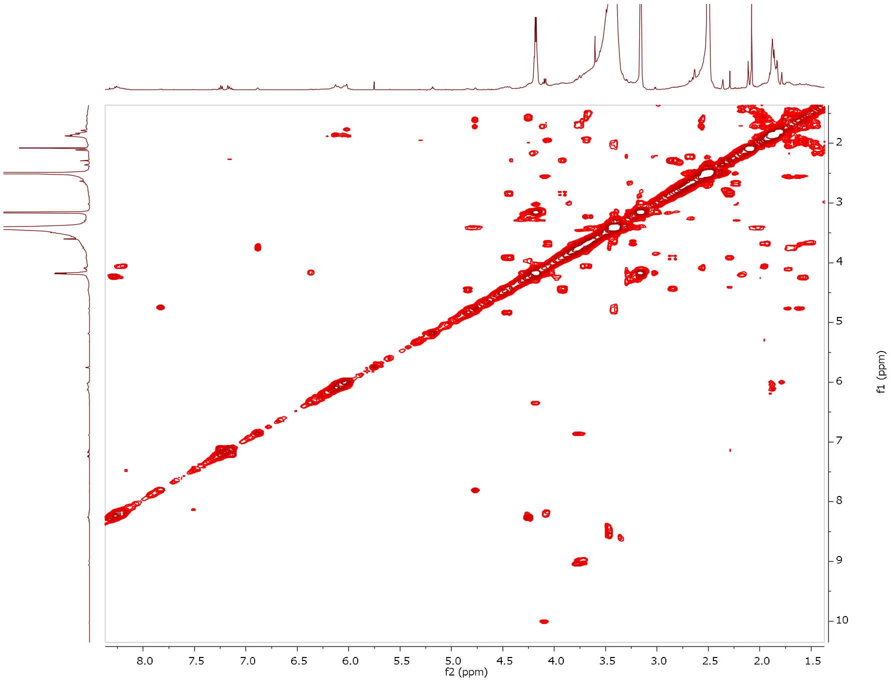

Figure S118. 2D-COSY-NMR spectrum of *N*-acetyl-*Z*-L-fusarinine A Ga^{3+} complex (**8-Ga**) in $(CD_3)_2SO$

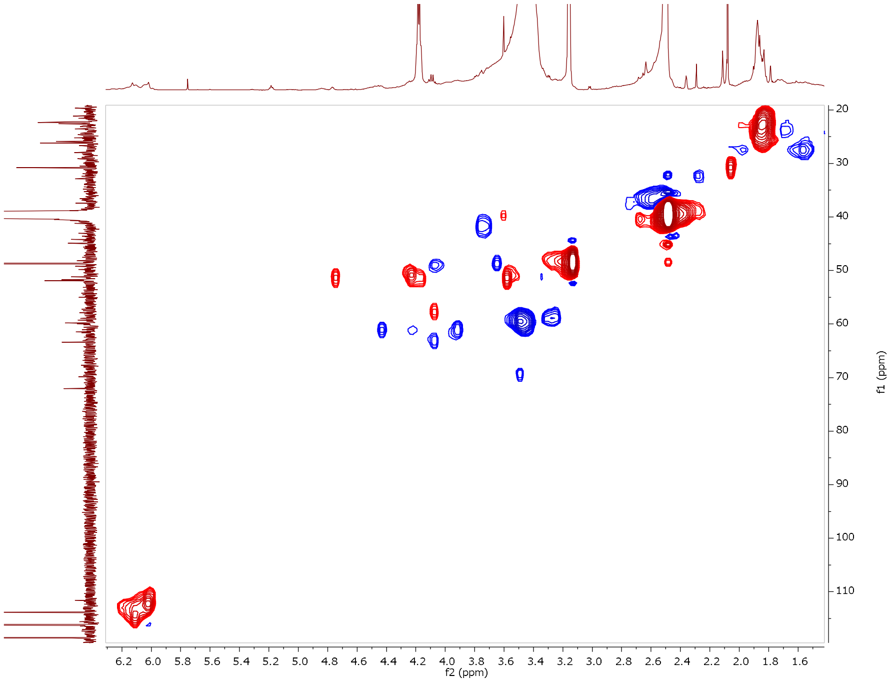

Figure S119. 2D-HSQC-NMR spectrum of *N*-acetyl-*Z*-L-fusarinine A Ga^{3+} complex (**8-Ga**) in $(CD_3)_2SO$

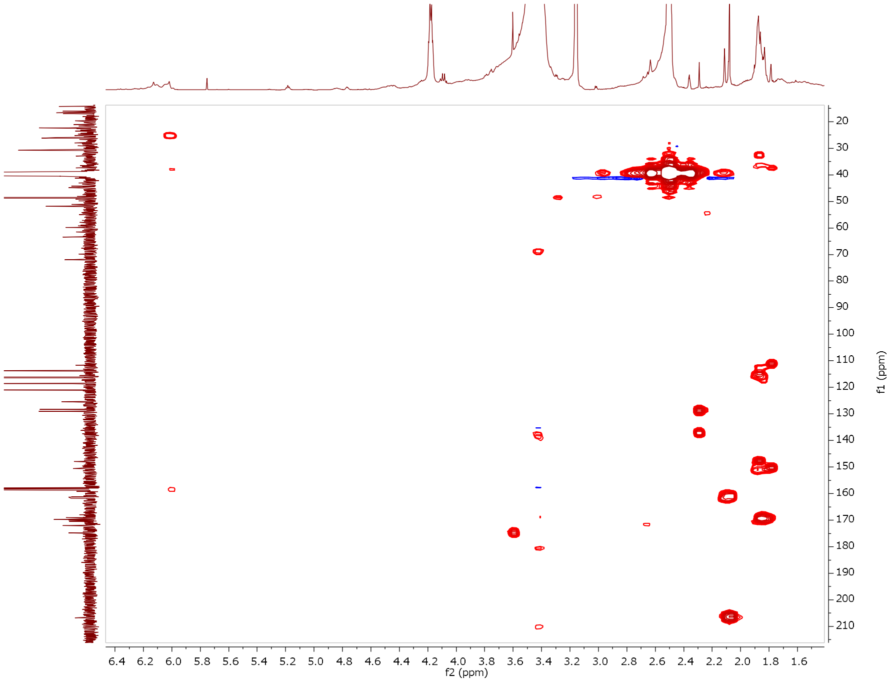

Figure S120. 2D-HMBC-NMR spectrum of *N*-acetyl-*Z*-L-fusarinine A Ga^{3+} complex (**8-Ga**) in $(CD_3)_2SO$

N-acetyl- *Z*-L-fusarinine A (8)

Table S27. Major *m/z* ions in the (+) HRMS-ESI of *N*-acetyl-*Z*-L-fusarinine A (**8**)

Adduct	Observed *m/z*	Theoretical *m/z*	Error [ppm]
$[M+H]^+$	545.2822	545.2822	-
$[M+Na]^+$	567.2641	567.2642	0.2
$[M+K]^+$	583.2381	583.2381	-

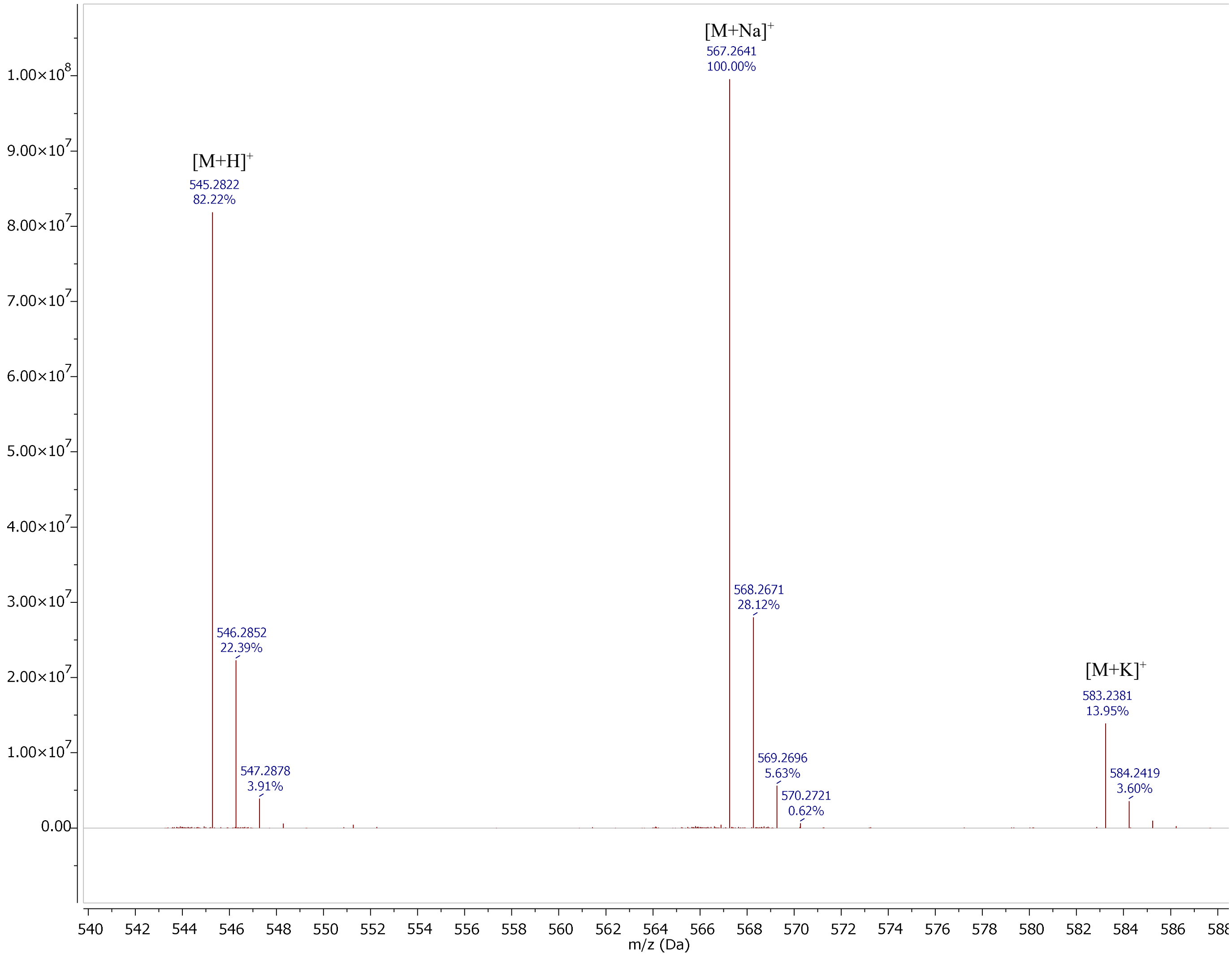

Figure S121. (+) HRMS-ESI of *N*-acetyl-*Z*-L-fusarinine A (**8**)

Table S28. NMR chemical shifts of *N*-acetyl-*Z*-L-fusarinine A (**8**) in CD_3OD (400 MHz)

Position	δ_C [a] mult	δ_H [b] mult (*J* in Hz)	∫	HMBC (H→C#)	COSY (H→H#)
**2**	55.77, CH	3.60, t (5.8)	1	3, 4, 5	4, 5
**2'**	53.72, CH	4.41 - 4.37, m	1	3', 4', 5'	4', 5'
3	174.39, C				
**3**	173.70, C				
**4 4'**	29.49, CH_2	1.89 – 1.75, m	4		2 2',
**5 5'**	24.37, CH_2<br>23.83, CH_2	1.75 – 1.61, m<br>1.89 – 1.75, m	3<br>1		6 6'
**6 6'**	48.04, CH_2	3.69 – 3.62, m	4	4 4'	5 5'
**8 8'**	164.20, C				
**9**	118.94, CH	6.37, s	1		16
**9'**	118.94, CH	6.40, s	1		16'
10	151.96, C				
**10'**	152.65, C				
**11**	33.40, CH_2	2.99 - 2.80, m	2		12
**11'**	37.41, CH_2	2.77 – 2.66, m	2		12'
**12**	64.79, CH_2	4.29, t (6.7)	2	10, 11	11
**12'**	61.33, CH_2	3.71, t (6.5)	2	10', 11'	11'
14'	173.50, C				
**15'**	22.32, CH_3	1.99, s	3	14'	
**16**	25.28 CH_3	1.94, d (1.4)	3	9, 10, 11	9
**16'**	25.28 CH_3	1.95, d (1.4)	3	9', 10', 11'	9'

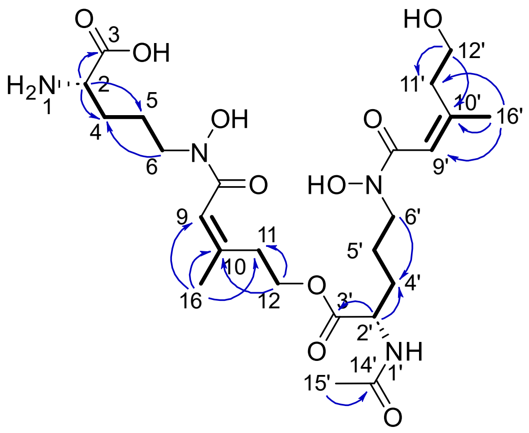

Figure S122. ^{1}H-^{1}H COSY (▬) and ^{1}H-^{13}C HMBC (→) correlations of *N*-acetyl-*Z*-L-fusarinine A (**8**) in CD_3OD

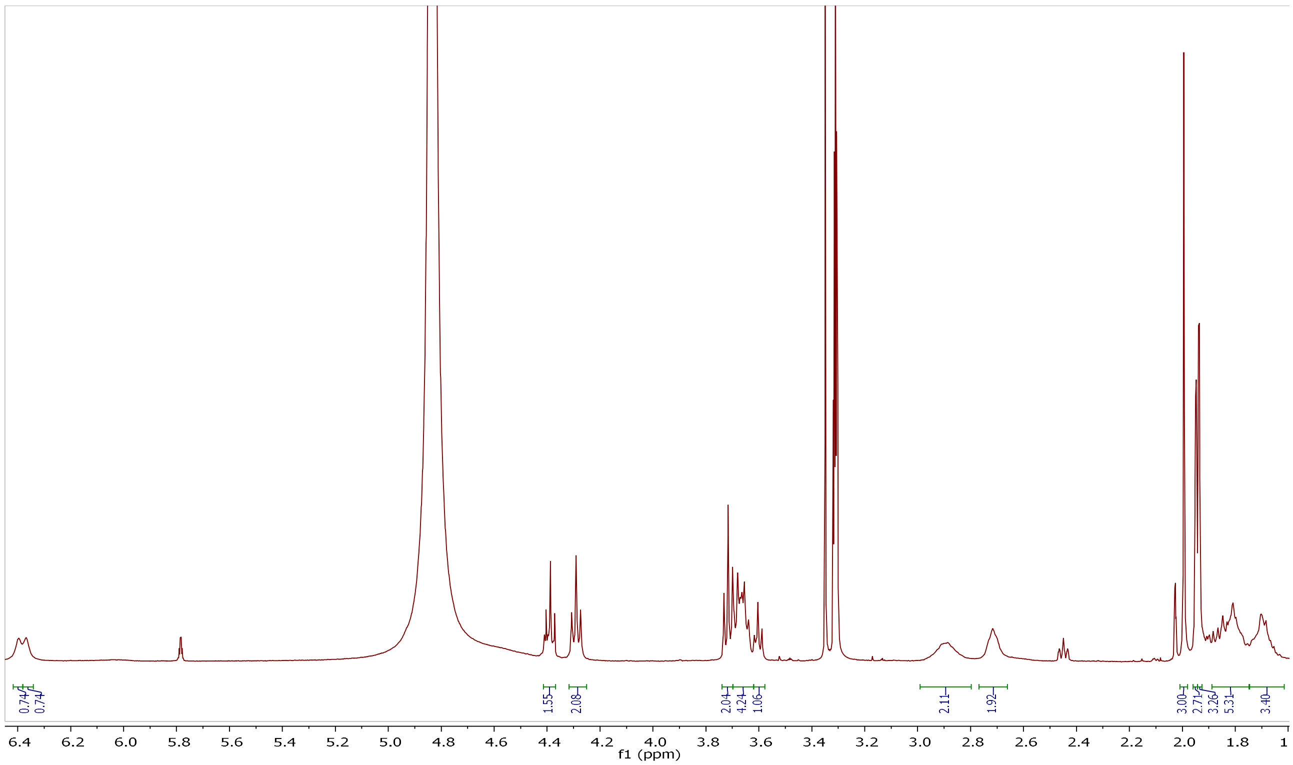

Figure S123. ^{1}H-NMR spectrum of *N*-acetyl-*Z*-L-fusarinine A (**8**) in CD_3OD (400 MHz)

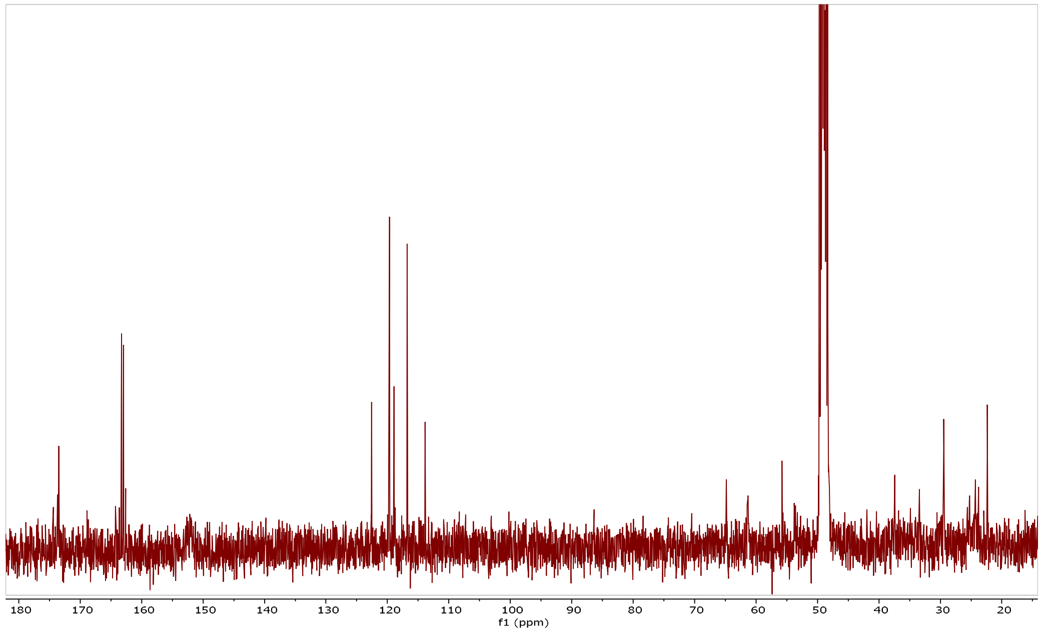

Figure S124. ^{13}C-NMR spectrum of *N*-acetyl-*Z*-L-fusarinine A (**8**) in CD_3OD (100 MHz)

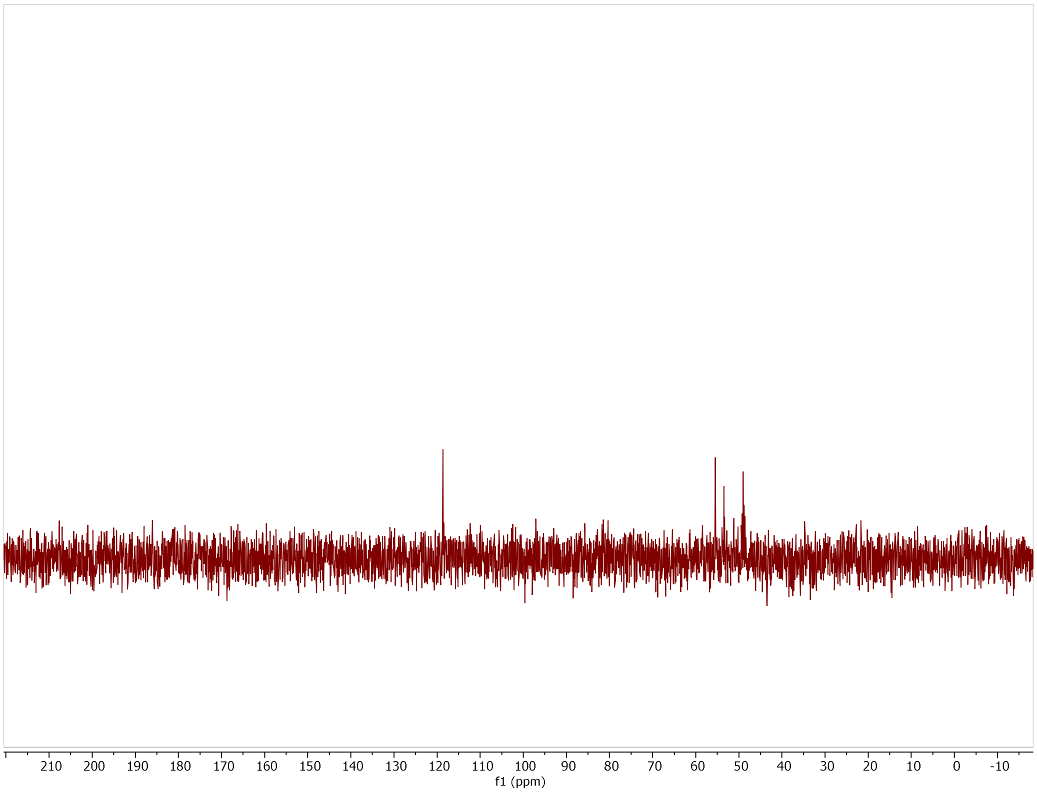

Figure S125. DEPT135-NMR spectrum of *N*-acetyl-*Z*-L-fusarinine A (**8**) in CD_3OD (100 MHz)

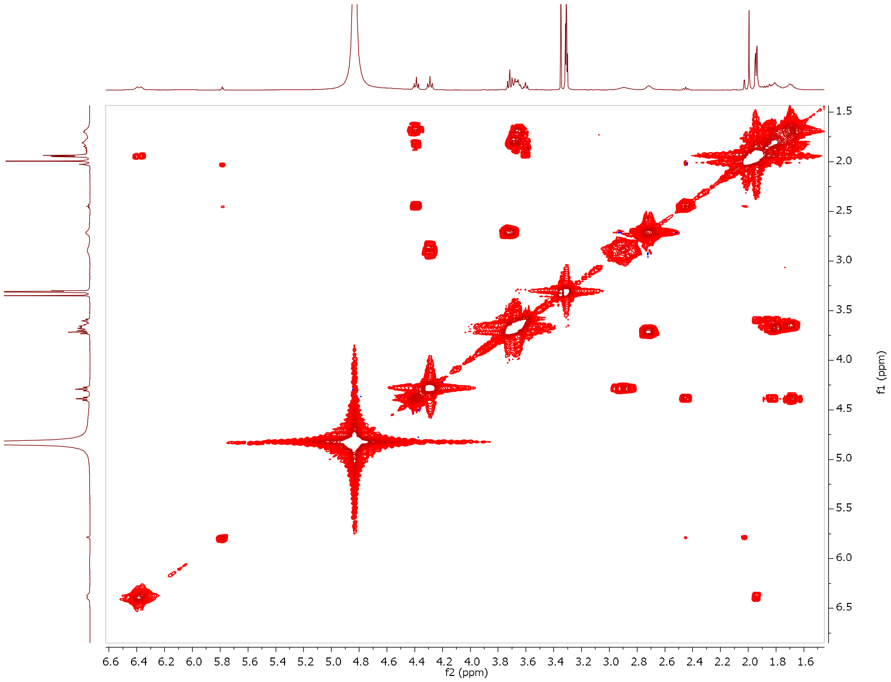

Figure S126. 2D-COSY-NMR spectrum of *N*-acetyl-*Z*-L-fusarinine A (**8**) in CD_3OD

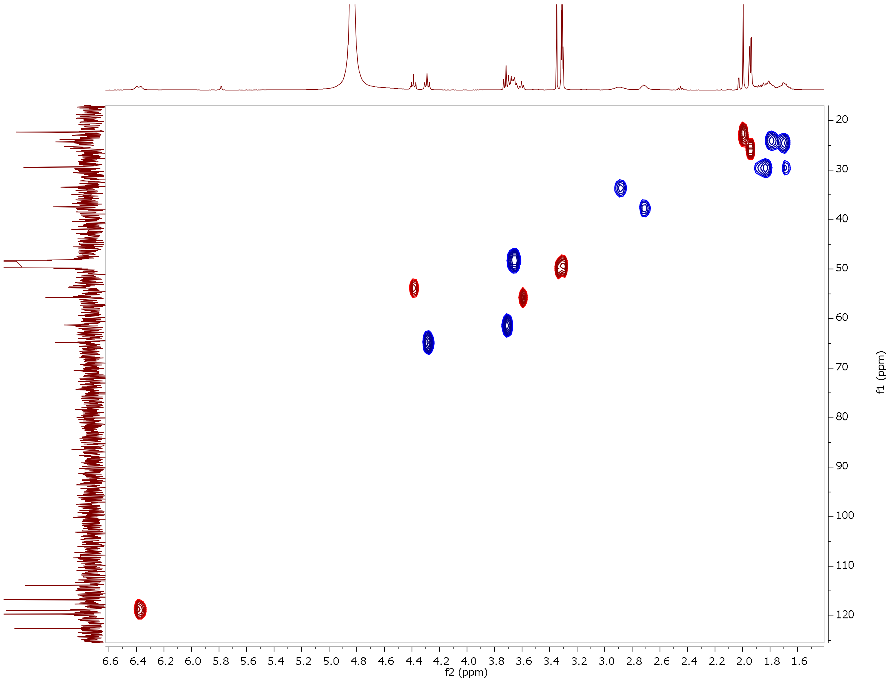

Figure S127. 2D-HSQC-NMR spectrum of *N*-acetyl-*Z*-L-fusarinine A (**8**) in CD_3OD

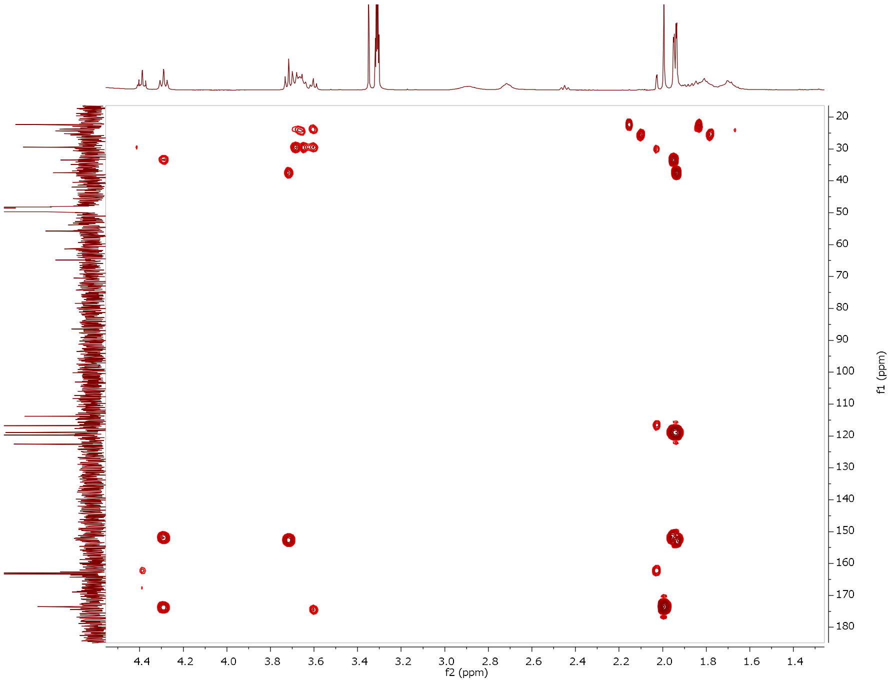

Figure S128. 2D-HMBC-NMR spectrum of *N*-acetyl-*Z*-L-fusarinine A (**8**) in CD_3OD

Table S29. NMR chemical shifts of N-acetyl-Z-L-fusarinine A (**8**) in $(CD_3)_2SO$ (400 MHz)

Position	δ_C [a] mult	δ_H [b] mult (*J* in Hz)	∫	HMBC (H→C#)	COSY (H→H#)
**1'**		8.37, d (7.4)	1	2', 14'	2'
**2**	53.80	3.28 - 3.25, m	1		4
**2'**	52.12	4.17 - 4.16, m	1	3'	4'
**3 3'**	172.29				
**4 4'**	28.06<br>28.66	1.59 - 1.51, m<br>1.65 - 1.61, m	2<br>2		2 2'
**5 5'**	23.17<br>22.32	1.59 - 1.51, m<br>1.65 - 1.61, m	2<br>2		6 6'
**6 6'**	46.45<br>46.62	3.55 - 3.48, m<br>3.62 - 3.60, m	2<br>2		5 5'
**8 8'**	165.98<br>166.61				
**9**	117.70	6.33, s	1	11, 16	16
**9'**	117.34	6.27, s	1	11', 16'	16'
**10**	149.63				
**10'**	151.06				
**11**	32.04	2.83 - 2.76, m	2	10, 12	12
**11'**	36.59	2.64 - 2.61, m	2	10', 12'	12'
**12**	63.44	4.15 - 4.13, m	2	10, 11	11
**12'**	59.80	3.55 - 3.48, m	2	10', 11'	11'
**14'**	169.80				
**15'**	22.64	1.84, brs	3	14'	
**16**	25.57	1.86, d (1.1)	3	9, 10, 11	9
**16'**	25.57	1.84, brs	3	9', 10', 11'	9'

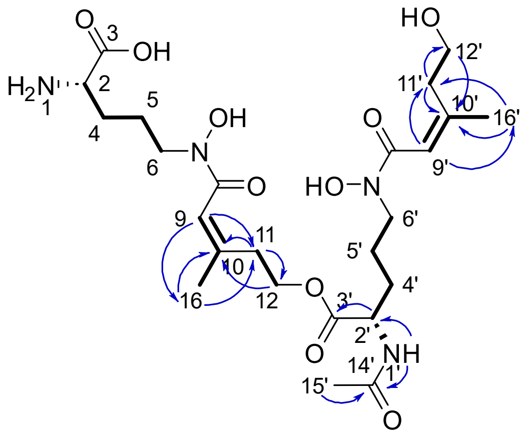

Figure S129. ^{1}H-^{1}H COSY (▬) and ^{1}H-^{13}C HMBC (→) correlations of *N*-acetyl-*Z*-L-fusarinine A (**8**) in $(CD_3)_2SO$

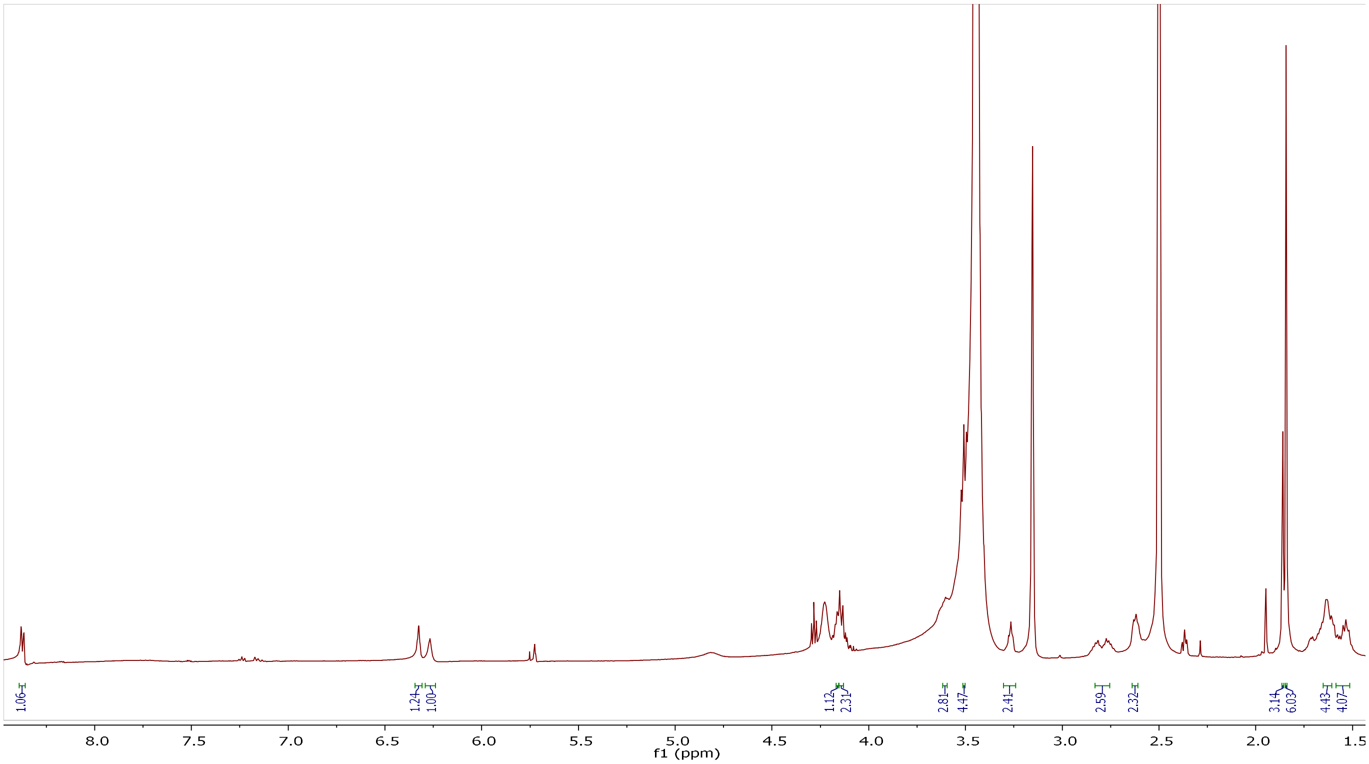

Figure S130. ^{1}H-NMR spectrum of *N*-acetyl-*Z*-L-fusarinine A (**8**) in $(CD_3)_2SO$ (400 MHz)

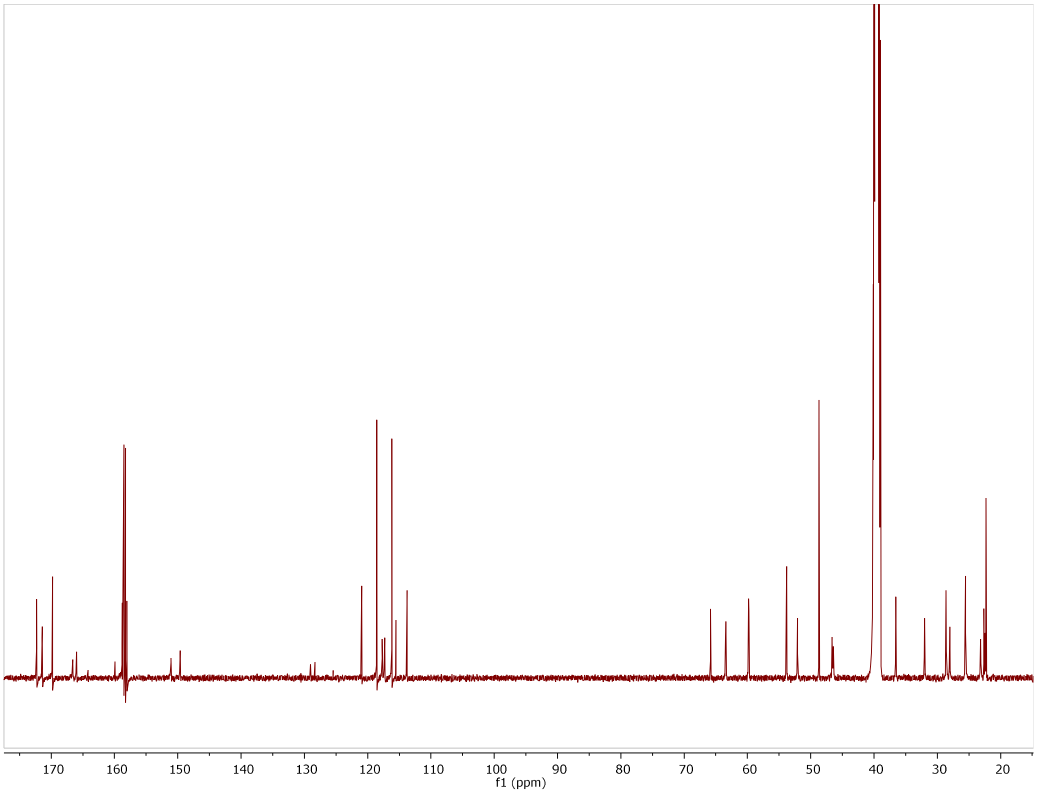

Figure S131. ^{13}C-NMR spectrum of *N*-acetyl-*Z*-L-fusarinine A (**8**) in $(CD_3)_2SO$ (100 MHz)

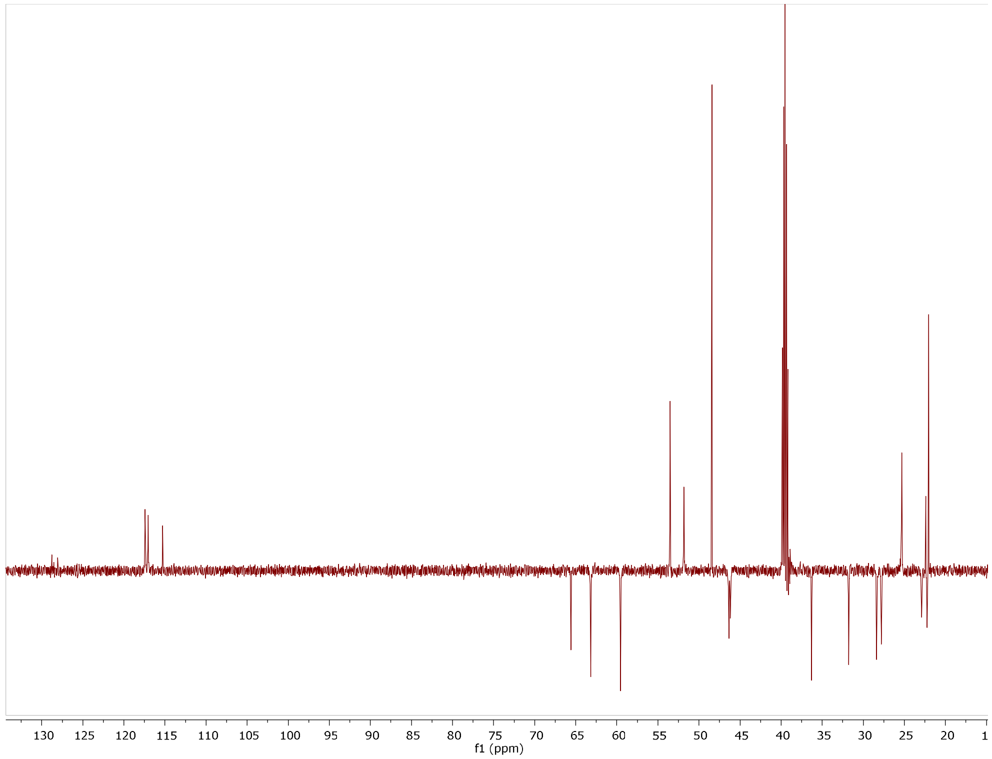

Figure S132. DEPT135-NMR spectrum of *N*-acetyl-*Z*-L-fusarinine A (**8**) in $(CD_3)_2SO$ (100 MHz)

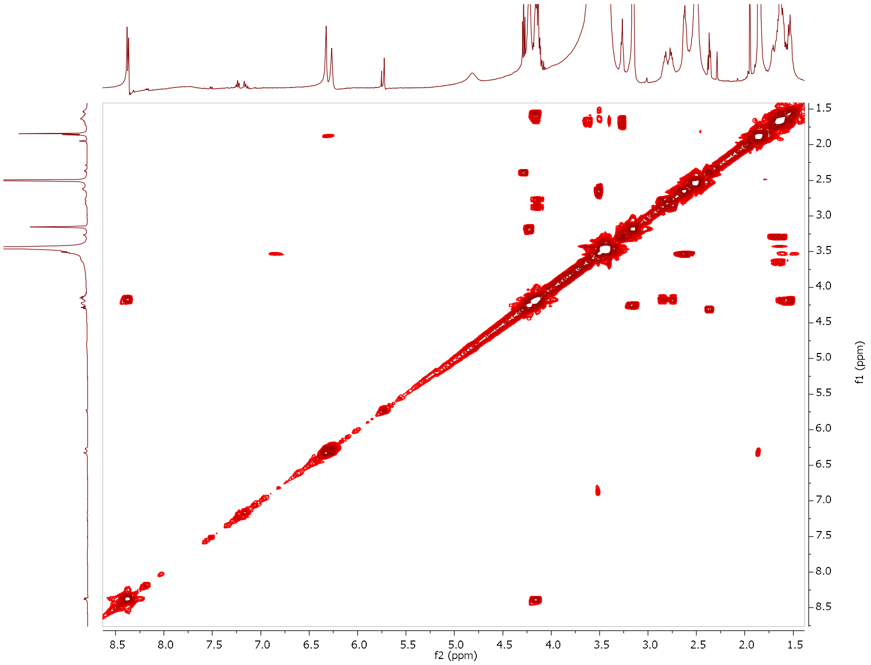

Figure S133. 2D-COSY-NMR spectrum of *N*-acetyl-*Z*-L-fusarinine A (**8**) in $(CD_3)_2SO$

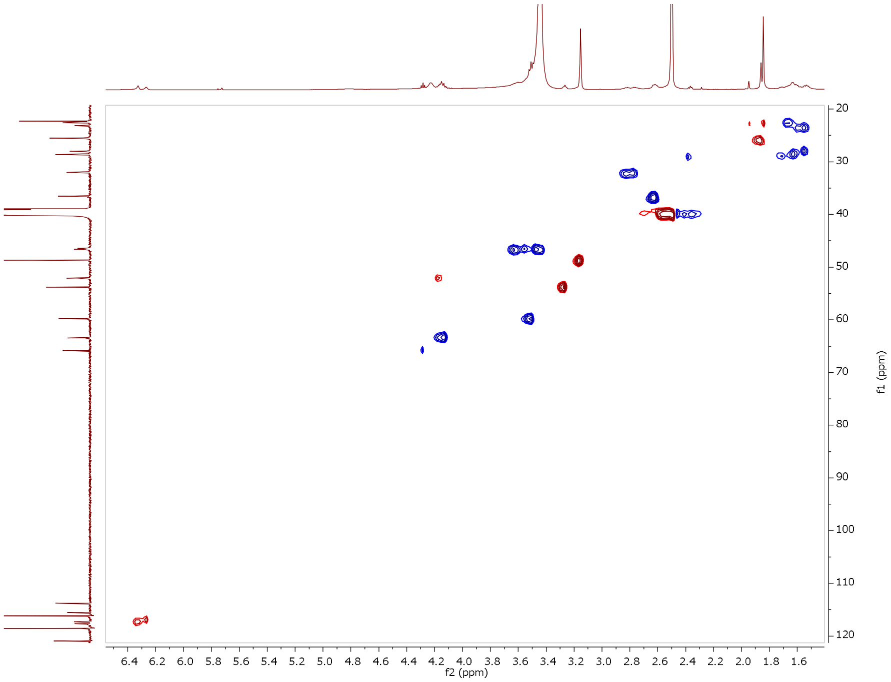

Figure S134. 2D-HSQC-NMR spectrum of *N*-acetyl-*Z*-L-fusarinine A (**8**) in $(CD_3)_2SO$

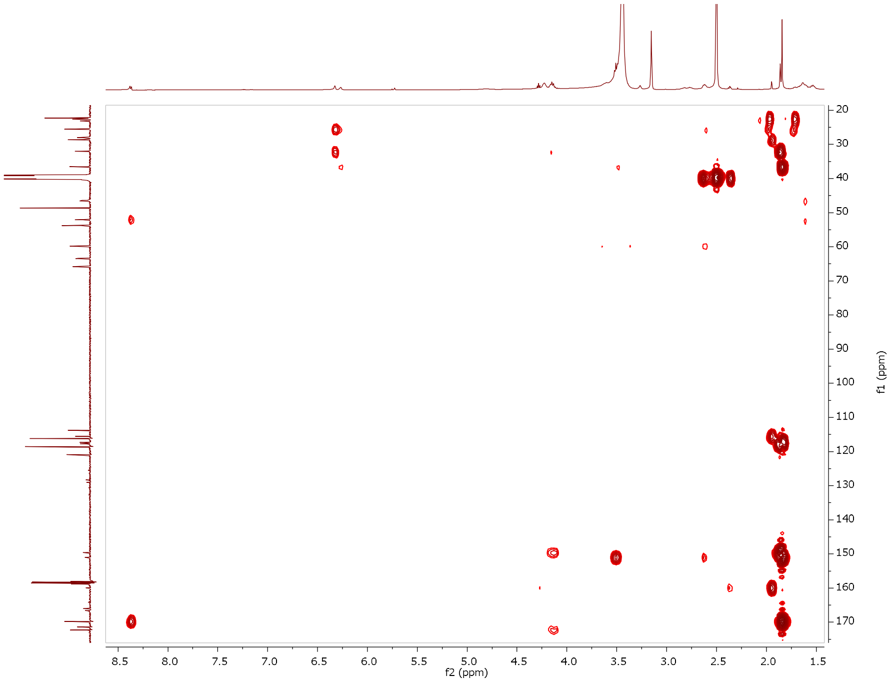

Figure S135. 2D-HMBC-NMR spectrum of *N*-acetyl-*Z*-L-fusarinine A (**8**) in $(CD_3)_2SO$

4-methyl-5,6-dihydro-2H-pyran-2-one

Table S30. Major *m/z* ions in the (+) HRMS-ESI of 4-methyl-5,6-dihydro-2H-pyran-2-one

Adduct	*m/z* observed	*m/z* calculated	Error (ppm)
$[M+Na]^+$	135.0416	135.0416	-

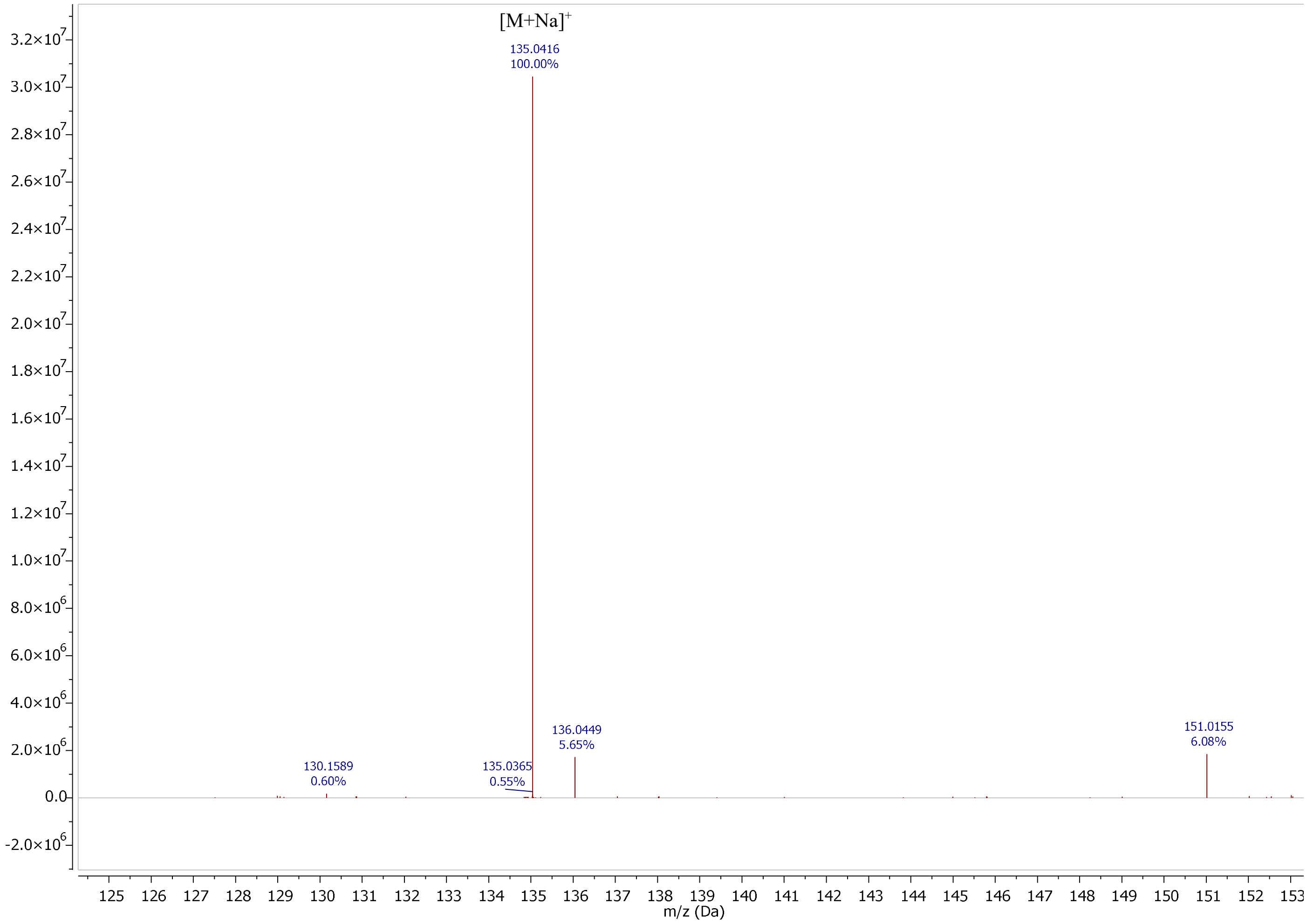

Figure S136. (+) HRMS-ESI of 4-methyl-5,6-dihydro-2H-pyran-2-one

Table S31. NMR chemical shifts of 4-methyl-5,6-dihydro-2H-pyran-2-one in CD_3OD (400 MHz)

Position	δ_C [a] mult	δ_H [b] mult (*J* in Hz)	∫	HMBC (H→C#)	COSY (H→H#)
**2**	167.86				
**3**	116.52	5.78, H (1.5)	1	2, 5, 7	5, 7
**4**	162.17				
**5**	29.55	2.45, ddt (7.2, 5.4, 1.5, 0.8)	2	3, 4, 6, 7	3, 6, 7
**6**	67.62	4.39, t (6.3)	2	2, 4, 5	5
**7**	22.99	2.03 dt (1.6, 0.8)	3	3, 4, 5	3, 5

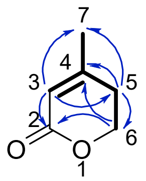

Figure S137. ^{1}H-^{1}H COSY (▬) and ^{1}H-^{13}C HMBC (⟶) correlations of 4-methyl-5,6-dihydro-2H-pyran-2-one in CD_3OD

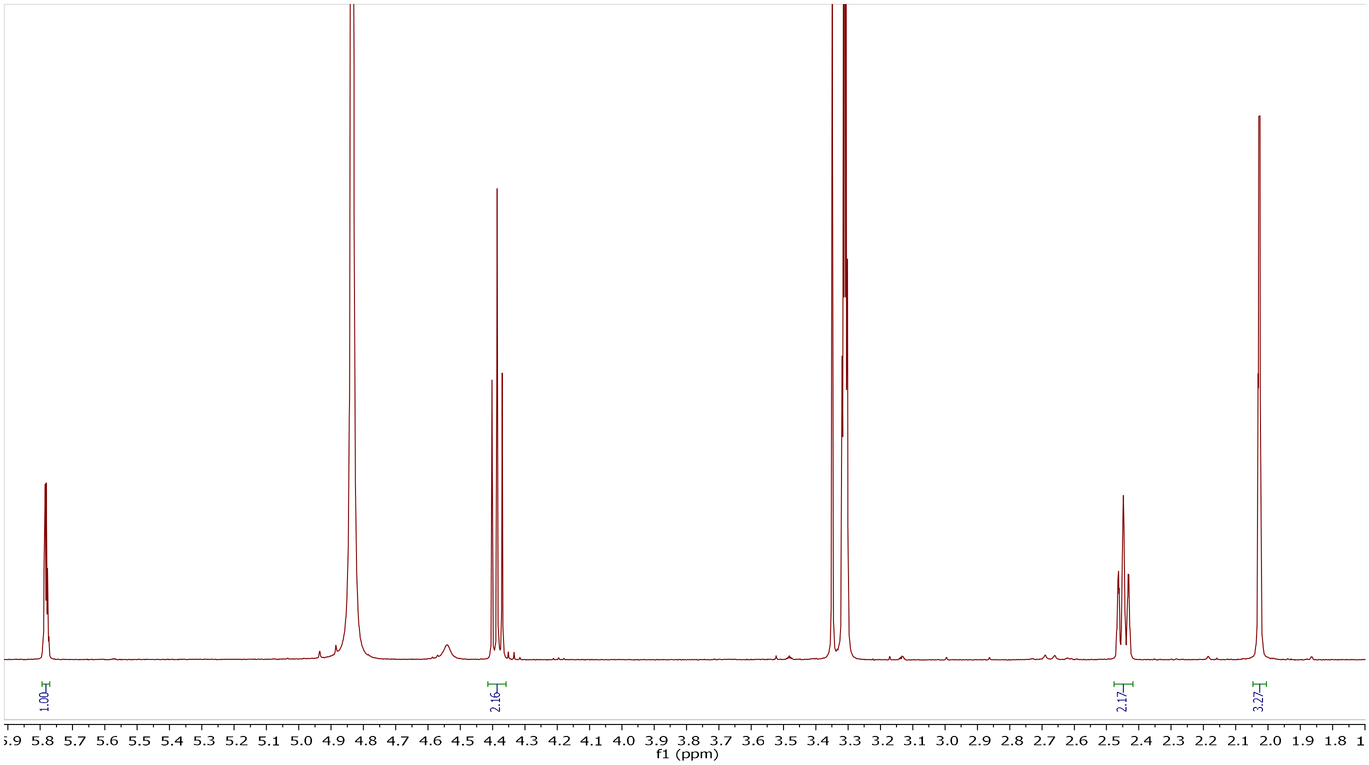

Figure S138. ^{1}H-NMR spectrum of 4-methyl-5,6-dihydro-2H-pyran-2-one in CD_3OD (400 MHz)

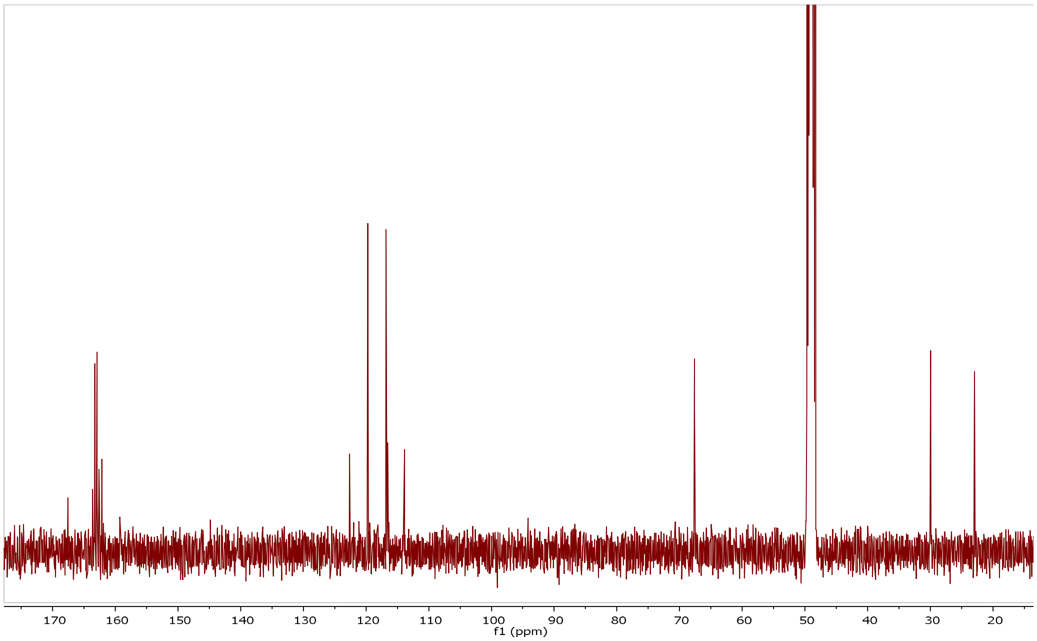

Figure S139. ^{13}C-NMR spectrum of 4-methyl-5,6-dihydro-2H-pyran-2-one in CD_3OD (100 MHz)

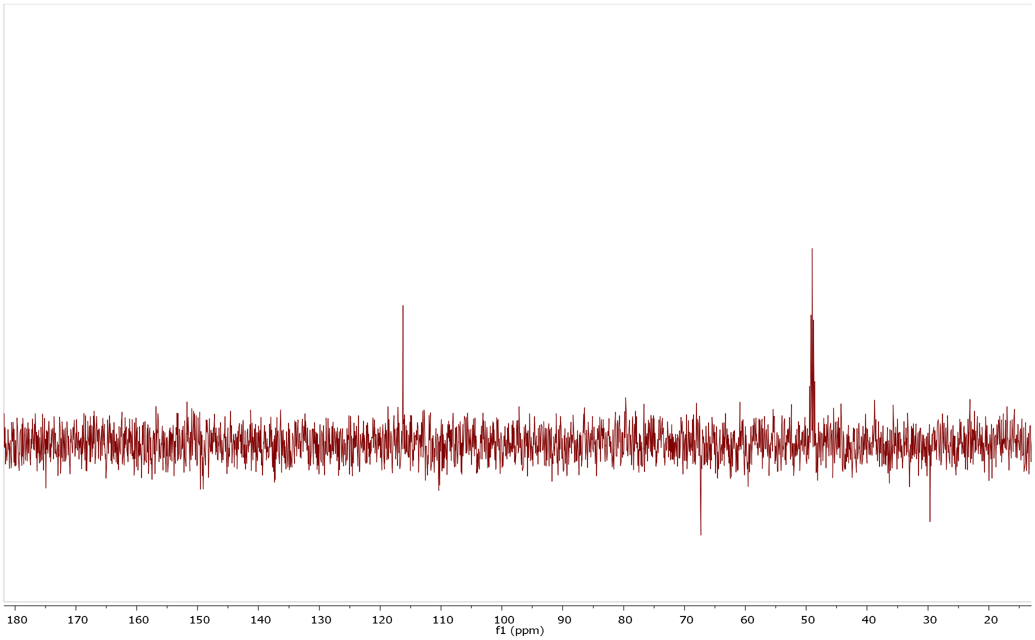

Figure S140. DEPT135-NMR spectrum of 4-methyl-5,6-dihydro-2H-pyran-2-one in CD_3OD (100 MHz)

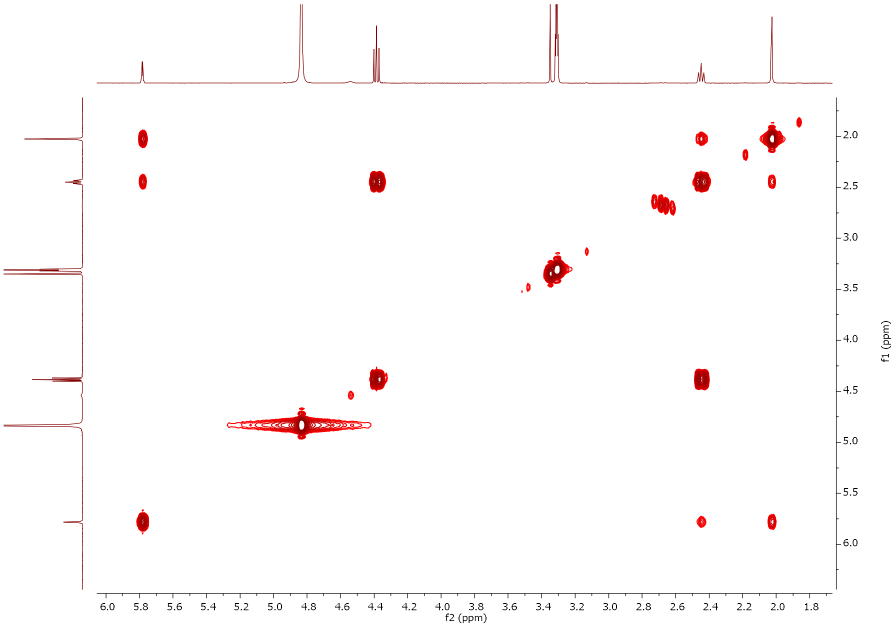

Figure S141. 2D-COSY-NMR spectrum of 4-methyl-5,6-dihydro-2H-pyran-2-one in CD_3OD

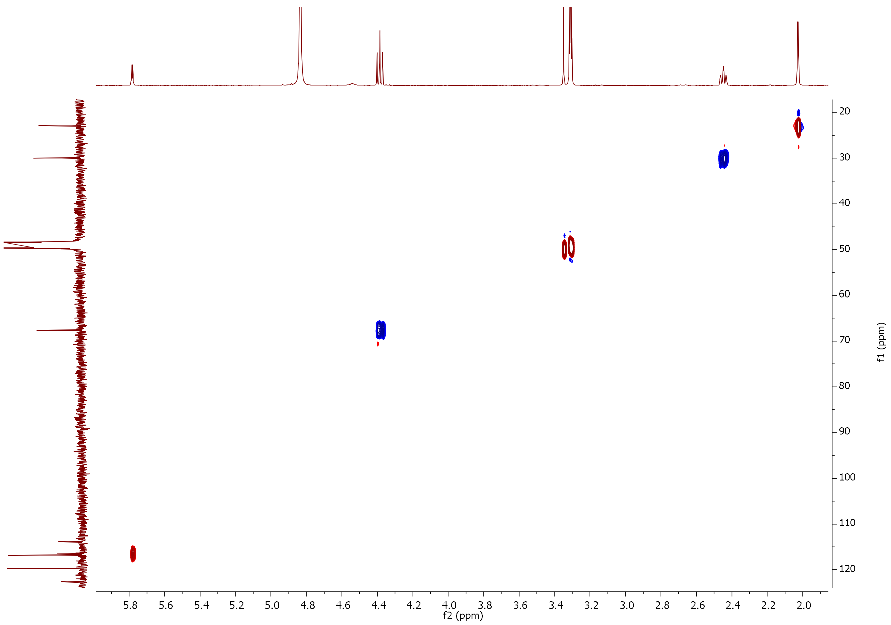

Figure S142. 2D-HSQC-NMR spectrum of 4-methyl-5,6-dihydro-2H-pyran-2-one in CD_3OD

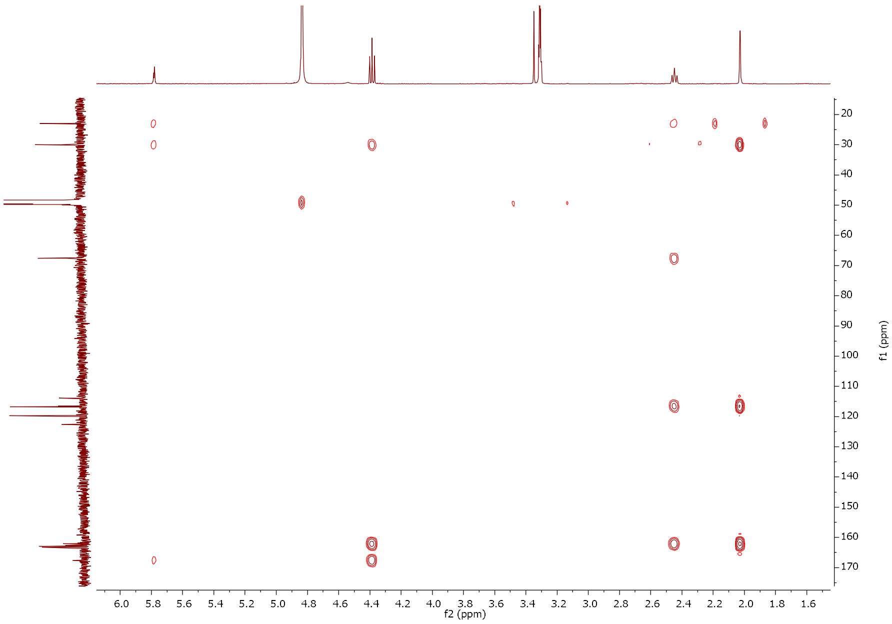

Figure S143. 2D-HMBC-NMR spectrum of 4-methyl-5,6-dihydro-2H-pyran-2-one in CD_3OD

Marfey's reaction

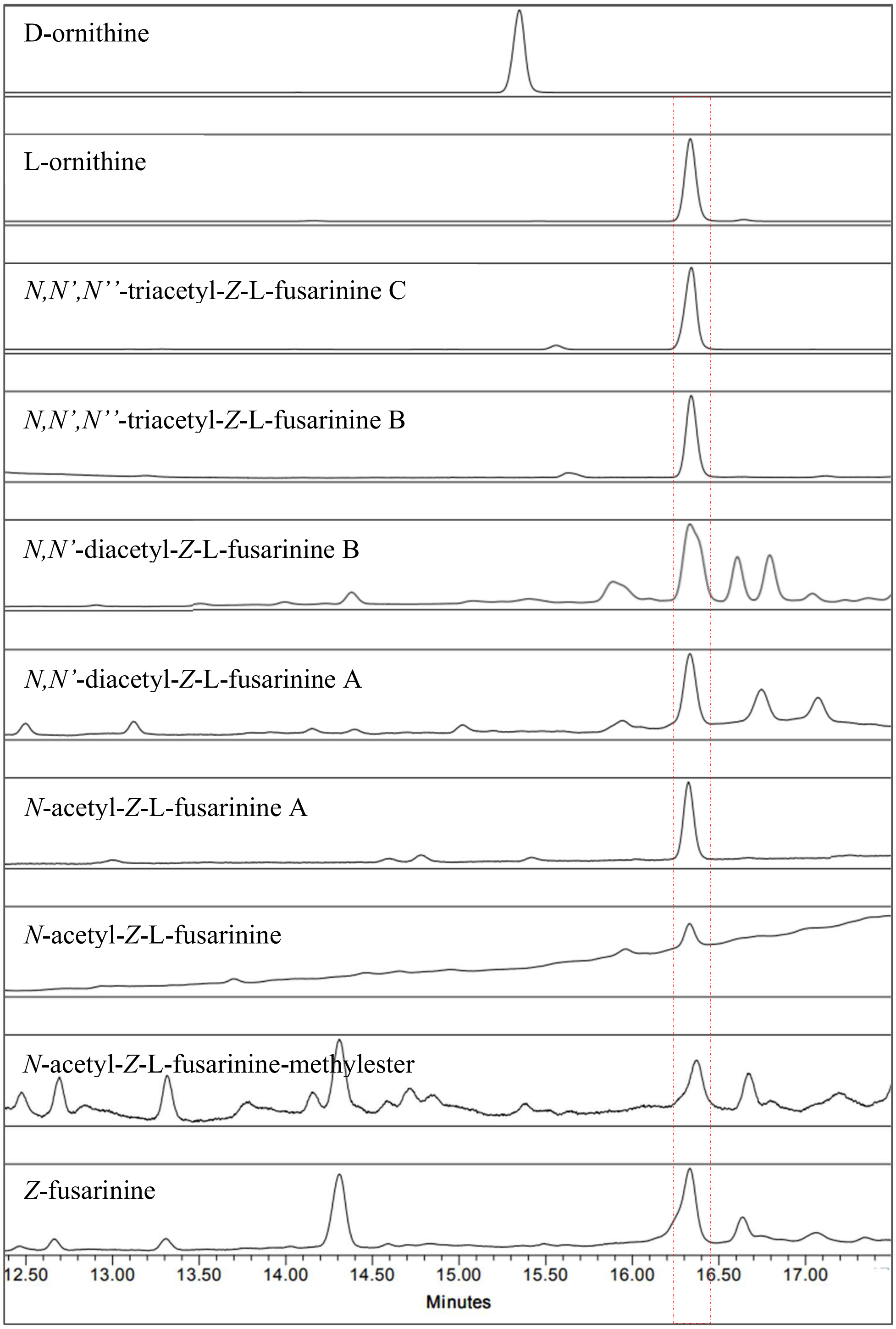

Figure S144. Analysis by advance Marfey´s method of 1-8: HPLC chromatograms with UV detector at 360 nm showing the L-FDAA derivatives of D- and L-ornithine standards and the hydrolyzed products isolated from the fungus P. verrucosus FAE27. Diastereoisomers with a retention time of 16.3 min, corresponding to those formed with L-ornithine, are highlighted with a red box.
